# Simulated digestions of free oligosaccharides and mucin-type *O*-glycans reveal a potential role for *Clostridium perfringens*

**DOI:** 10.1038/s41598-023-51012-4

**Published:** 2024-01-18

**Authors:** Andrew G. McDonald, Frédérique Lisacek

**Affiliations:** 1 Proteome Informatics Group, SIB Swiss Institute of Bioinformatics, 1211 Geneva, Switzerland; 2 School of Biochemistry and Immunology, Trinity College Dublin, Dublin 2, Ireland; 3 Computer Science Department, University of Geneva, Geneva, Switzerland; 4 Section of Biology, University of Geneva, Geneva, Switzerland

**Keywords:** Hydrolases, Glycobiology, Computational biology and bioinformatics, Dysbiosis, Microbiome, Symbiosis, Infant necrotizing enterocolitis

## Abstract

The development of a stable human gut microbiota occurs within the first year of life. Many open questions remain about how microfloral species are influenced by the composition of milk, in particular its content of human milk oligosaccharides (HMOs). The objective is to investigate the effect of the human HMO glycome on bacterial symbiosis and competition, based on the glycoside hydrolase (GH) enzyme activities known to be present in microbial species. We extracted from UniProt a list of all bacterial species catalysing glycoside hydrolase activities (EC 3.2.1.-), cross-referencing with the BRENDA database, and obtained a set of taxonomic lineages and CAZy family data. A set of 13 documented enzyme activities was selected and modelled within an enzyme simulator according to a method described previously in the context of biosynthesis. A diverse population of experimentally observed HMOs was fed to the simulator, and the enzymes matching specific bacterial species were recorded, based on their appearance of individual enzymes in the UniProt dataset. Pairs of bacterial species were identified that possessed complementary enzyme profiles enabling the digestion of the HMO glycome, from which potential symbioses could be inferred. Conversely, bacterial species having similar GH enzyme profiles were considered likely to be in competition for the same set of dietary HMOs within the gut of the newborn. We generated a set of putative biodegradative networks from the simulator output, which provides a visualisation of the ability of organisms to digest HMO and mucin-type *O*-glycans. *B. bifidum*, *B. longum* and *C. perfringens* species were predicted to have the most diverse GH activity and therefore to excel in their ability to digest these substrates. The expected cooperative role of Bifidobacteriales contrasts with the surprising capacities of the pathogen. These findings indicate that potential pathogens may associate in human gut based on their shared glycoside hydrolase digestive apparatus, and which, in the event of colonisation, might result in dysbiosis. The methods described can readily be adapted to other enzyme categories and species as well as being easily fine-tuneable if new degrading enzymes are identified and require inclusion in the model.

## Introduction

Human milk oligosaccharides (HMOs) are a class complex unconjugated sugars derived from lactose, and which is the third-most abundant component of milk, after lactose itself, and lipids, with their concentration being at their highest in colostrum^1^. Although undigested by the neonate, HMOs are thought to provide protection from pathogenic microorganisms by masking the epithelial cells of the developing intestinal mucosa^2,3^, promotion of mucin expression^4^ and by promoting the growth of lactobacilli and bifidobacteria specialising in their digestion^5^. Aside from protecting against necrotising enterocolitis^6^ or Streptococcus group B organisms^7^, HMOs may also provide other benefits to the neonate, including in neurodevelopment^8,9^ and prevention of allergic diseases^10–12^. The development of a healthy gut microbiome, as a consequence of human milk oligosaccharides in the diet, has indications of longer-term benefit to the infant, including reduced incidence of inflammatory bowel diseases^13^, neuroprotective effects^14^ and small-molecule homeostasis^15^. In this context, understanding the principles of HMO synthesis as well as the conditions upon which HMOs are potentially digested by the infant gut^16^, is a step towards answering the broad range of open questions. Previously, we have modelled the synthesis of the HMO portion of human milk and ran simulations that could account for around 90% of all structures observed and reported in the literature^17^. The logical follow-on is to consider the HMO degradation by the gut microbiome.

As evidence of the prominent role of carbohydrate-active enzymes (cazymes) in digestion of dietary and host oligosaccharides, 95% of the genomes contained in CAZy, the database in which those enzymes have long been collected and grouped into families^18^, as well as 85% of its entries, are from the superkingdom of Bacteria. Novel enzyme activities are usually inferred, both from genomic and^19^ metagenomic studies^20^ of these organisms. The IUBMB Enzyme List, with its Enzyme Commission (EC) number hierarchy, provides a functional classification of enzymes based on experimental evidence of the reactions they catalyse^21^, a classification which is independent of the specific protein or organism. The manually curated association between EC numbers and CAZy families is a source of identification of carbohydrate synthesis/degradation effectors. Genome automatic annotation that assigns protein function based on a broad variety of criteria, provides an independent starting point for selecting these cazymes. In the past decade, numerous studies have covered the analysis of the cazyme content in the gut microbiome as reviewed in^19,22,23^ where the focus is mostly on cazymes involved in catabolism by Bacteria. These are generally confined to the sub-subclass of glycosidases (GH family), classified under EC 3.2.1.-, and occasionally as polysaccharide lyases (PL family), which are classified under EC 4.2.2.-.

In the present account, we report on a new simulator of 13 hydrolase enzymes known to be possessed by gut microflora^24–26^, which we have used to investigate the potential of microbes to digest either HMOs or the glycoconjugates of the intestinal mucosa, and the possibility of cross-feeding^25,27^ and syntrophy^28^. We queried the UniProt protein database for the available evidence for proteins involved in glycoside hydrolase and sulfatase activity, correlated these with their corresponding EC numbers, and identified the CAZy families into which they are organised. Through simulated digestions of populations of HMOs and mucin-type (GalNAc-linked) *O*-glycans, we estimated the likelihood of different taxa and individual bacteria to grow on these classes of substrate. Previous attempts to simulate HMO catabolism have been made^29^ but were mostly based on the occurrence of specific (orthologous) genes in bacterial genomes.

Within a similar framework as that presented in^17^ for HMO biosynthesis, we generated a set of putative networks from the output of simulating HMO degradation. Network visualisation was kept consistent in terms of colour coding of HMO categories. A closer look at species distribution and contribution expectedly revealed *B. bifidum*, *B. longum* as having the most diverse GH activity. More surprisingly, *C. perfringens*, in the present study, is associated with similar capacities as the Bifidobacter species, which might have implications for the involvement of this pathogen in the development of necrotising enterocolitis^30^.

## Results

**Figure 1:**
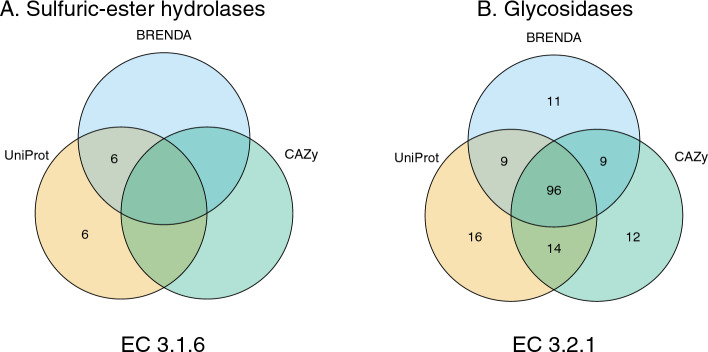
Numbers of distinct bacterial sulfatase (EC 3.1.6.-) and glycoside hydrolase (EC 3.2.1.-) activities in UniProt, cross-referenced with the BRENDA database, and CAZy. Sulfatases not being exclusively carbohydrate-active, no members of EC 3.1.6 are listed in the CAZy database. The BRENDA values were not sourced directly from the BRENDA database but represent EC numbers that are cross-referenced in UniProtKB.

Bacterial proteins associated with activities in sub-subclasses EC 3.2.1 (glycoside hydrolases) and EC 3.1.6 (sulfuric-ester hydrolases) were extracted from UniProt (Release 2023_02) as described in the Methods. Similar data were extracted from CAZy (May 2023 update) for EC 3.2.1. Amongst these proteins 155 complete EC numbers were found, when cross-referenced with the BRENDA^31^ repository, of which 30 were unique to UniProt and 20 were unique to BRENDA (Fig. 1B). Twelve proteins associated with sulfuric-ester hydrolase (sulfatase) activity were found, all of which were in UniProt, and six of which were in BRENDA (Fig. 1A).

### Enzymes and reactions

A Glycologue simulator of 12 glycoside hydrolases and 1 sulfatase was developed, based on their known involvement in HMO and mucin digestion by gut floral bacteria^24,25,32^. The enzymes of the model are shown in Table 1, numbered **1**–**13**, with the EC number, where available, comprising one sialidase (enzyme **1**), two galactosidases (**2** and **3**), two *N*-acetylgalactosaminidases (**4**, **8**), two *N*-acetylglucosaminidases (**5**, **13**), one β-*N*-acetylhexosaminidase (**7**), one sulfatase (**9**), three fucosidases (**6**, **10**, **11**) and the endo-glycosidase, lacto-*N*-biosidase (**12**). The corresponding activities were modelled in Glycologue^33^ notation, which uses a single-character encoding system for the monosaccharides. Reactions and glycan substrates were cross-checked with ChEBI^34^ and Rhea. Rhea accession numbers, where available, are included in Table 1.

**Table 1:** Enzymes of the model.

Enzyme no	EC number	Short name	Accepted name	CAZy families	Rhea ID
1	EC 3.2.1.18	exo-αSiaH	exo-α-sialidase	GH33,34,83, 177,181	–
2	EC 3.2.1.22	αGalH	α-galactosidase	GH4,27,31, 36,57,97,110	21,112
3	EC 3.2.1.23	βGalH	β-galactosidase	GH1,2,3,5,16,35,42,50,54,147,165,173	–
4	EC 3.2.1.49	αGalNAcH	α-*N*-acetylgalactosaminidase	GH27,36,109,129	15,085
5	EC 3.2.1.50	αGlcNAcH	α-*N*-acetylglucosaminidase	GH89	–
6	EC 3.2.1.51	αFucH	α-l-fucosidase	GH29,95,151	12,288
7	EC 3.2.1.52	βHexNAcH	β-*N*-acetylhexosaminidase	GH2,3,18,20,84,179	–
8	EC 3.2.1.97	endo-αGalNAcH	endo-α-*N*-acetylgalactosaminidase	GH101	30,983/54,540
9	EC 3.1.6.-	exo-sulfoH	(3S/4S/6S-Gal/GlcNAc sulfatase)	–	–
10	EC 3.2.1.63	α2FucH	1,2-α-l-fucosidase	GH95	10,816
11	EC 3.2.1.111	α3/4FucH	1,3-α-l-fucosidase	GH29	–
12	EC 3.2.1.140	LNBase	lacto-*N*-biosidase	GH20,136	21,568
13	EC 3.2.1.-	β6GlcNAcH	(6-β-*N*-acetylglucosaminidase)	–	–

Glycoside hydrolases included in the Glycologue simulator.

^a^A sulfatase enzyme (**9**) and 6-β-*N*-acetylglucosaminidase (**13**) are listed but were unused.

### CAZy families and organism taxonomic classification

From the UniProt dataset (Release 2023_02) 95 distinct CAZy glycoside hydrolase families were identified in Bacteria. The number of distinct simulated EC numbers available to individual species were counted. No organism was found to have all 11 fully characterised enzymes of the simulator, based on the assignment of a complete EC number in UniProt or BRENDA, the highest ranked being *Bifidobacterium bifidum*, with 10 full EC numbers associated with its GH-encoding proteins.

Of the 8368 bacterial species associated with glycoside hydrolase (GH) or sulfuric-ester hydrolase (SH) activities in UniProt/BRENDA, 448 were manually positively identified with human gut, the remaining 7920 being classed as non-gut species (often from environmental samples). A subset of 166 of the gut species have been identified as potential pathogens^35^.

From Table 1 an enzyme profile could be assigned to each species based on the presence or absence of a particular enzyme activity in the UniProt data. This enzyme profile value is an integer representing the state of enzyme activities (see Methods, Enzyme profiles), represented through this work as pX, with X an integer from 0 to ${\sum }_{i=1}^{13}{2}^{i-1}$= 8191. From the gut bacteria, 40 unique enzyme profiles were identified, compared with 79 profiles in non-gut species. Interactive Krona^36^ SVG (Scalable Vector Graphics) diagrams of gut, non-gut and all species (combined) are shown in Supplementary Information. On comparing the gut and non-gut microbial subsets, 4 enzyme profiles unique to gut species were identified, and 12 were unique to non-gut bacteria. The median numbers of simulated characterised hydrolytic enzymes available to non-gut (*n* = 7920) and gut (*n* = 448) species were found to be 2 and 3, respectively.

### Feeding HMOs to a simulated microbiome

**Figure 2:**
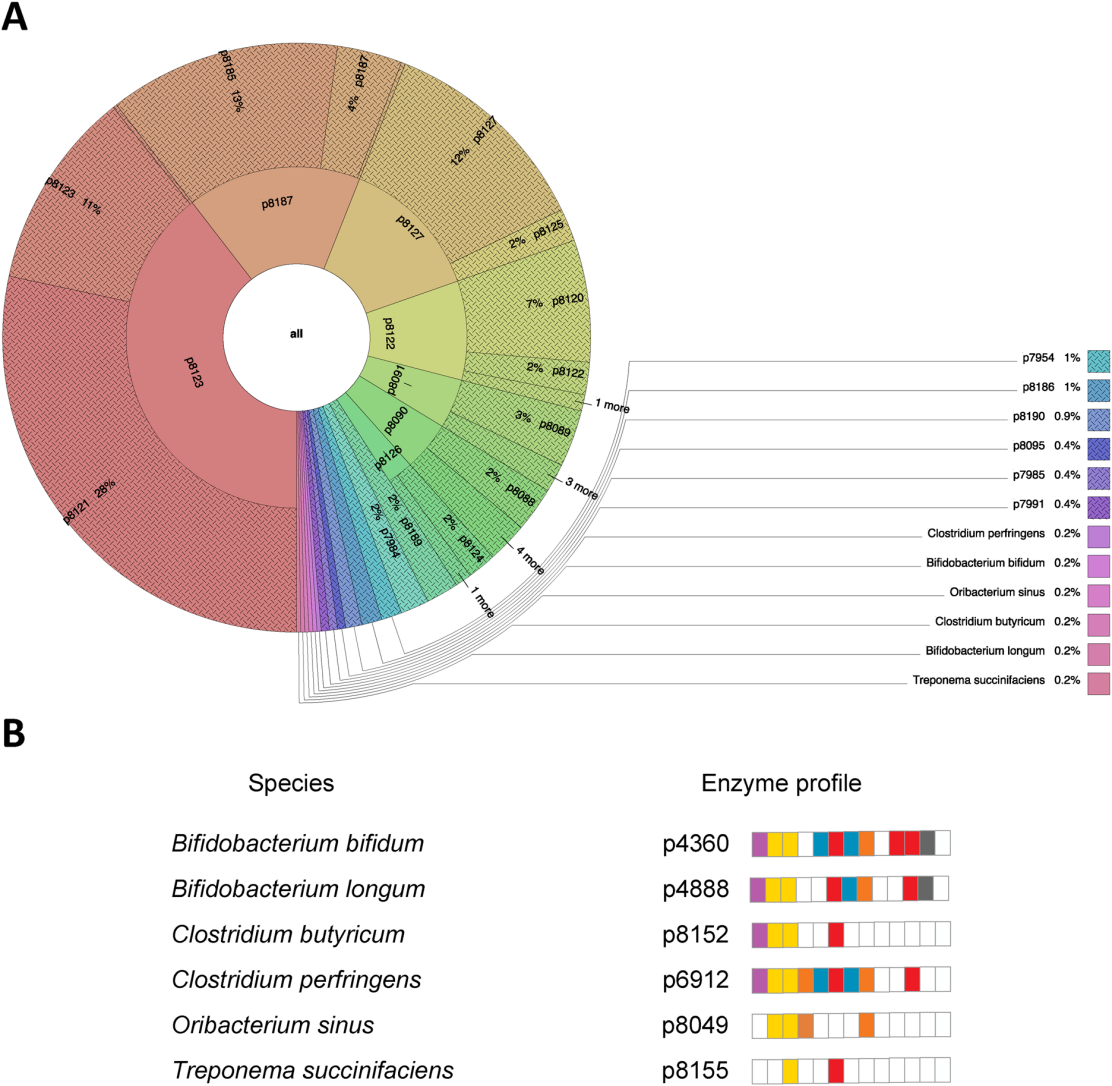
Mapping the enzymes used in carbohydrate (HMO) digestion to the full complement of simulated glycoside hydrolase enzymes available to an organism. (**A**) Krona diagram showing the GH enzyme profiles on the inner ring, which reflect simulated digestion of a heterogeneous population of HMOs, map to one or more profiles of available enzymes in the outer ring, which corresponds with the bacterial species. (**B**) Unique enzyme profile values belonging to the six named spaces in (**A**), *Bifidobacterium bifidum*, *Bifidobacterium longum*, *Clostridium butyricum*, *Clostridium perfringens*, *Oribacterium sinus* and *Treponema succinifaciens*. The enzyme profile value is illustrated by a vector of cells (**1** to **13**, left to right, representing the enzymes of Table 1) filled according to a colour representing the substituent removed, or bond cleaved: red (l-fucose); yellow (d-galactose); orange (GalNAc); blue (GlcNAc/HexNAc) magenta (sialic acid); grey (lacto-*N*-biose); white (no activity). A complete set of enzyme profile values is shown in Fig. 9, while interactive Krona diagrams are provided in Supplementary Information (Krona), in which each segment can be expanded to reveal the species associated with each profile.

A library of 226 experimentally determined HMOs^17^ was used as input to the Glycologue GH simulator, for every species within each subset of the species data. Every GH reaction was associated with one bit of information from the innate enzyme profile value. Once all substrates had been digested, as far as possible, within the simulator, the subset of the enzymes used on those substrates was stored as digestion profile value. Not all enzyme activities of the model were activated by the HMO set. For example, three species, *Bacteroides caccae*, *Adhaeribacter pallidiroseus* and *Zobellia galactanivorans*, express enzymes **1** through **8** (see Table 1), which is the enzyme profile value p7936. After simulated digestion, since none of the HMOs possessed an α-galactose or α-*N*-acetylglucosaminidase residue, the enzymes corresponding to the removal of these sugar units, namely α-galactosidase and α-*N*-acetylglucosaminidase were never activated, resulting in a further elimination of enzymes **2** and **5**. The substrate-specific, or environment-specific, profile value for these bacteria was therefore calculated to be p7954. The mapping of innate profile values to HMO-trained profile values is summarised in Fig. 2. Six species were discovered to possess unique subsets of the modelled enzymes, including the probiotic species *B. bifidum* and *B. longum*, but also two potential pathogens, *C. perfringens* and *Oribacterium sinus*.

**Figure 3:**
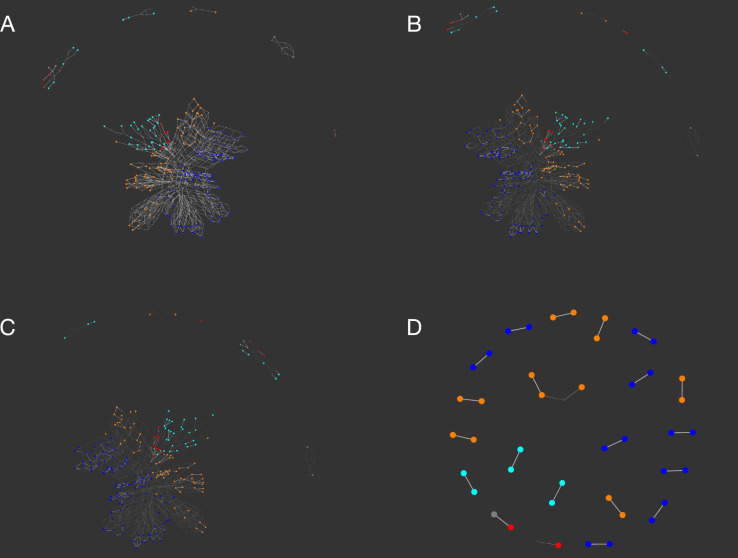
Simulated networks of HMO degradation by glycoside hydrolases expressed in Bacteria when each of 226 unique human milk oligosaccharides were submitted to the simulator. (**A**) Network obtained with all enzymes of the model available. (**B**) Networks corresponding to the enzyme profiles p4360 and p4888, representing *Bifidobacterium* spp. *bifidum* and *longum*, respectively. (**C**) Network p6912 (*Clostridium perfringens*). (**D**) Network p7984 (*E. coli* and other species). Between panels (**A**–**D**), increased fragmentation of the networks occurs, as the number of available GH enzymes active towards HMO substrates decreases (**B**–**D**). Nodes are coloured according to type of core structure based on the reducing end of the HMO^17^: red (lacto-*N*-tetraose), cyan (lacto-*N*-neotetraose), blue (lacto-*N*-hexaose), orange (lacto-*N*-neohexaose), grey (other).

**Figure 4:**
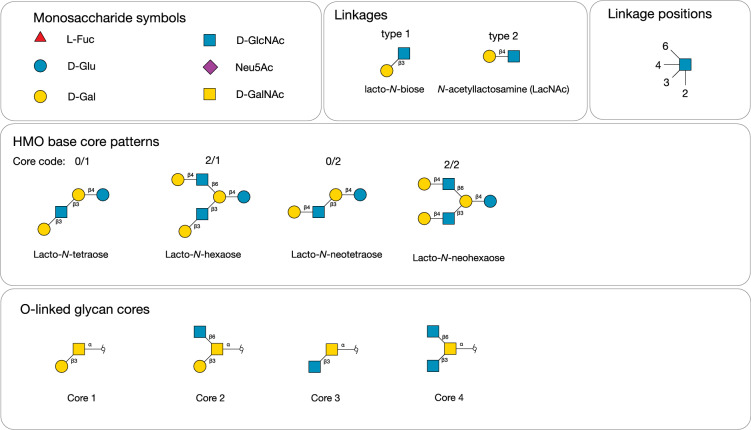
Symbols of common monosaccharides and cores found in human milk oligosaccharides and mucin-type *O*-glycans.

Simulated networks of the digestion of the HMOs are shown in Fig. 3. Nodes that were part of the experimentally determined HMO set are drawn at a larger node size than the intermediates and coloured according to the core type (Fig. 4) to be consistent with the representation previously used^17^ for HMO biosynthesis. Complete digestion of all HMOs to lactose was not possible with the current version of the GH enzyme simulator, owing to its inability to process 3-fucosylated lactose (3-FL). Nevertheless, both the size of the networks and the number of disjoint subnetworks could be matched with certain profile values. The number of reaction subnetworks is seen to increase, as the numbers of enzymes available to an organism decrease (Fig. 3A–D; see also Supplementary Information). The most complete digestion profile, with all GH enzymes active, is shown in Fig. 3A), with a single major network of hydrolase reactions, and several smaller islands. The network for the species *B. bifidum* and *B. longum* was partitioned into two large and five small subnetworks (Fig. 3B). (Fig. 3B), which was closely matched by the *C. perfringens* enzyme profile (Fig. 3C). A putative digestive network that is representative of several species is shown in Fig. 3D, including *E. coli*, but also 24 other species, of which 7 were associated with the gut microbia that include two other species from the same order of Enterobacterales, *Enterobacter cloacae* and *Salmonella enterica*, two from Bacteroidales (*Parabacteroides distasonis* and *Bacteroides stercoris*) and two within Eubacteriales (*Eisenbergiella tayi* and *Blautia wexlerae*). With 5 GH enzyme activities available, compared with 8 and 10 activities in panel B, the graph is noticeably more fragmented than those of Bifidobacteria, indicating only partial digestion of the HMO substrates. Other HMO-degradation networks are shown in Supplementary Information (Networks).

**Figure 5:**
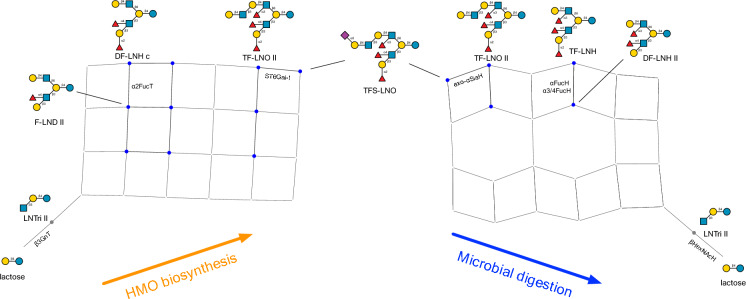
Simulated pathways of biosynthesis and glycoside hydrolase-catalysed degradation of the human milk oligosaccharide TFS-LNO. In the biosynthetic network, only products leading to TFS-LNO are included. Selected edges are labelled according to the enzymes assumed to be active at those steps of the pathway. Highlighted nodes represent experimentally characterised structures: grey node (LNTri II), blue nodes representing structures with a lacto-*N*-hexaose core (Fig. 4). Abbreviations used: LNTri II (lacto-*N*-triose II); TFS-LNO (a trifucosyl,monosialyllacto-*N*-octaose).

In Fig. 5, simulated networks of glycosyltransferase action during HMO biosynthesis^17^ are contrasted with the predicted networks of the glycoside hydrolases simulated in this study. The structure of TFS-LNO, a trifucosyl, monosialyl derivative of lacto-*N*-octaose^37,38^, was used as a reference. In both networks, several intermediates encountered have previously been characterised, and are highlighted in the figure, including F-LND III^39^, DF-LNH c^40,41^, TF-LNO II^39^, TF-LNH^40–42^ and DF-LNH II^40,42^. Not all paths through the networks are equally probable, and in the biosynthetic network, are regulated kinetically, through enzyme activities and their spatiotemporal localisations within Golgi^43^.

Differences in intermediates between biosynthetic and degradative pathways were also observed. When the computed intermediates of the HMO-Glycologue minimal biosynthetic networks, published previously^17^, were compared with those of the GH simulator acting on the same initial set of HMO substrates, the percentage of the intermediates that were common to both networks was found to be 59.1%, the differences being partly due to products terminating in 6-linked GlcNAc, as a result of the βHexNAcH activity (enzyme **7**) acting to remove β3-linked GlcNAc from a branched oligosaccharide.

### Potential energy scores

To quantify the effect of an innate glycoside hydrolase apparatus feeding on HMO substrates, and more readily to compare the ability of species to thrive on these dietary oligosaccharides, a scoring system was developed for each simulated digestion, as described in the Methods (Calculation of potential energy scores). First, a maximum score was determined for the digestion of 226 HMOs by the simulator with all enzymes active, and the scores of all unique enzyme profile values were assigned a potential energy (PE) score relative to this maximum, from 0 to 1.

**Figure 6:**
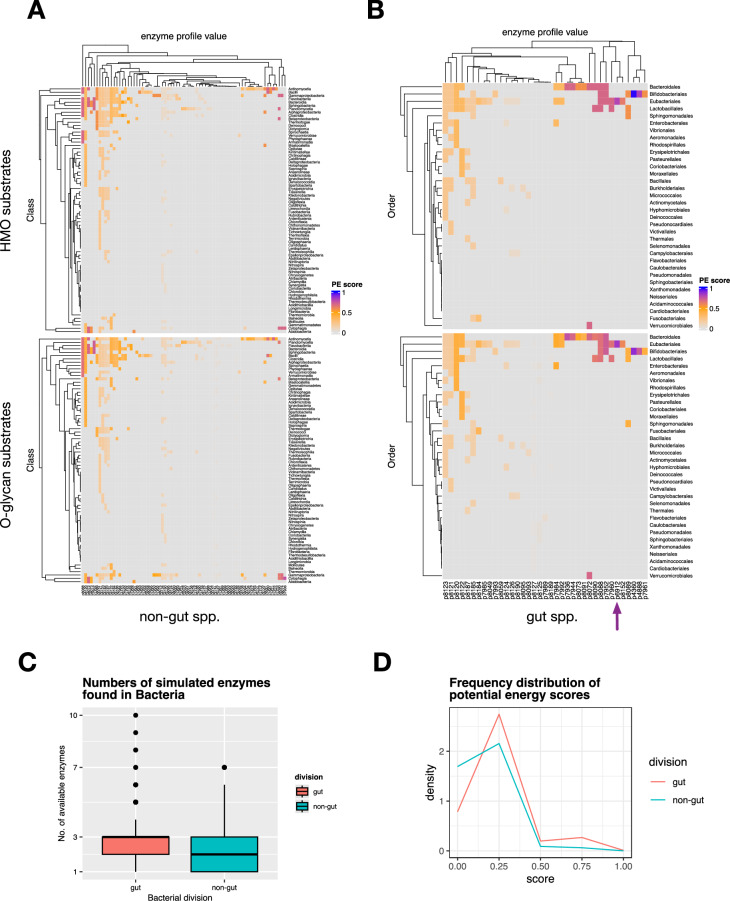
Predicted potential energy (PE) scores assigned to Bacteria fed a population of human milk oligosaccharides and mucin-type *O*-glycans. For each unique enzyme profile, with a subset of the simulated enzymes, a simulated glycoside hydrolase degradation network was generated, and the number of monosaccharides released was counted (the action of lacto-*N*-biosidase was counted as 0.5 instead of 1). Each profile was then scored based on the ratio of this value to the maximum possible value for the HMO dataset, with all enzymes active. Colours are assigned from grey through blue for the PE scores (0–1). (**A**) Non-gut species, grouped by taxonomic Class. (**B**) Gut bacterial species, grouped by taxonomic Order. The position of *C. perfringens* is indicated by an arrow. **C** Numbers of simulated enzymes available to gut and non-gut species. **D** Frequency distribution of potential energy scores of HMO-fed bacteria. Highly scoring gut species, fed in silico on HMOs (**A**, right) were *B. bifidum* (p4360), *B. longum* (p4888) and *C. perfringens* (p6912); the highest scoring on mucin-type *O*-glycans (**B**, right) were *B. bifidum* (p4360) and *C. perfringens* (p6912). Data are sourced from UniProtKB (https://uniprot.org). For comparison with the CAZy dataset, see Extended Data Fig. E2.

The highest scoring species in the gut subclass were *B. bifidum* and *B. longum,* with relative PE scores of 0.9888 and 0.8210, respectively, while *C. perfringens* scored 0.8089. These were coloured blue or purple in Fig. 6B (grouped at the level of taxonomic Order), while the closest scoring species were from the 7 bacteria referenced above (Fig. 3D), with a corresponding PE score of 0.6983, a value that was also achieved by several other GH enzyme profiles, such as p8072, which matched *Akkermansia muciniphila*, *Bacteroides reticulotermitis*, *Bacteroides xylanisolvens* and *Phocaeicola vulgatus*. Among the lowest scoring organisms on these substrates (PE value 0.0012) included *Enterococcus caccae* and *Salmonella typhi*, for which only two enzymes, α-galactosidase and β-*N*-acetylhexosaminidase, were retrieved from UniProt/BRENDA. For the 79 profiles in the non-gut bacterial subclass, PE scores ranged from 0.0007 (*Bacillus paranthracis*) to 0.7688 (*Adhaeribacter pallidiroseus* and *Zobellia galactanivorans*). The non-gut bacteria are grouped according to taxonomic Class in Fig. 6A. In both HMO and *O*-glycan simulated digestions, a similar pattern of clustering of profiles is evident, but the highest-performing species appear only in the gut category. Frequency distributions of potential energy scores in gut and non-gut Bacterial species are shown﻿ in Fig. 6D, along with the number of simulated enzymes available within each division.

### Simulated syntrophies

**Figure 7:**
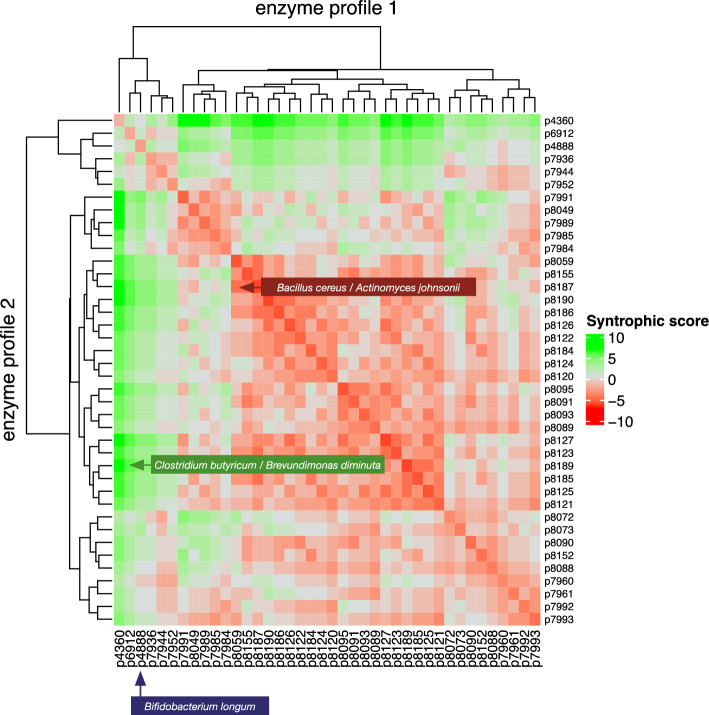
Predicted competition and symbiosis between gut bacteria, based on available enzyme profiles. For each bit in profiles *p*_1_ and *p*_2_, a syntrophic score increased by 1 when the bitwise comparison *p*_1_ XOR *p*_1_ = 1 (Boolean true), decreased by 0.5 when *p*_1_ AND *p*_2_ = 1 (Boolean true), and left unchanged when *p*1 AND *p*_2_ = 0 (Boolean false). A syntrophic pair (green) and competitive pair of species (red) are indicated. See text for details.

Bacteria expressing extracellular glycoside hydrolase enzymes can release substrates to the benefit of other species in a community, a process known as cross-feeding or syntrophy^27,28,44^. To investigate this phenomenon in silico, with the data in UniProt, we compared each enzyme profile in the simulator against the others, in pairwise fashion. Glycoside hydrolase enzyme profiles of the gut species were compared bitwise, as described in the Methods (Calculation of potential syntrophies) using Boolean exclusive-OR (XOR) and conjunction (AND). Where an enzyme activity was found to be lacking in one organism but present in the other, the score was incremented, as evidence of a potential collaboration, when supplied on substrates amenable to catabolism by that enzyme. Where the same enzyme activity was possessed by both profiles (each profile representing one or more species) the score was decremented, as an indication of the potential for competition on the same substrates. Enzymes absent from both profiles were treated neutrally. Thus, it was possible to assign an overall score for each pair, from the GH data in UniProt and BRENDA alone, without the need for simulated digestion. The potential syntrophic scores are illustrated in Fig. 7, where potential collaborations are represented as green pixels, competitors as red, and neutral interactions as white. Intraspecies competition^45^ is represented by the diagonal, in which identical profile values are compared.

Syntrophic scores (*x*) were found to range from − 6.0 to + 8.0. A general feature of the data in Fig. 7 is that the potential for collaboration between species exists for those expressing a greater variety of GH enzymes, in the region in which green pixels are dominant. A possible exception is the profile of *B. longum*, which tended to score neutrally against the profiles of other species. A potential for syntrophy between *Clostridium butyricum* (p8152) and *Brevundimonas diminuta* (p7991) is highlighted, where six enzymes from Table 1 are present in one and absent in the other. The potential for competition between *Bacillus cereus* (p8059) and *Acinetobacter johnsonii* (p8127), which share similar overall profiles, is also indicated.

With a score of 4.50, profiles p8072 and p7991 are likewise *complementary,* which suggests that gut species associated with these profiles, such as *Akkermansia muciniphila* and *B. diminuta* might collaborate within the host gut, as might *C. butyricum* and *B. diminuta* (p8072 and p8152), with *x* = 4.0*. A. muciniphila* and *C. butyricum* (p8072 and p8152) having a pairwise *x* value of -1.5 and are thus classed as competitive according to Fig. 7. The strength of the association, or the disassociation, based on enzyme complementarities, will be related to the potential energy extracted from a given substrate set.

### Feeding mucin-type *O*-glycans to a simulated microbiome

**Figure 8:**
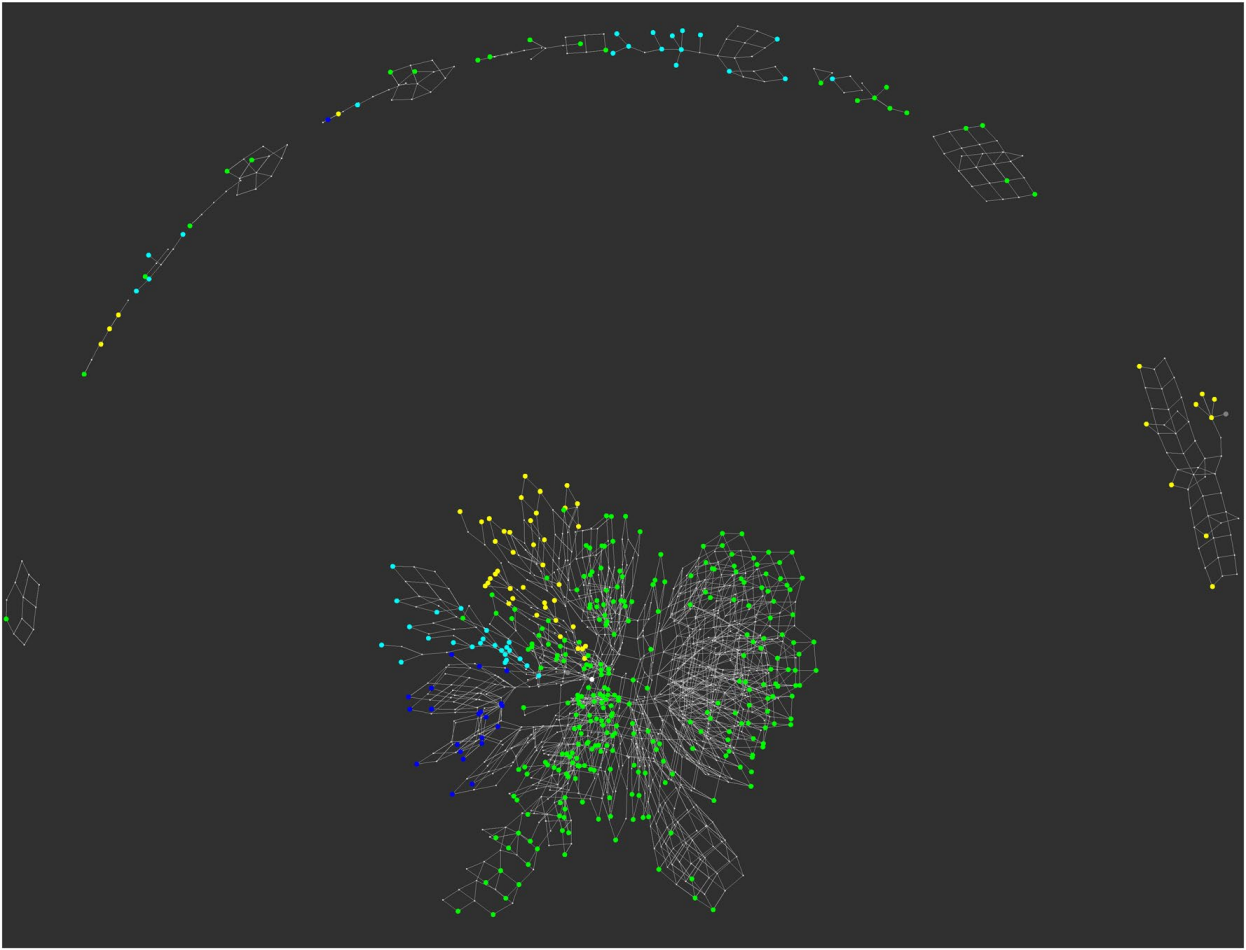
*O*-glycan degradation networks by glycoside hydrolases. 434 mucin-type GalNAc-linked glycans were submitted to the Glycologue glycoside hydrolase simulator for digestion by the enzymes of Table 1. Larger nodes are experimentally observed *O*-glycans, coloured according to the core type (cf. Figure 4): core 1 (yellow); core 2 (green); core 3 (cyan); core 4 (blue); core 5 (orange); smaller grey nodes are inferred intermediates.

For comparison with the HMO population, a set of 434 mucin-type *O*-glycans, characterised from various species, were used as input to the simulator. When all enzymes were available, the digestive network shown in Fig. 8 was obtained, which shows nearly complete digestion of all substrates. Despite the incompleteness of the simulated digestions, the potential energy scores were calculated for the non-gut and gut classes of bacteria, as shown in Fig. 6. With the large numbers of bacteria appearing in the former category, to maintain the same aspect ratios in the heatmaps, the non-gut data were clustered at the Class taxa level, and by Order in the case of gut species. Again, the Bifidobacteriales spp. scored highly, as did *C. perfringens*, although not as highly as when fed the HMO substrate population, perhaps because of a lack of LNB substrates for the Bifidobacteriales. In the upper quartile of these PE scores (PE > 0.6128), with values between 0.7 and 0.8, were profiles p8072, p7944, p7952 and p7936, which were associated, in the data extracted from UniProt, with *A. muciniphila* (and 3 other spp.), *Parabacteroides faecis*, *Bacteroides thetaiotaomicron* (and 3 other spp.) and *Bacteroides caccae*.

### Verification with the CAZy database

The problem of EC number misassignment, either manually or through software, has been noted previously^46–48^. Owing to the possibility of the existence of false positives, or mis-annotations, within the UniProt-derived data, the methods described here were applied to the corresponding data obtained from CAZy, as a way to verify the results independently. In a parallel set of experiments, gut and non-gut bacterial species were ranked according to the number of simulated glycoside hydrolases each possessed, and an enzyme profile value was assigned. With the 3,009,944 Bacterial entries in CAZy, 6,814 unique species were found, of which 5,690 are assigned GH families. Compared with the UniProt/BRENDA, the number of EC number assignments in CAZy was much smaller, resulting in 134 selected species for the simulations, of which 44 were assigned to the gut microbiota and 94 to non-gut. The median number of simulated enzymes available was also lower than in the UniProt/BRENDA-derived dataset, at 1 enzyme per species, for both the gut and non-gut associated subsets.

**Figure 9:**
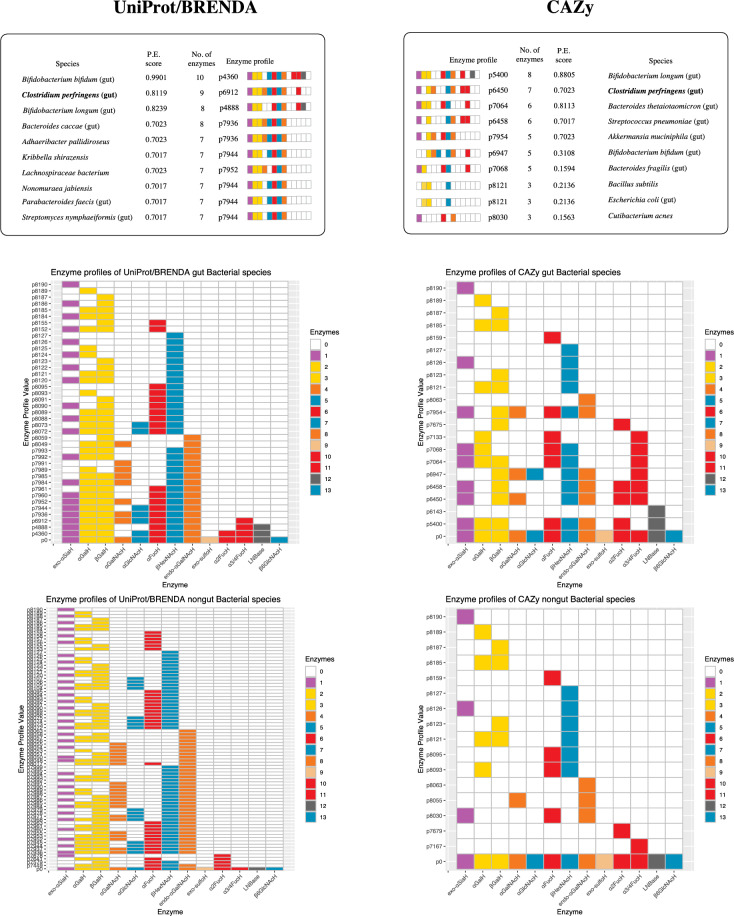
Comparison of UniProt- and CAZy-derived enzyme profiles of bacteria and simulated potential energy scores based on HMOs. A list of the top ten species in decreasing order of the number of simulated enzymes shown in Table 1, along with an enzyme profile value and a vector of cells (**1** to **13**, left to right) filled according to a colour representing the substituent removed, or bond cleaved. Colour key: red (l-fucose); yellow (d-galactose); orange (GalNAc); blue (GlcNAc/HexNAc) magenta (sialic acid); grey (lacto-*N*-biose); white denotes a complete absence of activity. The enzyme profile value is calculated from the source database for each organism. The relative potential energy (P.E.) score matching the enzyme profile of a species fed on a mixture of HMOs. The unique GH enzyme profile values obtained from UniProt/BRENDA (left panels) and CAZy (right panels) are shown beneath, divided into gut and non-gut categories. Each heatmap possesses, as a reference point p0, which is the default value with all enzymes available.

Simulations were repeated with the CAZy dataset, including HMO (Extended Data Fig. E1) and *O*-glycan digestion networks, the calculation of relative potential-energy scores (Extended Data Fig. E2) and pairwise enzyme-profile comparisons (Extended Data Fig. E3). Despite the differences in annotation, a similar pattern emerged: gut species consistently ranked higher both in the number of enzymes and the resulting PE score, as summarised in Fig. 9, when compared to non-gut spp. The enzyme profile values for UniProt- and CAZy-sourced microbiota are distinct, as shown by the profile of *B. bifidum*, which in the CAZy data does not possess the enzyme sialidase, EC 3.2.1.18 (enzyme **1** in Table 1), whereas 10 protein entries within UniProtKB were associated with this activity. The distinction between gut and non-gut bacteria is clearly seen in the heatmaps of Extended Data Fig. E2. Despite the discrepancies in annotation, *Clostridium perfringens* ranks second highest in the number of glycoside hydrolase enzymes active towards HMOs, in both datasets, and, in CAZy, the third-highest PE scorer, jointly with *A. muciniphila*.

## Discussion

Glycologue was originally developed as a simulator of biosynthetic pathways, based on a formal-language approach to describe the acceptor substrates of the glycosyltransferases and sulfotransferases^17,33,49,50^, and the present account is the first application of the method to the glycoside hydrolases. In the model reported here, therefore, we have deliberately focused on enzymatic as opposed to genomic/transcriptomic information associated with bacterial species, since our goal was to compare the potential rather than the observed ability of microbes to process human milk oligosaccharides.

Our analysis of the GH enzymes and their association with gut and non-gut bacteria supports the longstanding findings that Bifidobacteroides species are uniquely suited towards colonising human gut in early life, when fed a diet of complex lactose-based oligosaccharides such as HMOs. Our findings also confirm the report on *A. muciniphila* as being able to thrive in vitro when grown on human milk^28^. *A. muciniphila*-like species are observed to colonise human gut across all age groups, beginning in early life and increasing to adult-like levels within a year^51^. A mucin-degrading strain, Muc^T^, which at lower abundances has been shown to correlate with diseases in humans and mice, can also use HMOs as an energy source (see^52^).

A surprising finding was the case of *C. perfringens*, which scored highly when fed on these substrates, because of its broad glycoside hydrolase apparatus, viz., the enzymes **1**–**8** and **11** of Table 1. One report found no growth of this species on 2′-FL^53^, while another demonstrated growth of the *Clostridium perfringens* ATCC 13124 variant on porcine gastric mucin^54^. Therefore, the induction of a general α-l-fucosidase activity (EC 3.2.1.51) might be possible in an environment where complex fucosylated glycans are in abundance relative to simpler trisaccharides such as 2′-FL. This would support the hypothesis that early colonisation of human gut by HMO-specialising microbes protects against species with a preference for mucin *O*-glycans. Low relative PE scores were obtained for both non-gut species *Staphylococcus aureus* (0.2087) and *Staphylococcus epidermidis* (0.1733), despite a report that both species had shown potential for growth on HMOs^55^. Given that HMO concentrations were not found to decrease during these experiments, however, a mechanism for the promotion of these bacteria in milk, other than catabolism, is likely involved, a conclusion that would be supported by our data.

### Can Clostridium perfringens digest HMOs?

Necrotising enterocolitis (NEC) is an often fatal disease of early infancy, especially in preterm infants^56^. The causes and symptoms of NEC are multifarious, but bacterial agents are implicated in many cases, including members of the Clostridia, Enterococcus and Cronobacter genii^30^. HMOs are known to reduce the likelihood of NEC in many instances^56–59^ and have therefore been the focus of studies into their use as prebiotics^60,61^. Incidence of NEC has been associated with low HMO diversity in low birth-weight infants^62^. Dietary HMOs 2′-FL (2′-fucolactose), 6′-SL (6′-sialyllactose) and DS-LNT (disialyl lacto-*N*-tetraose)^63^ have shown, in animal models, to protect neonatal gut from NEC infection and are thus targets of therapeutic development in humans^64^. The mechanism of protection is likely immunomodulatory, promoting immune cell development and anti-inflammatory pathways^56,65^.

*Clostridium perfringens* is a Gram-positive spore-forming anaerobe found in both soil and human and animal gut, which is implicated in NEC^56^, gastroenteritis^66^ and histotoxic infections. As a food-borne pathogen, its prevalence in meat and dairy industries has been an ongoing concern^67–69^. *C. perfringens* types can produce a wide variety of toxins^70,71^, including alpha (CPA), beta (CPB), epsilon (ETX), iota (ITX) and enterotoxin (CPE). Perfringolysin O (PFO) is a pore-forming toxin that contributes to Clostridial myonecrosis (gas gangrene)^72^. A recent genomics study of *C. perfringens* having linked those with pfoA^+^ strains with *C. perfringens*-associated NEC in neonates^73^, the possibility that *C. perfringens* has a substantial number of the glycoside hydrolase enzymes necessary for HMO digestion may indicate that it has the potential for opportunistic colonisation of the infant gut and thereby contribute to clinical disease. A study of the coincidence of PFO toxins with GH enzyme activities may therefore be warranted. A resolution of the apparent paradox, which is that DS-LNT protects against NEC, while *C. perfringens* is predicted to be able to digest it, may be simply the competition that exists between Clostridial and Bifidobacter species for gut colonisation in early life. A more subtle effect, based on possible inhibition of the sialidases involved by the disialylated DS-LNT, might be elucidated, but confirmation would require a more detailed knowledge of the kinetics of exo-α-sialidases of these species, which is outside the scope of the present study.

The question also arises as to whether certain bacterial competitors in the gut might lower the average concentration of the more complex fucosylated HMOs, which would otherwise be capable of inducing the GH enzymes in *C. perfringens*, especially the sialidases^74,75^ and “generalist” α-l-fucosidase activity (EC 3.2.1.51, *cf*. Table 1)^54,76,77^. It is possible, therefore, that probiotic use would promote competition with *C. perfringens* and similar pathogenic organisms and species beneficial to the host.

### Gut versus non-gut species

The observed contrast between gut and non-gut bacteria may be enhanced by the noisy nature of the non-gut set which contains bacteria of mixed origins (terrestrial, marine, etc.). Nonetheless, differences are evident from a comparison of the number of GH enzymes available and the potential-energy scores obtained with these organisms, and in the heatmaps of Fig. 6 and Extended Data Fig. E2. It is most evident in the latter, where the non-gut species scored poorly. The higher scoring species in the heatmaps, fed on both HMO and *O*-glycan substrates, correlates with the numbers of simulated GH enzymes found in each database, which show a cluster of outliers in the gut category (Fig. 6C). These outliers include the known Lactobacillus spp. and others summarised in Fig. 9. More remarkable, however, is that all others are possible pathogens: *B. caccae*^78^, *B. fragilis*^79^, *C. perfringens* and *Streptococcus pneumoniae*^35^. This could explain the ability for pathogens to interfere and trigger inflammation or infection. Our results suggest that the glycoside hydrolases of *C. perfringens* be examined in greater detail. For example, further characterisation of the Calx-beta domain protein CPF_2130 (UniProtKB Entry A0A0H2YQI3) might be needed, to confirm that this has the fucosidase activity of EC 3.2.1.51 that is ascribed to it (see above).

### Limitations and future directions

Since our objective was to investigate possible GH activity on HMOs and mucin-type *O*-glycans, enzyme activities related fructofuranose (FOS) and galactooligosaccharide (GOS) hydrolysis were excluded from the simulator. In a future release, the enzymes β-fructofuranosidase (EC 3.2.1.80), β-fructosidase (EC 3.2.1.26) and endo-galactanase (EC 3.2.1.89), could be added, to simulate the activities of these bacterial enzymes on fructans and oligosaccharides from non-human milks. The present study has a number of limitations, including the possibility of noise in the data, referred to earlier, but also curation errors, such as misassignment of EC numbers in data repositories. The lack of EC numbers for the sulfatase (**9**) has already been noted, and entries in general have few Bacteria-associated examples in UniProt/BRENDA (Fig. 1A). A β6-linked GlcNAc hydrolase (**13**) exists but was inactive in our simulations owing to the lack of an EC number. It would be interesting to see how this latter enzyme influences the intermediates of the biodegradative HMO pathways, compared to those of biosynthesis. While these enzymes have been modelled in Glycologue, it will not be possible to integrate the simulator with the database-generated enzyme profiles until the enzymes have been officially classified within the EC system. Another possible limitation is that our results reflect a degree of bias in the underlying data, in the sense that certain organisms may have received more interest owing to their roles either in health or disease.

The glycoside hydrolase reactions modelled in this study convey less information than those of the corresponding glycosyltransferase simulators, which model enzymes possessing greater specificities. Accordingly, where the glycosyltransferase rules are readily reversible^49^, the corresponding reversed reactions of hydrolysis would result in many transglycosylated products, leading to increased uncertainty in the choice of the reversed degradation pathway, i.e., in the biosynthetic direction. In order to model, for example, the effect of sulfation on the exo-GH enzymes, additional information about known parent monosaccharide and modifier specificities would need to be added to the reaction rules.

Sugar modifiers such as sulfation or acetylation may block some GH activities, for example the 3S-Gal sulfatase of the bacterium *B. thetaiotaomicron* are inactive towards sulfated mucin *O*-glycans^32^. In the future, as sulfatases from different organisms are characterised, the generic sulfatase activity of enzyme **9** in the model that currently acts as a placeholder will be modified, as needed, and further activities added, such as 3S-GalNAc sulfatase^32^. A further complication to modelling these bacterial enzymes will be the presence of sulfoglycosidases in certain species, for example the 6-sulfo-β-d-*N*-acetylglucosaminidase of *Bifidobacterium bifidum*^80,81^, which has a preference for particular sulfation patterns of GlcNAc.

The potential-energy scores could be adapted to offset the current penalty applied to LNBase action, if an organism is found to possess a transport mechanism for uptake of LNB, as with the GNB/LNB transport system of *B. longum* subsp. *infantis*. In the future, bit masking could be used to incorporate transporter functionality, or to distinguish between intracellularly and extracellularly expressed glycosidase activities, by extracting subcellular location data from UniProtKB, and inverting the bits in the enzyme profile value of those enzymes that are only expressed internally by a given organism. Furthermore, as our results have shown, the pathway simulation for biosynthesis and degradation does not yield the same intermediary products. A more detailed study of these differences could reveal a bias in HMO composition depending on the presence/absence of specific bacterial species or strains. An extension to the simulator would be required to monitor this phenomenon. It is expected that the predictions of Fig. 7 would be the most affected by masking of activities through nutrient uptake and activation of intracellular digestion pathways. Since the scoring system in this work does not distinguish between intracellular or extracellular enzyme activities, the results are a preliminary observation only.

The methods described in this work can be adapted to other components of the human gut microbiome, such as archaea^82,83^, about which relatively little is known compared to bacteria, and to enzymes in additional EC subclasses. As more data on the glycoside hydrolase enzymes of the gut microbiome become available and are curated in UniProtKB, these could be included in the future, in a re-examination of the digestive abilities of these organisms.

## Methods

### Data extraction

Data were extracted from UniProt (https://uniprot.org) (Release 2023_02) using the RESTful Application Programming Interface (API)^84^. For bacterial proteins matching EC numbers in sub-subclass EC 3.2.1 (glycosidases) and EC 3.1.6 (sulfuric-ester hydrolases) the UniProtKB fields Entry, Entry Name, Reviewed, EC number, BRENDA, Protein names, Gene Names, CAZy, Organism, Length, Subcellular location [CC] and Taxonomic lineage were extracted using the stream method as tab-separated value (TSV) files for further offline processing. Queries matched EC numbers appearing in UniProt or BRENDA (Release 2023.1, February 1, 2023), using a Boolean OR, such as query = ((ec:3.2.1.*) OR (xref:brenda-3.2.1.*)). The method was sufficiently general that it could be adapted to other taxonomic domains, such as Archaea, and to other EC sub-subclasses.

Example: https://rest.uniprot.org/uniprotkb/stream?compressed=false&fields=accession,id,reviewed,ec,xref_brenda,protein_name,gene_names,xref_cazy,organism_name,length,lineage&format=tsv&query=((ec:3.2.1.*) OR (xref:brenda-3.2.1.*)) AND (taxonomy_id:2).

The CAZy database was queried for all EC 3.2.1.- entries appearing in the ExplorEnz database (Release 2023.01, January 2023) of IUBMB Enzyme Nomenclature^85^. Downloaded HTML files were parsed and saved as TSV files. Bacterial species names were cross-referenced with the taxonomic lineage data obtained from UniProtKB.

### Glycoside hydrolase simulator

Glycoside hydrolase (GH) enzymes were selected from the ExplorEnz based on their activities towards glycoconjugates and milk oligosaccharides, and correlated with their corresponding Rhea^86^ identifiers (Table 1). A simulator of the GH enzyme reactions was constructed, using the Glycologue formalism described previously^33,49^.

### Enzymes simulated

Thirteen activities were modelled, as shown in Table 1, with the index value **1**–**13**, the EC number, a short name, a longer accepted name, CAZy families and Rhea accession numbers. An alternative version of this table with the Glycologue reaction patterns used in the hydrolase model is given in Supplementary Information (Tables). Reactions within the simulator could be limited by Boolean conditionals, using regular expressions.

### Enzyme profile values

The Glycologue simulator represents enzyme profiles in terms of knockouts of individual activities, the combination of which is an integer ranging from 0 to 2^*n*^-1, where *n* is the total number of enzymes modelled, with bit value 0 representing an expressed enzyme activity, and 1 representing a complete knockout of that activity. The least significant bit of the profile value representing α-sialidase (**1**) in Table 1, and the most significant bit is that of β6GlcNAcH (**13**). For example, the bacterium *Fusicatenibacter saccharivorans*, which was found to possess only the enzyme activities **2**, **3** and **7**, has the profile value p8121 (8121 = 1 + 0 + 0 + 8 + 16 + 32 + 0 + 128 + 256 + 512 + 1024 + 2048 + 4096).

### Matching EC numbers with species

The data extracted from UniProtKB were filtered into separate files according to records matching complete EC numbers in the Glycologue simulator, resulting in 11 datasets, and similarly for CAZy. For each set of records matching a single EC number, a set of organism names was extracted. The number of unique activities within each sub-subclass and taxonomic domain was recorded within the UniProtKB and CAZy databases. To visualise the overlap between repositories, Venn diagrams were produced in R using package venn (venn_1.11).

Species were separated into classes, those known to be associated with human gut, and all others, which were described as non-gut. Of the gut species, a subset were identified as potentially pathogenic based on published reports^35^. From the UniProtKB lineage data, species were ranked according to their position with the superkingdom, phylum, class, order, family, genus and species, with the species being stored as the assigned binomial name. For every binomial species in each subcategory, a set EC numbers associated with GH activities in UniProt/BRENDA was deduced and an enzyme profile value assigned to that species. Krona^36^ graphs were generated from profile value and taxonomic lineage data.

### Glycoside hydrolase simulated digestions

Two sets of substrates, 226 human milk oligosaccharides based on lactose^17^, and 434 GalNAc-linked *O*-glycans, encoded in the Glycologue modelling language^33,49^, were obtained from published studies to use as input to the glycoside hydrolase simulator. For each species, each substrate in the set was passed to the enzymes capable of being expressed by the organism, as a supplied profile value, and degraded according to the exo- and endo-glycosidase reaction patterns. The process proceeded iteratively until no further degradation products were formed. The number of distinct simulated reactions was counted, along with the number of enzymes activities used during the degradation of the complete set of substrates. Each substrate-product reaction pair was recorded together with the enzyme catalyst. Reaction networks were drawn using Tulip^87^ software (https://tulip.labri.fr), using the Stress Majorization (OGDF) graph layout algorithm.

### Calculation of potential energy scores

Every successful hydrolase reaction releasing a monosaccharide was assigned a score of 1, and the release of a disaccharide such as Gal-(β1,3)-GlcNAc from lacto-*N*-biose was counted as 0.5, to simulate the effect of a delayed entrance to energy metabolism. A theoretical maximum energy score was calculated from the enzyme profile p0, where all enzymes were available to act on the set of all substrates. The organism-related energy score was then calculated as the fraction of simulated energy score for an organism divided by this theoretical maximum. Heatmaps were generated in R, coloured by calculated energy score, relating profile value to species grouped at the level of phylum, class, order, or genus.

### Calculation of potential syntrophies

Enzyme profile values were compared pairwise using a bitwise-XOR comparison, to obtain a profile of shared and unshared enzymes. The number of enzymes matching between profile values at the same bit position were scored positively if the value of the XOR. Since the Glycologue profile value encodes knockouts of activities, its bitwise inverse was calculated by a conjunctive AND operation with 2^*n*^-1, where *n* = 13. For each profile-value pair, (*p*_1_, *p*_2_), the result of (*p*_1_ AND *p*_2_ AND 2^*n*^-1) was recorded and scored according to the enzymes active in each profile. Where an enzyme was expressed in one organism but not the other, a collaboration might exist (syntrophy), while enzymes common to both were treated as competitive, and scored negatively. Enzymes lacking to both members of a pair were scored neutrally: *p*_1_ XOR *p*_2_ = 1, ∆*x* = 1; *p*_1_ AND *p*_2_ = 0, ∆*x* = 0; *p*_1_ AND *p*_2_ = 1, ∆*x* = –0.5. The final value of *x* was obtained as the sum of all the pairwise bit comparisons.

### Web application

The glycoside hydrolase simulator is embedded within each member of the family of Glycologue glycosylation-related enzyme simulators (https://glycologue.org), where it can be used to predict digestion networks of the products of glycosyltransferase enzymes.

### Supplementary Information

Supplementary Figures.

Supplementary Information.

## Data Availability

The HMO and *O*-glycan structures used as substrates of the hydrolase simulator are available as CSV files (Supplementary Information). The enzyme model simulator is available at https://glycologue.org/m/. GlyConnect Compozitor is available at https://glyconnect.expasy.org/compozitor/.
