# Supplementary material for: Simulated digestions of free oligosaccharides and mucin-type O-glycans reveal a potential role for Clostridium perfringens
Source: Sci Rep. 2024 Jan 18;14:1649. doi: 10.1038/s41598-023-51012-4 (PMC10796942; doi:10.1038/s41598-023-51012-4)
Supplement: Supplementary file 1 — Supplementary Figures. [file 41598_2023_51012_MOESM1_ESM.docx]

# Extended Data

**Figure E1.** Simulated networks of HMO degradation by glycoside hydrolases expressed in Bacteria, when each of 226 unique human milk oligosaccharides, were submitted to the simulator. Data are sourced from CAZy (<http://cazy.org>). A. Reference network obtained with all enzymes of the model available. B. Network corresponding to the enzyme profile p5400, representing *Bifidobacterium* *longum*. C. Network p6450 (*Clostridium perfringens*). D. Network p6143, corresponding to *B. sanguini*, which in the CAZy data expresses only LNBase (Table 1). Nodes are coloured according to type of core structure based on the reducing end of the HMO [17]: red (lacto-*N*-tetraose), cyan (lacto-*N*-neotetraose), blue (lacto-*N*-hexaose), orange (lacto-*N*-neohexaose), grey (other).

**Figure E2.** Predicted potential energy scores assigned to Bacteria fed a population of human milk oligosaccharides and mucin-type *O*-glycans. For each unique enzyme profile, with a subset of the simulated enzymes, a simulated glycoside hydrolase degradation network was generated, and the number of monosaccharides released was counted (the action of lacto-*N*-biosidase was counted as 0.5 instead of 1). Each profile was then scored based on the ratio of this value to the maximum possible value for the HMO dataset, with all enzymes active. Colours are assigned from grey through blue for the PE scores (0 to 1). **A**. Non-gut species, grouped by taxonomic Class. **B**. Gut bacterial species, grouped by taxonomic Order. The position of *C. perfringens* is indicated by an arrow. **C**. Numbers of simulated enzymes available to gut and non-gut species. **D**. Frequency distribution of potential energy scores of HMO-fed bacteria. Highly scoring gut species, fed *in silico* on HMOs (A, right) were *B. bifidum* (p4360), *B.* *longum* (p4888) and *C. perfringens* (p6912); the highest scoring on mucin-type *O*-glycans (B, right) were *B. bifidum* (p4360) and *C. perfringens* (p6912). Data are sourced from CAZy (http://cazy.org).

**Figure E3.** Competition and symbiosis between gut bacteria, based on available enzyme profiles. For each bit in profiles *p*_1_ and *p*_2_, a syntrophic score increased by 1 when the bitwise comparison *p*_1_ XOR *p*_1_ = 1 (Boolean true), decreased by 0.5 when *p*_1_ AND *p*_2_ = 1 (Boolean true), and left unchanged when *p*1 AND *p*_2_ = 0 (Boolean false). A syntrophic pair (green) and competitive pair of species (red) are indicated. Data are sourced from CAZy (<http://cazy.org>). See text for further details.
