# Supplementary material for: Simulated digestions of free oligosaccharides and mucin-type O-glycans reveal a potential role for Clostridium perfringens
Source: Sci Rep. 2024 Jan 18;14:1649. doi: 10.1038/s41598-023-51012-4 (PMC10796942; doi:10.1038/s41598-023-51012-4)
Supplement: Supplementary file 2 — Supplementary Information. [file 41598_2023_51012_MOESM2_ESM.zip › gutGH-SI/gutGH-SI-description.docx]

A unique role for *Clostridium perfringens* in the digestion of HMOs

Andrew G. McDonald^1,2,*^ and Frédérique Lisacek^1,*^

1. Proteome Informatics Group, SIB Swiss Institute of Bioinformatics, 1211, Geneva, Switzerland
2. School of Biochemistry and Immunology, Trinity College Dublin, Dublin 2, Ireland

*Corresponding author(s): [amcdonld@gmail.com](mailto:amcdonld@gmail.com); [frederique.lisacek@unige.ch](mailto:frederique.lisacek@unige.ch)

# Supplementary Information

## Description

The additional data and figures are provided in five sets, subdivided into two subclasses depending on the data source, UniProt or CAZy.

Data — Data downloaded from UniProt and CAZy, as tab-separated values (TSV) files. Taxonomic lineages are those of UniProt.

Krona — Interactive Krona charts (HTML) that map the available to used enzyme profile values for each organism and the individual EC numbers of the simulator to different species.

Networks — additional simulated digestion networks of gut microbial species, as JPEG images. Files are named according to the available enzyme profile, for example:

p4888-GH-network-pp-og.jpg

represents the network of enzyme profile value 4888, generated by the GH (glycohydrolases) simulator, postprocessed (pp) to highlight the experimentally observed structures in the *O*-glycan substrates dataset (og).

Only the networks of gut bacterial species are included. Networks for the enzyme profiles of non-gut species can be provided on application to the corresponding author.

Scores — the enzyme profile values and energy scores obtained with an organism based on the substrate sets in the corresponding files (folder “substrates”). Results are provided for the profiles of both gut and non-gut organisms fed on HMO (hmo) and *O*-glycan (og) substrates.

Substrates — the HMO and *O*-glycan substrates that were used as input to the glycohydrolase simulator, as CSV files. Structures are encoded in both GlycoCT condensed format and as the native Glycologue structure identifier.

Tables — Table SI-1, an alternative version of Table 1 in the main manuscript, to include the reaction rules Glycologue, with a key to the symbols used given in Table SI-2.
