# Supplementary material for: Simulated digestions of free oligosaccharides and mucin-type O-glycans reveal a potential role for Clostridium perfringens
Source: Sci Rep. 2024 Jan 18;14:1649. doi: 10.1038/s41598-023-51012-4 (PMC10796942; doi:10.1038/s41598-023-51012-4)
Supplement: Supplementary file 2 — Supplementary Information. [file 41598_2023_51012_MOESM2_ESM.zip › gutGH-SI/Tables/Supplementary Tables.docx]

# Supplementary Information (Tables)

**Table SI-1. Enzymes of the model.** Glycohydrolases included in the Glycologue simulator, with Glycologue reaction patterns. For symbols used, see Table SI-2.

| **Enzyme no.** | **EC number** | **Short name** | **Accepted name** | **Rhea Acc. no.** | **Reaction pattern^a^** |
| --- | --- | --- | --- | --- | --- |
| **1** | EC 3.2.1.18 | exo-αSiaH | exo-α-sialidase | [-](https://www.rhea-db.org/rhea/22932) | *[S?* + H2O = [S] + *[* |
| **2** | EC 3.2.1.22 | αGalH | α-galactosidase | 21112 | *[La?* + H2O = [L] + *[* |
| **3** | EC 3.2.1.23 | βGalH | β-galactosidase | - | *[Lb?* + H2O = [L] + *[* |
| **4** | EC 3.2.1.49 | αGalNAcH | α-*N*-acetylgalactosaminidase | 15085 | *[Va3[fa2]L* + H2O = [V] + *[[fa2]L* |
| **5** | EC 3.2.1.50 | αGlcNAcH | α-*N*-acetylglucosaminidase | - | *[Ya4L* + H2O = [Y] + *[L* |
| **6** | EC 3.2.1.51 | αFucH | α-l-fucosidase | 12288 | *[[fa?]* + H2O = [f] + *[* |
| **7** | EC 3.2.1.52 | βHexNAcH | β-*N*-acetylhexosaminidase | - | *[Yb?L* + H2O = [Y] + *[L*  *[Vb?L* + H2O = [V] + *[L* |
| **8** | EC 3.2.1.97 | endo-αGalNAcH | endo-α-*N*-acetylgalactosaminidase | 30983/54540 | [Lb3]VT + H2O = [[Lb3]V] + T  [Yb3]VT + H2O = [[Yb3]V] + T |
| **9** | EC 3.1.6.- | exo-sulfoH | (exo-sulfhydrolase) | - | [[s?]* + H2O = [s] + [* |
| **10** | EC 3.2.1.63 | α2FucH | 1,2-α-l-fucosidase | 10816 | *[[fa2]* + H2O = [f] + *[* |
| **11** | EC 3.2.1.111 | α3/4FucH | 1,3-α-l-fucosidase | - | *[[fa3]* + H2O = [f] + *[*  *[[fa4]* + H2O = [f] + *[* |
| **12** | EC 3.2.1.140 | LNBase | lacto-*N*-biosidase | 21568 | [L3Y3L4G] + H2O = [L3Y3] + [L4G]  *[[L3Y3L4* + H2O = [L3Y3] + *[L* |
| **13** | EC 3.2.1.– | β6GlcNAcH | (6-β-*N*-acetylglucosaminidase) | - | *[Yb6* + H2O = [Y] + *[* |

^a^Asterisks (*) act as a wildcard character, to denote an unspecified portion of the oligosaccharide. A question mark (?) denotes an unspecified linkage position on the parent monosaccharide. Symbols and abbreviations used in reaction patterns are those of Table SYMBOLS. A generic exo-sulfohydrolase (**9**) is listed but was unused.

**Table SI-2 Monosaccharide symbols.** Definition of monosaccharide units of HMOs represented as single-letter codes in Glycologue, symbols in the Symbol Nomenclature For Glycans (SNFG) notation [39], IUPAC symbols. For example, N-acetylgalactosamine (GalNAc) is represented by Glycologue as ‘V’, and sulfate by the lowercase ‘s’.

| **Glycologue symbol** | **SNFG symbol** | **IUPAC short name** | **Definition** | **Assumed  configuration** |
| --- | --- | --- | --- | --- |
| f | 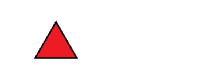 | Fuc | l-fucose | α |
| G | 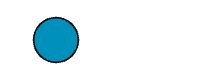 | Glc | d-glucose | β |
| L | 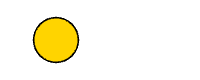 | Gal | d-galactose | β |
| S | 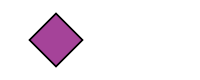 | Neu5Ac | *N*-acetylneuraminate | α |
| V | 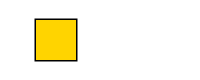 | GalNAc | *N*-acetyl-α-d-galactosamine | α |
| Y | 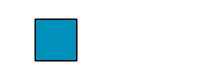 | GlcNAc | *N*-acetyl-α-d-glucosamine | β |
| s | **S** | sulfate | Sulfate | - |
| a, b | n/a | α, β | anomeric configuration | - |
