# Supplementary material for: Simulated digestions of free oligosaccharides and mucin-type O-glycans reveal a potential role for Clostridium perfringens
Source: Sci Rep. 2024 Jan 18;14:1649. doi: 10.1038/s41598-023-51012-4 (PMC10796942; doi:10.1038/s41598-023-51012-4)
Supplement: Supplementary file 2 — Supplementary Information. [file 41598_2023_51012_MOESM2_ESM.zip › gutGH-SI/Krona/UniProt-EC-Krona-graphs/gut-EC.krona.html]

Javascript must be enabled to view this page.

magnitude
magnitudeUnassigned

EC\_3.2.1.111
EC\_3.2.1.140
EC\_3.2.1.18
EC\_3.2.1.22
EC\_3.2.1.23
EC\_3.2.1.49
EC\_3.2.1.50
EC\_3.2.1.51
EC\_3.2.1.52
EC\_3.2.1.63
EC\_3.2.1.97

328823527815845284114

328823527815845284114

222

222

222

222

222

111

111

228576611135314

227576411135114

31

31

31

1

11

1

4717

11

11

11

231

11

11

11

11

11

11

2315

111

111

24

11

1

1

11

11

11

111

111

111

111

227525311124214

227525311124214

227525311124214

111

11

11

11

111

11

11

111

11

11

111

111

1111

1111

11

111

111

11111

111

111

111

111

1111

11

11111

11

111

1111

111

111

111111

111

111

111

111

111

11111

1111

111

11111111

1111

111

11

1111

111

111

1111

11

111

1111111111

111

1111

111

122

122

122

122

1

1

111

122

122

122

122

122

111

11

2648517516543

111

111

111

111

111

2646516516513

2646516516513

2777

1666

111

1

111

1111

111

111

11

1111

1111

66116

66116

11111

111

111

111

111

111

1826265311262

1622225310222

111111

1111

111111

11111

11111

11111

11111

11111

1111

111

111

11111

111111

111

11111111

1111

1111

111

111111

111111

111

111

24414

111

1111

111

11111

1111

1111

1111

46611161

46611161

1111111

11111

1111

111

1111

111

111

111

111

1424

1424

111

111

11

111

12

12

12

12

1

11

2039552396

125119

5

5

1

1

3

1

1

1

1

1

125114

13

12

1

11

1

1

11

11

11

1116

1

1

3

1

1

1

11

11

11

11

1

1

133

122

111

11

11

11

1

1

1

152942264

16

16

16

1

1

11

1

1

1

112431238

388

277

11

111

111

11

11

11

11

111

111

112119225

144

11

11

11

111

2222

1111

1111

111

111

11111

11111

14515

111

111

11111

111

11

7828

111

1111

11

222

1111

111

111

333

111

111

111

111

111

1

1

1

22

11

11

11

11

22

11

11

11

11

1245

1245

1245

11

11

1111

11

11

1222

1222

1222

111

1111

3

3

1

1

1

1

1

1

114

114

1

1

113

1

111

1

1

1

1

1

145

145

144

11

11

111

11

1

1

21

21

21

21

1

11

288212

13

13

2

1

1

11

3423

3423

3423

1111

1111

11

11

1

1

1

1

1111

1111

1235

11

11

11

22

22

11

11

1

1

112

111

111

1

1

22

22

1

1

1

1

12

12

12

1

11

111111

111111

111111

111111

111111

111111

13286995112767

936418313

61091

132

131

11

11

1

1

1

2431

2321

11

111

111

11

11

333

111

111

222

111

111

1

1

930318222

1

1

1

9102

111

111

11

11

661

11

111

11

11

11

11

12

1

11

333

333

111

111

111

11

11

1

1

916178172

1344

111

1111

11

111

813138132

111

11111

11111

111111

1111

1111

11111

111

1

111

1111

1111

111

1

111111

1587

1587

11

11

11

1465

111

111

211

111

1

1

1

111

111

1

1

1

1

111

111

111

111

111

111

11

11

11

11

1

1

1

1

1224449514374

1224449514374

18811113122

122121

111111

111

1769112101

1111

11

11

111

11

111111111

111

111

111

111

1111

15541

15541

111

11

111

11111

111

3455

1

1

11

11

1

1

11

11

111

111

1111

1111

1111

1111

121

1

1

1

1

11

11

10262641151

266261

111

111

1111

11111

11111

111

111

111

12212

111

11111

111

111

22

11

11

111

111

11

11

221

1

11

11

1221

1111

11

1111

1111

24412

1111

11

11

11111

222

111

111

111

111
