# Supplementary material for: Simulated digestions of free oligosaccharides and mucin-type O-glycans reveal a potential role for Clostridium perfringens
Source: Sci Rep. 2024 Jan 18;14:1649. doi: 10.1038/s41598-023-51012-4 (PMC10796942; doi:10.1038/s41598-023-51012-4)
Supplement: Supplementary file 2 — Supplementary Information. [file 41598_2023_51012_MOESM2_ESM.zip › gutGH-SI/Krona/UniProt-EC-Krona-graphs/nongut-EC.krona.html]

Javascript must be enabled to view this page.

magnitude
magnitudeUnassigned

EC\_3.2.1.18
EC\_3.2.1.22
EC\_3.2.1.23
EC\_3.2.1.49
EC\_3.2.1.50
EC\_3.2.1.51
EC\_3.2.1.52
EC\_3.2.1.63
EC\_3.2.1.97

8502973386089522225386147

8502973386089522225386147

8144583752911

11111

693246344171

322111

322111

11111

11111

11

11

111

111

24311124

23210123

111

111

11

11

1111

1111

111

111

1

1

111

111

111

111

222

111

111

11

11

821

11

1

11

1

11

1

1

1

1111

211

11

11

1

1

111

111

1111

21

21

11

11

1

1

11

39273320411

131211514

222

111

111

3232

11

1111

1111

111

111

1111

1111

1111

1111

22221

11111

1111

22111

111

1111

111

111

2513201425

2221

1111

111

212

11

111

11

11

33

11

11

11

355

111

111

111

11

11

121

11

11

111

111

424223

1111

1

1111

111

11111

1

11

11

332

11

111

111

1

1

3223

1111

11

1111

111

111111

111111

111111

11111121111

1111

55515

1111

44414

1111

1111

1111

1111

1111

11111

11111

1111

444241

111111

111111

111111

33313

2222

1111

1111

11111

11111

223

112

112

11

11

11

11

111

1565

111

111

111

111

111

1454

1

1444

1122

1111

11

11

1

222

222

111

111

111

111

55610

3347

3347

111

2336

1111

3

1

1

1

1111

111

111

1

1

1

1

1

1111

1111

11021114

11

11020113

1412111

2513

11

11

11

2311

1

1111

11

1167

112

11

11

155

11

11

111

11

11

111

3

3

1

2

1

1

111

111

111

111

541

541

1

1

11

11

22

11

11

111

111

11

11

11

11

11

11

931128

931128

4125

4125

222

111

111

1

1

232

111

11

11

7

1

1

1

1

1

1

1

518122

518122

417121

11

11

1

11

1

11

11

1

111

1

1

11

2

11

111

111

1111

11

11

1

11

11

111

111

11

11

11

1

1

22

11

11

11

11

11

46537

1213

111

112

1

1

111

12212

11111

11111

11111

11111

111

111

111

111

11111

11111

127951104812180106826

342

342

342

111

12

1

11

111

31

21

11

1

1

1

1

1

1

1

1

12

1

1

1

1

2

2

2

1

1

121935103112179105626

1111

22

22

22

11

11

453

453

453

11

11

111

111

11

1201917

1201917

11

11

331

111

11

11

111

111

151215

11

111

1

11

1

11

11

111

11

1111

11

11

1

1

111

11

11

11

1

1

262242538172736

262242538172736

233

111

111

11

22

11

11

8819

1111

111

11

11

111

111

111

111

111

11111

11111

111

252122388152576

111

111

1111111

1111

1

111

111

111

11

111

111111

11

11

111

11

111

111

11

111

111

1

111

111

111

111

111

11

111

111

1

111

111

111

111

11

111

111

1

11111

11

111

111

111

111

11

111

222

1111

111

1

11

111

111

111

11

11

111

111

111

1111

111

1111

11

111

111

111

111

111

1111

111

111

111

111

111

1

111

111

111

111

111111

111

111

11

111

111

111

111

111

111

11

111

111

111

111

111

111

11111

111

111

111111

11

111

111

111

111

1111

111

111

11

11

111

11

11

11111

11

11

1

111

111

111

111

111

111

111

11

111

111

111

111

111

111

11

111

111

111

111

111

1

111

11

111

111

111

1111

111

111

111

111

111

111

1111

11

111

111

111

111

111

111

111

1111

111

111

111

111

111

111

111

11

111

11

111

11

111

11

11

11

111

111

111

111

11

11

111

1

111

111

1

111

111

11

111

1

111

111

11111

111

111

11111

1111

111

111

1

111

111

111

111

11

11111

111

111

11111

1111

11111

1

111

111

111

11

111

111

111

111

111

111

11

111

111

111

111

11

1111111

111

111

111

111

111

111

111

111

111

111

111

111111

111

111

111

11111

1

111

111

111

111

11

111

111

111

111

111

111

254

254

1

122

11

11

1

32

1

1

1

11

5113110281212

5113110281212

31515215

111

11111

111

1111

111

111

111

111

1111

111

111

1111

111

111

111

111

111

444

111

111

111

111

333

111

111

111

111

111

111111

111111

11

11

111

111

11

11

111111

111111

1

1

1

1

444

111

111

111

111

26664371

111

111

11

111

111

111

111

111

1

111

111

111

111

111

111

111

111

111

1

111

111

1

1111

111

111

111

111

111

111

111

111

111

111

111

1

111

111

11

111

111

111

111

1111

111

111

111

111

11

111

1

1

111

111

111

111

111

111

111

11111

111

111

111

1

111

111

111

111

111

111

111

111

111

111

111

111

111

111

333

111

111

111

12

1

11

111

111

111

1111

1111

11

11

555

111

111

111

111

111

11

11

11

71312113

71312113

3444

1111

1111

1111

111

48718

111

1111

1111

111

111

11111

111

111

111

111

7910

7910

22

11

11

777

111

111

111

111

111

111

111

1

1

62541293

111

62339273

1123

1

1111

11

112156

11

11

111

11

111

11

11

1111

11

111

11

11

11

11

1

111

111

1

11541

1

111

1111

11

11

111

111

11

11

1

1

111

111

133

11

111

11

11

11

11

11

111

111

1

1

11

11

11

11

111

111

21232

11111

11

1

11

111

10831025141154

114212227

1

1

111

111

11

11

112192122

11

1

1

1

11

111

11

11

11

11

111

11

1

111

1

11

11

11

1

11

1111

111

111

1

1

11111

1

11

112

1

111

65463111664

244

11

111

111

11

1

1

222

111

111

222

111

111

111

111

777

111

111

111

111

111

111

111

11111

11111

22

11

11

5262617264

111

111

111

111

11111

111

111111

111

111

111

111

1111111

111

111

111111

111

11111

111

1111

111

111

111

111

111

11111

111

5727

1111

11

1111

111

11

111

111

2314

11

1111

11

11

555

111

111

111

111

111

133

111

11

11

1

1

11

11

11

111

111

312152120

1111

1111

111

111

111

111

11

11

111

111

1671110

1

111

111

1

1111

1

111

111

111

1111

1324

1111

11

1

111

1

1

1

1

111

312

22

22

11

1

1

11

222

1

1

1

221

221

11

111

24227263182075

220234241

11718419

11

111

1111

1111

111

111

111

111

111

111

111

11

111

111

111

11

111

1111

11111

111

111

11221

11

11111

111

111

11

11

11

11

434

111

111

323

11

1

11

1

11

1211613091002

1215212

11

11

11

111

1

11

111

1

111

11

111

111

1111

1111

11

1

111

2211

11

1111

1

1

1111

1111

155251

11111

111

1111

1111

111

22

11

11

11

11

111

111

111

111

781

11

11

111

11

11

1

11

11

457

111

111

1

1

111

11

111

111

1

1

1

1

11

11

333

111

111

111

222

111

111

111

111

12

1

11

11

11

111

111

111

111

11

11

23

11

11

1

1111

1111

123

111

1

11

442

11

11

111

111

11

11

111

111

111

1

1

1

11

11

111

111

1

1

735412411

111

11

11

111

111

111

1111

11

111

1111

111

11

111

11

1111

1111

1111

1111

11

111

1111

111

1

111

111

111

11111

1

111

11

1

11

111

1

11

11

111

111

1

111

111

111

111

111

111

111

11

11

1

1

111

111

2

1

1

11

11

22

11

11

222

111

111

16614

111

111

11

11111

111

11

1211

11

111

221

111

11

11

11

12

12

11

1

421

21

1

11

211

111

1

291414

171111

111

111

11

1111

111

11

111

11

11

111

111

1222

1111

111

11

11

12211

12211

11

11111

16214

11

11

11

11

11

11

11

11

211

11

1

1

11

11

1

1

1

333

333

111

111

111

711113

11

11

122

111

11

133

111

11

11

23

11

11

1

111

111

2212

1111

111

111

111

111

111

111

1271

1111

1111

16

1

1

1

11

1

1

1333

222

111

111

1111

1111

333451201

45

1

11

11

11

11

1

1

23

1

11

11

121

11

11

14

11

1

1

1

1

1

44

11

11

11

11

124

1

11

111

1

12

11

1

1

1

318211111

111

11

1

11

11

1

1

111

111

111

111

111111

11

111

11

1111

11

11

111

11

111

1

1

11

11

2

1

1

11125

111

111

111

111

341

1

11

111

11

1

1

1

111

111

11

11

11

11

12

11

1

11

11

442

221

11

111

111

111

11

11

12212

111

11111

6724

6724

1

1

221

11

111

4423

11

1111

111

1111

188

188

188

11

11

11

11

111

11

11

11

1111

1111

1111

1111

207187271141

11

197087271141

1

1

726281236

11111

11

111

111

111

1

11

111

11

1

111

111

1

1111

111

111

111

111

111

111

111

11

111

11111

11111

111

1

111

111

111

111

11

111

1

111

111

11

22212

11111

1111

111

111

111

111

1491

11

1

11

1

11

111

1

11

1

111

111

111111

111

111

111

111

111

111

111

111

111

111

111

111

111

12212

11111

111

1

1

111

111

11

11

144114

111111

111

111

111

111

111

111

111

16518

1

111

11111

111

1

111

11

111

111

111

111111

111111

222

111

111

36

1

1

1

11

1

1

11

1

1

231015

11

1

1

111

11

11

1

11

11

111

1111

11

11

1

1

1

114

111

1

1

1

117

1

1

1

11

1

1

11

2

1

1

953181173

1710127

765

11

11

11

1

1

1

111

1

1

111

1

1

1

1

1

94121

1

1

11

1

1

1

1

1

1

11

1

111

11

1

1

11

1

1

1

11

1

111

111

1

111

111

111

111

73

73

11

1

1

1

1

1

1

1

1

2

2

1

1

914123

914123

1

111

1

1

1

1

1

1

1

11

1

1

1

111

111

1

1

1

1

11

1

11

11

1

1

1

1

1

23117

1

1

1

1

1

3

1

1

1

410

1

1

1

1

1

1

1

1

1

1

1

1

1

1

1614

1

1

1

1

11

1

1

1

1

1

1

11

1

1

11

1

1

1

111

117684410695

125232201

111

111

111

23

11

1

11

118152161

11

11

1

11

1

11

1

1

11

11

1

1

11

1

11

11

11

1

1

1111

1

11

11

1

1

11

1

1111

11

1

1

11

11

11

111

111

111

531411528

3313

1111

111

111

27737

11111

111

11111

111

111

1111

111

24

1

1

11

11

11

1

1

222

111

111

112111

1

111111

111

111

576

1

11

111

11

11

111

111

11

11

12

1

11

332

111

11

111

1133

1111

11

11

111

111

1111

1111

111

111

1

1

11

4171714174

111

4161614164

111

1111111

111

111

111

111

111

111

111

111111

111

111

111111

111111

111

111

12313

12313

111

11

11111

3876

1111

2765

221

221

111

11

1111

1111

222

111

111

111

1

1

1

23313

1

111

111

111

12112

11

11

11

11

1

1

1

11

11

11

1

927341336

111

111

111

111

111

41213214

111

41112213

22

22

22

222

111

111

111

47729

111

111

1111

1111

11

11

111

111

111

11111

11111

22112

1111

1

111

1

1

1

1

1

11

1222

1111

1111

111

3111616

1476

1476

111

111

111

111

111

11

11

11

11

11

11

111

111

2789

2688

1111

1111

111

111

111

111

22

11

11

111

111

111

1111

1111

11

11

111111

12

1

11

14414

11111

111

111

111

111

222

111

111

111

1484856093014418532

7415623017211370

111

7415522917211369

238518176

111

111

1

1

14414

111

111

111

11111

21

11

1

55

1

11

1

11

11

11

11

11

1

1

126406168

1

111

111

1

1

11

11

11

111

11

1

11

11

111

11

11

11

111

111

11

1

11

111

1

1

1

111

11

1

111

11

1

111

1

111

1111

11

11

11

1

111

1

1

11

1

11

111

111

111

111

1

111

11

1

11

11

111

11

111

111

1

1

11

111

1

111

11

11

11

11

11

691151759210285

1

1

2236

1

111

11

11

11

111

122

11

111

211

11

11

12

1

1

1

111

111

1

1

355

111

11

111

1

111

1

227

1

1

11

1

1

1

1

1

11

11

11

11

11

11

11

11

11

2

1

1

112

1

111

1

1

11

11

2245

11

11

11

111

1111

14126

11

1

111

11

11

1

111

12

1

11

1

1

1

1

111

111

1

1

11

11

111

111

13

1

11

1

1

1

11

11

1

1

1111

1111

111

111

111

111

111

111

122

11

111

11

11

22

11

11

2243

11

1111

11

111

3

1

1

1

115121

1

1

11

11

1

1

1

1

1

1

111

1

1

1

1

11

111

11

1

1

1

3445

1

1111

1111

1111

111

11

1

1

1

1

11

1

1

11

11

55102410

111111

1111

11

11

11

111111

11111

11111

11

11

1127

1

1

11

1

111

1

11

11

11

1

1

2513

1

1

111

11

1111

11

11

111111

111111

1

1

1

1

111111

1

1

1

1

12

1

11

1

1

11

11

256

11

11

1

111

111

11

258

111

11

11

1

11

1

11

11

2398

11

11

1

111

11

1111

1

1

1

111

11

1

1

12

1

11

33

11

11

11

1

1

1

1

11

11

33645611284

111

1

111

111

11

111

111

111

1

111

111

111

1

111

111

111

111

1

111

111

111

1

11

11

1

1

1111

1

1111

1

1

111

111

11

1

1

1111

1111

111

1111

111111

1

1

1

111

111

1

1111

1

11

1

1111

1111

1

111

111

1

111

111

1111

111

111

111

111

11

1

1111

1

1

1

1

111

1

111

11

111

1

1

11

111

1

1

1111

1

1

11

111

1111

111

1

1111

1

1

111

1

111

1

1

1

1

1111

1

11

111

1

111

31518

11

11

111

1

11

11

11

11

11

11

111

11

11

1

11

1

111

11

35

1

1

11

1

1

11

333

111

111

111

111

111

1

1

3

1

1

1

11

11

1

1

1

1

1

1

21

1

1

2

1

1

114

1

1

1

1

1

1

111

1

1

1

1111

2589101149175

1

2589101149174

266

111

44

11

11

11

11

111

111

122

122

11

111

15

15

11

1

1

1

1

1

1

1

5393412264

111

111

171733

1

111

111

111

1

11

111

11

111

11

111

11

111

1

111

111

11

1

1

11

1

1

1

111

111

1

1

11

11

11

111

1

11

3621116

11

111

11

111

1

1111111

11

11

11

18816

111

111

1

111

11

1

1

111

1

111

111

1

11

111

1

11

76117

11111

111

111

111

11

111

111

1444

111

111

1333

1111

111

111

111

111

111

11

11

419181227

212

11

111

1111

1111

111

1131014

11

111

111

111

11

111

11

1111

111

111

111

11

111

11

111

111

111

111

11

11

21113

11111

1

11

11

11

1

1

11

11

111

819334

2

1

1

512120

11

1

11

11

1

1

1

1

1

111

1

111

11

1

1

11

1

1

1

1

11

11

1111

1111

1214

1

1

1111

11

1

1

2

1

1

111

22

11

11

721241228

535

11

111

11

111

111

11

11

111

111

581111

111

111

111

1111

11

1111

1111

11

111

1111

111

11

11

111

111

11

11

122112

111

111111

111

111

1

1

12313

1111

111

111

113

113

11

1

1

11

11

6626172

6626172

6626172

111

111

111

111

111

111

12

11

1

222

111

111

444

111

111

111

111

111

111

1111

1111

13

11

2

111

111

2303132

111

111

111

111

111

11

111

111

1111

111

1111

111

111

111

111

111

222

111

111

111

111

111

111

1

111

111

111

111

111

111

111

111

111

11

11

111

111

1111

122

111

11

111

111

333

111

111

111

111

111

111

111

111

111

111

111

222

111

111

1

1

112

111

1

1111

1111

111

111

12

11

1

111111

23831025412962

1111

12837136

111

41010

22

11

11

444

111

111

111

111

44

11

11

11

11

11617117

11212

11

11111

222

111

111

111

111

111

111

222

111

111

333

111

111

111

222

111

111

111

111

111

222

111

111

798

111

222

111

111

111

111

11

11

111

111

11

11

111

111

11

11

2154644412592

1

1

1

28881

11111

666

111

111

111

111

111

111

1111

1111

11

11

11

3891310

11111

11111

11111

11111

111

25617

1111

11

11

11

11111

111

111

111

111

111

5596

11

11

2131

11

11

111

11

11

1111

1221

111

111

1111

112111

1

1

111111

111111

111

111

111

111111

11

11

11

1

1

1

41317413

31014310

111

11111

111

111

11

111

1111

11

11

1

11

1

11

111

11

11

111

111

11111

11111

111

3992138

122112

111111

111

155114

111

111

11

111

111111

111111

111111

111

222

222

111

111

2441141

11111

11111

111111

111111

111

111

111

228110

228110

11516

11516

11

11

11

111

11

111

1111

1111

12

12

1

11

11

111111

1590105546125

1590105546125

1111

1488103546123

1

1

6374611249

11

111111

11

11

111

11

111

111

111

11

111

1

111

1111

1

111

111

111

111

111

111

111

111

11

1111

111

111

111

1111

111

111

111

111

111

111

111

11

111

111

111

111

11

111

11

111

111

111

11111

111

112

11

11

2

1

1

111

111

11111

11111

4302512331

111

11

111

111

111

11

11

111

111

11

11

111

1

222

11111

111

11

1111

111

111

111111

111

111

111

1111

111111

111

111

111

111

1

111

111

111

11

11

31120326

1

1

11111

1111

11

111

11111

111

11

111

1

11

111

1

1

11

222

11

1

111

111

111

11

11

11

234

111

111

11

1

1222

111

1111

111

111

111

1144

1144

1144

111

111

111

133

11

12

1

11

11

11

1

1

111

11

1

1

1

1

1151

1151

1151

13

1

1

1

1

1

1

1

121

111

111

1

1

11

11

11

11

11

11

2

1

1

1

1

1

1

12

1

2

2

2

1

1

1

1

22

22

22

21

11

11

1

1

1

29371910641112542755

173451

4

4

1

1

1

1

1

1

1

1

1

1

413

413

11

11

22

1

11

1

11

1

1

31328

1111

13

3

1

1

1

1

1

1

8

1

1

1

1

1

1

1

1

1

1

1

1

1

11

11

1

1

1

11

11

25

1

1

2

1

1

22

11

11

4

3

1

1

1

1

2

2

1

1

1

1

6

1

5

3

1

1

1

1

1

1

1

1111

311

1

1

1

27

11

1

1

1

1

1

1

11

2

1

1

2

1

1

1

11

1

1

29217

28216

10

9

1

1

1

1

1

1

1

1

1

1

3

3

1

1

1

12211

1

11211

1

1

1

1

11

1

1

11

1

1

1

1

1

3

3

1

1

1

1

22

22

1

11

1

11

1111

1210

112

11

11

11

3

3

2

1

1

1

1

13

12

1

1

11

1

1

1

1

1

346716714567

1127

14

4

1

1

1

1

1

1

6

1

1

1

1

1

1

3

1

1

1

3

2

1

1

1

1

1

1

1

1

118

1

1

1

1

1

1

11

11

1

1

11

2

1

1

55763

11122

17

1

1

1

1

1

11

1

2

1

1

11

11

1

1

2

1

1

2

1

1

1

1

11

11

2

1

1

1

1

1

1

1

1

43540

111

111

4

1

1

1

1

111

1

1

21220

1

1

1

11

1

1

1

111

1

1

1

11

1

1

1

11

1

1

1

1

11

11

2

1

1

1

1

1

1

1

1

1

1

2

1

1

1

1

1

1

111

111

1

1

111

11

11

11

11

11

11

266215813434

1

1

2

1

1

112

1

111

431461274

2021227

1

111

11

1

111

111

111

1111

111

111

11

111

1

1111

11

11

1

111

111

11

1

111

111

111

111

1

1

111

11

1

44

11

11

11

11

171112

11

111

111

111

11

11

111

111

1111

1

111

11

3

1

1

1

3

1

1

1

11

11

123

111

11

1

2

1

1

111

111

2

1

1

1

1

1111

7

1

1

1

1

1

1

1

133

111

11

11

1215

1111

11

1

1

1

12

11

1

115

1

1

1

111

1

5

1

2

1

1

1

1

1

1

1111

1

1

1

1

1313

11

11

1111

1111

1111

1

1

1

1

2

1

1

83475

1

1

1

1

4

1

1

1

1

111

1

1

1

1

114

1

111

1

1

1

1

2

1

1

1

1

2

1

1

1

1

1

1

3

1

1

1

1

1

2

1

1

3

1

1

1

1

1

226

1

1

111

111

1

1

1

1

1

1

3

1

1

1

13

11

1

1

1

1

1

1

11

11

1

1

1

1

4

1

1

1

1

1

1

57

1

1

11

11

11

11

11

1

1

12

1

1

1

1

1

1

1

1

1

1

1

1

1

1

459

11

1

1

2

1

1

1

1

2

1

1

1

1

5

1

1

1

1

1

1

1

11

11

4

1

1

1

1

8

1

1

1

1

1

1

1

1

3

1

1

1

1

1

2

1

1

3

1

1

1

1

1

216

1

1

11

1

1

1

1

1

1

1

1

11

1

1

1

1

1

1

4

1

1

1

1

1

1

111

111

2

1

1

12

1

11

11

11

42199182

1115

1

1

1

11

1

1

11

1

1

1

1

1

1

1

1

1

1

1823

11

1

1

11

1

11

1

111

1

1

11

1

11

1

1

1

1

11

1

1

11

1

1

115459

11

111

11

11

111

111

11

1

11

11

11

11

11

11

11

11

11

11

11

1

1

11

111

11

111

11

11

11

11

11

111

11

111

11

1

11

11

11

11

11

11

11

11

1

111

11

111

11

11

11

11

11

11

11

111

11

11

11

111

1122

11

1111

11

11

11

11

11

11

52225

11

11

11

11

111

11

11

1

111

111

11

11

11

11

11

11

1

11

11

111

11

1

111

11

11

1

1

1

1

111

1

1

428

1

1

1

1

1

1

11

1

1

1

1

11

1

1

1

1

1

1

1

1

11

1

1

1

11

1

1

1

3

1

1

1

11

11

1

1

11

1

1

1

1

1

1

1

1

1

1

1

345

11

111

111

11

11

1

1

1

1

38

11

1

1

11

1

1

1

11

1

1

111

39

8

1

2

1

1

1

1

1

1

1

1

1

1

1

1

1

16

1

3

1

1

1

11

1

1

1

1

1

1

1

1

1

1

1

1

1

3

1

1

1

1

1

1

2

1

1

1

1

2

1

1

1

2

2

1

1

5

1

1

3

1

1

1

1

1

4

4

4

4

1

1

1

1

15

14

14

14

1

1

1

11

1

3

1

1

2

2

1

1

1

11529646194151335

1

1

1

1

5

5

1

4

1

1

1

1

111

111

11133350

11133250

1

1

11

11

111

111

11

11

1

1

111

111

11

11

16

1

11

1

1

1

1

111

111

11

111

111

1

1

1111

11

11

1357

111

11

1111

1

111

11

1

1

1

11

11

11

11

22

11

11

11

11

235

1

11

111

1

111

113

1

11

11

11

11

1

1

2688

11

1111

111

111

11

111

111

1111

1

1

1

11110

1114

12

1

11

111

111

1

6

1

1

1

1

1

1

1

2

1

1

1

1

2119

1115

4

1

1

1

1

1

1

1

1

1

1

1

1

1

1

11

1

1

1

1

1

1

12

11

1

12

1

11

11

1

1

1

2

1

1

12

12

1

11

1

1

2262891121

2262891121

1

4122431

1

111

11

111

111

11

1

11

1

111

111

111

111

11

1

11

2222

11

1

111

11

111

111

111

11

1

11

1

11

111

1444

111

111

1111

111

2

1

1

1

1

113

1

111

1

164357176

1111

1

11

1111

11

111

1

1111

11

111

1111

1

111

11

11

11

111

111

11

1

1111

11

1

11

111

111

111

111

111

1

111

111

11

111

11

1

11

11

111

111

1

11

111

111

11

11

111

111

1111

111

111

111

111

1

1111

111

1

111

111

111

1

1

111

111

1111

1111

111

1

111

1111

111

111

111

11

111

1

1233

11

111

1111

151101574236

1922128

111313

111

111

111

111

111

111

111

11

111

111

11

111

111

111

111

4

1

1

1

1

6616

111

111

111

111

1111

111

11

11

113

1

1

111

213141

116

1

1

1

1

111

1

1119

1

1

1

1

1

1

11

1

1

1

1

1

1

1

11

1

1

1

1

111

111

9

1

1

1

1

1

1

1

1

1

15

11

1

1

1

1

1

1

1735136

111

111

1111

1111

41414

11

11

11

111

11

11

11

111

11

11

11

111

11

111

233

111

111

11

222

111

111

61112

11

11

111

111

11

111

111

11

111

111

11

1

133

11

111

11

96264189

111

111

111

111

1

1

181111

111

1111

111

111

111

111

111

11

111

11

11

91010

111

111

111

11

111

111

111

111

111

111

111

111

11

11

11

11

111

111

222

111

111

122

111

11

291010

111

111

111

1111

11

111

111

111

111

1111

111

111

11

11

55

11

11

11

11

11

12313

11111

11

111

111

111

22

11

11

22124

11

11

1

11

11

11

11

11

11

11

11

11

11

11

11

11

11

111

11

11

11

22

1

11

11

11

11

1111

1111

1111

2234

111

1111

111

1

22

11

11

242731

1133

11

111

111

12

1

11

111113

1

1

11

11

111

11

11

11

111

11

11

11

11

11

11

11

1

1

122

111

11

188

11

111

11

11

11

11

11

11

2325

11

11

223

11

111

11

111

111

115

1

1

111

111

1

1

1

1

1

1

111

22

11

11

11

11

4

4

1

1

1

1

1

1

1

441423

441422

111

111

2

1

1

421312

111

11

11

111

111

11

1

111

11

111

111

11

11

13

1

11

1

4

1

1

1

1

1

1111

1

1

434563311126

1

41437331193

2113

1

11

1

1

1

11

1

1

11

1

1

1

1

323

11

111

111

1

1

13273527

11

11

11111

11

11

111

11

11

11

11

11

11

11

111

11

1111

11

1111

11

11

111

11

11

111

11

11

111

1

1

9

1

1

1

1

1

1

1

1

1

1

1

1111

11

11

1343320

1

1

11

1

11111

11

1

1

1

1

1

1

11

1

1

1

1

1

11111

1111

5118

111

11

11

11

11

11

1

1

3

1

1

1

5

1

1

1

1

1

201932

111

111

1

1

214

1

111

1

11

1

1

1

1

12

11

1

111

111

111

1

1

11

11

1

1

1

1

444

111

111

111

111

11912

11

111

111

222

111

111

111

111

1

11

111

1

1

81923126

1

1

11

1

1

1

11

111

111

111

2101025

4

1

1

1

1

1

1

1

1

5

1

1

1

1

1

1

1

1111

2

1

1

1

1

1

1

5

1

1

1

1

1

99

11

11

11

11

11

11

11

11

11

3

1

1

1

1

1

44583

36

1

1

11

11

11

1

1

1

1

1

1

1

1

1

1

4164

1

1

1

1

1

1

1

1

1

1

1

1

1

1

1

1

1

1

1

1

111

1

1

11

1

1

1

1

1

1

1

1

1

1

1

1

1

1

1

1

1

1

1

1

11

1

1

1

1

1

1

1

1

1

1

1

1

1

1

11

1

1

1

1

114

1

1

1

111

2

1

1

111

111

2

1

1

1

1

1

1

1

5

1

3

1

1

1

1

1

122

122

111

11

232

121

111

1

111

111

1

1

1

13

12

11

1

1

1

1

1

1

179226

2

1

1

1

1

1118

1118

1

1

1

1

1

1111

1

1

127

12

11

1

2

1

1

2

1

1

111

111

111

111

4417

1

1

111

111

11

11

11

11

111

111

111

11

11

227

227

1

1

1

1

1

1

224

1

111

1

111

1111

20305621138

22815

2

1

1

11

11

22

11

11

225

1

1

111

1

111

224

11

11

11

11

11

71826128

11

11

1233

1111

11

111

1

1

291213

11

111

111

111

1

1

111

111

11

111

1111

111

111

11

111

111

1

1

111

111

1111

1111

1233

111

111

111

11

11

11

11

1111

1111

1111

57926

111

56825

1

11

1

1

1

1111

1

1

1

11

1

1

1

11

1

111

111

11

1

1

1

1

11

11

1

11

1

1

11

236

11

8

1

1

1

1

1

1

1

1

18

1

1

1

1

1

1

1

1

1

1

1

1

1

1

1

1

1

1

19

1

1

1

1

1

1

1

11

1

3311227

339225

11

1

11

1

1

11

1

1

1

111

1

1

1

11

1

1

11

1

1111

1

111

11

1111

11

1

22

11

11

1

1

2

2

1

1

1

1

11

1

1

1

1

111

111

1

1

1

11

11

11

11

22353

11

22221

1

1

1225

1

1111

1

111

1

1

1

1

1

1

2

1

1

2

1

1

11

11

1

1

1

1

1

1

1

1

2

1

1

1

1

22

1

1

3

1

1

1

1

1

1

3

1

1

1

1

1

3

1

1

1

1

1

1

1

1

1

1

1

1

1

4

1

1

1

1

1

1

1

1

1

1

1

1

2

1

1

1

3

2

1

1

1

1

1

14

13

1

1

12

11

1

1

1

1

328

1

323

2

1

1

321

1

1

1

1

1

1

1

1

1

1

1

1

1

1

1

11

1

1

1

1

1

1

1

4

1

1

2

1

1

1

111

111

111

1243222

1

1

1

2

2

1

1

1043220

1013200

1

1

1

1

1

1

1

1

1

1

1

11

11

1

1

1

1

1

1

1

1

1

1

1

1

1

1

1

1

1

1

1

1

1

1

1

1

1

1

1

1

1

1

1

1

1

1

1

1

1

1

1

1

1

1

1

1

1

1

1

1

1

1

1

1

1

1

1

111

1

1

11

1

1

1

1

1

1

1

1

1

1

1

1

1

1

1

1

1

1

1

1

1

1

1

1

1

1

1

1

1

1

1

1

1

1

11

1

1

1

1

1

1

1

1

1

1

1

1

1

1

1

11

1

11

1

1

1

1

1

1

1

1

1

1

1

1

1

1

11

1

1

11

1

1

1

1

11

1

1

1

1

1

1

1

1

1

1

1

1

1

1

1

1

1

1

1

1

1

1

1

1

1

1

1

1

1

1

1

1

1

1

1

1

11

1

1

1

1

11

1

1

1

1

1

1

1

1

1

1

1

12

11

1

1

1

22

11

11

1

10

1

1

1

1

1

1

1

1

1

1

2

1

1

1

1

1

1

1

2

1

1

51634

1

1

43

43

11

1

1

11

1

418

1

1

2

1

1

2

1

1

5

1

1

1

1

1

1

1

1

1

23

11

1

11

1

22

11

11

11211

1

11

11

1

113

1

1

111

1

1

14

1

1

1

11

1

31388

31388

1

1

111

114

1

1

1

11

1

1

1

1

1

1

1

1

1

1

2

1

1

1

1

12

1

1

1

1

1

1

1

1

1

1

1

1

254

1

1

1

1

1

1

1

1

11

1

1

1

1

1

1

1

1

1

1

1

1

1

1

1

1

1

1

1

1

1

1

11

1

1

1

1

1

1

1

1

1

1

1

1

1

1

1

1

1

1

1

1

1

1

1

1

11

11

11

11

953504281835772

13

3

1

1

2

1

1

1

116

111

11

11

1

1

5

1

1

1

1

2

1

1

1

1

331291511149

111

24636980

22

1

1

11

1111

1111

4431

111

111

111

111

11

11

1

1

11

11

111

111

11

11

1111

1111

111

111

445

111

111

1

111

111

1

1

221

11

111

11

11

1111

1111

1

1

11

11

1

1

111

111

111

111

1

1

1

1

1

1

1332

11

111

11

1

1

31

1

1

11

1

1

221

11

111

1

1

111

111

1

1

11

11

111

111

1

1

11

11

11

11

111

111

1

1

11

11

1

1

1774

11

111

111

11

111

11

1111

11

11

11

11

221

11

111

1

1

11

11

1

1

1111

1

1

2

1

1

11

11

111

111

5316

1

11

1

1

1

1

11

1

111

1

2

1

1

1

1

1

11

1

11

11

1111

1111

2

1

1

1

1

1

1

432

11

111

11

11

121

11

11

1

1

1

1

1

1

111

111

11

11

1

1

11

11

11

11

3

1

1

1

1

1

111

11

1

1111

1111

11

11

11

11

1

1

96581168

111

111

111

111

11

1

1

22

11

11

1

1

1233

1

11

11

11

11

113

1

1

111

111

111

1221

11

1111

22

11

11

2

1

1

11

11

1

1

11

11

1

1

784

1

11

111

11

111

111

11

111

121

1

1

1

1

22

11

11

1111

1111

2

1

1

1

1

11

11

1

1

3

1

1

1

1

1

112

111

1

1

1

11

11

2222

1111

1111

11

11

11

1

1

552

11

11

111

11

111

111

111

495

1

1

111

1

111

111

1

1

1

111

1

1

1

1

22

11

11

11

11

11

1

1

1

1

1

1

1

1

1

1

1

11

11

11

11

222

111

111

556

1

11

111

111

11

111

1

1

11

11

1442

11

11

111

1111

67

11

1

11

11

11

11

11

1111

1111

1

1

1

1

112

1

111

2

1

1

4517

4517

111

112

11

11

1

1

2313

11

111

1111

114

114

3

1

1

1

111

1113

1111

2

2

1

1

12

11

1

1

911696516182

47961516110

2212

1111

111

111

111

111

111

1

1

11

11

11

11

2114

11

11

111

1

111

1

1

114101215

1

1

11

11111

11

11

1

111

111

111

1

1

11

11

111

1

111

11

1111

1

111

1556

1

111

111

1111

111

1

11

2433241163

11

111

11

1

11111

1111

11

1

111

111

11111

1

1

1

111

1

111

1

11

111

1

111

11

1

1111

11

11111

111

1

11

111

111

1111

1

1

11

1

1

111

11

111

1

1111

111

111

11111

2

1

1

111

11

11

111

1

111

1111

1111

1

111

1

1

1

11

11

1111

1

11

76113

11

1

11

1

11

1

1

111

1111

1

11

1

11

11

11

11

11

11

111

5312862

12

11

1

214

1

11

1

1

11

227

1

1

11

11

1

11

1

1

11

1111

1111

223

111

111

1

112

1

111

13

1

11

1

113

1

111

1

13

1

11

1

554

11

1

11

111

11

111

1

11

11

445

111

111

111

111

1

1

1

333

111

1

11

111

13

1

11

1

1

1

1

1

26814

111

1

11

1

1

1111

111

11

11

1

11

1

11

11

111

558

222

11

1

111

11

1

1

3

1

1

1

11

11

111

111

1

1

1111

517201102

1

1

6519

217

11

1

11

11

1

1

1

2212

111

1111

22

11

11

191275

1

1

111

111

1

1

11

11

1

1

111

111

7

1

1

1

1

1

1

1

11

11

11

1

1

1

1

1

1

1

1

1

1

1

1

23

11

11

1

17

1

1

1

1

11

1

1

1210

1

1

1

1

11

1

1

111

1

1

1

1

2419

11

1

1

11

1

1

1

1

111

1

1

1

11

11

1

1

1

1

1

11

11

1

1

8

1

1

1

1

1

1

1

1

1

1

11

1

1

11

11

42314

1

1

11

11

11

11

1

1

1

1

11

11

1

1

1

1

1111

14

1

1

11

1

11

11

1

1

2

1

1

1

1

1

1

11111

11111

11111

11111

416613812281

8125

1

1

719

1

1

11

1

1

1

1

1

11

11

1

1

1

1

1

11

1

11

1

1

1

12

1

11

1

1

1

1

11

113

11

11

11

11

1

1

1118

1

1

12

1

11

1

1

1

1

1

1

1

111

111

171112

123

1

11

111

455

111

111

111

11

111

123

1

11

111

221

11

111

12218

1117

1

1

1

1

1

11

1

11

18

1

1

1

1

1

1

1

1

1

13

11

1

1

1111

1111

1

1

1

12

12

1

11

238117

1

1

2

1

1

13

1

1

11

1255

11

111

1111

11

11

1316

11

1

1

1

11

1111

12

11

1

1

2

2

1

1

111

1

1

1

1

1

1

1

1

1

1

1

1

161441

122

111

11

1

1

11

11

11

11

11

1

1

4718

1

1

1

1

1

1

1

11

11

1

11

1

11

111

11

1

1

11

11

1

12

1

1

1

1

1

1

1

3

1

1

1

11

11

1

1

1

1

11

11

6

1

1

1

1

1

1

269

154

11

11

1

111

11

1

1

3

1

1

1

111

213

213

1

11

111

11

11

11

11

11

5

1

1

1

1

2

1

1

1

1

1

1

14

1

1

1

1

12

11

1

4

4

1

1

1

1

311

1

1

1

1

29

1

1

1

1

1

11

1

1

1

1

1

1

11

11

11

241516

11

11

1

1

22

11

11

241212

111

111

11

11

11

111

111

11

11

1111

11

11

11

11

11

13

1

1

11

11

1

1736701185

11

11

11

11

1

1

1123

11

111

11

11

11

2

1

1

1334

1111

1

111

111

121

1

111

123

11

11

11

1226501158

11

11111

1111

11111

1111

11

111

11

11

11

1111

111

1

11

111

111

111

11

11

11

11

111

1

1111

11111

1

1

11111

111

1

11

111

11

11

111

11

1

1111

1

11

1

11

11

11

11111

111

1111

11

11

111

1

111

1111

11

1111

11111

111

11

1

11

1478

11

111

11

1

111

11

1111

11

1

1

1

111

1

1

1

1

1

1111

2

2

1

1

1

1

1

1

1

1

1

1

2121212326

111

2111112325

55117

1

1

11111

111

111

111

111

222110

11

1

11

11

1

11

11

11

11

1

111

111

1115

1

1

1

1

11

11

22112

111

11111

11

11

11

11

1

1

1

93340233

1242

1111

131

121

11

11

11

11

1

131

1

121

111

111

111

1

222

222

111

111

111

111

111

111

2322

1

1

2221

111

1

1

1

111

1

1

1

3775

1111

2664

1553

1

1

1

1

1111

111

111

11

11

1

1

11

11

1111

11111

1112

1111

1

1

11111

544

11

11

11

11

212

212

11

11

111

111

111

91412

33

33

33

11

11

11

9119

222

111

111

111

11

11

11

564

454

111

111

111

111

1

11

11

111

111

111

111

111

111

1

1

1

1

1

113

11

12

12

11

11

1

1

1

242475130

1111

222273128

1121

1121

1121

1

1111

1111

151502

151502

141492

1

1

11

1

1

1

1

11

11

11

1

11

1

1

1

1

1

11

1

1

1

11

1

1

1

1

11

11

1

11

11

11

1

1

1

1

1

11

1

111

1

1

1

11

1

1

1

11

1

1

1

1

51920124

222

222

111

111

111

310109

111

111

1111

111

111

1111

1222

111

1111

222

111

111

111

111

11

11

4

1

1

3

1

1

1

26718

26718

111

11

11111

11

1

1

1

111

111

111

1111

2222

2222

2222

2222

2222

1111

1111

13283215635

111

111

111

111

111

1

1

1

1

2125

1

1

1111

1

1

1

1

112

112

11

11

11

11

1111

5232315523

1333

1222

1111

111

111

111

4191915519

4181815518

222

111

111

1222

111

1111

111111

111111

1661336

111111

111

11111

111

111111

111

111

111

133113

111111

111

111

111

111

111

111

111

111

111

31414

1111

2313

2313

111

111

111

111

1111

38

27

27

16

11

1

1

4

1

1

1

1

11

11

34411

34411

11

11

11

1

1

37

11

26

3

1

1

1

11

11

11

1

1

323

323

213

1

111

11

1

1

1

1

1

1

1

1

1

11

11

11

11

11

11

2222

1111

1111

1111

111111

111111

2546

2435

1111

1324

1112

1

1

1111

11

11

111

111

11359879942542517

111

111

4612716538824

1111

1

1

1

1

43615

12311

12311

111

1111

1

1

3133

1

1

2

1

1

11

11

11

111

111

1

1

1

1

4011614937734

11111

1

1

1

2

1

1

33

33

11

11

11

4162028

111

111

11

11

11

11

111

111

1

1

11

11

2131

11

111

11

111

111

1

1

1

1

111

111

1111

231

11

111

1

11

11

1

1

111

111

11

11

111

111

1

1

11

11

172734327

11

11

2

1

1

141919219

1

1111

1

111

11

1111

11

1

1

1

111

111

1

1111

11

11

1111

111

1111

11

1111

11

111

11

1111

1

1

111

1

1

111

11

11

11

11

11

11

1

1

221

11

111

1

1

11111

1

1

221

11

111

11

11

1

1

1

1

1

1

1

1

1

1

111

111

21

21

1

11

13

1

1

12

1

11

10546532243

111

111

11

11

111

111

12

1

11

11

11

11111

11111

11

11

22

11

11

11

11

11

11

111

111

11

11

11

11

11

11

1111111

1

1

111

111

331

11

11

111

1

1

111

111

443

11

111

111

111

1

1

1

1

11111

11111

111

111

11

11

11

11

111

111

15614

111

11

11111

111

1

111

11111

11111

111

111

1111

1111

111

111

11

11

1

1

23

11

11

1

145

11

11

1

111

11

22

11

11

44

11

11

11

11

22

11

11

11

11

11

11

1111

1111

1

1

1

332

11

11

222

111

111

12

1

1

11

11

211

1

1

111

11

11

11

11

11

211

111

1

1

32

1

1

11

11

2

1

1

41

1

1

21

1

11

1

1

3341

1

1

111

1231

11

111

11

11

11

1782

1672

111

111

1

1

111

441

11

111

11

11

11

11

11

11

11

624495981732113

131711956946

120215

11

11

771

11

11

11

111

11

11

11

11

11

551

11

1

111

1

11

11

111

111

1441

11

11

11

1111

22

11

11

772

1

1

11

11

11

11

11

11

22

1

1

2

221

111

11

939483275

673

111

1

1

1

11

1

11

11

1

11

933413245

1

1111

11

1

1

111

11

111111

111

11

111

11

1

11111

11

11

111

11

11111

11

1111

11

111

11

1

11

11

11

1

11

1111

11

111

11

11

1

1111

11

111

11

11

111

111

11

11

111

11

11

37785337

2211

1

11

111

1

1

66

11

11

11

11

11

11

55

11

11

11

11

11

354

11

11

11

111

111

12

11

1

78

11

11

11

11

11

11

1

11

221

111

11

9113

1

11

1

11

1

11

11

111

11

11

1

1

111

572

11

11

11

11

11

1

11

1

111

111

1666

111

111

111

111

1111

111

112

1

111

10722

11

11

1

11

1

1

1

11

1111

1

1111

12

11

1

666

111

1

111

111

222

11

11

11

11

11

1983

1

11

111

11

1111

11

111

11

11

122

11

111

4

1

1

1

1

11

11

2632231

182014

1

11

1

111

11

111

11

1

11

111

11

111

11

111

111

111

111

11

11

111

111

111

1

1

6107

11

11

11

1

11

1

1

111

111

1

1

11

1

1

1111

1111

11

11

11

1111

48277402112267

610

1

1

2

1

1

67

11

11

1

11

11

11

11

1

1

2811

11

11

22

11

11

11

11

11

11

1

1

1

1

11

11

2

1

1

12

11

1

1

1

1

1

451

451

11

11

11

1

1

11

231101309105

111

111

342

1

11

1

1

1

1

11

3131413

1111

111

1111

111

111

111

111

111

111

1111

11

11

111

111

333

111

111

111

11

11

1691106984

111

11

111

111

11111

111

111

11

11

111

11

111

1111

11

11

11111

111

1111

111

11

111

11

111

111

11

11

111

11

111

111

11

11

111

1111

111

111

11

11

11

111

1

11

11

1111

1111

11

111

11

111

111

111

11

1111

111

111

111

111

1111

111

111

111

111

1

11

11

11

111

1111

1111

111

11

11

11

111

1111

111

11

111

11

111

1111

222

111

111

11111

11

11

111

111

11

1111

11

11111

111

111

1111

1111

11111

111

111

111

1111

11

11

111

11

1

1

1111

342

111

11

11

11

310126

351

11

11

1

111

1

1

1

663

11

111

1

11

11

11

111

11

11

22

11

1

1

1111

1111

176

11

11

22

11

11

142

11

1

1

111

1

1

333593

111

111

43

1

11

1

11

1

222762

11

11

1

1

11

1

1

11

1

1

111

1

1

1

1

1

1

1

1

1111

1

1

1

1

111

1

1

1

13

1

11

1

171311892914

33

11

11

11

1232

11

11

11

11

11

1

1

11

11

11

11

1

1

5

1

1

1

1

1

1

1

8816

11

11

111

1111

111

111

111

111

11

11

331

111

11

11

1

1

1

1

294

111

11

11

1

1

1

1

1

11

1

1

1

22

11

11

11

11

1

1

232

1

111

111

322

11

1

11

11

1861

11

1

11

111

11

1

111

11

11

11

2774

1111

111

111

11

1

11

1

11

11

11

11

111

111

111

111

221

111

11

11

11

11

1

1

22

11

11

1

1

22

11

11

17813

111

11

11

11111

11

11

11

11

31635133

11

1

1111

11

1

11

111

11

11

1

1

1

11

1

1

1

1

11

111

1

1

1

1

1

1

1

11

1

11

1

1

1

11

111

1

11

11

11

111

111

11

1

1

2

1

1

11

11

1331

1111

11

11

214

1

1

11

1

1

1

1

1

331

111

11

11

11

11

253

1

1

1

1

111

11

1

342

111

11

11

11

1552

111

11

111

11

111

14

11

1

1

1

32

11

11

1

1

1

1

1

2810

1

11

11

11

1

11

1

111

11

11

11

1

1

341

111

11

11

1

232

11

111

11

12

1

11

2272

11

1

11

1

11

11

111

1741

111

111

11

1

11

1

1

11

11

11

11

11

11

652

111

11

11

1

111

11

1

1

11

11

332

11

111

111

1

1

232

11

111

11

111

111

1

1

11

11

1

1

11111

11

11

11

11

11

29168

19112

11

1

1

1

1

991

11

11

111

11

1

11

11

11

11

22

11

11

33

11

11

11

11

155

155

1

1

11

1

1

133

1

1

11

1

1

1

1

1

1

110144

11

10134

1

32

1

1

22

11

11

7104

111

11

11

1

1

1

1

11

11

11

11

1

1

1

1

1

1

11

11

1

1

22

1

11

1

1126

115

11

11

11

1

1

1

4

1

1

1

1

2

1

1

111
