# Supplementary material for: Simulated digestions of free oligosaccharides and mucin-type O-glycans reveal a potential role for Clostridium perfringens
Source: Sci Rep. 2024 Jan 18;14:1649. doi: 10.1038/s41598-023-51012-4 (PMC10796942; doi:10.1038/s41598-023-51012-4)
Supplement: Supplementary file 2 — Supplementary Information. [file 41598_2023_51012_MOESM2_ESM.zip › gutGH-SI/Krona/UniProt-EC-Krona-graphs/all-EC.krona.html]

Javascript must be enabled to view this page.

magnitude
magnitudeUnassigned

EC\_3.2.1.111
EC\_3.2.1.140
EC\_3.2.1.18
EC\_3.2.1.22
EC\_3.2.1.23
EC\_3.2.1.49
EC\_3.2.1.50
EC\_3.2.1.51
EC\_3.2.1.52
EC\_3.2.1.63
EC\_3.2.1.97

3293832074138104602675669261

3293832074138104602675669261

2222

2222

2222

2222

2222

1111

1111

55610

3347

3347

111

2336

1111

3

1

1

1

111

111

1111

1111

1

1

1

1

1

1111

223

111

112

112

11

11

11

11

2222

1111

1111

1111

22

11

11

11

11

11

1

1

933130

933130

519124

519124

418123

11

11

11

1

11

11

1

111

2

1

111

11

1

111

11

11

1

11

11

1

11

1

1111

11

111

111

11

11

11

4135

4135

222

111

111

8

1

1

1

1

1

1

1

1

232

11

111

11

1

1

1144

1144

1144

111

111

111

133

11

12

1

11

11

11

111

1

1

1

1

11

2546

111

2435

1324

1112

1

1

1111

111

11

11

1111

2371

2371

2371

225

1

1

222

11

111

1

1

1

1

121

111

111

1

1

22

22

22

21

1

1

11

11

1

11021114

11020113

541

541

111

111

1

1

22

11

11

11

11

1412111

1167

112

11

11

155

11

11

11

111

11

2513

11

2311

1111

11

1

11

11

111

111

111

111

111

3

3

2

1

1

1

11

2

1

1

1

1

1

1

1565

111

111

111

111

111

1454

1

1444

1122

1111

11

11

1

222

222

111

111

242475130

222273128

1121

1121

1121

1

1111

51920124

222

222

111

111

111

4

1

1

3

1

1

1

26718

26718

11

111

111

1

111

1

11111

111

1

11

310109

1111

111

111

1222

111

1111

111

111

222

111

111

111

111

1111

11

11

1111

151502

151502

141492

1

1

1

11

1

1

1

1

11

1

11

1

1

1

11

1

11

1

1

1

1

1

1

11

1

1

1

1

11

1

1

1

1

11

11

1

1

1

111

11

1

11

11

1

11

11

11

1

1

1

1

1

1111

1111

16616

444

111

111

111

222

222

222

111

111

11111

111

111

111

111

11

11

11

11

11

11

1145684898913750124

11

11

11

11

11

2152211

11

1152111

331

11

11

1

1

22

11

11

111

111

111

111169

1

1

11

11

111

111

111

11

11

1

1

211

1

111

221

111

11

1

1

1

1

1

1

22

1

11

1

1

1

1

1

2

1

1

122

11

111

1

1126

111

115

1

1

1

11

11

11

4

2

1

1

1

1

1

1

210179

155

155

1

1

11

133

1

1

11

1

1

1

1

1

110122

11

10101

11

11

22

11

11

22

11

11

33

11

11

11

111

1

11

11

11

11

1

1

1

1

2

2

1

1

1

16817121481121198

1

1

1

1

1

1

1

1

1782

1672

441

111

11

11

11

1

1

111

111

111

11

11

11

16216019881111108

11

11

12

1

1

11

11

72025213

11

11

11

11

2311

11

1

1

111

11

11

111

111

1

1

11

11

111

111

111

111

111

111

1111

1111

1

1

111

111

111

111

1

1

1

1

11

11

1

1

1111

1111

1111

11

11

11

11

1

1

2131

11

111

11

111

111

111

111

48951

111

27851

111

111

111

11

11111

11

11

111

1

1

1

1

1

11

11

11

211

1

1

111

1

1

1

211

111

1

1

2

1

1

33

33

11

11

11

332

11

11

222

111

111

11

11

21

21

11

1

32

2

1

1

11

11

1

1

162

1

1

1

1

131

11

1

11

1

1

1

1

11

11

11111

20809173394

233

111

111

11

11

11

1111

1111

1111

1111

22

11

11

12

1

11

2561

1111

11

111

1

11

11

11

11

11111

11111

1111111

443

11

111

111

111

111

111

1331

11

1111

11

111

111

1

1

11

11

22

11

11

111

111

11

11

11

11

111

111

111

111

23

1

11

11

122

111

11

311123101

111

1

111

11111

111

11111

111

111

1111

11111

11

111

13313

11111

111

111

25512

11

11111

11

11

1111

11

11

111

111

1

1

221

11

11

1

111

111

11

11

22

11

11

111

111

111

111

11

11

23

1

11

11

331

111

11

11

11

11

11

11

111

111

111

111

44

11

11

11

11

1

1

11111

11111

11111

11111

1

1

13

1

1

12

11

1

1253545116392

11

11

11

11

1

1

1

1

221

11

111

11

11

1

1

1

1

1

1

1212528114291

1111

11

11

1

111

11

1

1

111

111

111

1

1111

11

111

111

1111

111

1

1

111111111

11

1111

11

11

11

1111

111

1

11

111

1

11

1

1111

1111

111

1

111

1111

111

1111

1

1

1

1

133121

11

111

111111

1

1

2

1

1

11111

11

11

221

111

11

43615

12311

12311

111

1

1111

3133

1

1

2

1

1

11

11

111

111

11

1

1111

714856392535216

22201226141168

121215

1

1

22

11

11

551

111

11

1

11

11

1

771

11

11

11

11

11

111

11

11

11

11

11

1441

11

1111

11

11

111

111

18556511447

17465411377

11

1111

111

1

11111

111

11

111111

11

11

11

111

11

11

11

1

1111

11

11

1111

111

11

1

1111

1

11

11

11111

111

111

111111

11111

1111

11

1

1

1111

111

1

11111

11

111

111

111

111

11

11

111111

11111

1

11

1

11

11

1111

111

111

11

11

11

1111

111

19117

1

1

11

11

1

11

1

1111

111

111

1

11

111

11

11

2935261

1

1

1111

1111

6107

11

11

111

1

11

1

11

1

1

1

11

111

1

1

212317

111

111

111

11

111

1

111

111

111

11

11

11

111

111

11

111

1

111

11

111

11

1

111

111

11

11

11

11

38695339

10722

1

1

1

11

1111

11

1

1111

11

1

11

11

11

2211

1

111

11

111

111

112

111

1

354

111

11

111

11

11

332

111

11

111

1

1

572

1

11

1

11

11

11

11

11

55

11

11

11

11

11

1983

11

11

111

11

1111

11

11

1

111

11

11

12

11

1

122

111

11

89

11

11

11

11

1

11

11

11

11

4

1

1

1

1

12

11

1

15174

11

111

1

11

1

111

11

1

1

11

11

11

111

11

11

11

11

11

1

11

11

666

111

1

111

111

222

11

1666

111

111

111

111

111

1111

78

11

11

11

11

11

11

11

1

882

221

111

11

1

1

11

11

33

1

2

1

1

1

11

11

11

11

48283412112358

342

11

11

111

11

171331932945

11

11

11

11

294

1

1

1

11

1

11

1

11

111

1

11

1

1

1

1

11

11

22

11

11

8816

111

111

11

111

1111

111

11

111

111

111

1

1

322

11

1

11

11

11

11

1

1

11

11

1

1

652

111

11

1

11

11

111

2810

1

111

11

11

11

11

1

11

1

11

11

1741

1

1

1

11

11

111

111

1

1

11

1

1

1232

11

11

11

11

111

111

11

11

232

11

11

111

11

11

33

11

11

11

221

11

111

1

1

341

111

1

11

11

232

11

11

111

11

11

11

11

1971

11

11

1

11

1

11

111

11

111

1331

1111

11

11

2

1

1

1

1

1552

111

111

111

11

11

22

11

11

1

1

14

1

1

1

11

11

11

232

1

111

111

111

111

1

1

2272

11

11

1

1

11

111

11

11

11

5

1

1

1

1

1

342

11

11

11

111

11

11

332

111

11

111

214

1

1

1

1

11

1

17813

11

11

11111

11

111

11

11

11

253

11

1

1

1

111

1

1

31838154

11

1

1

11

111

11

1

1

11

111

1

1

11

1

11

1

1

1

11

11

111

1

1

1

1

11

11

11

1

111

11

1

1111

1

1

11

1

11

111

111

1

1

11

111

1

1

331

111

11

11

2774

1

11

111

11

1111

111

11

11

1

1

1

1

1

1

1

32

11

1

11

1

1

12

11

1

1

1

331

11

111

11

22

11

11

11

11

11

11

1

1

1

1

610

2

1

1

67

11

11

11

11

11

11

1

1

1

2811

11

11

22

11

11

1

1

2

1

1

11

11

11

11

1

1

12

1

11

11

11

1

1

1

1

176

142

1

11

1

111

1

1

22

11

11

11

11

3438113

44

11

1

11

1

1

1

111

111

233072

11

1

1

1

1

1

1

1

1

1

1

11

1

1

1

11

11

1

1111

1

1

11

111

1

1

1

11

1

1

111

1

13

11

1

1

1111

1111

231131339108

1

1

333

111

111

111

11

11

1692107985

11111

111

111

111

111

111

1111

111

1111

11111

111

1111

111

111

11

111

11

11

1

11

11

11

111

1111

111

111

111

11

111

11

111

11

111

1

11

111

1111

11

111

11

111

11

1111

111

111

11

111

111

1111

111

1111

11

11

111

111

111

111

111

111

111

11

1111

11

1111

11

111

11

111

1111

111

11

11

1111

11

111

111

11

111

11111

222

11

111

11

111

11

11

111

11111

1111

11

111

1111

111

11111

11

11

111

11

111

111

11

11

111

11

11

1111

111

342

1

11

1

1

1

11

1

111

111

3151615

111

111

1111

111

111

111

111

11

1111

111

11

1111

111

111

111

111

1111

310126

11

11

1

1

663

11

11

111

1

11

111

11

22

1

1

11

351

1

1

111

11

11

451

451

11

11

1

1

11

11

1

1

1111

11

11

11111

111

111

11

11

11

11

11

11

1

1

1

1

1

11

11

11

11

1

1

1

46537

11111

1213

112

111

1

1

111

12212

11111

11111

11111

11111

111

111

111

111

11111

34411

34411

37

26

3

1

1

1

11

11

1

1

11

11

1

1

1

1

11

11

11

1

1

1

323

323

1

1

213

11

1

111

1

1

11

11

11

11

11

11

111111

111111

2213510081114222931121130

31

1

1

1

1

21

1

11

1

1

1

342

342

342

12

11

1

111

111

2222

1

1

1

1

1222

1222

122

1

111

1

2

1

1

3876

2765

1111

221

221

111

11

222

111

111

111

1111

1

1

1

1111

221289921095222921107130

453

453

453

111

11

11

111

11

312

11

22

22

1

11

1

62544303

111

62342283

111

111

11

11

111

111

112187

11

11

111

1

1111

111

111

11

11

11

1

11

1

11

11

11

11

11

21232

11

11111

11

1

111

111

11

11

133

11

11

111

111

111

1

1

1

11

11

11

11

1

1

111

111

1123

1

11

1111

11

11

11541

1111

1

111

11

11

111

22

22

22

11

11

6724

6724

221

11

111

1

1

4423

11

1111

111

1111

117684410695

125232201

118152161

11

1111

11

1

11

1

11

1

11

1

11

1

11

11

1

1

1

11

1

1

11

1

11

1

1

11

1111

11

1

11

11

1

23

1

11

11

11

11

111

111

111

111

111

111

4171714174

4161614164

111

111111

111

111

1111111

111

111

111

111

111

111111

111111

111

111

111

111

111

531411528

3313

111

111

1111

11

1133

1111

11

11

27737

11111

11111

111

111

1111

111

111

1

1

12

11

1

1

1

576

111

111

11

11

1

11

111

11

111

111

11

222

111

111

1111

1111

111

111

332

111

11

111

24

11

1

11

1

111

111

112111

1

111111

12313

12313

11111

111

11

11

7910

7910

22

11

11

1

1

777

111

111

111

111

111

111

111

11

11

11

71312113

71312113

111

111

3444

1111

1111

111

1111

48718

1111

111

111

111

1111

111

11111

111

1111

1111

1111

1111

10831025141154

111

111

65463111664

5727

1111

111

11

1111

111

111

11

222

111

111

5262617264

1111111

111

11111

1111

111

111

111

111

111111

111

111

111

111

111

11111

111

11111

111

111

111

111

111

111

111

111111

111

111

111

1

1

11111

11111

222

111

111

2314

11

1111

11

11

777

111

111

111

111

111

111

111

133

111

11

11

1

1

555

111

111

111

111

111

244

111

111

11

11

22

11

11

11

11

11

111

312152120

1

1

1324

111

1

1111

11

1111

1111

1671110

1

1111

1111

111

1

111

111

111

1

111

111

111

111

111

1

1

111

111

11

11

114212227

111

111

11

11

1

1

112

1

111

112192122

11

1

1

111

1

11

1

11

11

111

11

1

1111

11

1

11111

1

111

11

11

11

11

1

111

111

1

11

11

188

188

188

11

11

111

11

11

11

11

11

228727211134914

228727211134914

11

11

331

11

11

111

227676511134714

1111

111

111

1111

1111

1111

11

11111

11

11

111

11

11

11

111

1

111

111

11

111

11111

1

111

111

1111

11

111

11111111

111

11

11

11

11

111

11

111

1111

1111

111

111

11

1

111

111

111

111

1111111111

11

111

111

11

111

111

1111

1111

11111

111

111

111

111

11

1111

1

11

111

11

111111

11

111

11

11

1

1

111

111

1111

222

1

1

1

221

221

11

111

207288271151

197188271151

16518

111

1

111

111

1

11

111

11111

111111

111111

11

12212

11111

111

111

111

111111

111

111

111

111

111

111

111

111

111

111

111

15102

111

1

11

11

11

1

1

1

11

111

111

111

231015

11

111

1

11

1111

11

1

1

11

1

11

11

11

1

111

1

726281236

111

111

111

111

111

111

11

111

11111

111

111

111

111

111

111

111

111

111

11

1

11

111

1111

1

111

111

111

111

1

111

11

1

11111

11111

1

11

111

111

114

1

1

1

111

111

111

11

11

2

1

1

111

111

1

1

1

1

117

11

1

11

1

1

1

1

111

111

22212

1111

11111

36

1

1

1

1

11

1

11

111

111

1

1

111

111

222

111

111

144114

111111

111

111

111

11

254

254

1

32

1

11

1

1

122

1

11

11

953181173

111

111

111

111

23117

1614

11

11

1

1

1

1

1

1

1

1

1

1

1

1

11

1

1

1

1

410

1

1

1

1

1

1

1

1

1

1

1

1

1

1

1

1

3

1

1

1

1

1

111

2

2

1

1

914123

914123

11

11

1

1

1

1

111

1

1

1

1

1

111

1

1

1

1

1

1

1

1

1

1

1

1

11

11

111

73

73

1

11

1

1

1

1

1

1

1

1710127

94121

1

11

1

111

11

1

11

1

1

1

11

1

1

1

11

1

1

1

1

111

1

1

1

111

1

1

765

1

11

111

1

1

1

1

111

11

1

11

1

1

5113110281212

5113110281212

111

111

444

111

111

111

111

1111

1111

555

111

111

111

111

111

333

111

111

111

1

1

444

111

111

111

111

26664371

1

111

111

111

111

111

111

1

111

111

11111

111

111

111

111

1111

111

11

111

111

111

111

111

111

1

111

111

111

111

111

111

1

111

1

111

111

111

1

111

111

111

111

111

111

111

111

111

111

111

111

111

1

111

111

111

111

11

111

111

111

111

111

111

1111

111

11

111

111

111

111

111

111

111

1

1

333

111

111

111

12

1

11

111

111

31515215

111

111

111

1111

1111

111

111

111

11111

1111

111

111

111

111

111

111111

111111

111

111

111

111

111

111

111

11

11

111111

111111

111

111

11

11

11

11

262242538172736

262242538172736

252122388152576

111

222

1111

111

11

111

1111111

111

111

111

111

111

111

111

11

11

111

11

111

11

111

111111

111

1111

111

11

11

111

111

1111

111

111

111

111

11111

111

111

111

111

11

111

111

111

111

111

11

11

111

11

111

111

111

111

111

111

11

111

111

11111

1

11

111

111

111

111

111

111

11

111

111

11

111

111111

111

1111

111

111

111

111

111

111

11

1111

111

111

111

11

111

111

11

111

111

111

11

111

111

111

111

111

1111

111

1

111

111

111

1111

111

111

11

111

111

111

111

111

11

11

111

111

111

111

111

111

111

111

11

111

111

111

111

111

111

11

111

111

11111

111

111

111

111

111

11111

111

11111

1111

111

111

1

111

111

111

11

111

11

111

111

111

111

11111

111

111

111

111

1

111

111

11

111

11111

11

111

111

111

11

1

111

1

11111

111

11

11

1111

111

1111

1

111

111

111

111111

111

111

1

111

11

1

111

111

111

1111111

111

11

111111

111

111

111

111

111

1111

111

111

111

111

111

111

111

111

111

1

111

111

111

111

11

111

11

111

1

111

111

111

111

111

111

111

111

111

111

11

111

1

111

111

111

111

111

11

11111

111

11

111

111

111

111

111

111

111

11

1

11

1

8819

111

111

1111

111

11

111

111

11

111

11111

11111

111

22

11

11

233

111

111

11

24231270192145

291414

11

11

1222

1111

111

171111

11

11

111

11

111

111

111

111

111

1111

11

421

211

111

1

21

11

1

12212

11111

111

11125

111

111

1

12

1

11

111

111

1

1

111

111

11

11

11

11

11

11

341

11

111

11

1

1333

222

111

111

1111

1111

442

11

11

111

111

221

111

11

445

111

111

334

1

11

11

11

1

11

1

1

1

12211

12211

11111

11

220234241

11221

11111

11

11718419

111

111

1111

111

111

11

1111

111

11111

111

111

111

11

111

11

111

111

111

1111

111

111

11

11

111

111

12118133101052

1215212

1111

111

111

11

111

11

11

1

11

11

1

11

1

111

1111

111

111

11

11

155251

1111

111

11111

111

1111

12

11

1

111

111

735432451

111

11

1111

11

11

1111

111

11

111

111

1

1

111

1111

111

111

11

1111

11

111

111

1

111

1

1

111

11

111

11

111

111

111

1

111

111

1111

111

11

111

11

111

11111

11

1111

1111

111

11

111

111

1

222

111

111

2211

11

1111

1

1

1

1

781

11

11

11

111

11

11

1

11

11

11

11

11

22

11

11

23

11

1

11

111

111

1

1

1211

111

11

111

111

111

111

221

11

111

111

11

11

11

11

221

1

1

11

1

1

1

111

111

123

1

11

111

442

111

111

11

11

111

111

16614

111

11

11

111

111

11111

1111

1111

11

11

222

111

111

1111

1111

333

111

111

111

111

111

111

111

111

111

457

111

111

1

1

11

111

111

22

11

11

11

11

11

11

1

1

11

11

2

1

1

16214

11

11

11

11

11

11

11

11

11

11

211

11

1

1

111

12

12

1

11

333

333

111

111

111

711113

2212

111

1111

111

111

122

111

11

111

111

133

11

11

111

23

11

11

1

11

11

1271

16

1

11

1

1

1

1

1111

1111

335481211

451

11

11

11

11

11

319221111

11

11

111

111

111

11

111111

11

111

111

11

11

1

111

11

1

1

1111

1

111

11

111

1

11

1

1

124

1

1

111

11

11

11

56

11

11

1

11

11

11

2

1

1

121

11

11

23

11

1

11

1

1

1

1

14

1

1

11

1

12

11

1

11

11

111

111

23313

12112

11

11

11

11

11

11

1

1

1

11

1

1

111

111

111

113

11

12

12

11

11

1

1

1

111

111

93340233

91412

33

33

33

11

11

11

9119

111

111

111

111

111

111

222

111

111

111

11

11

11

564

11

11

454

111

111

1

111

111

1242

131

1

121

11

11

11

11

1111

131

1

121

1

111

111

111

11111

11111

1112

1

1

1111

222

222

111

111

111

111

111

111

3775

2664

1111

1553

1

1

111

111

1

1

1

1

11

11

11

11

1111

1111

2322

1

1

1

1

1

2221

111

111

1

1

1

544

111

11

11

11

11

111

212

212

111

11

11

13283215635

111

111

111

111

111

5232315523

1333

1222

111

111

1111

111

111

4191915519

4181815518

111

111

111111

111111

1661336

111111

111111

111

11111

111

111

1222

1111

111

222

111

111

111

111

111

133113

111

111111

111

111

111

111

2125

1

1

1

1

112

112

11

11

11

11

1111

1

1

31414

2313

2313

111

111

111

111

1111

1111

1111

1

1

1

1

8144583752911

693246344171

24311124

1111

23210123

1

1

11

11

222

111

111

111

111

211

11

11

821

11

11

11

1

1

1

1

1

111

111

1

1

1111

111

111

111

111

11

11

111

111

1111

1111

11

21

21

1

1

11

11

322111

322111

11111

11111

111

111

11

11

39273320411

131211514

111

111

111

111

3232

1111

11

1111

22221

11111

1111

1111

1111

222

111

111

22111

1111

111

1111

1111

2513201425

332

111

111

11

11

11

1

33

11

11

11

212

11

111

424223

1

1111

111

1111

11111

111

111

3223

11

1111

1111

2221

1111

111

121

11

11

1

1

11

11

355

111

111

11

111

11

111

111111

111111

111111

11111121111

1111

55515

1111

44414

1111

1111

11111

11111

1111

1111

1111

1111

444241

33313

2222

1111

1111

11111

11111

111111

111111

111111

11111

38

11

27

27

11

16

1

1

11

4

1

1

1

1

12

1

2

2

2

1

1

1

1

1

1

10283511437

111111

12

11

1

3111616

11

1476

1476

11

11

111

111

111

11

11

111

111

111

111

11

11

2789

11

2688

1111

1111

111

111

111

22

11

11

1111

1111

111

111

111

111

1222

1111

1111

111

513141315

111

512131314

1

1

1

22

22

22

11

47729

1

1

11111

11111

111

111

1111

1111

111

111

111

11

11

22112

111

1111

1

133113

111

122112

111111

111

111

111

111

111

111

31375711191312572850

4

4

4

4

1

1

1

1

356917215586

55768

43545

4

1

1

1

1

1

1

1

1

1

1

11

11

3

1

1

1

2

1

1

1

1

111

111

111

111

111

1

1

1

1

1

1

1

1

21223

1

1

11

1

1

1

1

11

1

1

1

1

1

1

1

11

1

1

1

111

1

1

1

1

1

111

11122

2

1

1

11

11

2

1

1

1

1

1

1

2

1

1

1

1

1

1

17

1

1

1

1

11

1

1

2

1

1

1

1

11

11

39

8

1

1

2

1

1

1

1

1

1

1

1

1

1

1

5

3

1

1

1

1

1

1

1

2

1

1

1

1

1

3

1

1

1

1

1

1

16

1

1

11

1

1

1

1

1

1

1

1

1

1

1

1

3

1

1

1

2

2

1

1

2

1

1

1

11

11

11

11

11

11

111

276416314448

1

1

112

1

111

12

11

1

2

1

1

1

1

422102185

1

1

1122

11

1111

1115

1

1

1

1

1

1

1

1

1

11

1

1

1

1

11

1823

11

1

1

11

11

1

1

11

1

11

1

1

1

111

11

1

1

1

1

11

1

1

1

11

11

1

1

11

1

1

1

1

1

1

1

1

1

1

1

115560

11

111

111

11

111

111

11

11

11

11

1

11

111

11

11

111

11

11

111

11

1

11

11

11

11

11

11

11

11

11

1

11

11

111

11

11

11

11

11

11

11

11

1

11

11

111

11

111

11

11

1

111

11

11

11

11

11

11

11

11

11

11

1

1

428

11

1

1

1

1

1

1

1

1

1

1

1

1

1

1

11

1

1

1

11

1

11

1

1

1

1

1

1

11

11

111

1

1

345

11

111

111

11

11

11

11

1

1

62427

11

111

111

1

111

11

1

11

1

11

11

11

11

11

111

11

11

11

11

11

11

11

11

111

11

11

111

3

1

1

1

1

1

6

1

1

1

1

1

2

1

1

1

1

38

11

11

1

1

1

1

1

11

111

111

1111

2

1

1

1

1

431471275

133

111

11

11

2021227

111

111

111

11

11

111

1

1

111

1111

111

11

1111

111

11

11

1

111

111

1

111

1

111

111

11

1

111

1

1

111

2

1

1

123

1

11

111

1215

1

1111

1

11

1

55

11

11

11

11

11

2

1

1

1

1

3

1

1

1

111

111

171112

111

111

11

111

111

1111

1

11

111

11

111

11

1111

3

1

1

1

11

11

7

1

1

1

1

1

1

1

115

1

1

1

111

1

845181

1

1

2

1

1

2

1

1

7

1

1

1

1

1

1

1

1

1

13

1

1

1

1

1

1

1

1

1

1

1

1

1

1

1

1

1

111

1

1

1

1

1

1

1

1

1

1

114

1

1

111

1

3

1

1

1

2

1

1

3

1

1

1

11

11

2

1

1

57

11

11

1

11

1

11

11

1

1

11

11

1

1

226

1

1

1

1

111

111

3

1

1

1

1

1

1

1

1

1

1

1

15

11

1

1

1

1

13

1

1

11

1

1

1

1

11

11

1313

11

1111

11

1

1

562

1

1

1

1

8

1

1

1

1

1

1

1

1

11

5

1

1

1

1

1

1

1

1

1

1

1

318

1

1

1

1

11

1

1

1

11

1

1

1

1

1

1

1

1

11

4

1

1

1

1

4

1

1

1

1

1

1

2

1

1

3

1

1

1

4

1

1

1

1

1

1

1

1

11

11

2

1

1

2

1

1

1

1

2

1

1

1

1

1

1

1111

1111

12

1

11

1127

3

2

1

1

1

1

1

14

1

1

3

1

1

1

6

1

1

1

1

1

1

4

1

1

1

1

1

1

1

118

1

1

1

1

2

1

1

11

11

11

1

1

1

1

1

1

31218

11

30217

1

10

9

1

1

1

1

1

1

1

1

1

1

22

22

1

1

11

3

3

1

1

1

3

3

1

1

1

14212

1

13212

1

1

1

1

1

1

1

11

1

11

11

1

1

1

1

3

1

1

2

2

1

1

1

1210

1

1

112

11

11

11

1

3

3

2

1

1

1

1

13

12

11

1

1

1

1

173451

1

1

1

311

11

27

11

1

1

2

1

1

1

1

11

1

1

1

1

1

2

1

1

1

4

4

1

1

1

1

1

1

1

31328

13

8

1

1

1

1

1

1

1

1

1

1

3

1

1

1

1

1

1

1

1

1

11

11

1

1

1

4

1

3

1

1

1

11

11

1111

25

2

1

1

1

1

22

11

11

1111

413

413

22

1

1

11

11

11

11

1

1

6

1

5

3

1

1

1

1

1

1

1

2

2

1

1

1

1

1

1

130324503114151398

434563311129

201932

111

111

111

444

111

111

111

111

214

111

1

1

11

111

111

12

11

1

1

1

1

1

1

1

1

1

11

11

1

1

11912

111

1

111

111

222

111

111

11

11

111

111

1

1

1

41437331196

2114

1

11

1

1

1

1

11

1

1

1

1

11

1

1

323

111

11

111

1

1

3

1

1

1

11

11

5119

1

111

11

1

11

1

11

11

11

1343320

11

1

11

11

1

1

1

1

1111

1

1

1

1

1

1

1

1

11111

1

11111

1

1

13273527

11

111

11

11

11111

11

11

11

11

11

11

11

11

11

11

111

11

11

11

111

1111

11

111

11

11

111

1111

1111

6

1

1

1

1

1

1

1

1

9

1

1

1

1

1

1

1

1

1

4

4

1

1

1

1

1

1

1

11110

1114

12

1

11

1

111

111

6

1

1

1

1

1

1

1

2

1

1

20305621138

71826128

1

1

1111

11

11

1233

111

111

111

111

111

11

11

111

111

1111

1111

291213

111

11

1

1

111

11

111

111

1111

111

111

111

11

111

1233

111

1111

11

11

11

1111

1111

1

1

22815

11

11

11

22

11

11

224

11

11

11

11

2

1

1

225

1

1

111

1

111

1

1

236

8

1

1

1

1

1

1

1

1

11

18

1

1

1

1

1

1

1

1

1

1

1

1

1

1

1

1

1

1

19

1

1

1

1

1

1

1

1

11

57926

111

56825

1

11

1

1111

1

111

11

1

1

1

1

1

1

1

1

1

1

1

111

11

1

11

11

1

11

1

11

1

11

11

1

1

1

1

111

111

1

1

1

2

2

1

1

1

1

3311227

22

11

11

339225

1111

11

1

1

111

1

1

1

11

1

111

1

1

1

11

1111

11

1

1

11

1

11

1

1

11

111

111

2364931126

2364931126

1

1

113

111

1

1

2

1

1

1

1444

111

1111

111

111

174561181

111

11

11

1

11

1111

1111

1

111

1111

1

111

111

111

111

111

1

1

111

11

111

1

111

11

11

111

11

111

111

11

1

111

1

11

11

1111

1

111

111

111

11

11

111

1111

11

11

1111

111

111

11

1111

1

1111

111

11

11

111

111

1

111

11

111

1111

111

111

11

1

111

111

11

111

11

1

111

111

1111

111

1

111

111

1111

4122431

111

111

111

11

11

1

11

111

11

11

111

1

111

111

11

11

11

2222

1

1

111

1

111

111

11

11

111

1

1

111

1233

11

111

1111

1253228

1053226

1

1

22

11

11

10

1

1

1

1

1

1

1

1

1

1

1

1

1

1023206

1

1

1

1

1

1

1

1

1

1

1

1

1

1

1

1

1

1

1

1

1

1

1

1

1

1

11

1

1

1

1

1

1

1

1

1

1

111

11

1

1

1

1

1

1

1

1

1

1

1

1

1

1

1

1

1

1

1

1

1

1

1

1

1

1

1

11

1

1

1

1

1

1

1

1

1

1

1

11

1

1

1

1

1

1

1

1

1

1

1

1

1

1

1

1

1

1

1

1

1

11

1

1

1

1

1

1

1

1

1

1

1

1

1

1

1

1

1

1

1

1

1

11

1

1

1

1

1

1

1

1

1

11

1

1

1

1

1

1

1

1

1

1

1

1

1

1

1

1

1

1

1

1

1

1

1

1

1

1

11

11

1

1

1

1

1

1

1

1

1

1

1

1

11

1

1

1

11

1

1

1

1

1

1

1

1

1

1

1

1

1

1

1

1

1

1

11

1

1

1

1

1

1

1

1

1

1

2

1

1

12

11

1

1

1

1

2

2

1

1

1

1

1

561625

1

561624

541514

11

11

11

111

111

111

111

11

1111

1

11

111

11

111

111

111

111

2

1

1

13

1

1

11

4

1

1

1

1

1

1

2119

1

1

1

1115

12

1

11

1

1

1

1

1

1

1

1

4

1

1

1

1

1

1

1

1

1

1

11

1

1

12

1

11

11

81923126

122

122

111

11

11

1

1

1

1

1

11

232

121

1

111

111

111

44583

2

1

1

114

1

111

1

1

1

1

2

1

1

1

1

1

1

4164

1

1

1

1

1

1

1

1

1

1

1

1

1

1

1

1

1

1

11

1

1

111

1

1

1

1

1

1

1

1

1

1

1

1

1

1

1

1

1

1

1

1

1

1

11

1

1

1

1

1

1

1

1

1

1

1

1

1

1

11

1

1

1

1

111

111

1

1

1

36

11

1

1

1

11

11

1

1

1

1

1

1

1

1

2101025

4

1

1

1

1

1

1

1111

1

1

1

1

1

1

1

1

2

1

1

5

1

1

1

1

1

3

1

1

1

5

1

1

1

1

1

99

11

11

11

11

11

11

11

11

11

1

1

13

12

11

1

1

1

1

1

1

5

1

1

1

3

1

1

1

111

111

111

12

12

11

1

1

1

2

1

1

227

227

1

1

1

1

224

111

1

1

111

1

1

2

1

1

179226

111

1118

1118

1

1

1

1

1111

1

1

1

127

111

2

1

1

12

1

11

2

1

1

111

111

2

1

1

1

1

4417

11

11

11

11

11

11

111

111

1

1

111

111

111

111

111

111

1111

51634

43

43

1

1

11

11

1

11211

1

1

1

1

11

11

113

1

1

111

14

1

1

1

11

418

1

23

11

1

11

1

1

2

1

1

1

1

2

1

1

5

1

1

1

1

1

22

11

11

1

1

1

1

1

22353

11

1

1

3

2

1

1

1

1

1

1

2

1

1

1

1

1

22

1

1

1

1

1

4

1

1

1

1

1

1

1

1

3

1

1

1

3

1

1

1

1

1

1

1

3

1

1

1

1

1

1

1

22221

1

1

1

1

1

1

2

1

1

2

1

1

1

1

1225

111

1

1

1111

1

2

1

1

1

1

1

1

1

1

1

1

11

11

1

1

1

1

5

5

1

4

1

1

1

1

15

14

2

1

1

12

11

1

1

1

1

328

323

2

1

1

321

1

1

1

11

1

1

1

1

1

1

1

1

1

1

1

1

1

1

1

1

1

1

1

1

4

2

1

1

1

1

1

1

1

1

1

41492

41492

1

1

11

11

1

1

3157

1

1

1

1

1

1

1

1

1

1

1

1

1

1

1

1

1

1

1

1

1

1

1

1

1

1

1

1

111

1

1

1

1

1

1

1

1

1

1

1

1

1

11

1

11

1

1

1

1

1

1

1

1

1

1

1

1

13

1

1

1

1

1

1

1

1

1

1

1

1

1

1

1

11

11

111

114

1

1

1

1

11

1

1

1

1

1

1

1

1

1

2

1

1

1

1

12133755

12133655

1

1

11

11

113

11

11

1

11

11

11

11

11

11

235

1

11

111

1

111

2688

111

1111

111

111

111

1111

11

11

11

11

11

11

1

1

1357

1111

111

11

1

11

111

1

111

111

1111

11

11

22

11

11

111

111

111

111

1

1

11

11

1

1

1

1

1

1

1

1

11

2410

1

1

11

111

1

11

11

11

1

1

111

111

1

11

11

11

11

1

1

261331886273

115

1

1

111

111

1

1

1

1

1

1

1922128

113

1

1

111

111313

111

111

111

111

111

111

11

111

111

111

111

111

11

11

11

4

1

1

1

1

6616

111

111

111

111

111

1111

111

111

242933

122

11

111

111214

11

111

11

11

111

11

11

11

1

1

11

11

11

11

12

11

1

11

1144

111

11

111

11

11

11

188

11

11

11

111

11

11

11

11

1

1

2043144

111

111

233

111

111

11

222

111

111

1111

1111

62121

111

11

11

11

11

111

11

11

111

11

11

11

111

11

11

111

11

11

11

11

111

133

111

11

11

71213

11

111

111

11

111

1

111

11

111

111

111

11

11

22

11

11

11

11

111

2347

11

11

223

11

111

11

11

11

122

111

11

213142

9

1

1

1

1

1

1

1

1

1

1119

1

1

1

1

1

1

1

11

11

1

1

1

1

1

1

1

1

1

1

15

1

1

1

11

1

117

1

1

1

111

1

1

1

1

1

111

111

2082833113

4111212

1111

1111

111

111

111

111

1111

111

111

1111

11

111

11315115

111

111

111

111

111

111

11111

111

111

11

111

111

111

111

11

111

111

191515

11

11

111

111

1111

111

111

111

111

111

11

111

11

11

11

11

11

333

111

111

111

22

11

11

55

11

11

11

11

11

111

111

11

11

22

11

11

111

111

1

1

11

11

33415

1111

11111

1

111

111

111

111

122

111

11

1111

111

111

11

11

1111

1111

111

111

12313

111

11111

11

11

11

444

111

111

111

111

729331

11

11

111

11

1111

111

11

111

11

11

1

11

11

11

111

11

222

22

1111

11

11

11

11

11

1

11

11

11

11

1111

973584361837784

116

5

1

1

2

1

1

1

1

1

1

111

1

1

11

11

12

11

1

1

4517

4517

1

1

111

112

11

11

2313

1111

11

111

2131212329

2121112328

222112

1

11

11

11

1

1

11

11

1

11

1

11

55117

111

111

111

1

11111

111

1

22112

11111

111

111

111

11

1115

11

1

1

11

1

1

111

1

1

1

1

9119100518185

558

222

1

11

111

1

1

11

11

11

3

1

1

1

1

1

111

111

5312862

112

111

1

333

111

11

1

111

13

11

1

1

1

1

227

1

1

1

11

1

1

11

11

1

1

214

1

11

1

1

11

113

1

1

111

11

11

1

1

13

1

11

1

26814

11

1

11

1

111

111

11

1

11

11

11

1

111

1

1111

223

1

111

111

554

1

111

11

11

11

1

111

1111

1111

11

12

11

1

445

111

111

111

1

111

13

1

11

1

48265518113

76113

1

1111

11

1

11

11

11

11

11

1

111

1

11

1

1

1556

1111

111

1

1

111

111

11

2212

111

1111

11

11

1

1

111

111

1

1

111

111

111

2114

111

11

11

1

11

11

114101215

111

1

11

1111

11111

11

1

11

1

1

111

1

1

111

111

11

1

111

11

11

2463641366

11

1111

1

1111

1

1

1

1

11111

1

11

11

1111

111

1

11

1111

111

111

111

1111

11

11

11

1

1111

1111

2

111

1

11

11

111

11111

111

11

1

11

1

111

111

11

111

1111

1

11

11

111

111

111

111

111

1

1

1

111

1

1

111

11111

1

1

11

1

1111

1

111

11

1

11111

1

111

11

11

11

111

2

2

1

1

1

1

331301511149

96581168

1

1

112

111

1

113

111

1

1

1

1

1

1

1

1

22

11

11

1

1

556

1

1

111

11

111

1

11

111

11

11

11

1

1

1

1

1

1

1

1

1

1

1

22

11

11

111

111

1

1

1

1

11

11

1

1

552

11

11

11

111

111

121

1

1

1

1

22

11

11

784

11

1

111

111

111

111

11

11

222

111

111

11

11

1

1

22

11

11

111

111

2

1

1

2

1

1

2222

1111

1111

11

1

1

1

1

11

11

2

1

1

3

1

1

1

11

11

495

111

1

111

1

1

1

111

1

111

1

112

1

111

11

11

11

11

1111

1111

1442

111

1111

11

11

1233

1

11

11

11

11

111

111

11

11

1

1

11

1

1

67

11

11

11

11

11

1

11

1221

11

1111

1111

1111

11

11

111

111

111

24646980

111

111

221

11

111

111

111

4431

111

111

111

111

2

1

1

111

111

432

11

11

111

11

11

11

445

111

111

111

1

111

1

1

11

11

11

11

1

1

31

11

1

1

111

111

1

1

11

11

1

1

1111

1111

1111

1

1

1

1

111

111

1

1

111

111

1

1

3

1

1

1

1111

1111

1

1

11

11

11

11

2

1

1

11

11

6316

1

1

1

111

1

1

2

1

1

11

1

11

1

1

1

1

1

11

1

1

1

1

1

221

11

111

11

11

221

111

11

1

1

1774

111

1111

11

11

111

11

111

11

11

11

11

1

1

121

11

11

1

1

1

1

1

1

1

1

1332

1

1

11

11

111

11

11

1

1

1

1

11

11

11

11

11

11

11

11

1

1

22

1

1

11

11

11

1

1

1111

1111

11

11

1

1

11

11

111

1

11

1111

1111

111

111

111

111

1111

1111

1

1

11

11

111

111

1

1

426814112286

1

1

1

12

11

1

1

11

11

11

12

12

11

1

1

1

1

1111

1111

11

11

1

1

5

1

1

2

1

1

1

1

1

213

213

11

1

111

238117

1255

11

11

111

1111

11

1

1

2

1

1

1316

1111

1

11

1

1

11

13

1

1

11

14

12

11

1

1

1

1

1

8125

1

1

719

1

1

1

1

1

1

1

1

1

1

11

1

11

1

1

1

11

11

11

1

1

12

1

11

1

1

11

1

1

113

11

11

1

1

11

11

271443

11

11

11

11

1

1

1

1

3

1

1

1

1

1

1

1

1

1

122

111

11

11

117

1

1

1

1

111

1

1

11

11

4719

11

1

1

11

11

1

1

1

1

1

11

11

1

11

11

1

111

1

1

1

1

12

1

1

1

1

1

11

11

311

1

1

1

1

29

1

1

11

1

1

1

1

1

1

1

1

1

111

11

11

11

2

2

1

1

1736701185

1

1

11

11

11

11

111

123

11

11

11

2

1

1

121

111

1

1334

111

1

1111

111

11

11

1226501158

11111

1111

11

11

111

1111

111

111

11

11

1111

11

111

111

1

111

111

11

11

11

1

11

1

11111

11

1111

1111

11

1

1

11111

11

11

11

1

1111

11111

11

1

111

11

11111

111

111

111

1

11

111

11

1111

1

11

111

1

11

11

1111

11111

11

11

1

1

1123

11

111

11

1

1

1478

111

11

11

1

11

11

1

1111

111

1

1

1

171112

455

111

111

111

11

111

123

111

11

1

123

1

11

111

221

11

111

12319

119

11

1

1

1

1

1

1

1

1

1

1117

1

1

11

1

11

1

1

1

13

1

11

1

1

1

1

11

11

11

269

1

1

3

1

1

1

154

11

1

111

11

11

111

241718

241414

111

111

11

11

11

11

11

11

11

11

111

111

1111

11

1

1

11

11

22

11

11

1

1

1

4

4

1

1

1

1

1

1

1

1

1

1

1118

111

111

1

1

12

11

1

1

1

1

1

1

1

1

13

1

11

11

1

1

11111

11111

11111

11111

1111

13

1

3

2

1

1

1

1

1111

114

114

111

3

1

1

1

1

1

618211103

191275

1

1

1

1

1

1

11

11

1

1

7

1

1

1

1

1

1

1

1

1

1

1

11

8

1

1

1

1

1

1

1

1

111

111

11

11

23

1

11

11

11

11

2419

1

11

1

11

1

1

111

11

1

1

1

1

1

11

1

1

1

1

1

1210

1

1

111

1

1

1

1

11

1

1

11

1

1

1

1

1

1

17

1

1

1

1

1

1

11

1

1

1

1

1

1

1

1

111

111

11

11

2

1

1

1

1

1

1

1111

42314

1

1

1

1

1

1

1111

1

1

11

11

11

11

14

1

1

1

11

1

1

11

11

11

11

1

1

1

1

6519

2212

111

1111

22

11

11

217

11

1

1

11

11

1

1

1113

1111

2

2

1

1

15

1

14

14

14

1

11

1

1

1111

1745336603719579075

113

113

1

1

11

11

11

7415723018211371

7415622918211370

691151759210285

11

11

111

111

1

1

11

11

22

11

11

11

11

11

11

12

11

1

1

1

111111

3

1

1

1

1

1

111

111

33645611284

111

1

111

1

1111

11

1

111

111

111

1

1

1

1111

11

1

1

1

111

111

1

1

1

1

1

111

1111

111

111

111

11

1

111

1

111

11

11

111

111

1

1111

1

1

111

1111

111

1

1

11

1111

111

1

1

111

1

1

1

111

1

111

111

1111

111

111

1

1

1

111111

111

11

1111

111

1

111

1

1111

1

111

1

1111

11

1111

111

1

111

1

1

1111

1

1

1111

1

111

111

1

111

111

1

11

111

111

111

11

111

1111

111

13

1

1

11

11

11

11

11

35

11

1

11

1

1

1

333

111

111

111

111

111

11

11

111

111

211

11

11

1

1

258

11

11

11

11

1

1

111

11

11

11

12

1

1

1

112

1

111

11

11

1

1

2243

11

11

1111

111

111

111

2398

111

11

1

11

111

11

1

1

1

1111

11

122

111

11

1

1

2245

1111

11

111

11

11

111

111

1

1

2236

11

11

11

111

111

1

111

111

1

1

11

11

55102410

11

111111

11

1111

11

11111

11111

111111

11

11

2513

1

1111

111

11

1

227

1

11

1

1

1

1

11

1

1

1

1

14126

111

11

11

1

111

11

1

11

11

1

1

1

1

256

11

11

1

11

111

111

11

11

115121

1

1

1

1

1

11

1

1

1

1

11

1

11

11

1

1

111

1

1

1

111

1

1

1

1

33

11

11

11

1

1

11

11

1127

1

1

111

1

11

11

1

31518

11

111

11

11

11

11

11

1

11

111

11

111

11

11

1

1

11

11

1

1

1

1

355

11

1

1

111

111

111

1

1

1

1

3445

1

111

1111

1111

1111

1

1

3

1

1

1

11

1

1

11

1

1

1

1

1

1

12

11

1

1

1

111111

111111

2

1

1

11

11

1111

1111

12

1

11

122

111

11

11

11

114

1

1

111

1

1

1

1

1111

21

2

1

1

1

1

1

1

1

239519177

11

11

21

11

1

1

1

1

1

126406168

11

11

11

1

11

1

111

11

1

111

1

11

1

1

11

11

111

111

1

1

1

11

1

11

111

1

11

11

111

111

1

111

11

1

11

111

111

11

111

111

111

111

11

1

11

111

111

1

11

1

1

111

11

1111

11

1

111

11

11

1

1

11

111

11

111

11

11

111

111

14414

111

11111

111

111

111

111

111

111

55

1

11

11

11

11

1

111

111111

228110

228110

11

1111

1111

11516

11516

11

111

11

11

11

111

12

12

11

1

6626172

6626172

6626172

111

111

2303132

111

111

111

11

111

111

111

111

1111

111

1

111

111

111

111

111

111

111

111

111

111

1111

111

111

222

111

111

111

111

111

111

122

111

11

13

2

11

111

111

1111

1111

111

111

1111

1111

1111

111

111

111

111

222

111

111

12

11

1

333

111

111

111

112

1

111

111

111

111

111

444

111

111

111

111

111

111

111

111

11

11

12

1

11

111

111

1

1

111

111

111

111

222

111

111

111

111

111111

49129153119281475

1111

12837136

798

222

111

111

111

111

111

111

111

111

111

11

11

11

11

11

11

11617117

222

111

111

111

111

333

111

111

111

111

111

222

111

111

111

111

11212

11

11111

222

111

111

111

222

111

111

41010

22

11

11

44

11

11

11

11

444

111

111

111

111

111

47100115109281105

49102149

155114

111111

11

111

111

111

122112

111

111111

111

1111

1111

111111

111111

1

1

1

5596

1111

11

11

1221

111

111

2131

11

111

11

11

11

1111

111

111

111

1

1

1

11

11

11

3891310

11111

11111

25617

11

1111

11

11111

111

111

11

11111

11111

111

62024420

41620316

111

1111

1

111

111

1

1

11

111

111

11

1111

111

111

11

11

111

11

11111

11

11

111

11

111

1111

1111

111

111

11111

11111

111

111

111

11

11

11

216135

111111

111111

1524

111

11

111

1

111

111

111

111

2034345311343

35515

11111

111

1111

111

1111

11111

1628285310282

111

111

111

1111

1111

111

111

11111

111

111

11111

11111

111

111

111111

111

111

11111

111111

11111111

111

111111

111111

111111

1111

11111

1111

11111

48811181

48811181

1111

1111111

111

111

1111

11111

111

111

2101022101

111

11111

11111

111111

111111

66116

111

111

111

111

111

11111

111

111

111111

2589101149175

2589101149174

11

11

721241228

122112

111

111111

111

111

11

11

1

1

12313

111

111

1111

111

111

11

11

535

11

111

111

111

11

111

111

11

11

581111

111

1111

1111

111

11

1111

1111

111

111

111

11

419181227

11

11

11

11

111

111

111

1

1

11

11

111

111

1111

1111

21113

11

11111

1

1131014

11

111

111

11

11

111

111

111

11

111

11

111

111

1111

212

11

111

111

111

111

819334

512120

1

111

111

1

1

11

1

11

1

11

1

11

1

1

1

1

1

1

1

11

111

2

1

1

2

1

1

11

11

1111

1111

1

1

22

11

11

1214

1111

11

1

1

15

15

11

1

1

1

1

111

1444

1333

1111

111

111

111

111

1

1

1

122

122

11

111

5393412264

18816

111

1

11

1

111

1

111

111

1

11

111

11

1

111

1

111

171733

111

111

11

111

1

11

111

11

1

1

1

111

111

1

1

111

11

11

1

11

11

11

1

11

1

1

11

111

111

1

111

111

111

76117

11111

111

11

111

111

111

111

3621116

1

11

11

1111111

111

111

11

111

111

11

11

266

111

111

111

44

11

11

11

11

1

1591105546127

1591105546127

111

1111

1489103546125

111

111

11

11

111

111

1

1

112

11

11

6374611249

111

111

111

111

111

1111

111

111

111

111

111

111

11

111

11

111

1111

111

111

111111

111

111

11

111

1111

111

111

111

111

111

11

111

11

111

11

11

111

111

111

11

111

1

111

111

11

111

1

111

11111

31220328

11

111

11

11111

1

11

1

111

1

11

1

111

111

11

11

111

1111

222

11

11111

1

111

1

11

11

111

1

111

111

2

1

1

234

111

111

11

1

4302512331

111111

222

111

111111

1111

1

1

111

11

111

11

11111

111

111

111

11

11

111

1111

111

111

111

111

111

111

111

111

111

11

11

111

11111

11111

1222

111

1111

111
