# Supplementary material for: Simulated digestions of free oligosaccharides and mucin-type O-glycans reveal a potential role for Clostridium perfringens
Source: Sci Rep. 2024 Jan 18;14:1649. doi: 10.1038/s41598-023-51012-4 (PMC10796942; doi:10.1038/s41598-023-51012-4)
Supplement: Supplementary file 2 — Supplementary Information. [file 41598_2023_51012_MOESM2_ESM.zip › gutGH-SI/Krona/CAZy-EC-Krona-graphs/gut-EC.krona.html]

Javascript must be enabled to view this page.

magnitude
magnitudeUnassigned

EC\_3.2.1.111
EC\_3.2.1.140
EC\_3.2.1.18
EC\_3.2.1.22
EC\_3.2.1.23
EC\_3.2.1.49
EC\_3.2.1.50
EC\_3.2.1.51
EC\_3.2.1.52
EC\_3.2.1.63
EC\_3.2.1.97

3327313463112916

3327313463112916

11131315115114

1577352

1577352

111

111

111

11

11

11

1555342

11111

11111

444332

1

111111

11

111111

11111

186811262

186811262

1111

1111

1111

23311

12211

11

11111

111

111

141311131

141311131

111111111

11

11

111

11111

11111

11111

111

111

111

111

111

111

46727

46727

1122

1122

1122

1111

11

24424

111

111

111

23323

11111

11111

111

111

11111

11111

1111

1111

1111

1111

111111

111111

111111

111111

111111

111111

455335

455335

455335

455335

455335

1111

111111

111111

111

111111

2245512512

2245512512

2245512512

2245512512

2245512512

11111111

1111

1111

111

1111111111
