# Supplementary material for: Simulated digestions of free oligosaccharides and mucin-type O-glycans reveal a potential role for Clostridium perfringens
Source: Sci Rep. 2024 Jan 18;14:1649. doi: 10.1038/s41598-023-51012-4 (PMC10796942; doi:10.1038/s41598-023-51012-4)
Supplement: Supplementary file 2 — Supplementary Information. [file 41598_2023_51012_MOESM2_ESM.zip › gutGH-SI/Krona/CAZy-EC-Krona-graphs/nongut-EC.krona.html]

Javascript must be enabled to view this page.

magnitude
magnitudeUnassigned

EC\_3.2.1.18
EC\_3.2.1.22
EC\_3.2.1.23
EC\_3.2.1.49
EC\_3.2.1.50
EC\_3.2.1.51
EC\_3.2.1.52
EC\_3.2.1.97

111933329252

111933329252

251

1

1

1

1

1

241

131

1

1

1

121

121

111

1

11

11

11

11

357182

357182

11111

11111

11111

11111

2211

11

11

11

1111

1111

1111

21241

21241

21241

1111

1111

1

1

111

111

111

111

11

11

11

11

2211

2211

2211

2211

2211

1111

11

11

11

11

11

11

2

2

2

2

2

1

1

4281310

417139

11

11

11

11

11

11

11

11

13133

13133

11111

11111

222

111

111

22

1

1

1

21

1

1

11

11

11

11

11

11

1111

1111

1111

1111

111

111

111

111

111

3673144

22111

22111

22111

11

11

11111

11111

223322

223322

11111

11111

11111

112221

11

11

111111

111111

1

1

12211

12211

11

11

11

11111

11111

11111

1111

1111

1111

1111

1111

1111
