# Supplementary material for: Simulated digestions of free oligosaccharides and mucin-type O-glycans reveal a potential role for Clostridium perfringens
Source: Sci Rep. 2024 Jan 18;14:1649. doi: 10.1038/s41598-023-51012-4 (PMC10796942; doi:10.1038/s41598-023-51012-4)
Supplement: Supplementary file 2 — Supplementary Information. [file 41598_2023_51012_MOESM2_ESM.zip › gutGH-SI/Krona/CAZy-EC-Krona-graphs/all-EC.krona.html]

Javascript must be enabled to view this page.

magnitude
magnitudeUnassigned

EC\_3.2.1.111
EC\_3.2.1.140
EC\_3.2.1.18
EC\_3.2.1.22
EC\_3.2.1.23
EC\_3.2.1.49
EC\_3.2.1.50
EC\_3.2.1.51
EC\_3.2.1.52
EC\_3.2.1.63
EC\_3.2.1.97

3338506795205418

3338506795205418

2

2

2

2

2

1

1

111

111

111

111

111

111

11131520115124

15911362

131

121

121

111

1

1

1

1

1588352

111

111

111

22

11

11

11

11

1555342

11111

11111

444332

1

111111

11

111111

11111

186911262

186911262

23311

12211

11

11111

111

111

1111

1111

1111

11111

11111

11111

141411131

141411131

11

111111111

111

11

1

122111

111111

111111

111111

111111

111111

11

11

11

11

2271012131314

2271012131314

11111

11111

11111

11111

2211

11

11

11

1111

1111

1111

21241

21241

21241

1111

1

1

1111

2245512512

2245512512

2245512512

111

1111

11111111

1111111111

1111

111

111

111

111

11

11

11

11

1111

1111

1111

1111

1111

1111

2211

2211

2211

2211

2211

11

1111

881521317

111

111

111

111

111

871421316

11

11

11

11

1111

1111

1111

1111

24525

23323

11111

11111

11111

11111

111

111

122

111

111

11

11

11

11

11

11

2233

2233

2233

1111

1111

11

22

21

1

1

11

11

1

1

1

13133

13133

222

111

111

11111

11111

711126179

577346

577346

455335

455335

1111

111111

111111

111111

111

11111

11111

11111

11

11

11

223322

223322

11111

11111

11111

112221

1

1

111111

111111

11

11

22111

22111

22111

11

11

11111

11111
