# Supplementary material for: Simulated digestions of free oligosaccharides and mucin-type O-glycans reveal a potential role for Clostridium perfringens
Source: Sci Rep. 2024 Jan 18;14:1649. doi: 10.1038/s41598-023-51012-4 (PMC10796942; doi:10.1038/s41598-023-51012-4)
Supplement: Supplementary file 2 — Supplementary Information. [file 41598_2023_51012_MOESM2_ESM.zip › gutGH-SI/Krona/available-used_enzyme_profiles/uniprot-pval-spp.krona.html]

Javascript must be enabled to view this page.

magnitude
magnitudeUnassigned

pval-spp

448

177

127

1

1

1

1

1

1

1

1

1

1

1

1

1

1

1

1

1

1

1

1

1

1

1

1

1

1

1

1

1

1

1

1

1

1

1

1

1

1

1

1

1

1

1

1

1

1

1

1

1

1

1

1

1

1

1

1

1

1

1

1

1

1

1

1

1

1

1

1

1

1

1

1

1

1

1

1

1

1

1

1

1

1

1

1

1

1

1

1

1

1

1

1

1

1

1

1

1

1

1

1

1

1

1

1

1

1

1

1

1

1

1

1

1

1

1

1

1

1

1

1

1

1

1

1

1

1

1

1

1

49

1

1

1

1

1

1

1

1

1

1

1

1

1

1

1

1

1

1

1

1

1

1

1

1

1

1

1

1

1

1

1

1

1

1

1

1

1

1

1

1

1

1

1

1

1

1

1

1

1

5

1

1

4

1

1

1

1

11

4

1

1

1

1

7

1

1

1

1

1

1

1

1

1

1

21

1

1

2

1

1

4

1

1

1

1

3

1

1

1

11

1

1

1

1

1

1

1

1

1

1

1

2

2

1

1

4

4

1

1

1

1

74

57

1

1

1

1

1

1

1

1

1

1

1

1

1

1

1

1

1

1

1

1

1

1

1

1

1

1

1

1

1

1

1

1

1

1

1

1

1

1

1

1

1

1

1

1

1

1

1

1

1

1

1

1

1

1

1

1

1

1

1

16

1

1

1

1

1

1

1

1

1

1

1

1

1

1

1

1

1

1

1

1

1

1

2

1

1

1

1

42

30

1

1

1

1

1

1

1

1

1

1

1

1

1

1

1

1

1

1

1

1

1

1

1

1

1

1

1

1

1

1

4

1

1

1

1

8

1

1

1

1

1

1

1

1

21

14

1

1

1

1

1

1

1

1

1

1

1

1

1

1

2

1

1

3

1

1

1

2

1

1

1

1

1

1

1

1

2

1

1

1

1

1

1

1

8

8

1

1

1

1

1

1

1

1

61

53

1

1

1

1

1

1

1

1

1

1

1

1

1

1

1

1

1

1

1

1

1

1

1

1

1

1

1

1

1

1

1

1

1

1

1

1

1

1

1

1

1

1

1

1

1

1

1

1

1

1

1

1

1

8

1

1

1

1

1

1

1

1

5

4

1

1

1

1

1

1

7

7

1

1

1

1

1

1

1
