# Supplementary figures and images for: Simulated digestions of free oligosaccharides and mucin-type O-glycans reveal a potential role for Clostridium perfringens
Source: Sci Rep. 2024 Jan 18;14:1649. doi: 10.1038/s41598-023-51012-4 (PMC10796942; doi:10.1038/s41598-023-51012-4)

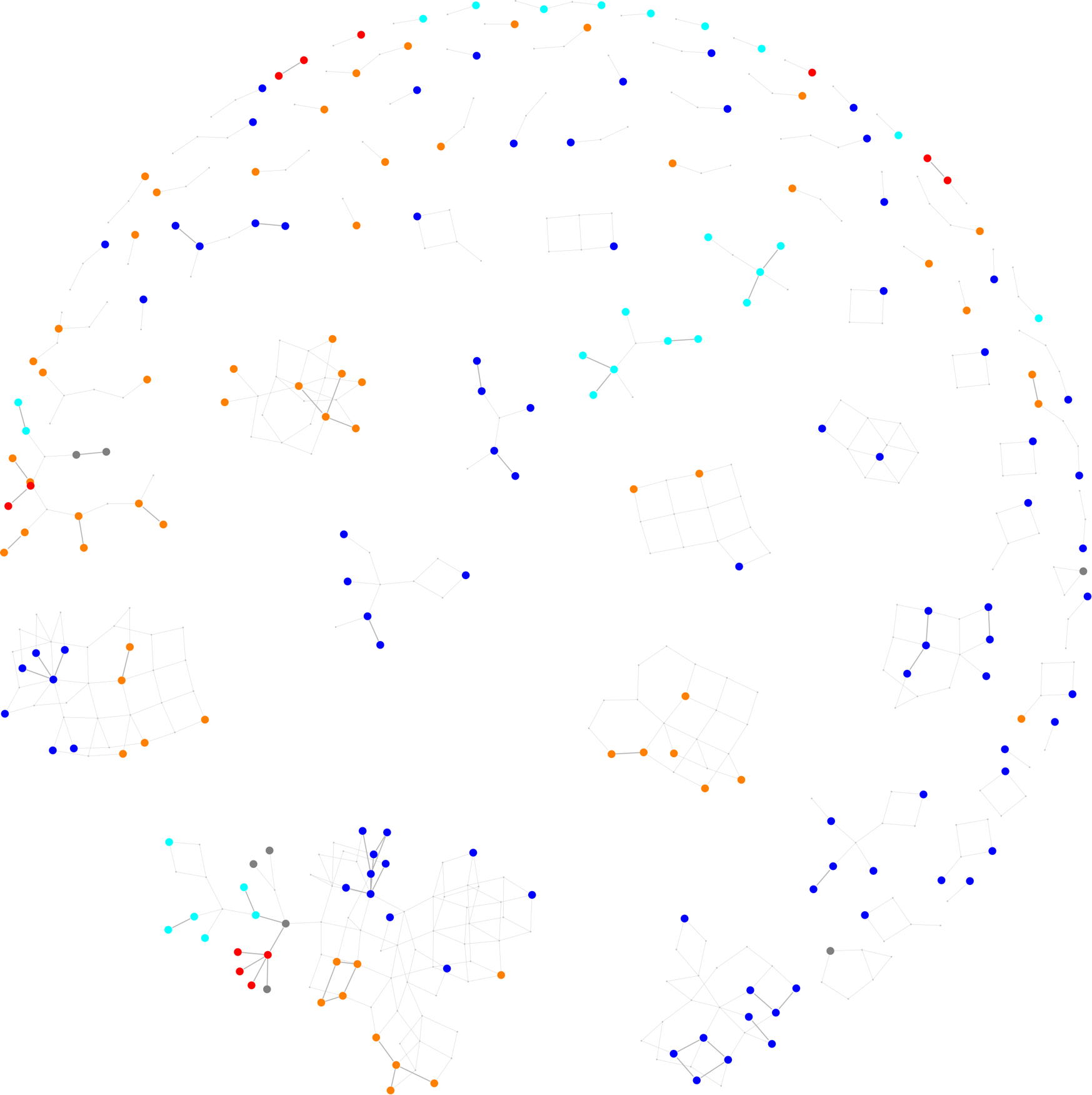

Supplement: Supplementary file 2 — Supplementary Information. [file 41598_2023_51012_MOESM2_ESM.zip › gutGH-SI/Networks/UniProt-HMO-networks-gut/p7986-GH-network-pp-hmo.jpg]

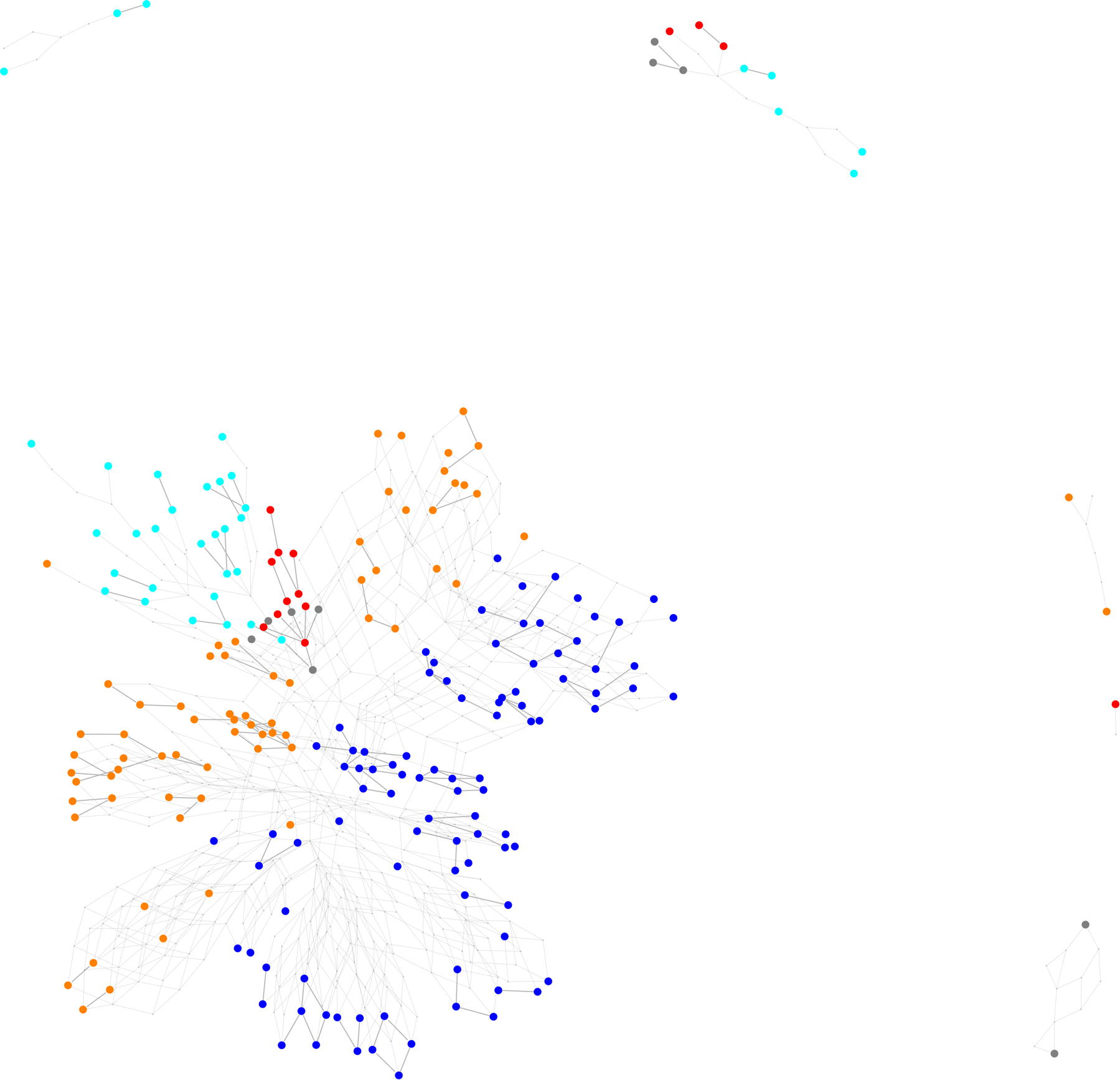

Supplement: Supplementary file 2 — Supplementary Information. [file 41598_2023_51012_MOESM2_ESM.zip › gutGH-SI/Networks/UniProt-HMO-networks-gut/p7954-GH-network-pp-hmo.jpg]

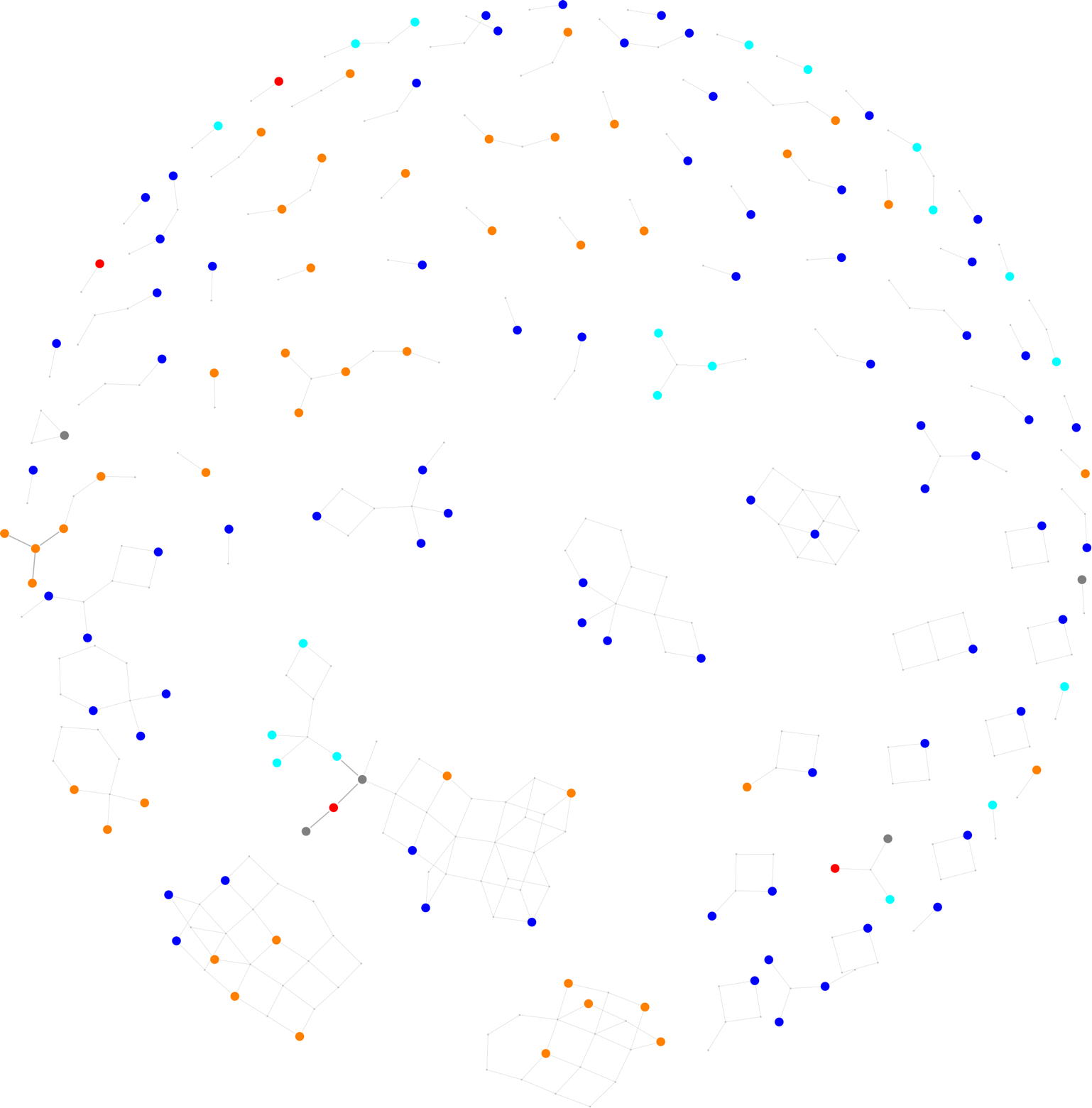

Supplement: Supplementary file 2 — Supplementary Information. [file 41598_2023_51012_MOESM2_ESM.zip › gutGH-SI/Networks/UniProt-HMO-networks-gut/p8123-GH-network-pp-hmo.jpg]

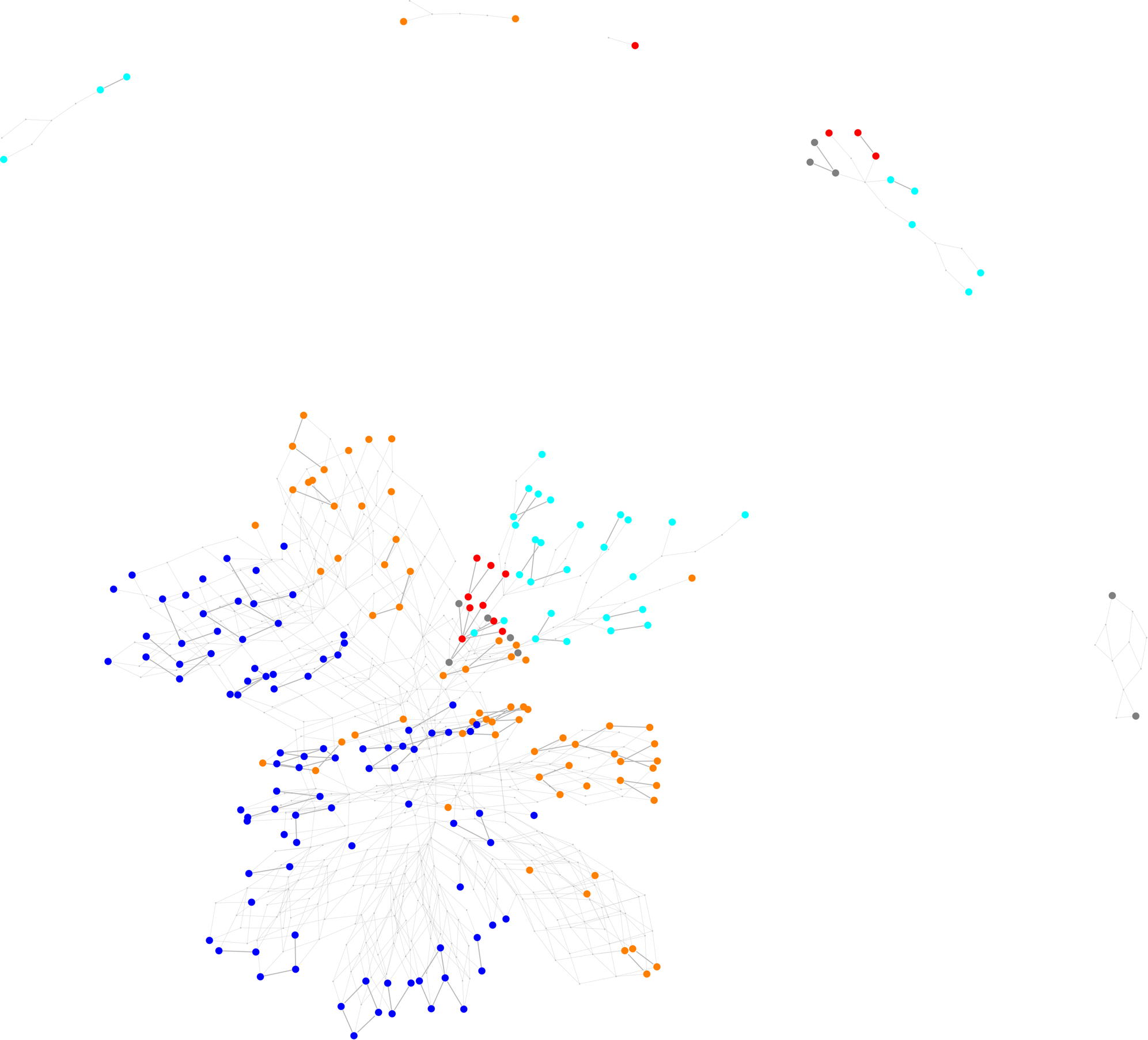

Supplement: Supplementary file 2 — Supplementary Information. [file 41598_2023_51012_MOESM2_ESM.zip › gutGH-SI/Networks/UniProt-HMO-networks-gut/p8090-GH-network-pp-hmo.jpg]

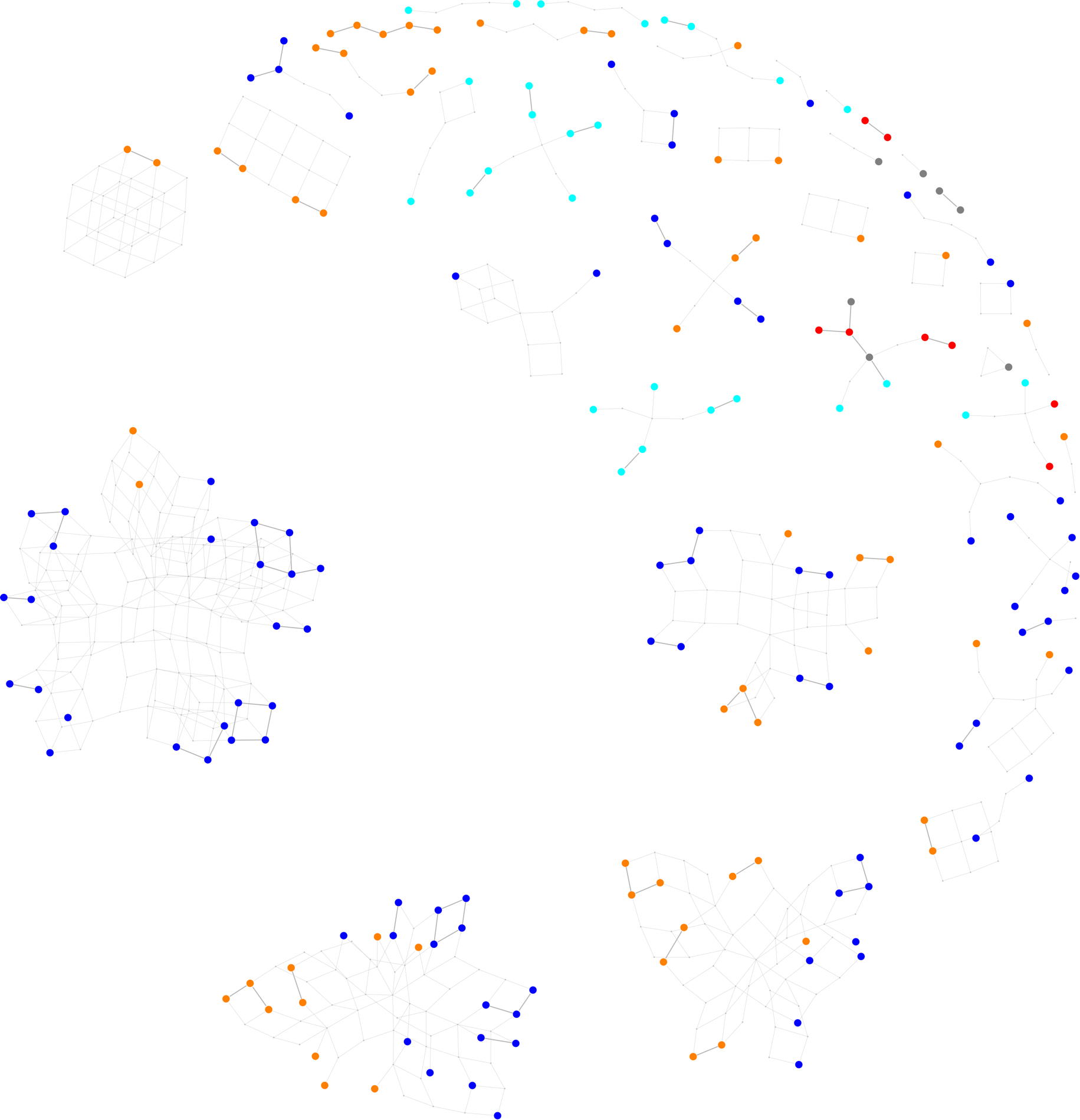

Supplement: Supplementary file 2 — Supplementary Information. [file 41598_2023_51012_MOESM2_ESM.zip › gutGH-SI/Networks/UniProt-HMO-networks-gut/p8155-GH-network-pp-hmo.jpg]

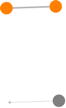

Supplement: Supplementary file 2 — Supplementary Information. [file 41598_2023_51012_MOESM2_ESM.zip › gutGH-SI/Networks/UniProt-HMO-networks-gut/p8127-GH-network-pp-hmo.jpg]

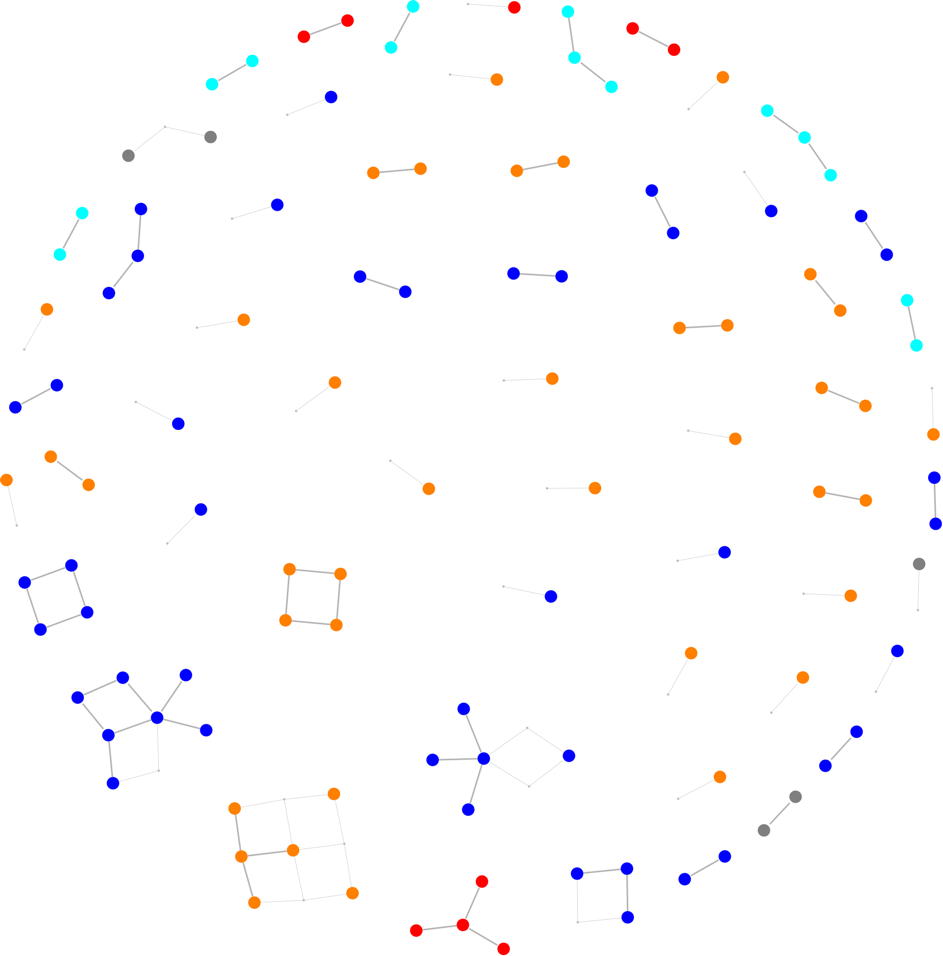

Supplement: Supplementary file 2 — Supplementary Information. [file 41598_2023_51012_MOESM2_ESM.zip › gutGH-SI/Networks/UniProt-HMO-networks-gut/p8191-GH-network-pp-hmo.jpg]

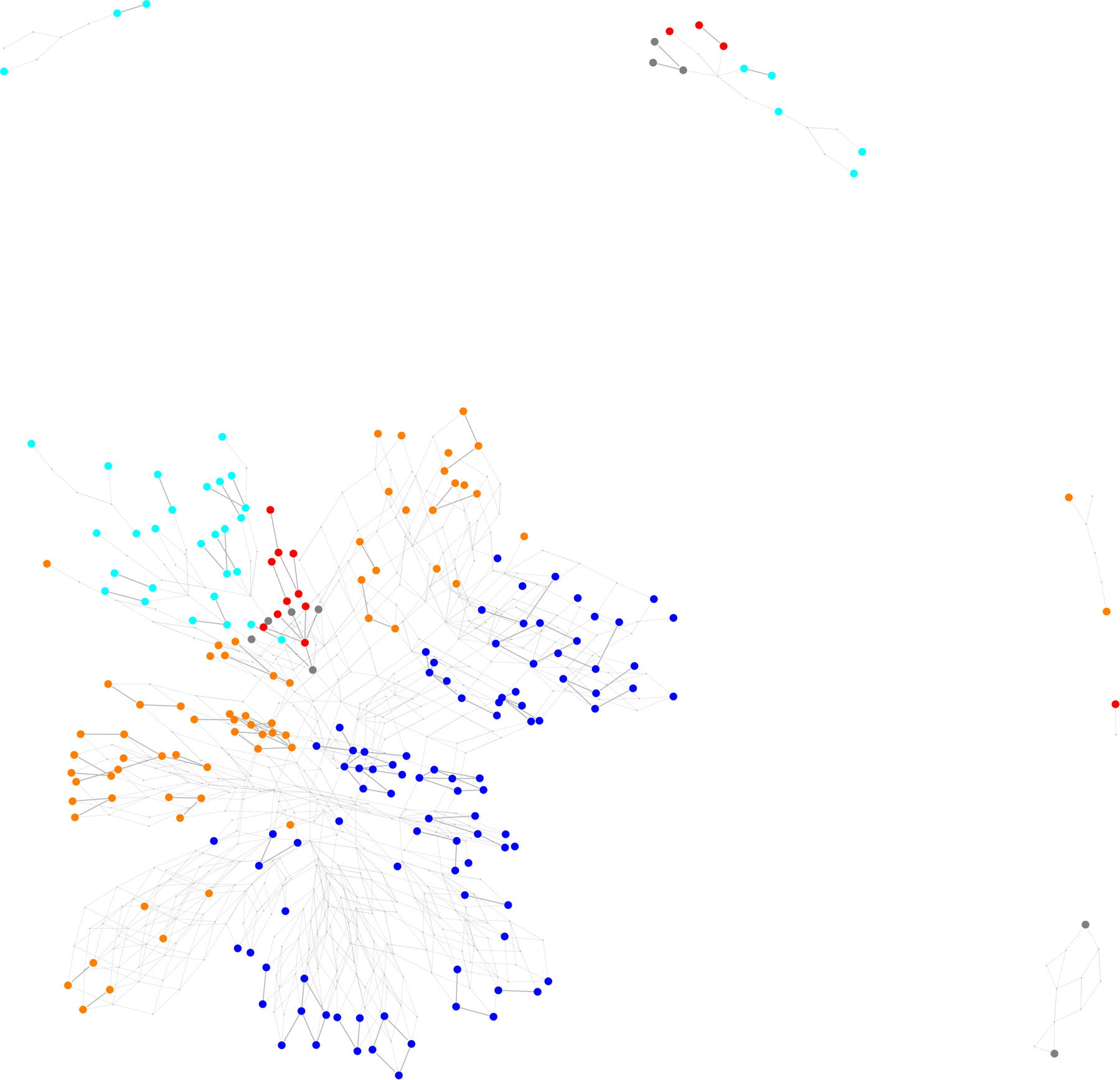

Supplement: Supplementary file 2 — Supplementary Information. [file 41598_2023_51012_MOESM2_ESM.zip › gutGH-SI/Networks/UniProt-HMO-networks-gut/p6930-GH-network-pp-hmo.jpg]

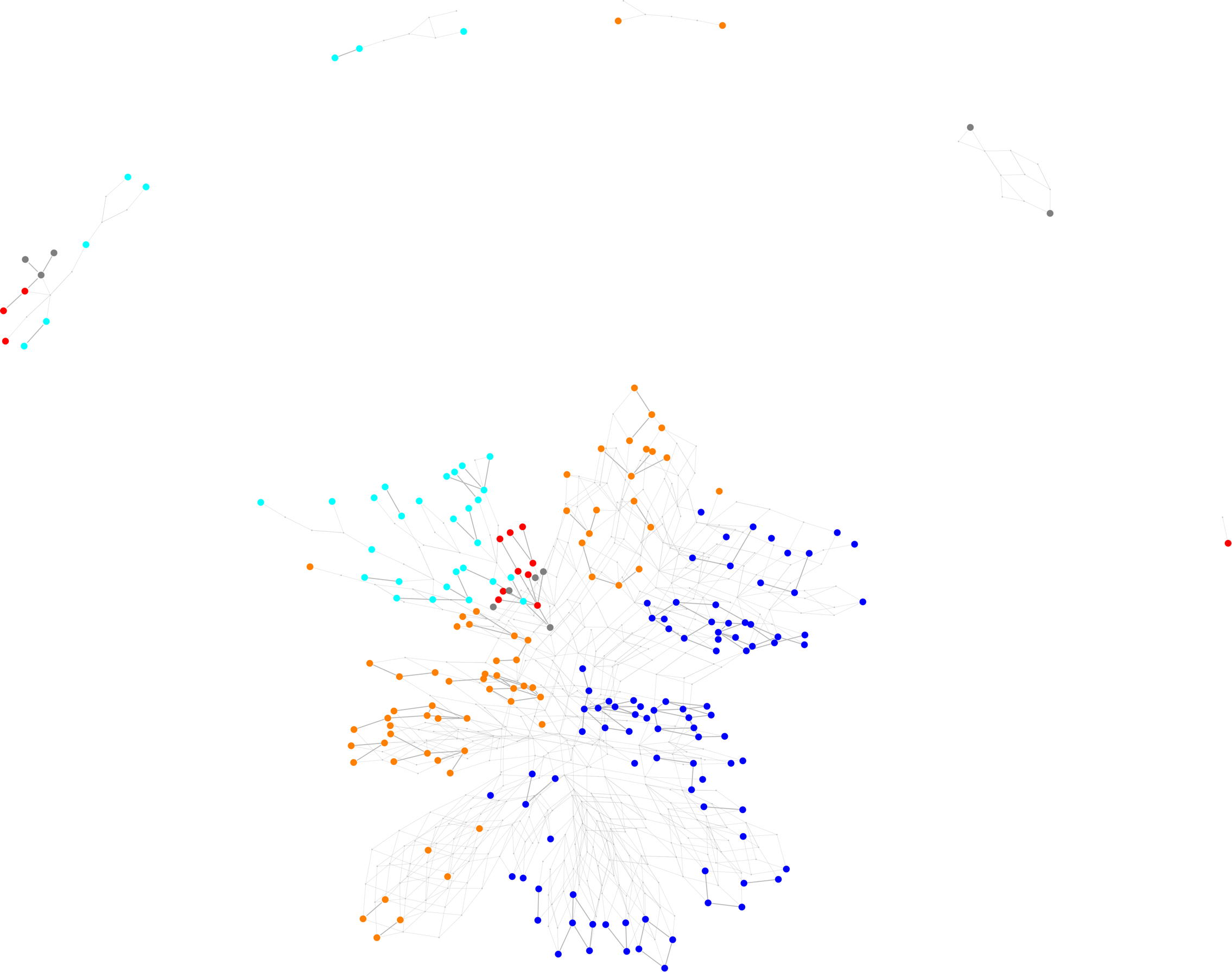

Supplement: Supplementary file 2 — Supplementary Information. [file 41598_2023_51012_MOESM2_ESM.zip › gutGH-SI/Networks/UniProt-HMO-networks-gut/p5018-GH-network-pp-hmo.jpg]

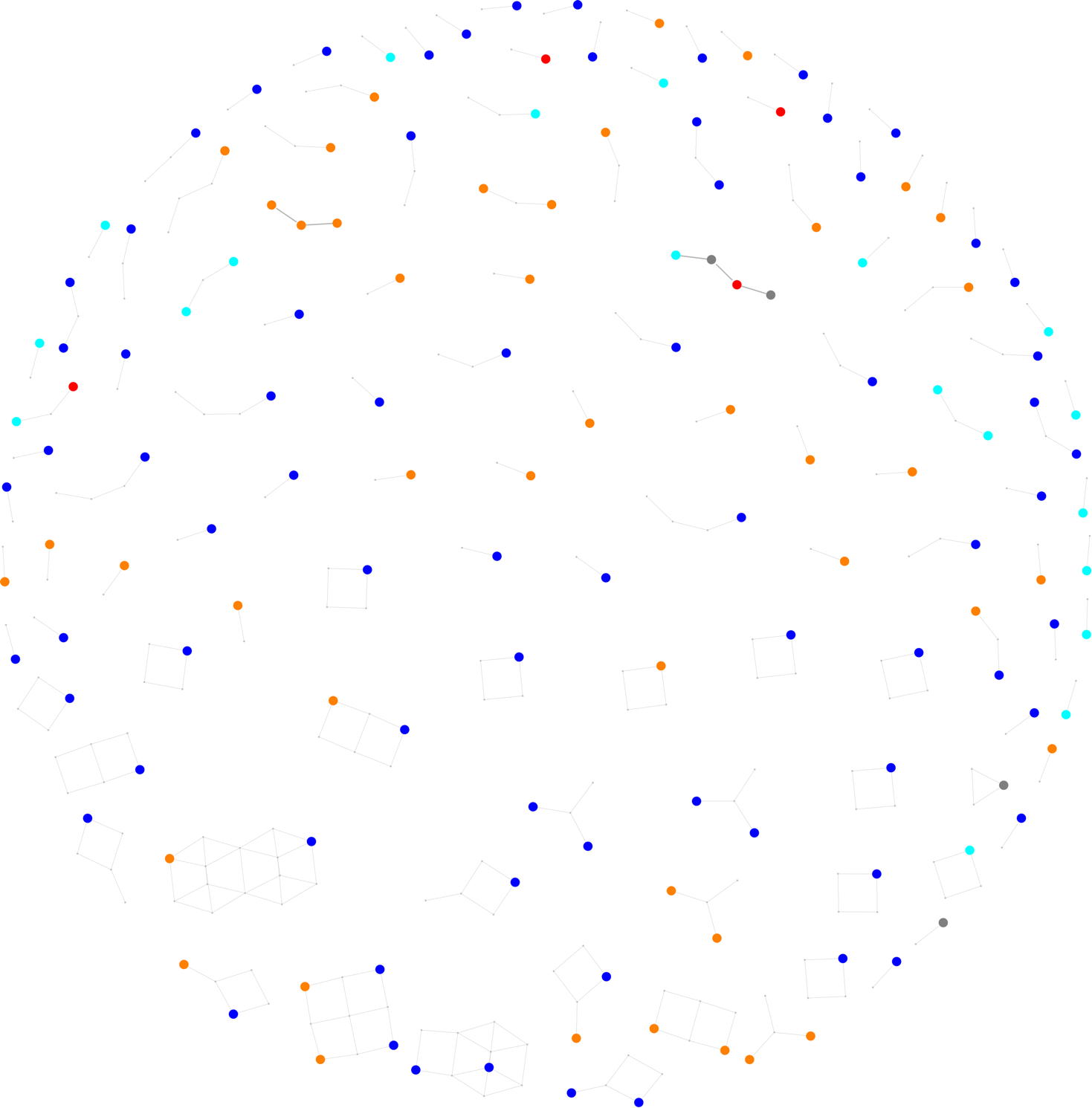

Supplement: Supplementary file 2 — Supplementary Information. [file 41598_2023_51012_MOESM2_ESM.zip › gutGH-SI/Networks/UniProt-HMO-networks-gut/p8187-GH-network-pp-hmo.jpg]

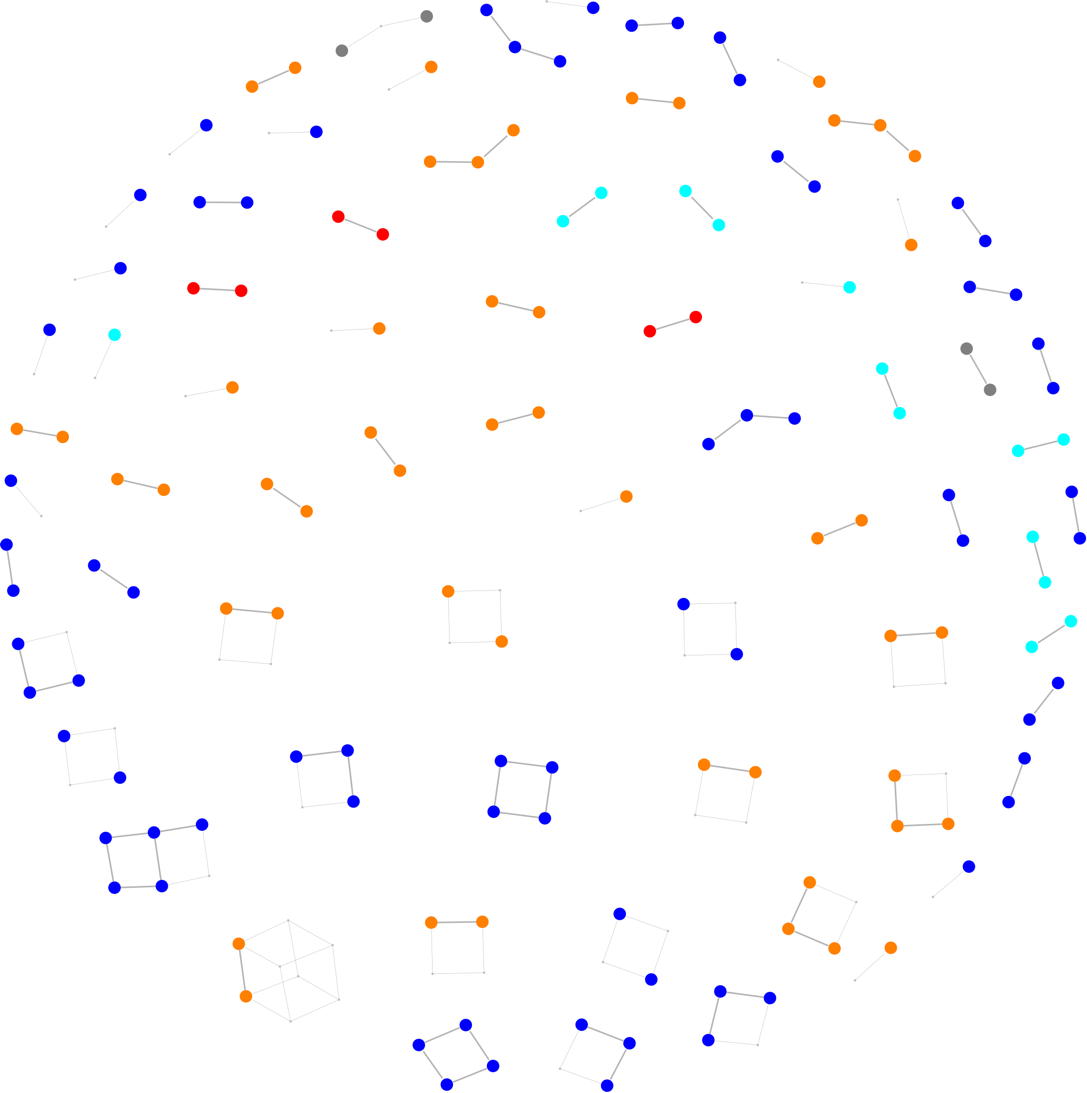

Supplement: Supplementary file 2 — Supplementary Information. [file 41598_2023_51012_MOESM2_ESM.zip › gutGH-SI/Networks/UniProt-HMO-networks-gut/p8095-GH-network-pp-hmo.jpg]

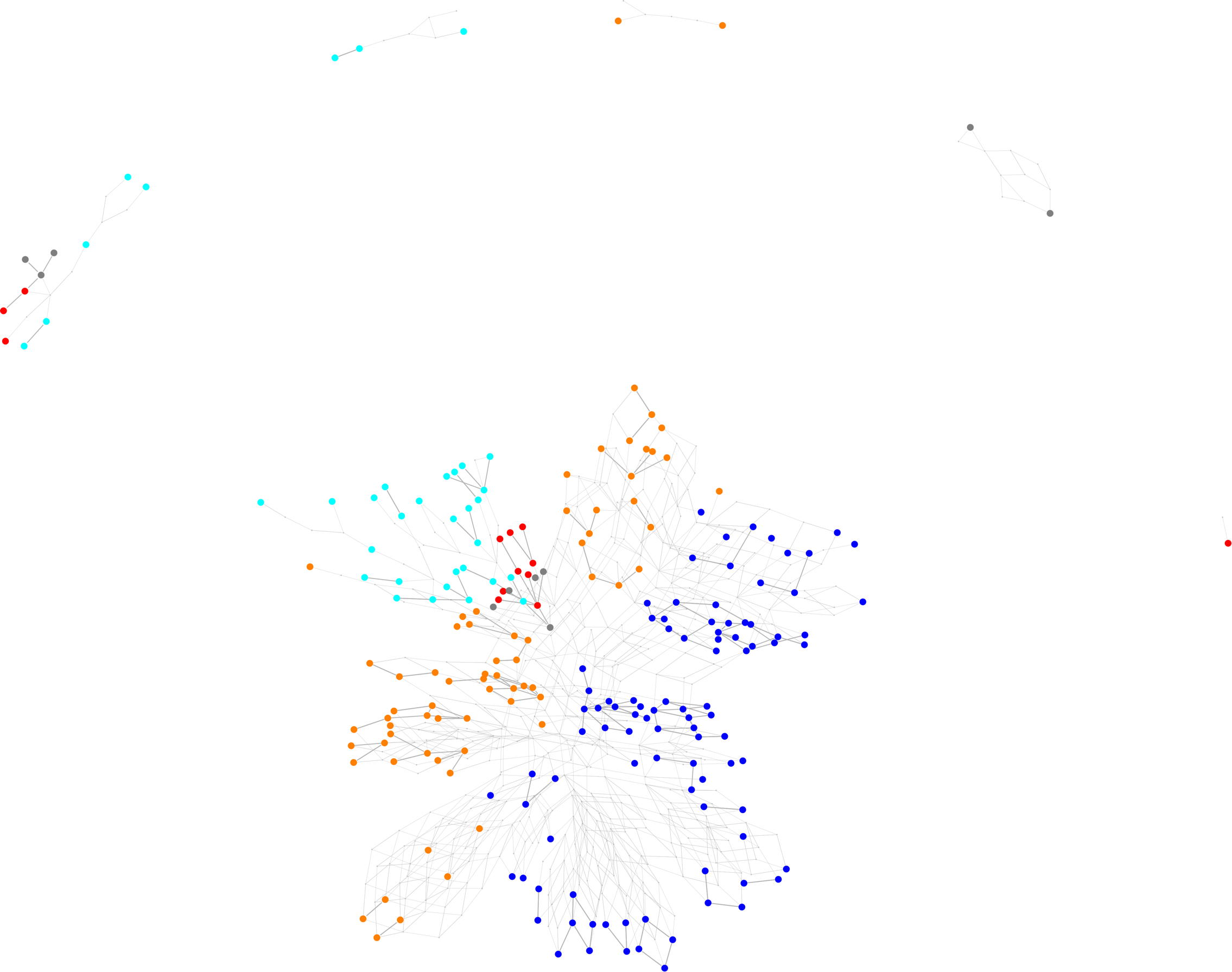

Supplement: Supplementary file 2 — Supplementary Information. [file 41598_2023_51012_MOESM2_ESM.zip › gutGH-SI/Networks/UniProt-HMO-networks-gut/p4506-GH-network-pp-hmo.jpg]

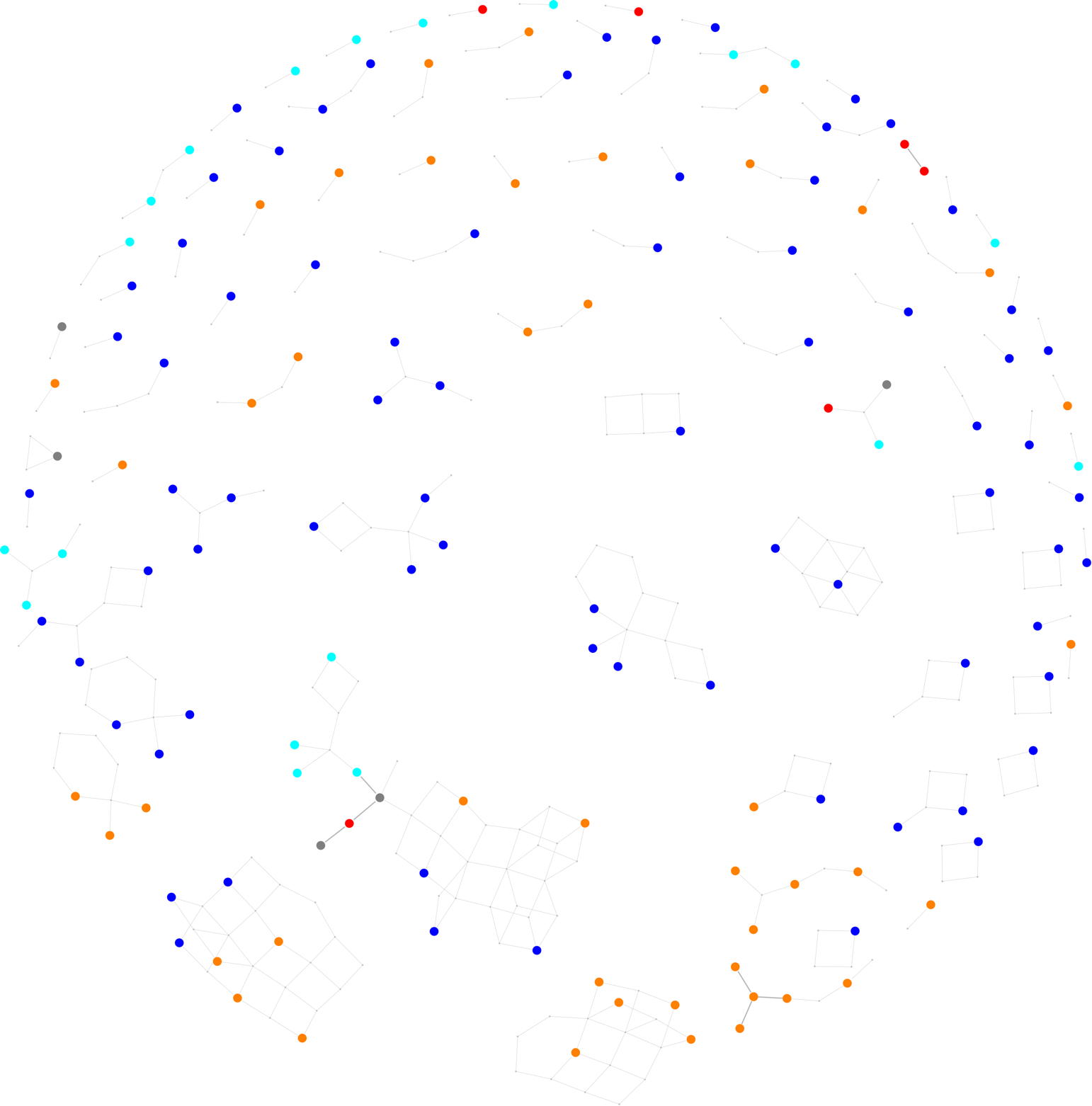

Supplement: Supplementary file 2 — Supplementary Information. [file 41598_2023_51012_MOESM2_ESM.zip › gutGH-SI/Networks/UniProt-HMO-networks-gut/p7987-GH-network-pp-hmo.jpg]

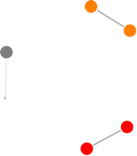

Supplement: Supplementary file 2 — Supplementary Information. [file 41598_2023_51012_MOESM2_ESM.zip › gutGH-SI/Networks/UniProt-HMO-networks-gut/p7991-GH-network-pp-hmo.jpg]

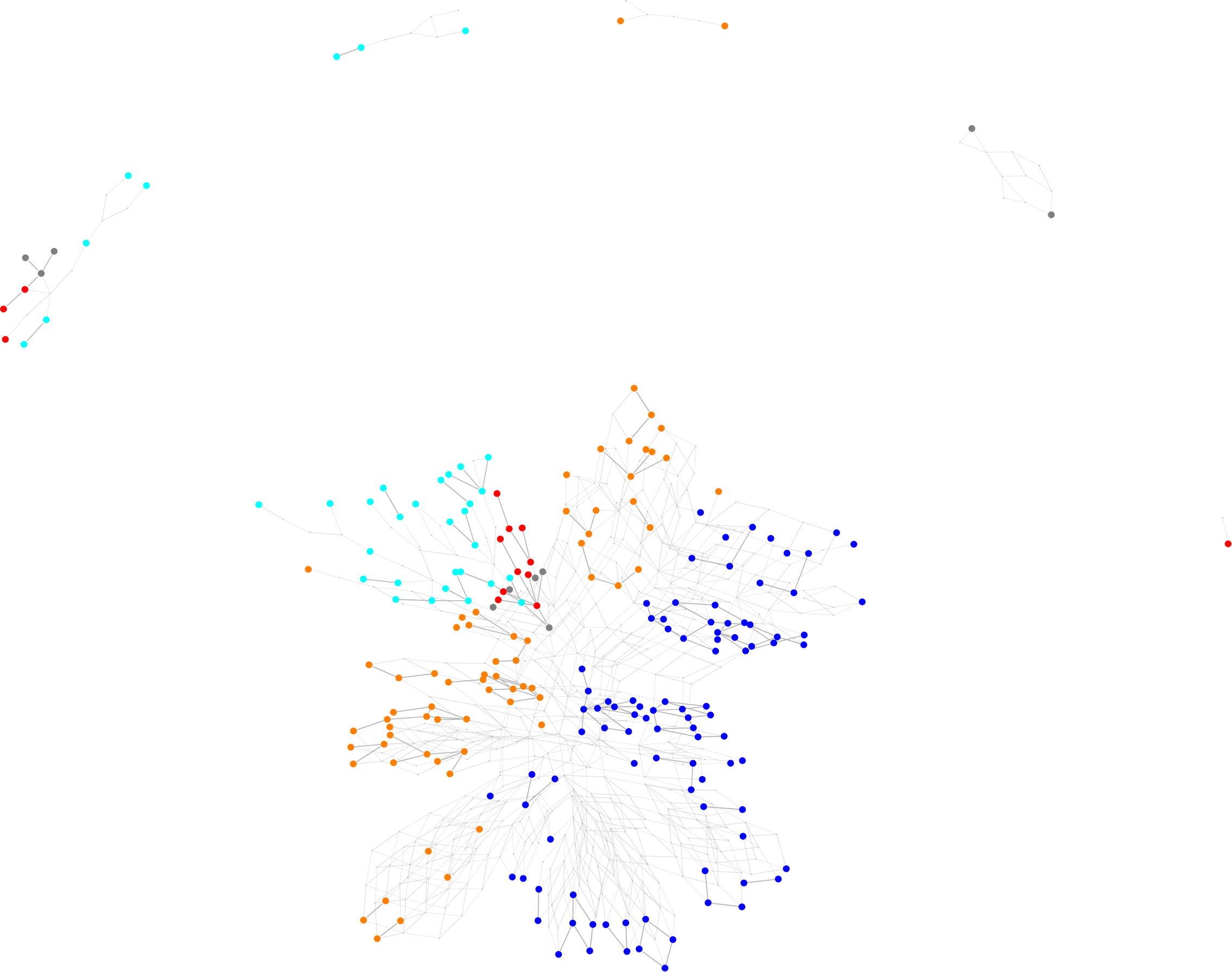

Supplement: Supplementary file 2 — Supplementary Information. [file 41598_2023_51012_MOESM2_ESM.zip › gutGH-SI/Networks/UniProt-HMO-networks-gut/p274-GH-network-pp-hmo.jpg]

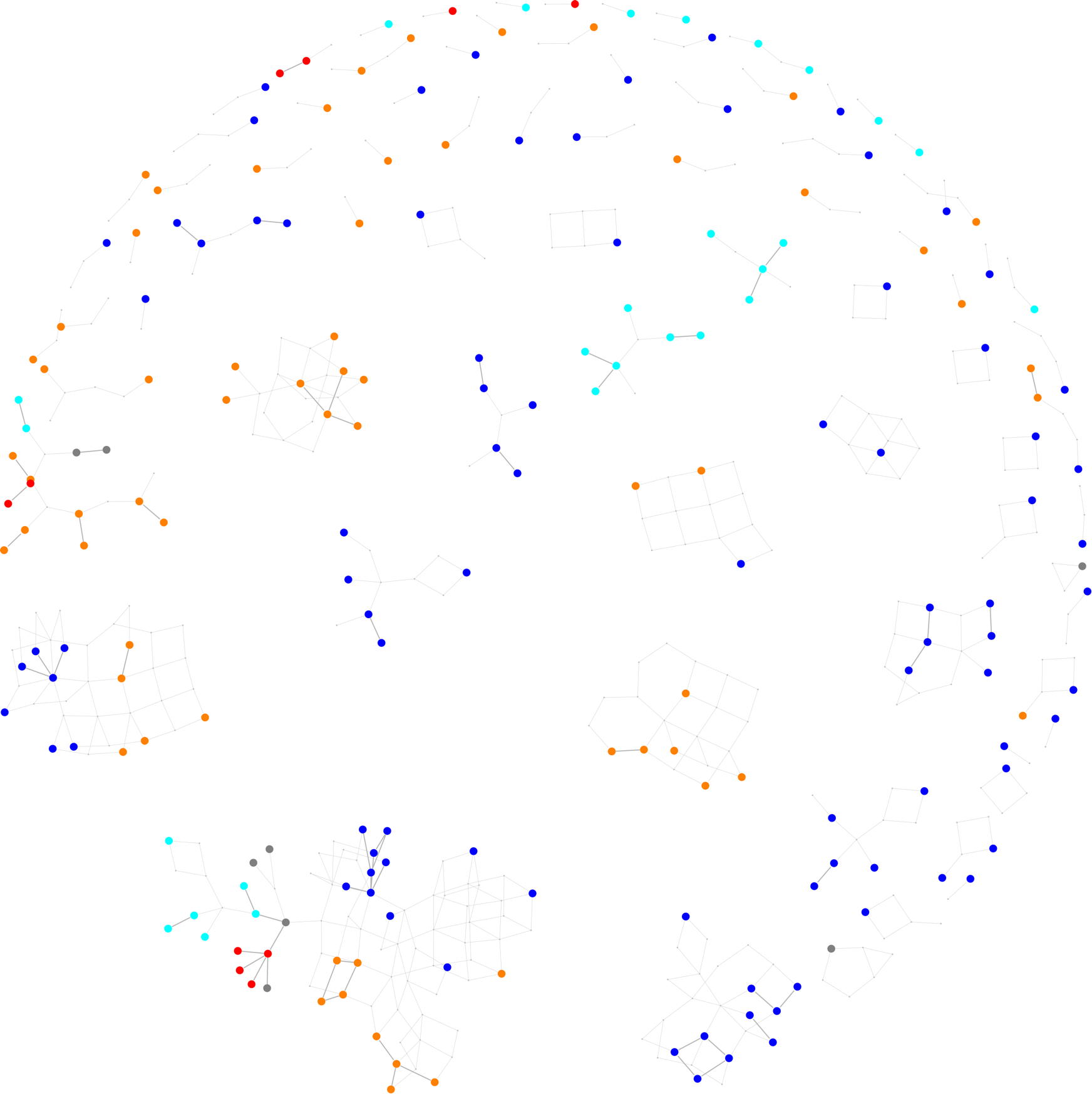

Supplement: Supplementary file 2 — Supplementary Information. [file 41598_2023_51012_MOESM2_ESM.zip › gutGH-SI/Networks/UniProt-HMO-networks-gut/p8122-GH-network-pp-hmo.jpg]

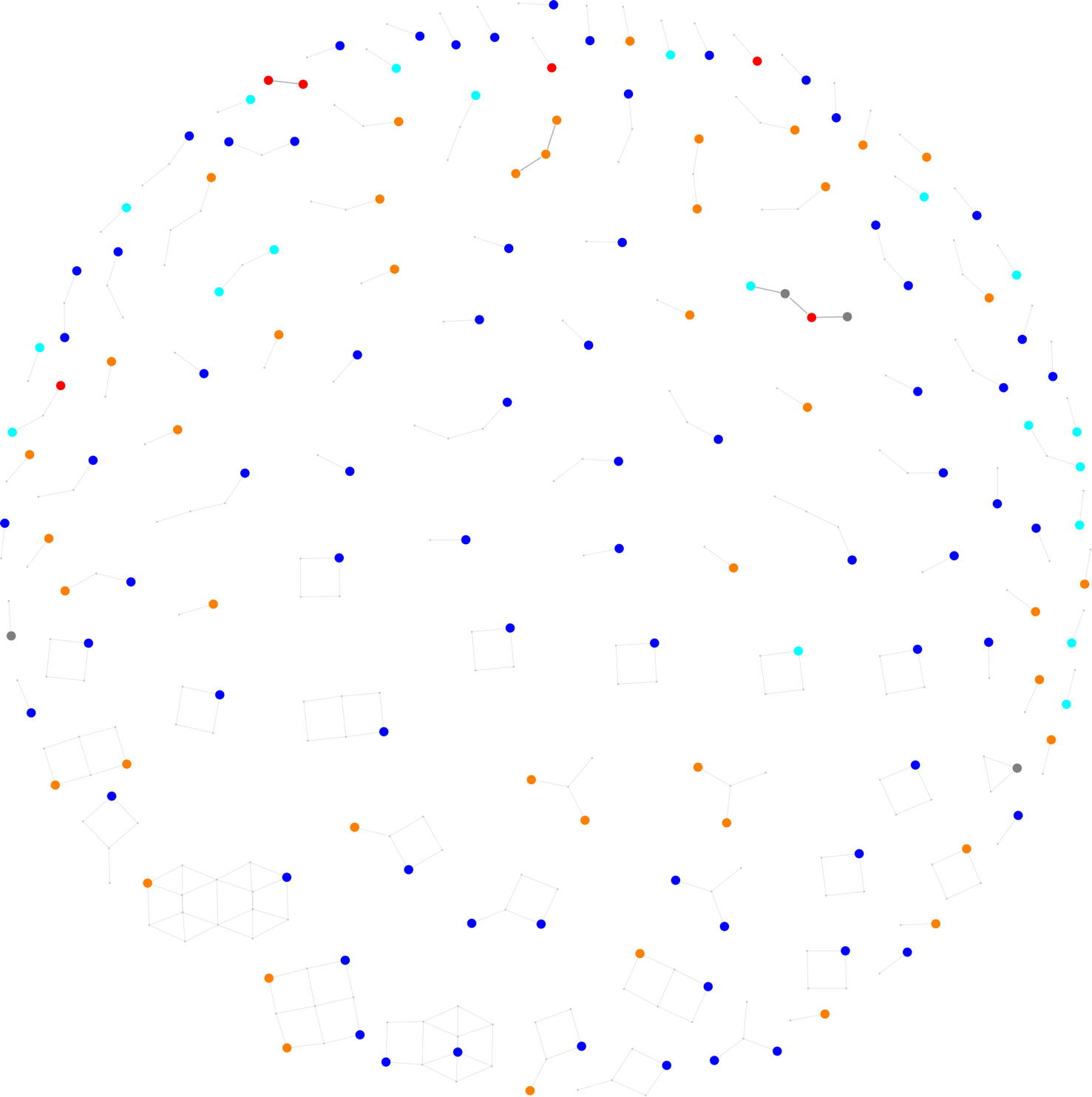

Supplement: Supplementary file 2 — Supplementary Information. [file 41598_2023_51012_MOESM2_ESM.zip › gutGH-SI/Networks/UniProt-HMO-networks-gut/p8051-GH-network-pp-hmo.jpg]

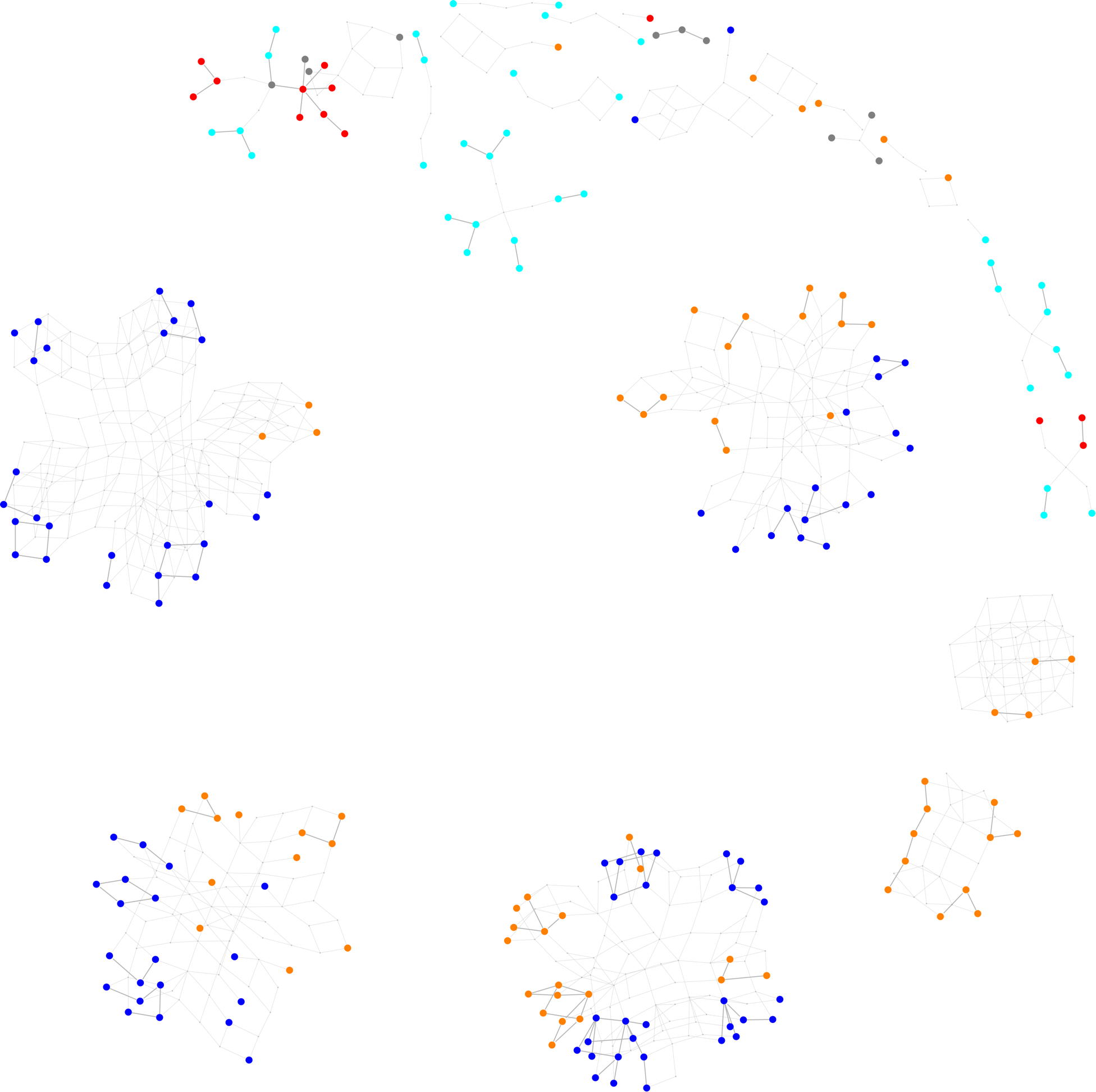

Supplement: Supplementary file 2 — Supplementary Information. [file 41598_2023_51012_MOESM2_ESM.zip › gutGH-SI/Networks/UniProt-HMO-networks-gut/p8154-GH-network-pp-hmo.jpg]

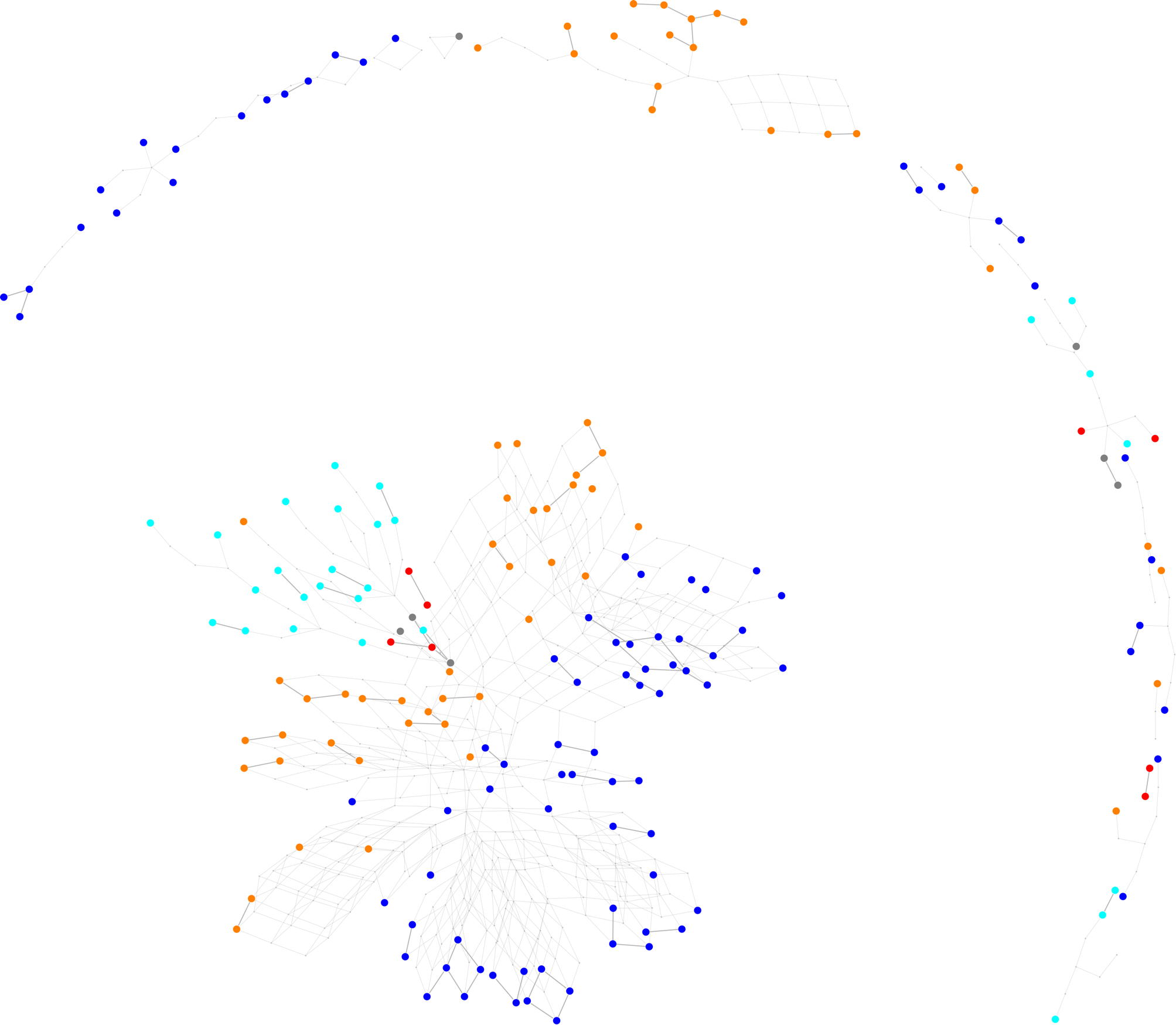

Supplement: Supplementary file 2 — Supplementary Information. [file 41598_2023_51012_MOESM2_ESM.zip › gutGH-SI/Networks/UniProt-HMO-networks-gut/p8091-GH-network-pp-hmo.jpg]

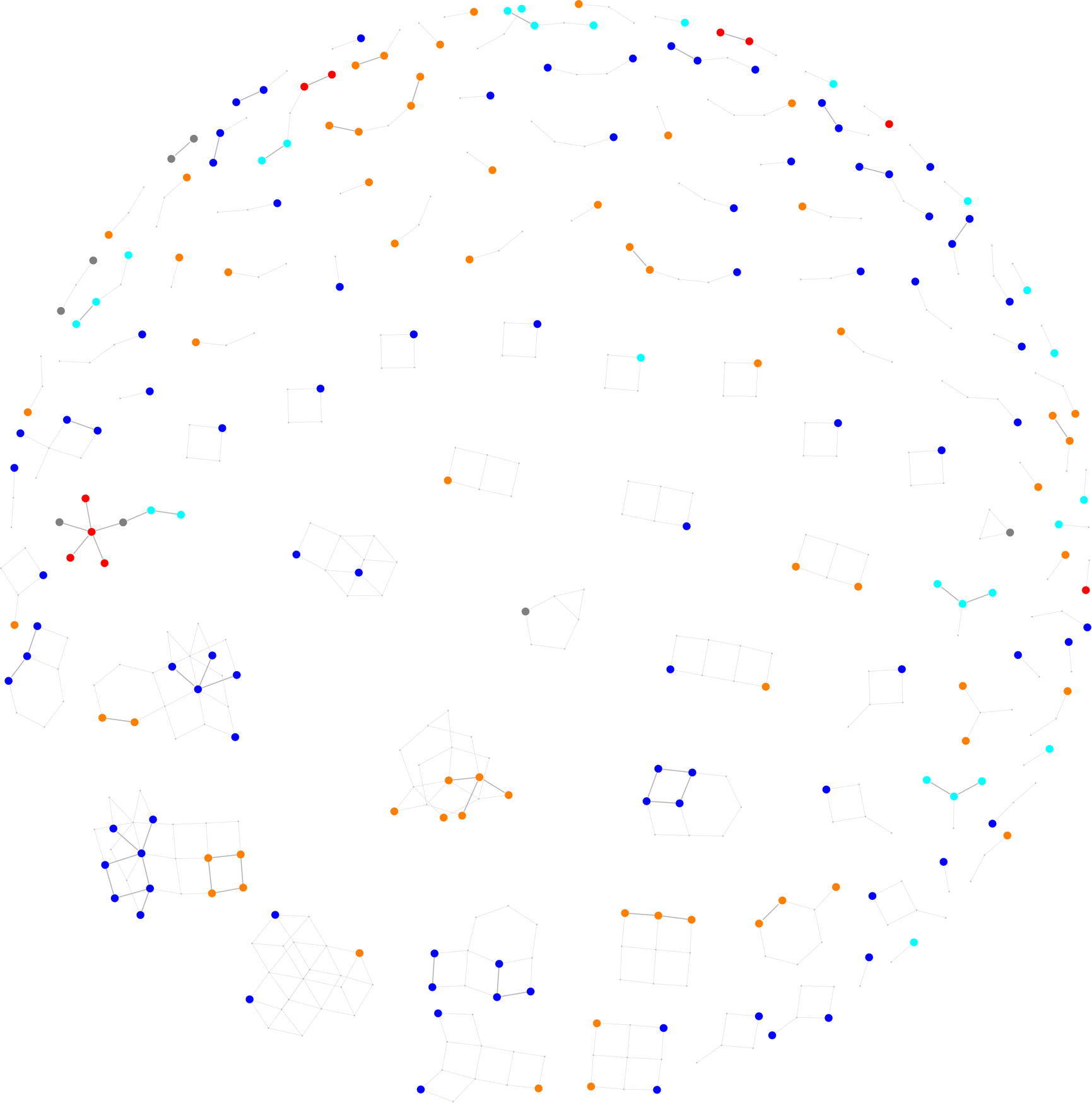

Supplement: Supplementary file 2 — Supplementary Information. [file 41598_2023_51012_MOESM2_ESM.zip › gutGH-SI/Networks/UniProt-HMO-networks-gut/p8186-GH-network-pp-hmo.jpg]

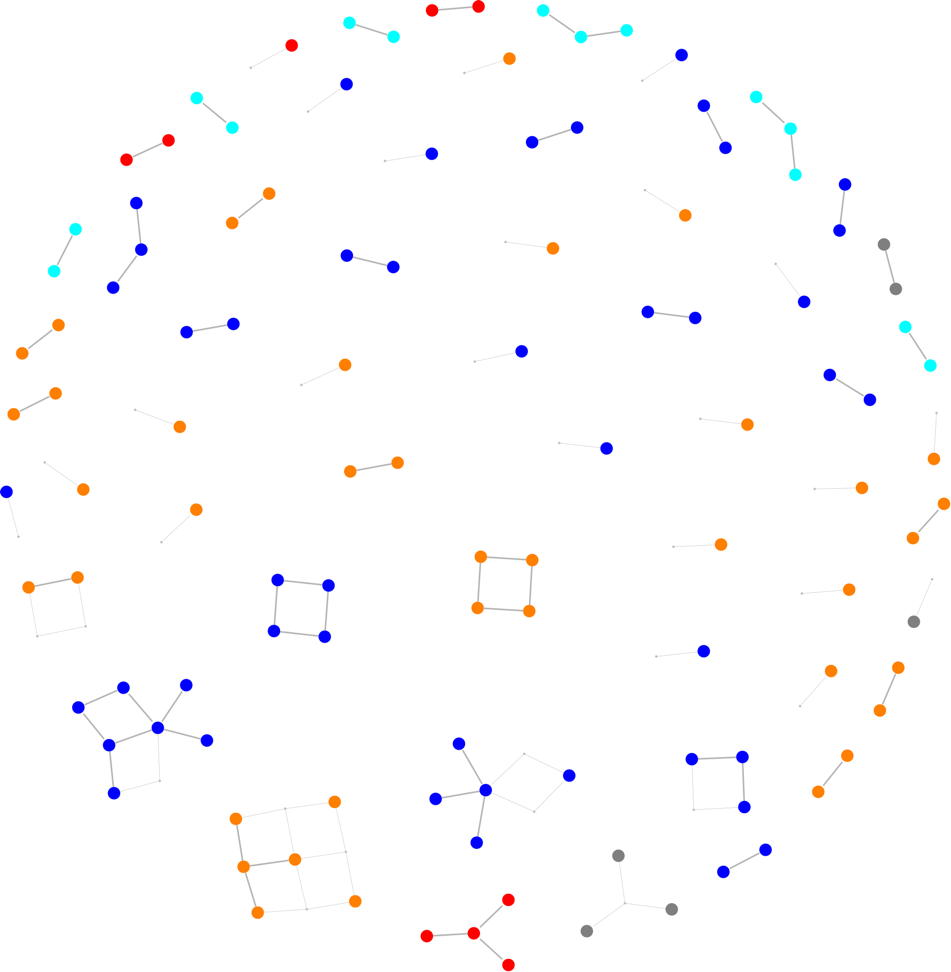

Supplement: Supplementary file 2 — Supplementary Information. [file 41598_2023_51012_MOESM2_ESM.zip › gutGH-SI/Networks/UniProt-HMO-networks-gut/p8126-GH-network-pp-hmo.jpg]

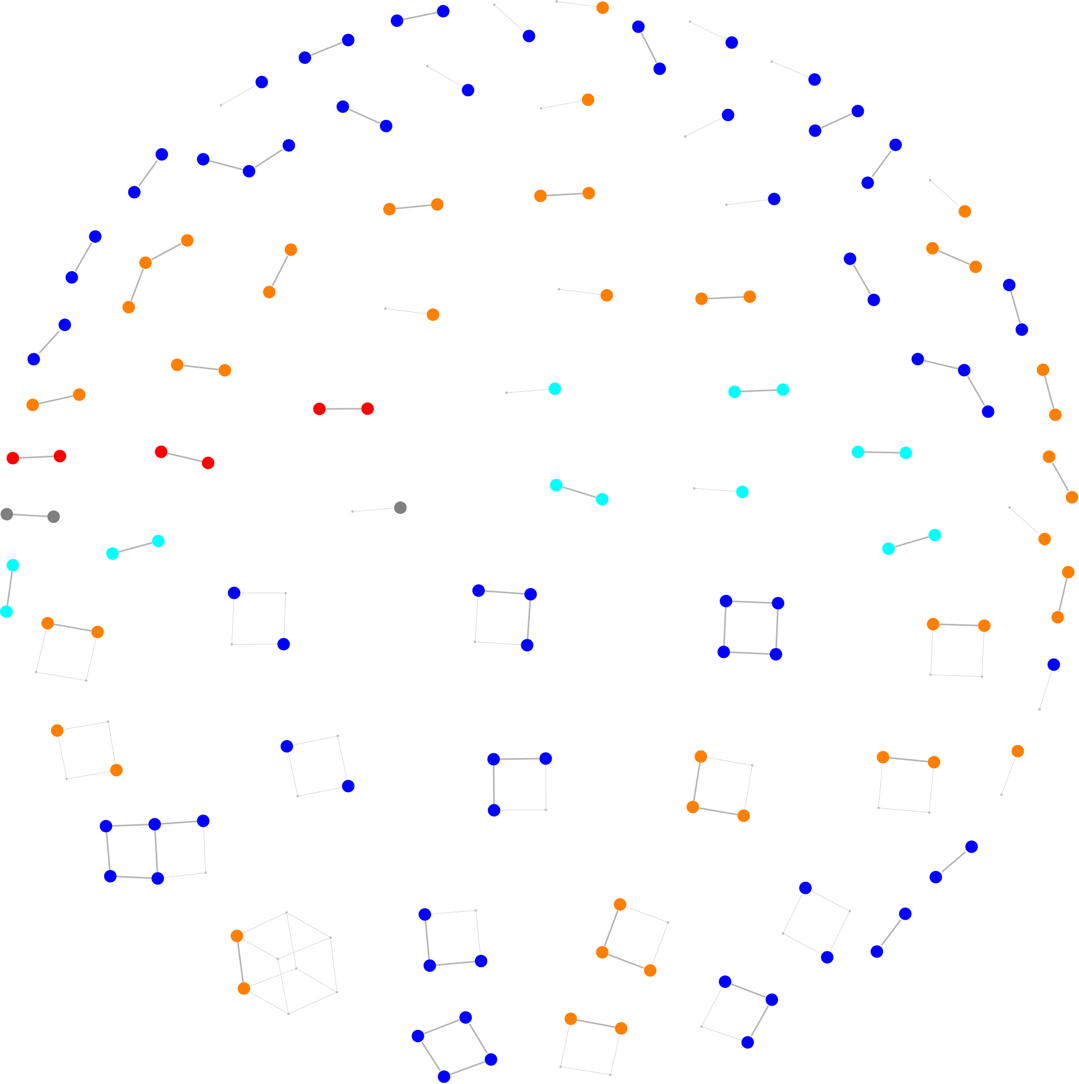

Supplement: Supplementary file 2 — Supplementary Information. [file 41598_2023_51012_MOESM2_ESM.zip › gutGH-SI/Networks/CAZy-HMO-networks-gut/p8159-GH-network-pp-hmo.jpg]

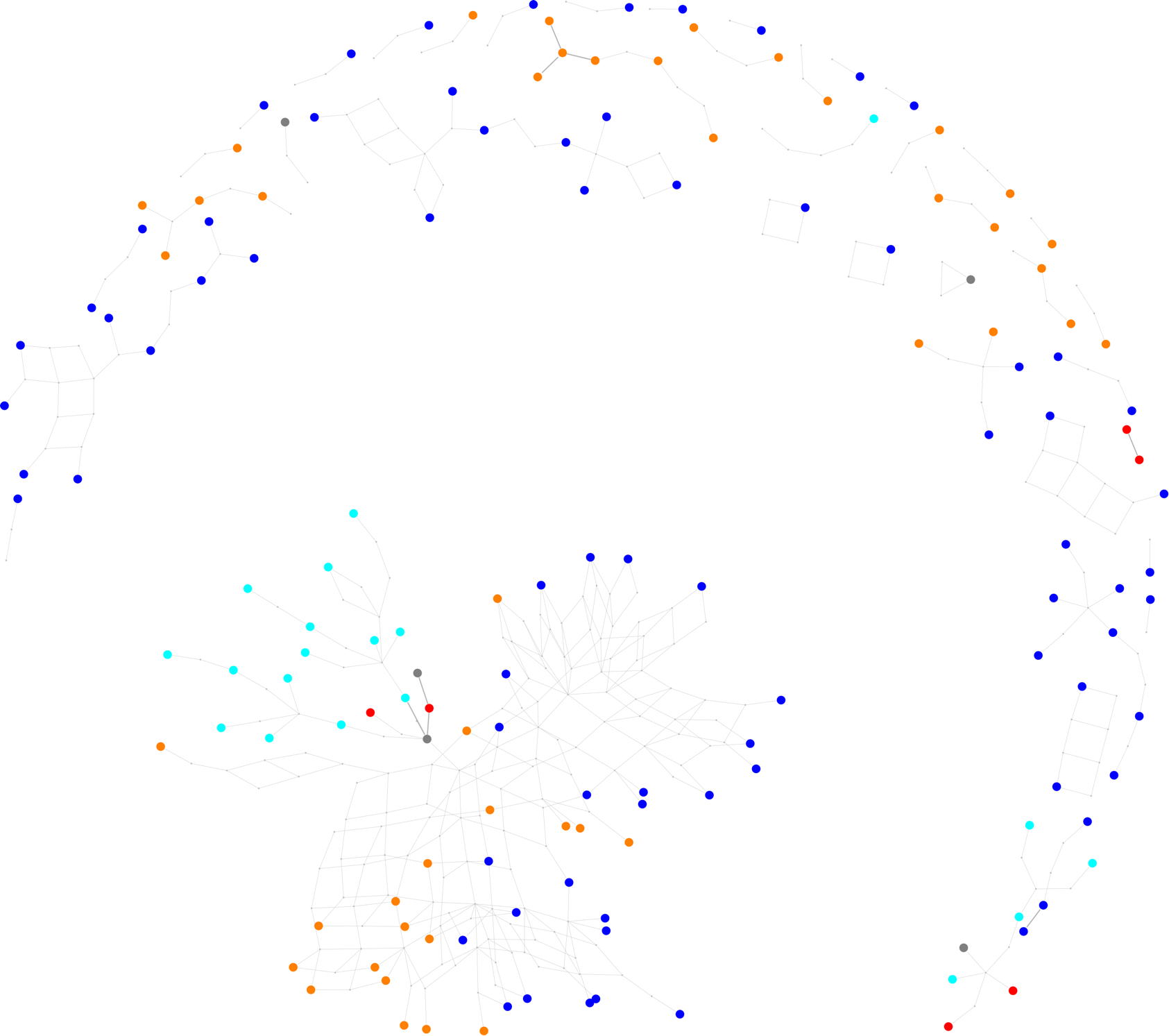

Supplement: Supplementary file 2 — Supplementary Information. [file 41598_2023_51012_MOESM2_ESM.zip › gutGH-SI/Networks/CAZy-HMO-networks-gut/p6963-GH-network-pp-hmo.jpg]

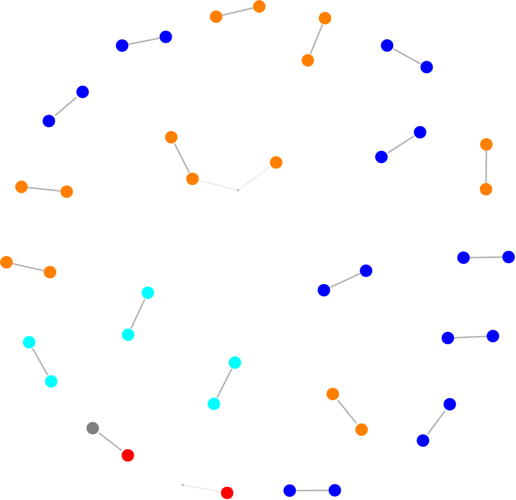

Supplement: Supplementary file 2 — Supplementary Information. [file 41598_2023_51012_MOESM2_ESM.zip › gutGH-SI/Networks/CAZy-HMO-networks-gut/p6143-GH-network-pp-hmo.jpg]

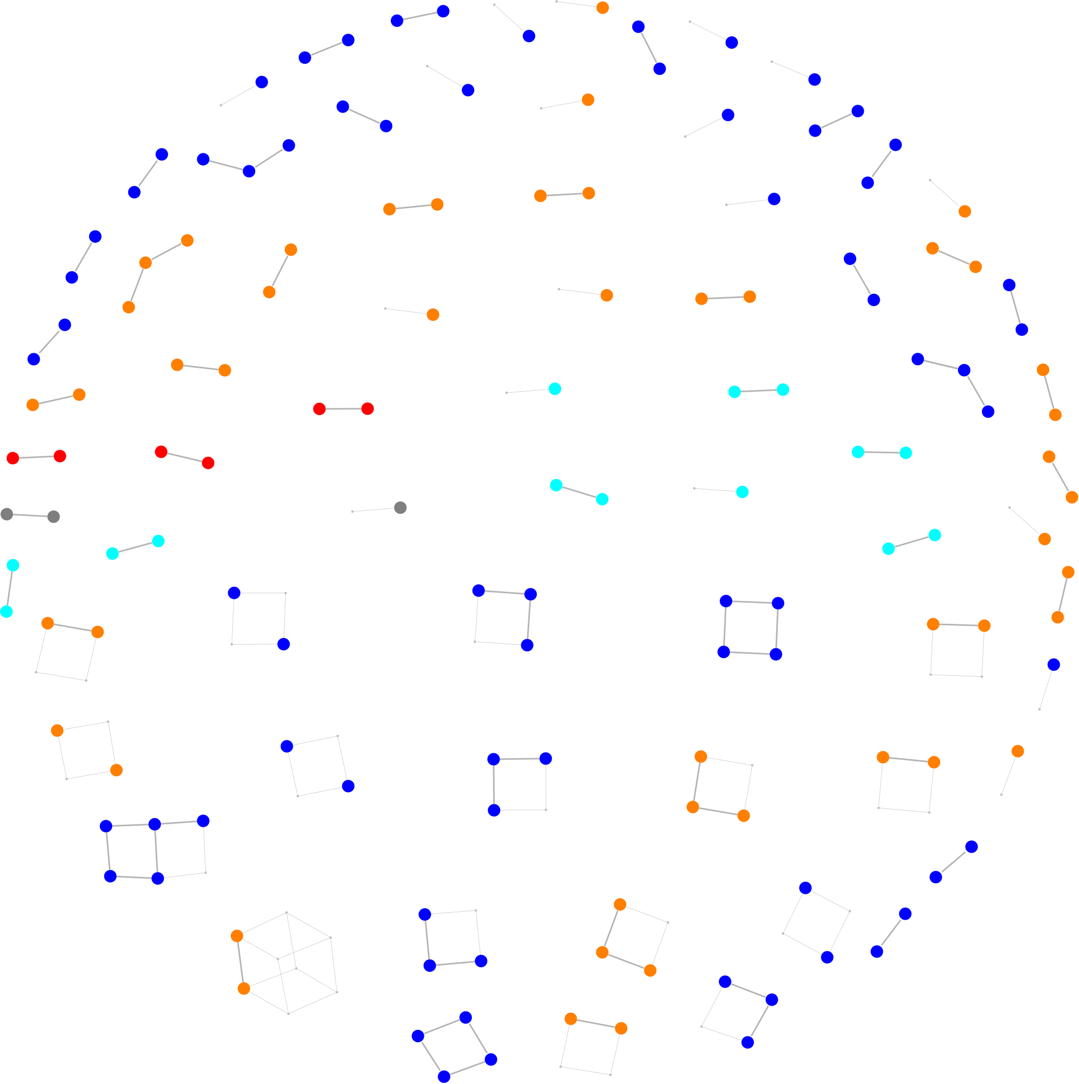

Supplement: Supplementary file 2 — Supplementary Information. [file 41598_2023_51012_MOESM2_ESM.zip › gutGH-SI/Networks/CAZy-HMO-networks-gut/p7135-GH-network-pp-hmo.jpg]

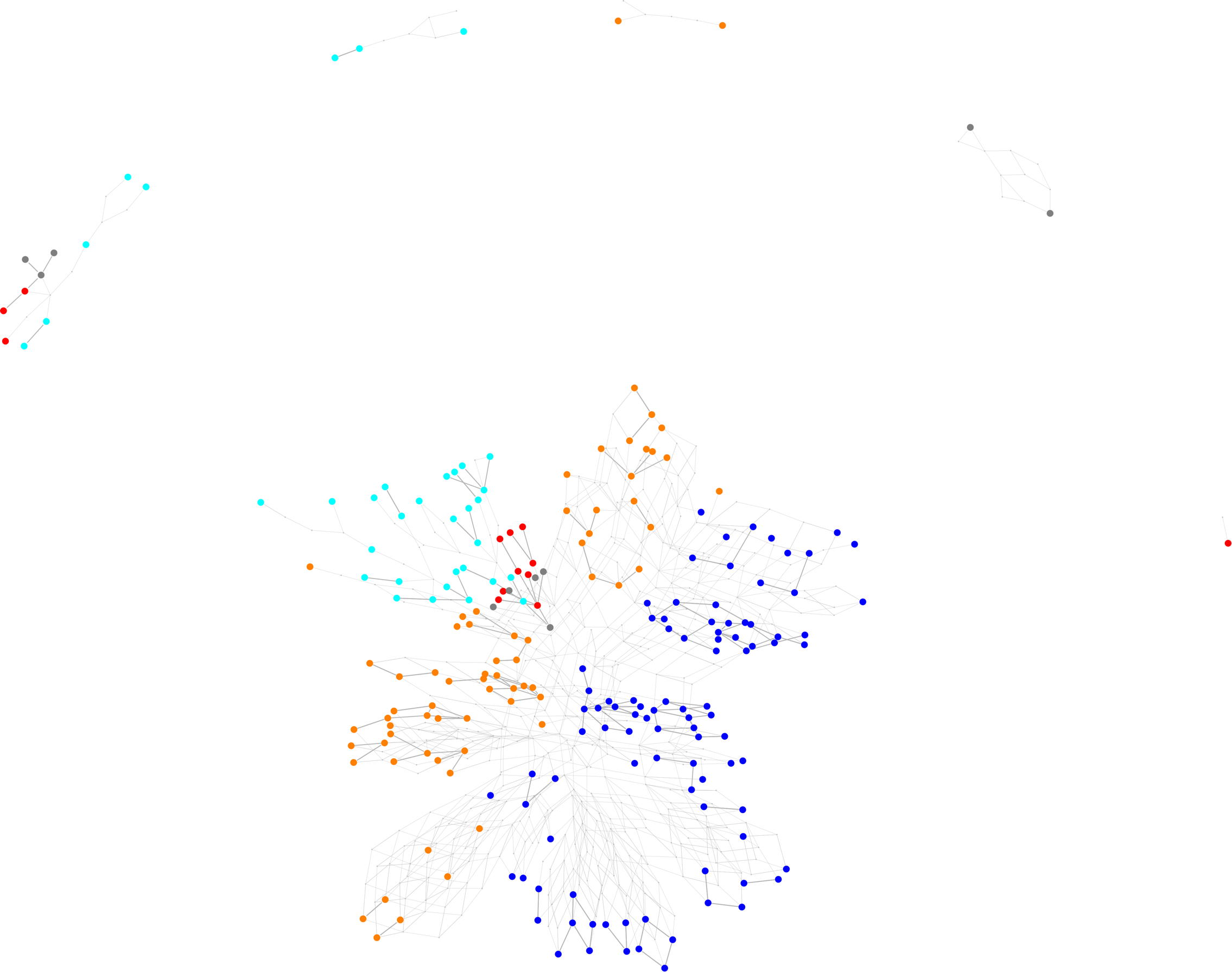

Supplement: Supplementary file 2 — Supplementary Information. [file 41598_2023_51012_MOESM2_ESM.zip › gutGH-SI/Networks/CAZy-HMO-networks-gut/p5530-GH-network-pp-hmo.jpg]

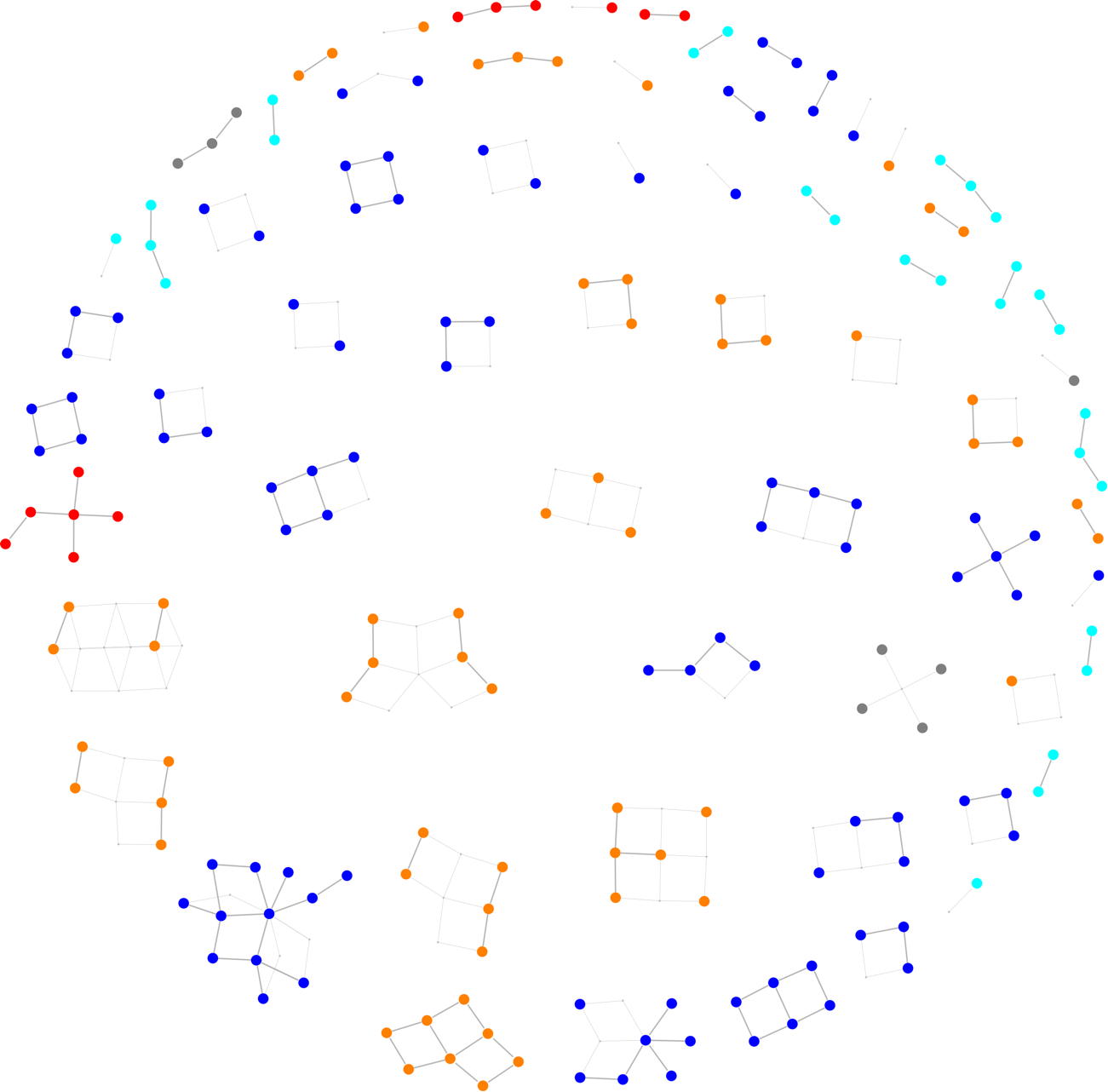

Supplement: Supplementary file 2 — Supplementary Information. [file 41598_2023_51012_MOESM2_ESM.zip › gutGH-SI/Networks/CAZy-HMO-networks-gut/p7070-GH-network-pp-hmo.jpg]

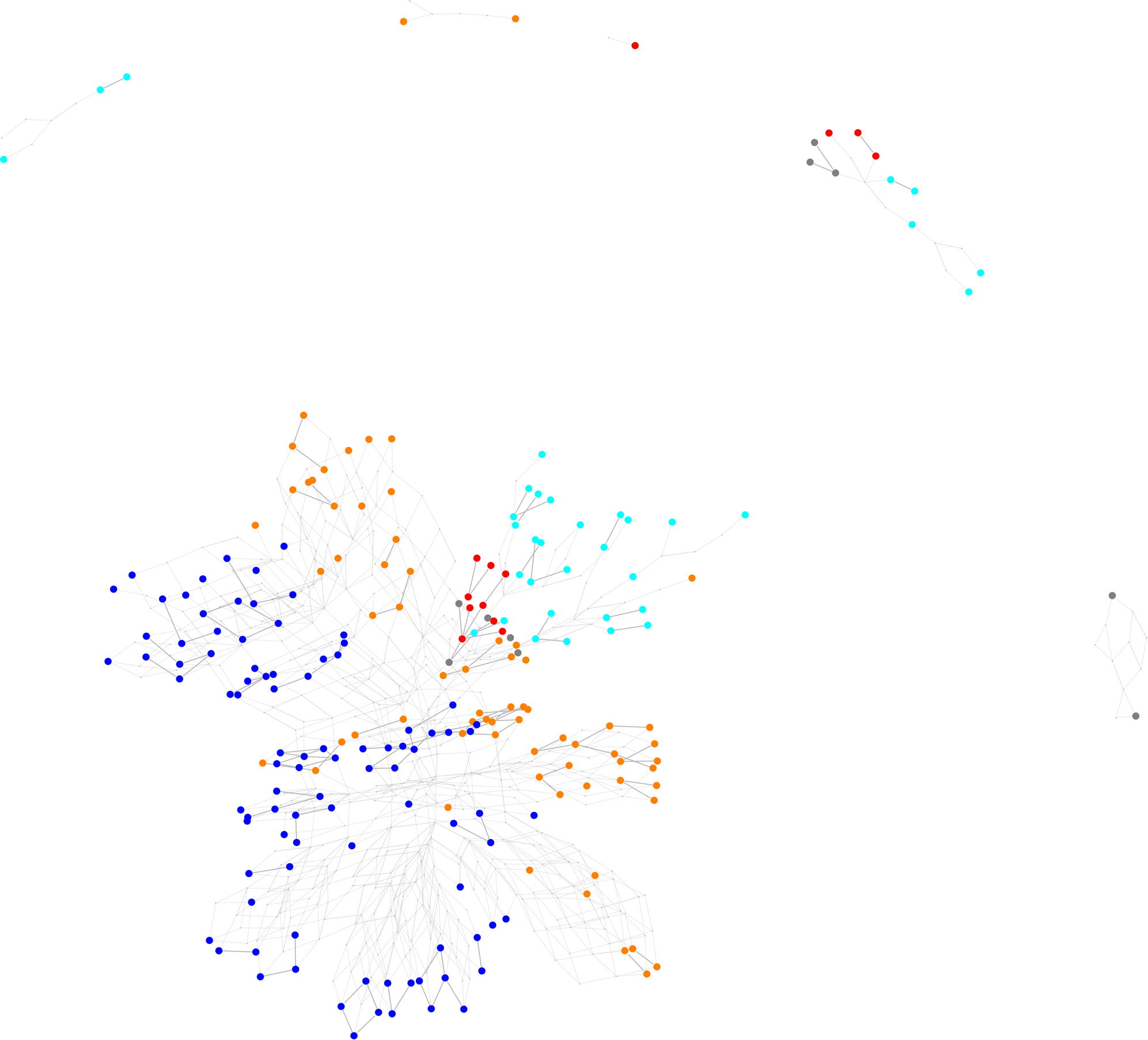

Supplement: Supplementary file 2 — Supplementary Information. [file 41598_2023_51012_MOESM2_ESM.zip › gutGH-SI/Networks/CAZy-HMO-networks-gut/p7066-GH-network-pp-hmo.jpg]

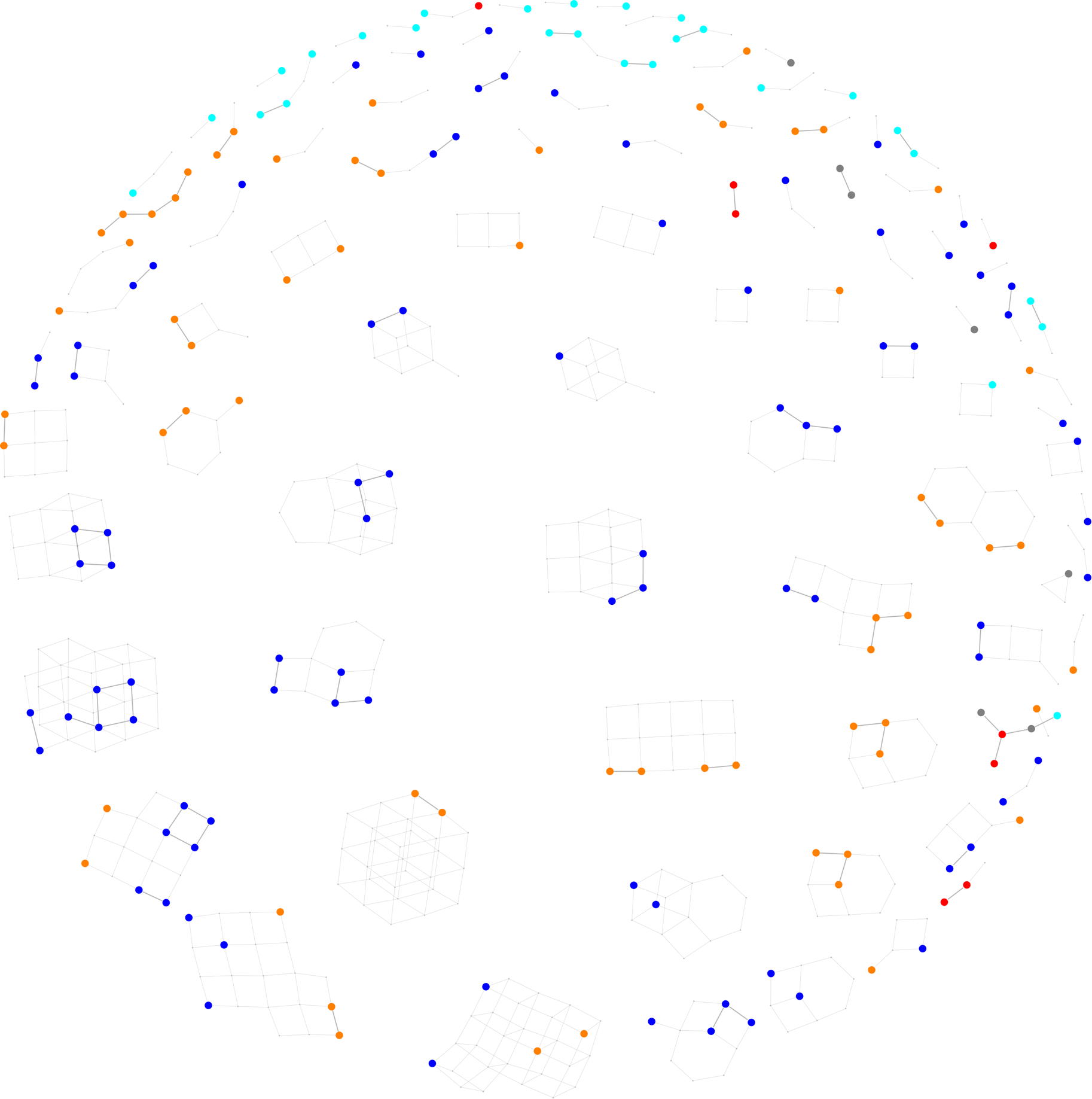

Supplement: Supplementary file 2 — Supplementary Information. [file 41598_2023_51012_MOESM2_ESM.zip › gutGH-SI/Networks/CAZy-HMO-networks-gut/p7675-GH-network-pp-hmo.jpg]

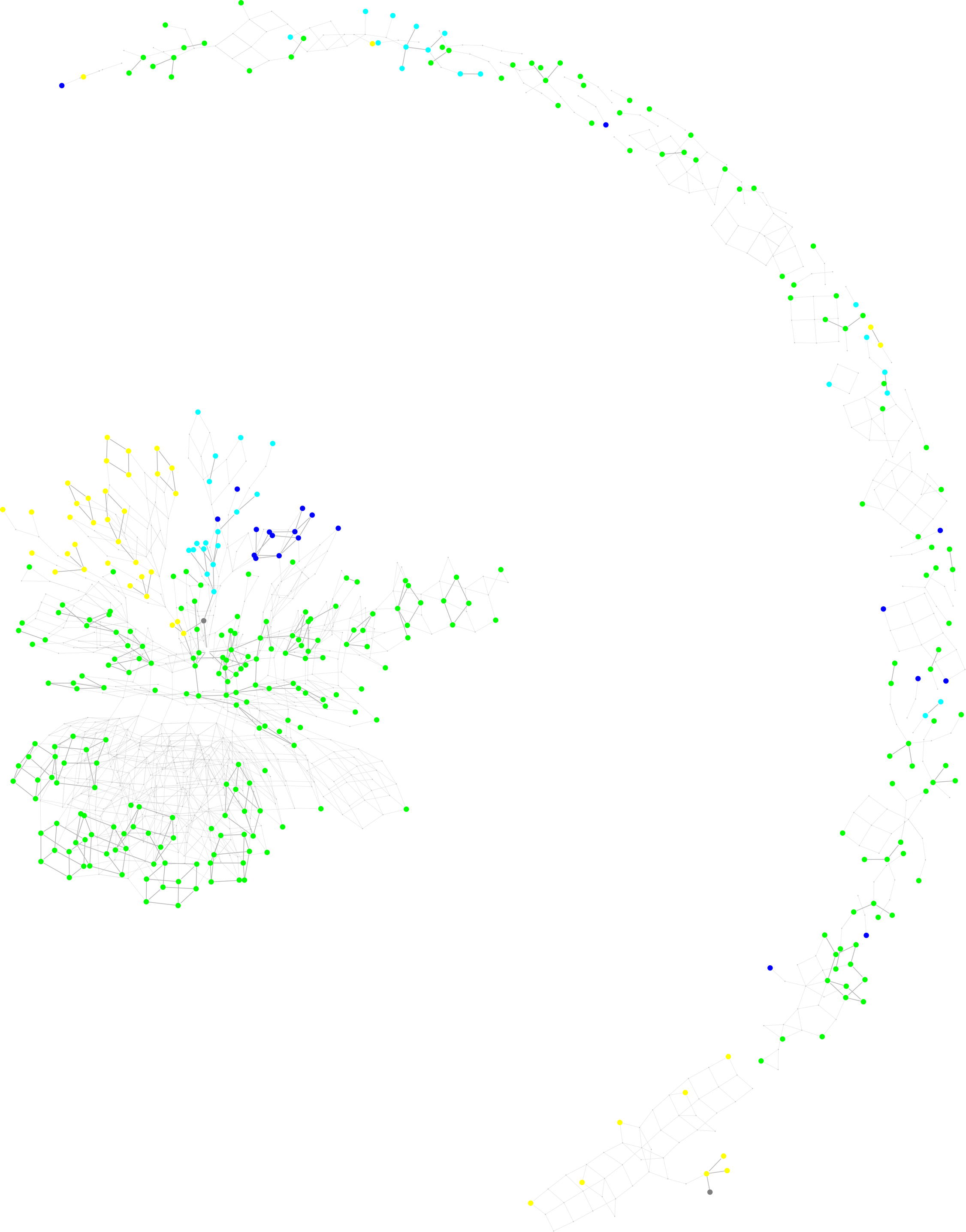

Supplement: Supplementary file 2 — Supplementary Information. [file 41598_2023_51012_MOESM2_ESM.zip › gutGH-SI/Networks/CAZY-O-glycan-networks-gut/p7066-GH-network-pp-og.jpg]

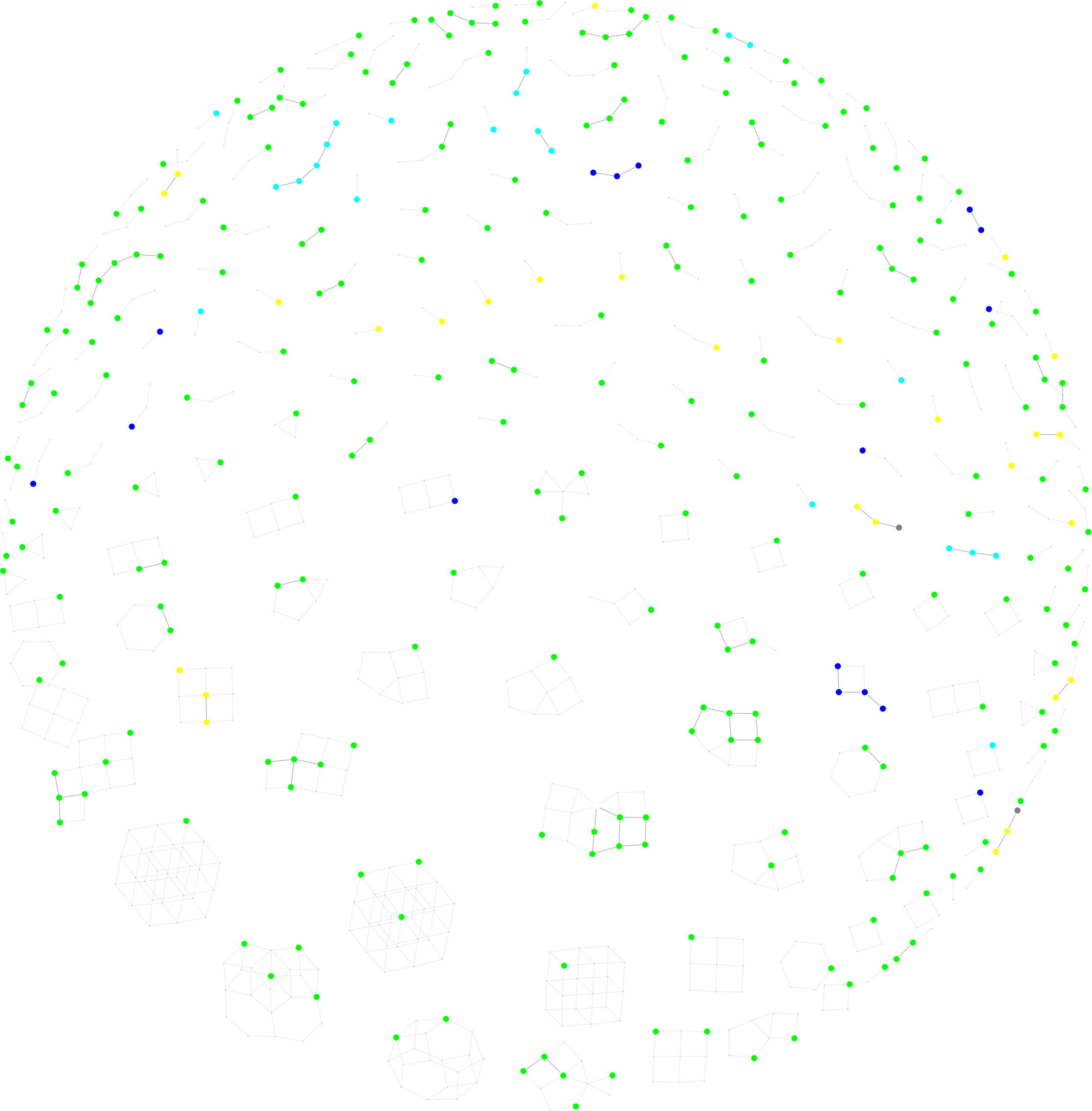

Supplement: Supplementary file 2 — Supplementary Information. [file 41598_2023_51012_MOESM2_ESM.zip › gutGH-SI/Networks/CAZY-O-glycan-networks-gut/p7675-GH-network-pp-og.jpg]

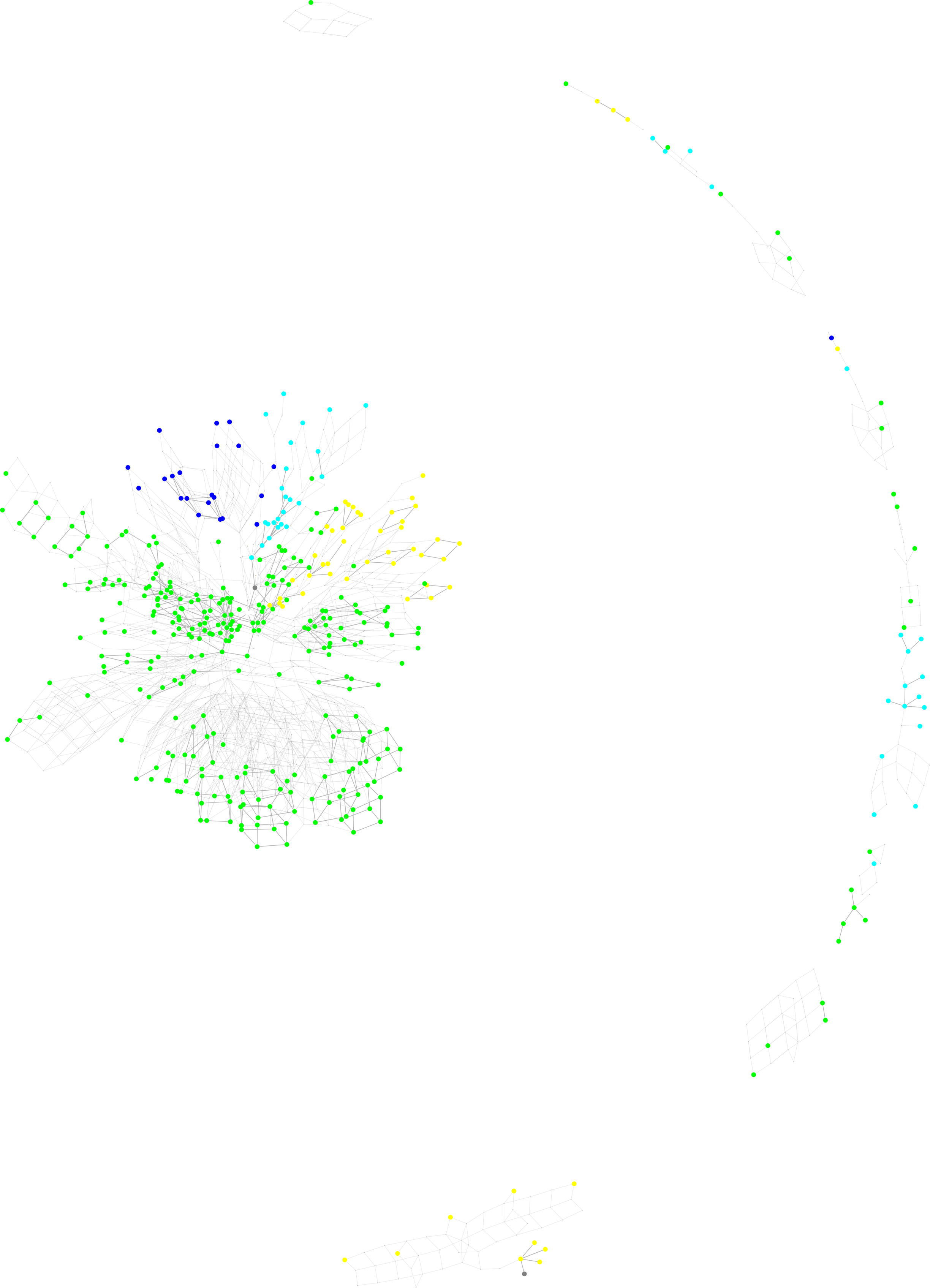

Supplement: Supplementary file 2 — Supplementary Information. [file 41598_2023_51012_MOESM2_ESM.zip › gutGH-SI/Networks/CAZY-O-glycan-networks-gut/p0-GH-network-pp-og.jpg]

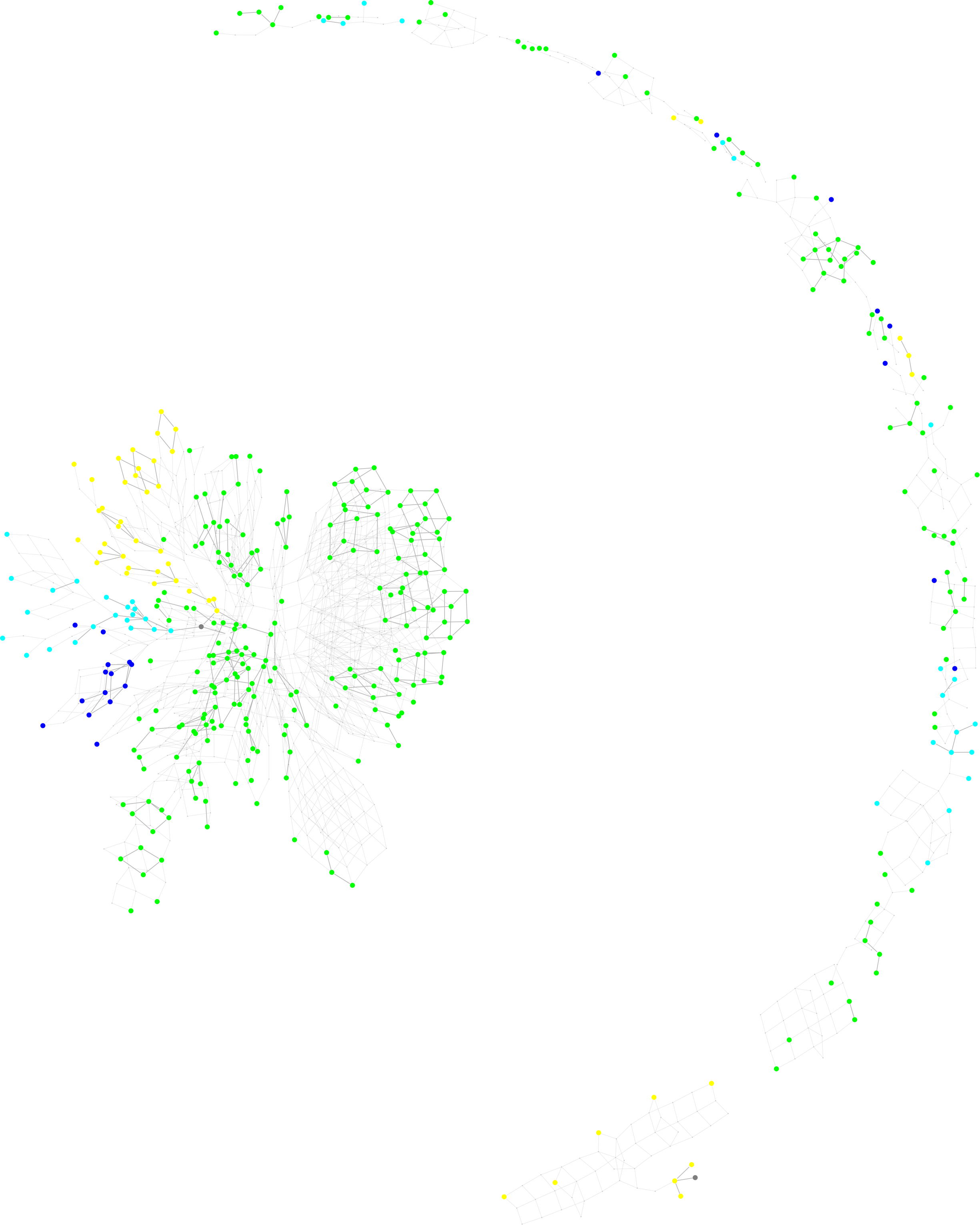

Supplement: Supplementary file 2 — Supplementary Information. [file 41598_2023_51012_MOESM2_ESM.zip › gutGH-SI/Networks/CAZY-O-glycan-networks-gut/p7954-GH-network-pp-og.jpg]

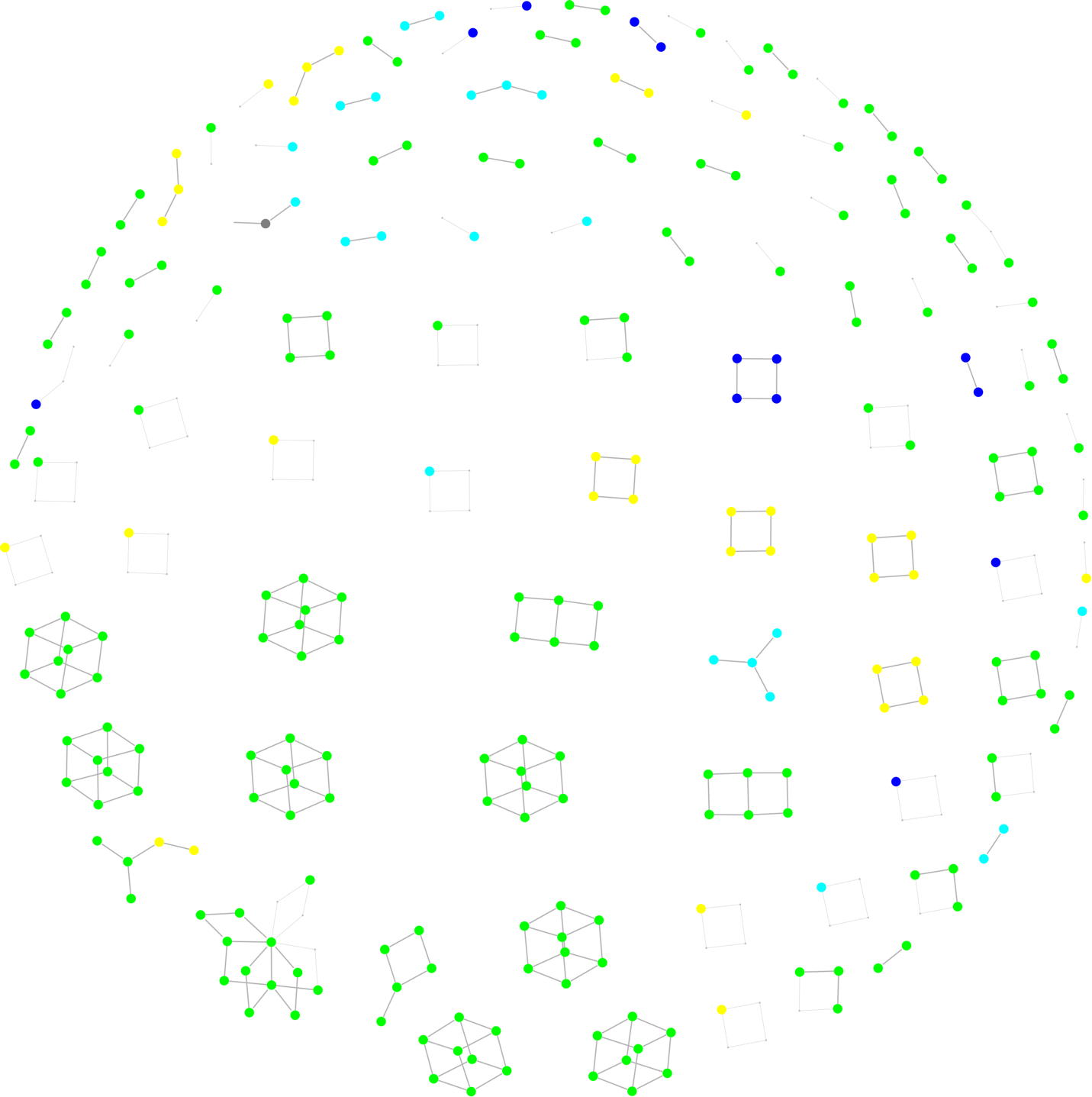

Supplement: Supplementary file 2 — Supplementary Information. [file 41598_2023_51012_MOESM2_ESM.zip › gutGH-SI/Networks/CAZY-O-glycan-networks-gut/p8126-GH-network-pp-og.jpg]

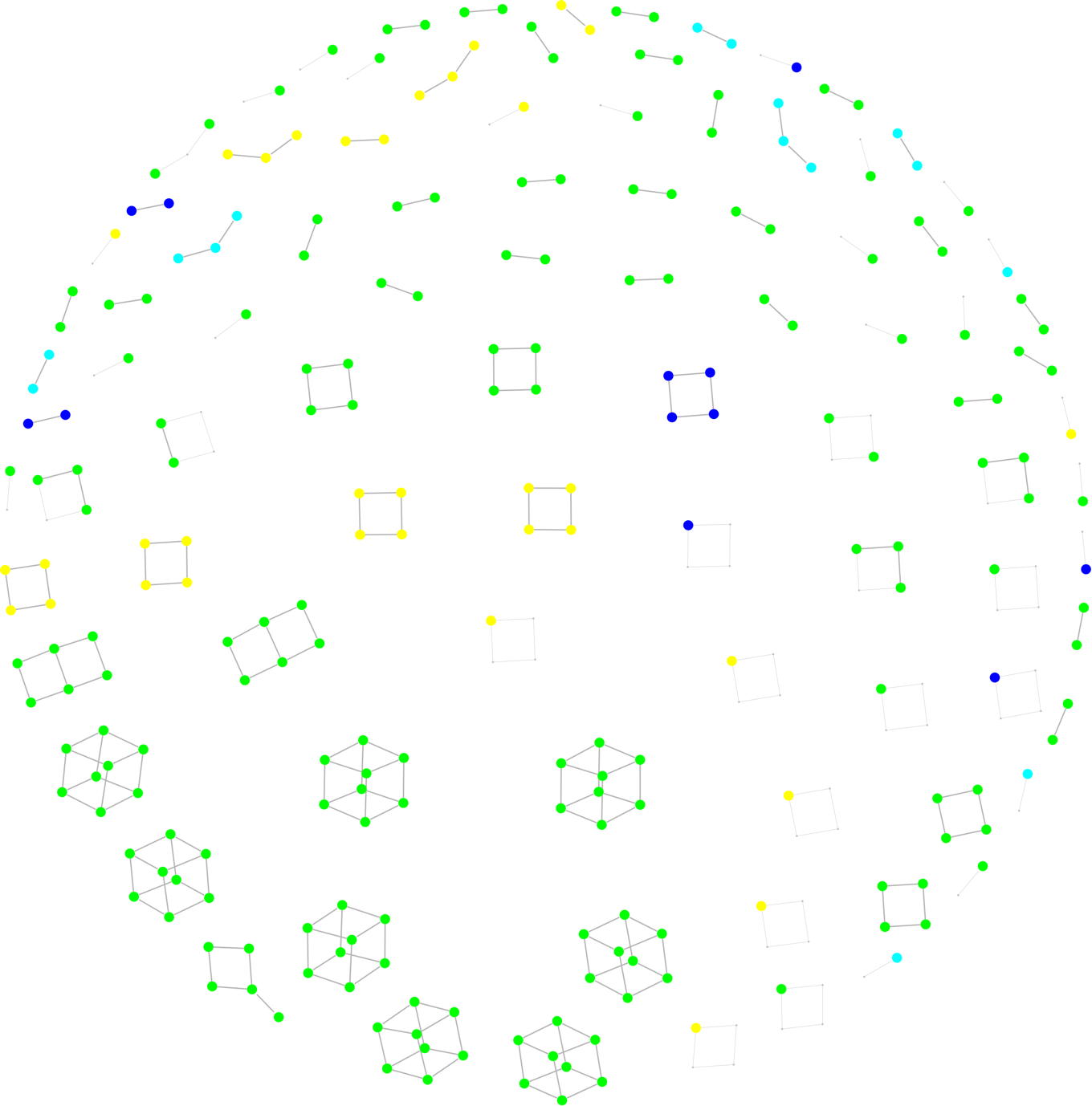

Supplement: Supplementary file 2 — Supplementary Information. [file 41598_2023_51012_MOESM2_ESM.zip › gutGH-SI/Networks/CAZY-O-glycan-networks-gut/p8191-GH-network-pp-og.jpg]

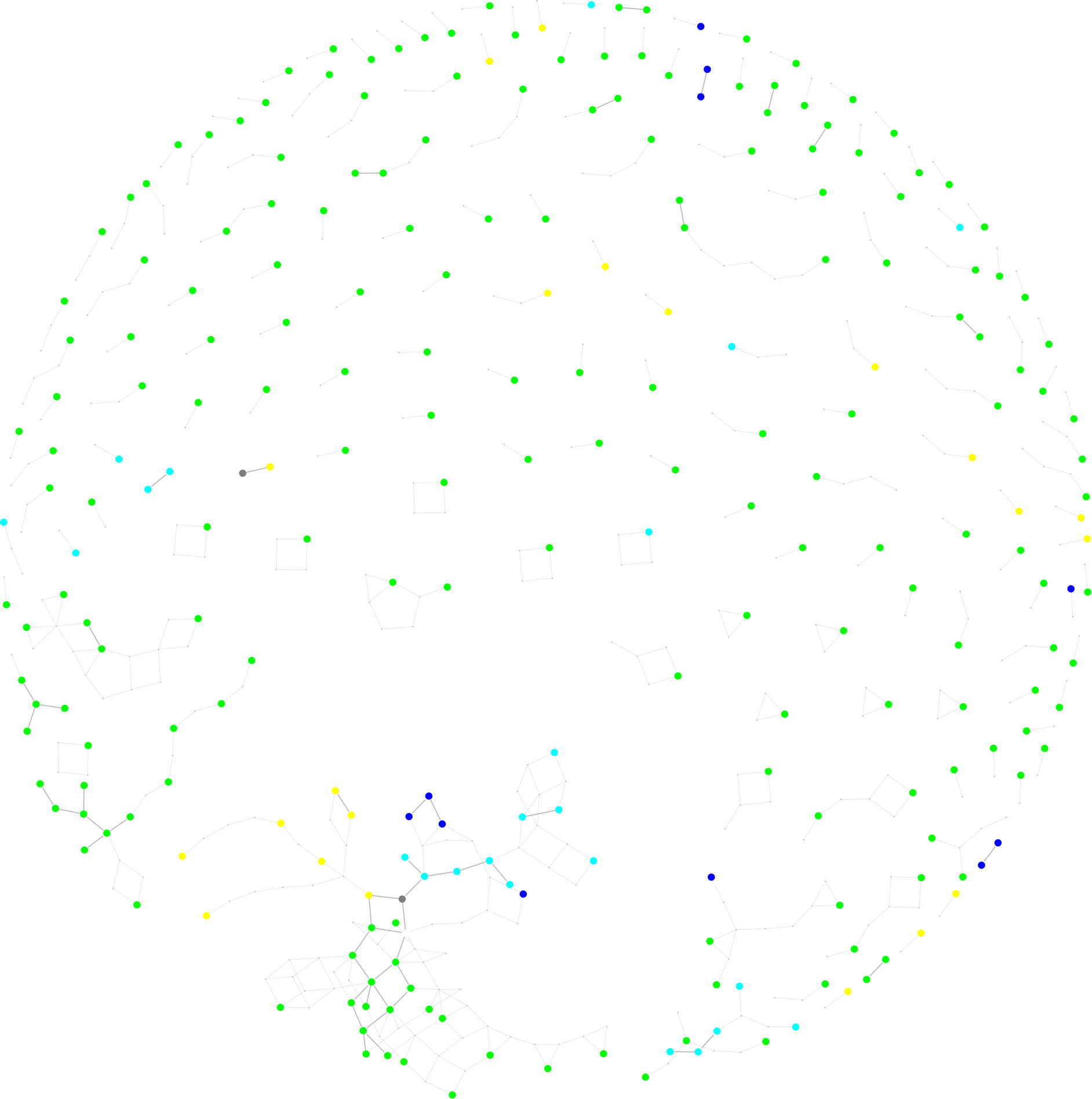

Supplement: Supplementary file 2 — Supplementary Information. [file 41598_2023_51012_MOESM2_ESM.zip › gutGH-SI/Networks/CAZY-O-glycan-networks-gut/p8123-GH-network-pp-og.jpg]

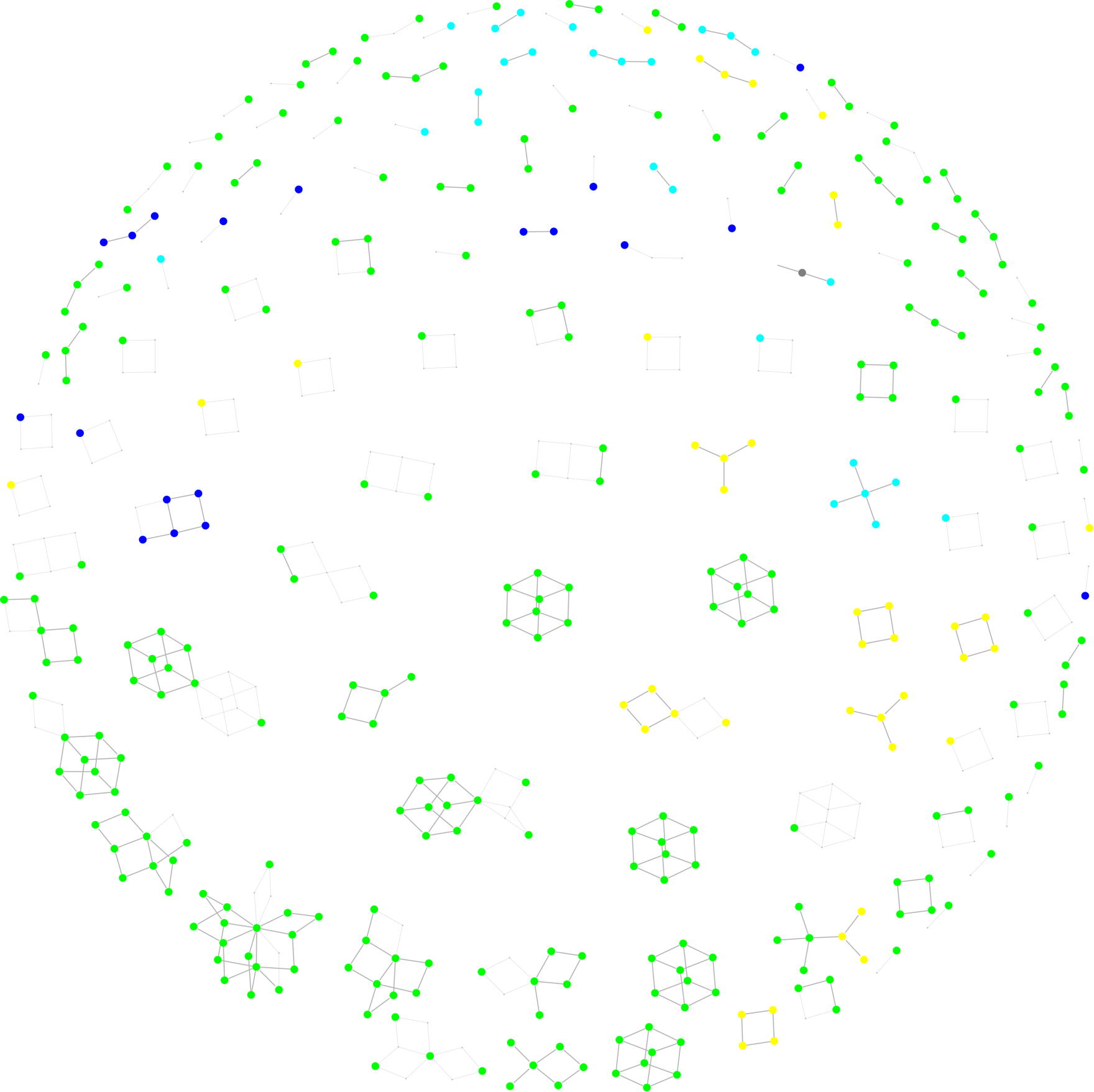

Supplement: Supplementary file 2 — Supplementary Information. [file 41598_2023_51012_MOESM2_ESM.zip › gutGH-SI/Networks/CAZY-O-glycan-networks-gut/p7135-GH-network-pp-og.jpg]

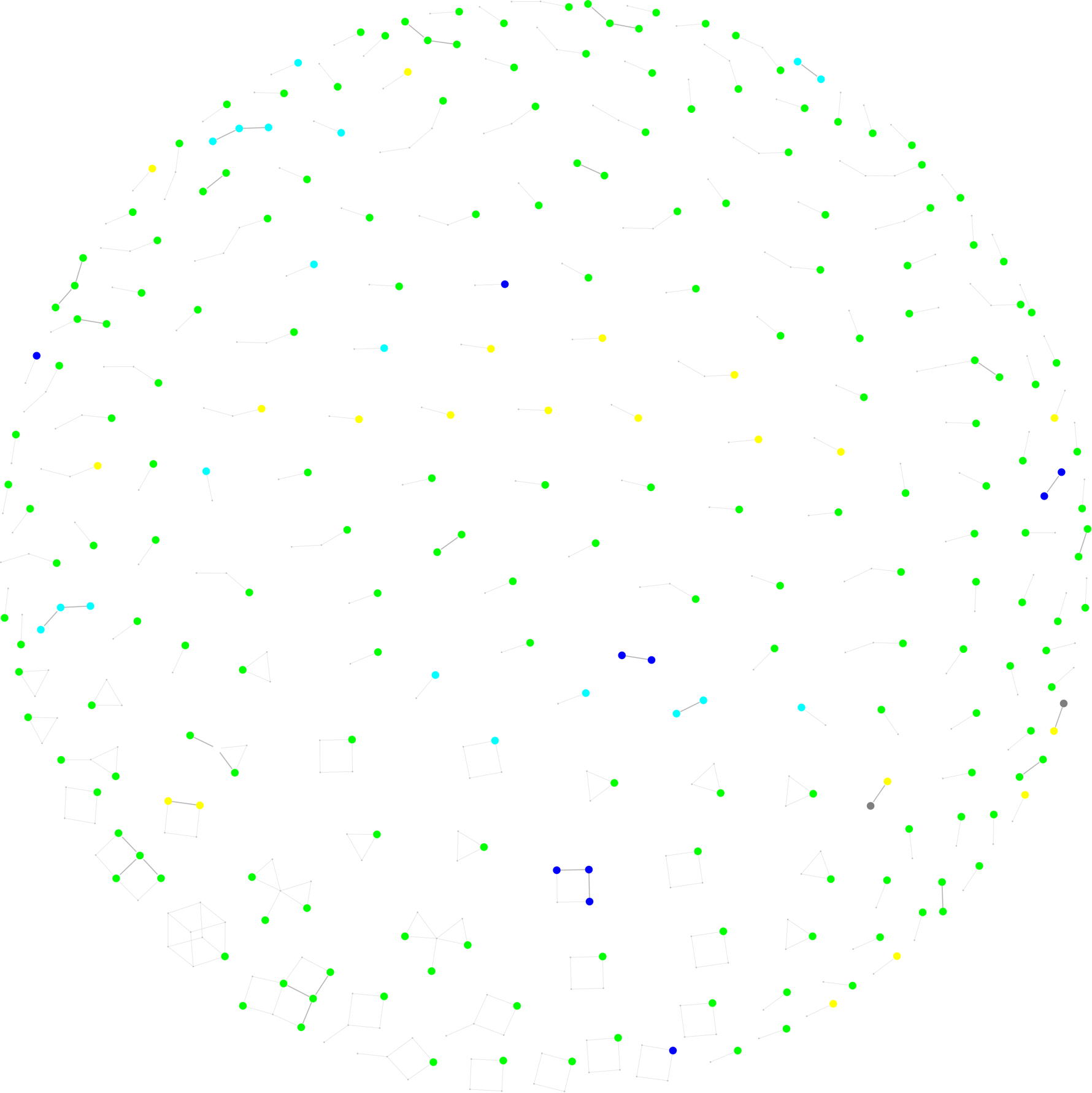

Supplement: Supplementary file 2 — Supplementary Information. [file 41598_2023_51012_MOESM2_ESM.zip › gutGH-SI/Networks/CAZY-O-glycan-networks-gut/p8187-GH-network-pp-og.jpg]

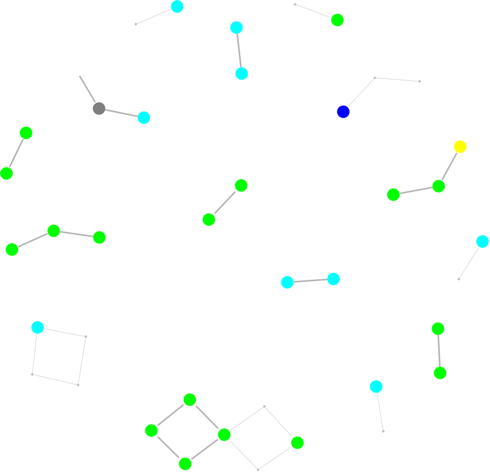

Supplement: Supplementary file 2 — Supplementary Information. [file 41598_2023_51012_MOESM2_ESM.zip › gutGH-SI/Networks/CAZY-O-glycan-networks-gut/p8127-GH-network-pp-og.jpg]

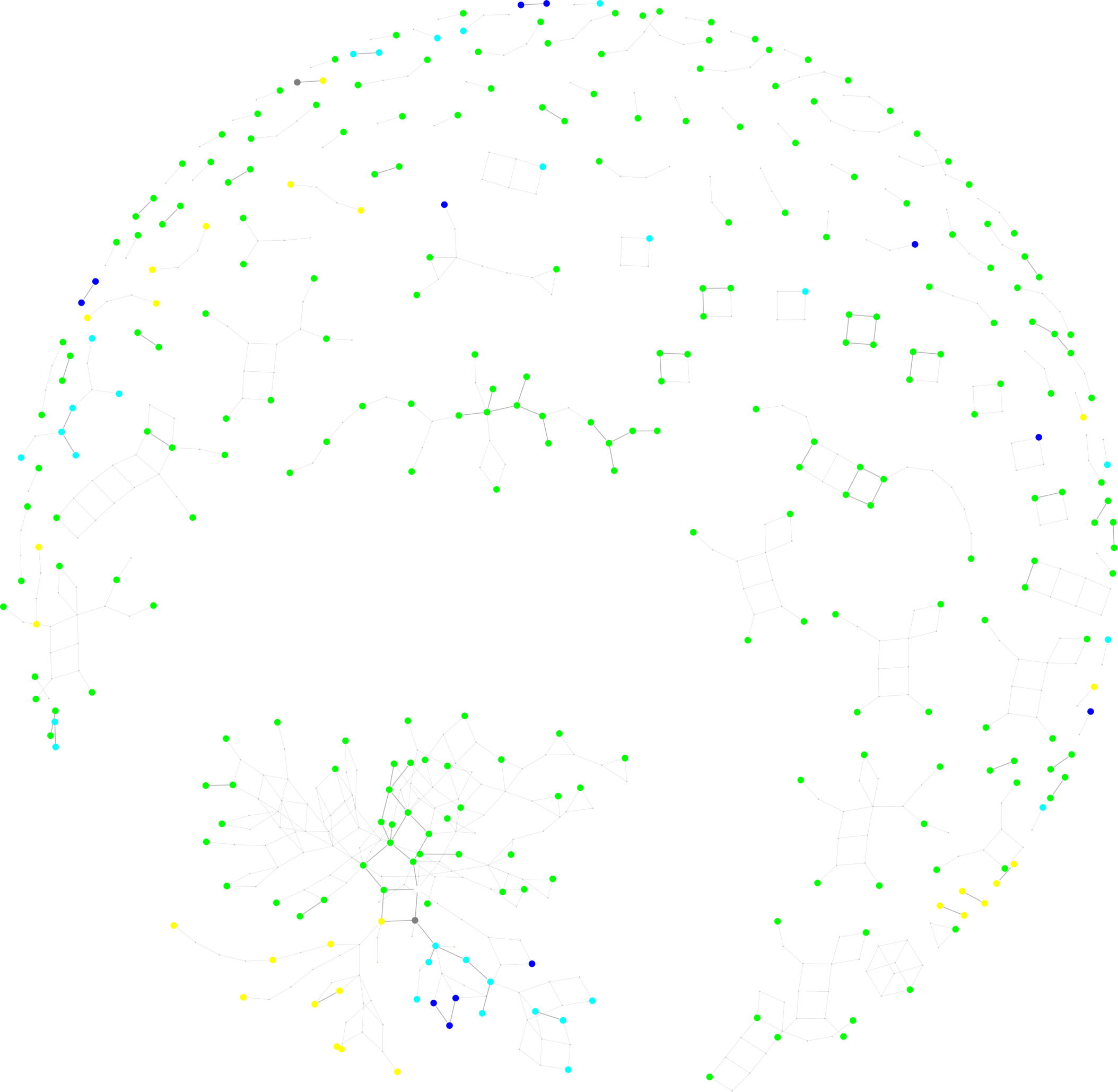

Supplement: Supplementary file 2 — Supplementary Information. [file 41598_2023_51012_MOESM2_ESM.zip › gutGH-SI/Networks/CAZY-O-glycan-networks-gut/p6963-GH-network-pp-og.jpg]

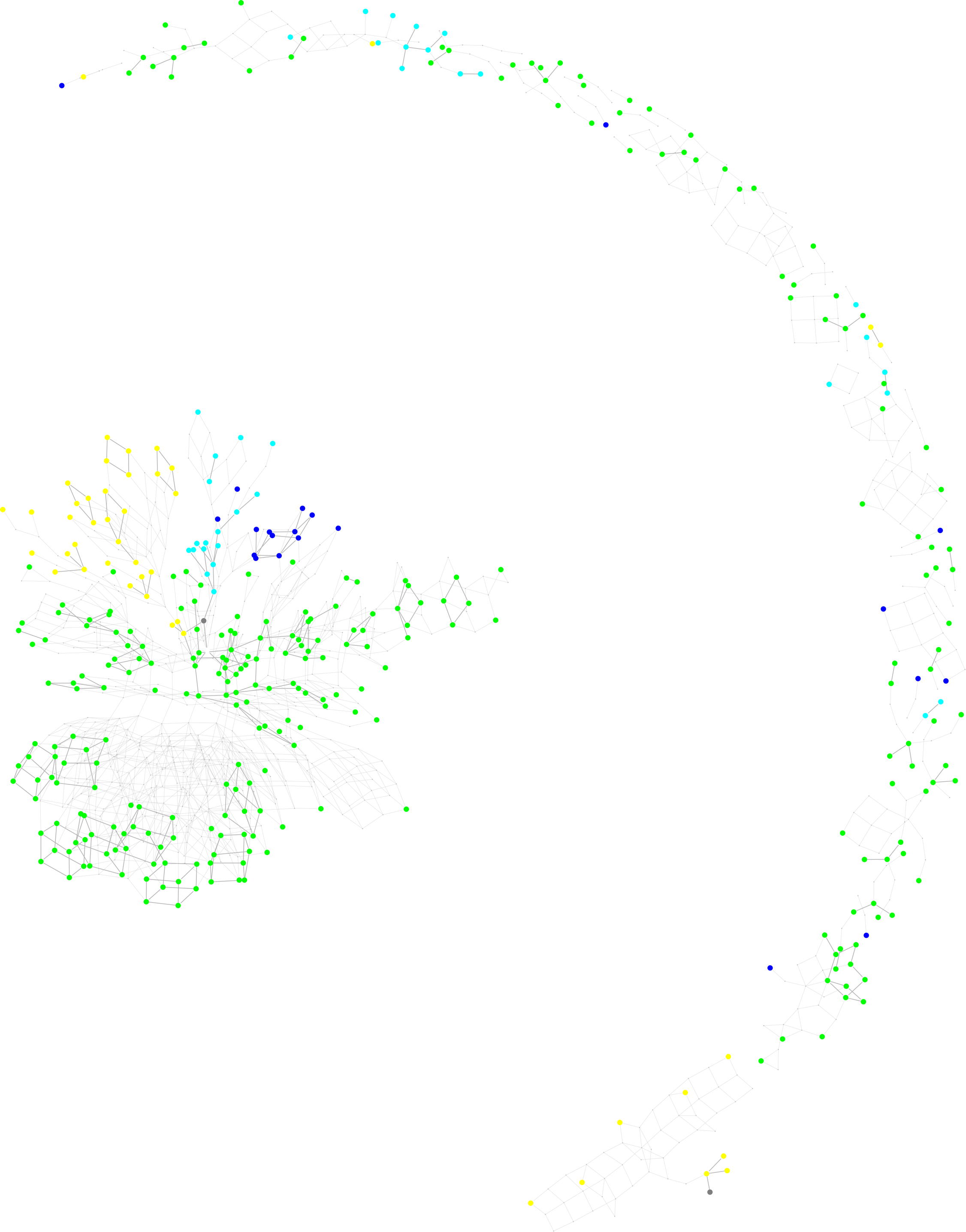

Supplement: Supplementary file 2 — Supplementary Information. [file 41598_2023_51012_MOESM2_ESM.zip › gutGH-SI/Networks/CAZY-O-glycan-networks-gut/p6586-GH-network-pp-og.jpg]

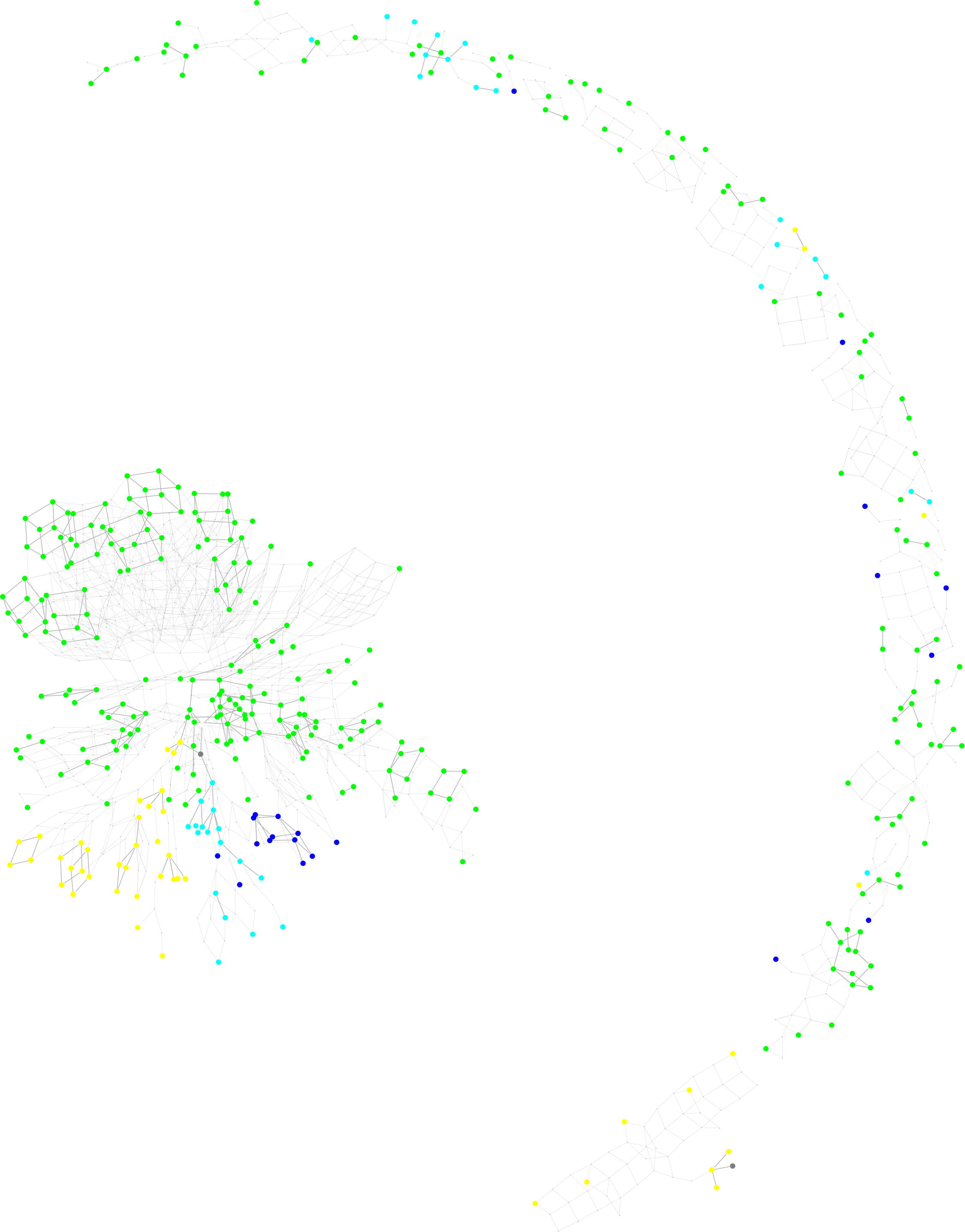

Supplement: Supplementary file 2 — Supplementary Information. [file 41598_2023_51012_MOESM2_ESM.zip › gutGH-SI/Networks/CAZY-O-glycan-networks-gut/p5530-GH-network-pp-og.jpg]

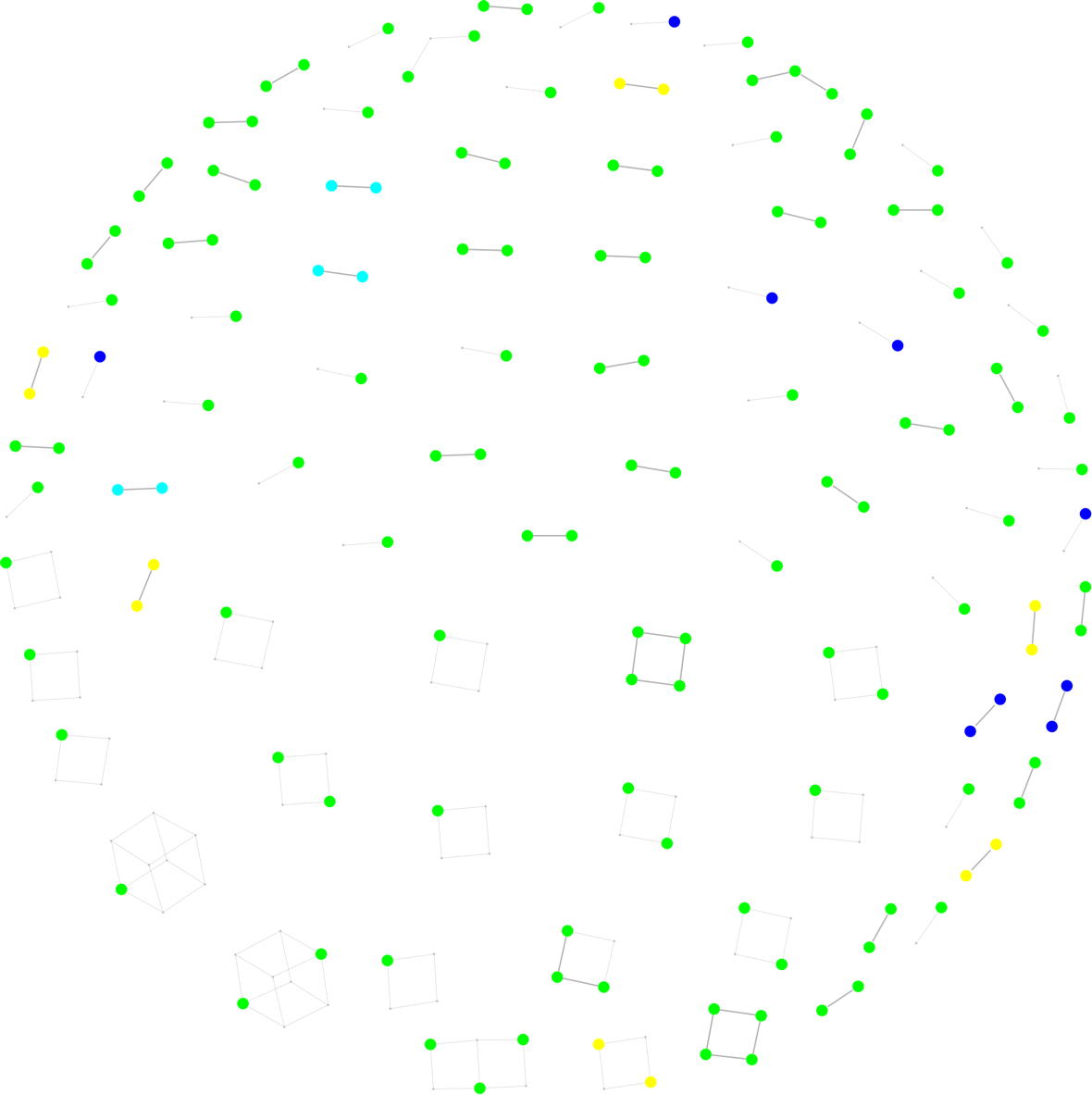

Supplement: Supplementary file 2 — Supplementary Information. [file 41598_2023_51012_MOESM2_ESM.zip › gutGH-SI/Networks/CAZY-O-glycan-networks-gut/p8159-GH-network-pp-og.jpg]

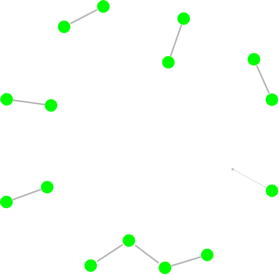

Supplement: Supplementary file 2 — Supplementary Information. [file 41598_2023_51012_MOESM2_ESM.zip › gutGH-SI/Networks/CAZY-O-glycan-networks-gut/p6143-GH-network-pp-og.jpg]

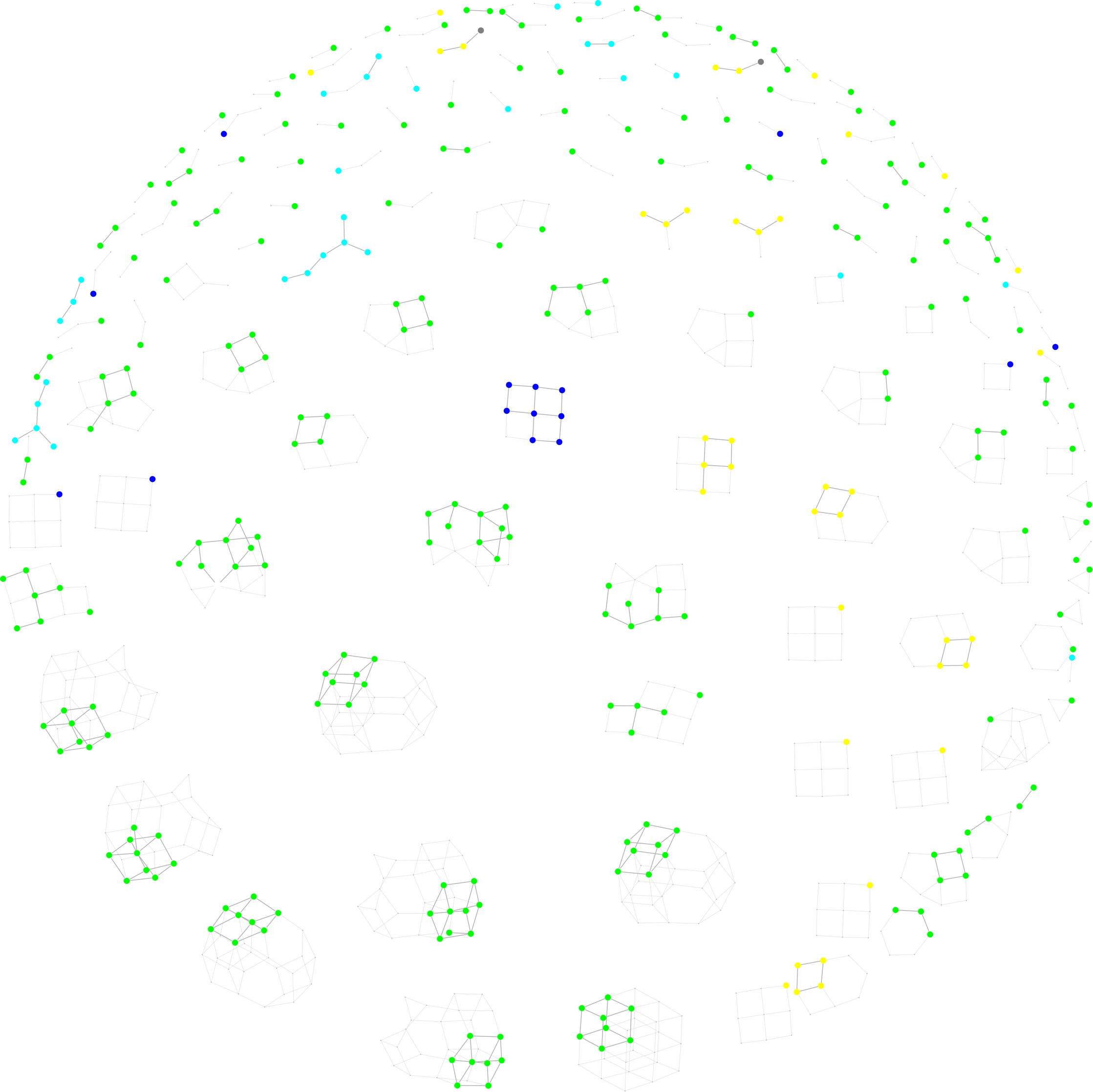

Supplement: Supplementary file 2 — Supplementary Information. [file 41598_2023_51012_MOESM2_ESM.zip › gutGH-SI/Networks/UniProt-O-glycan-networks-gut/p8186-GH-network-pp-og.jpg]

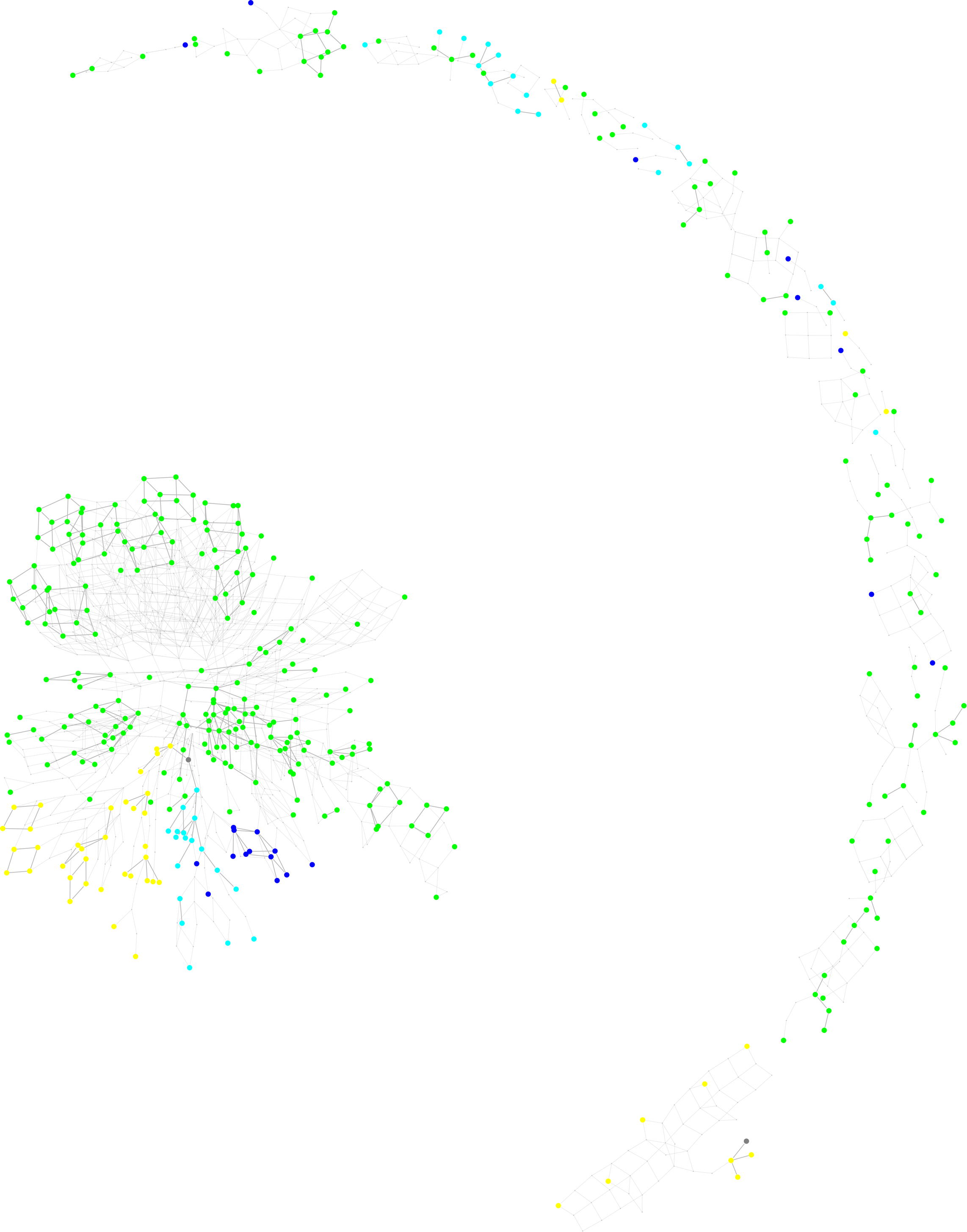

Supplement: Supplementary file 2 — Supplementary Information. [file 41598_2023_51012_MOESM2_ESM.zip › gutGH-SI/Networks/UniProt-O-glycan-networks-gut/p4888-GH-network-pp-og.jpg]

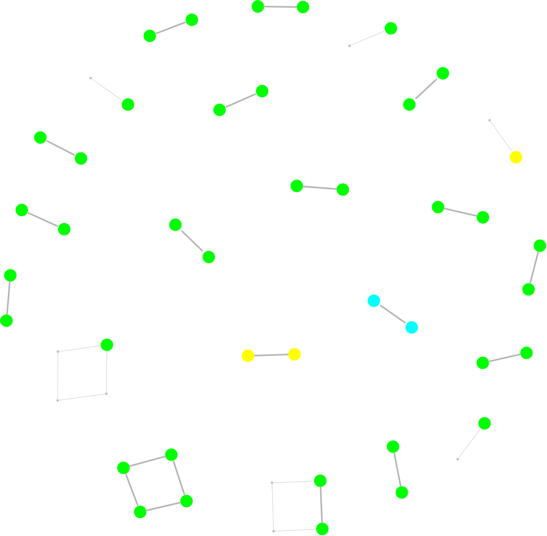

Supplement: Supplementary file 2 — Supplementary Information. [file 41598_2023_51012_MOESM2_ESM.zip › gutGH-SI/Networks/UniProt-O-glycan-networks-gut/p8189-GH-network-pp-og.jpg]

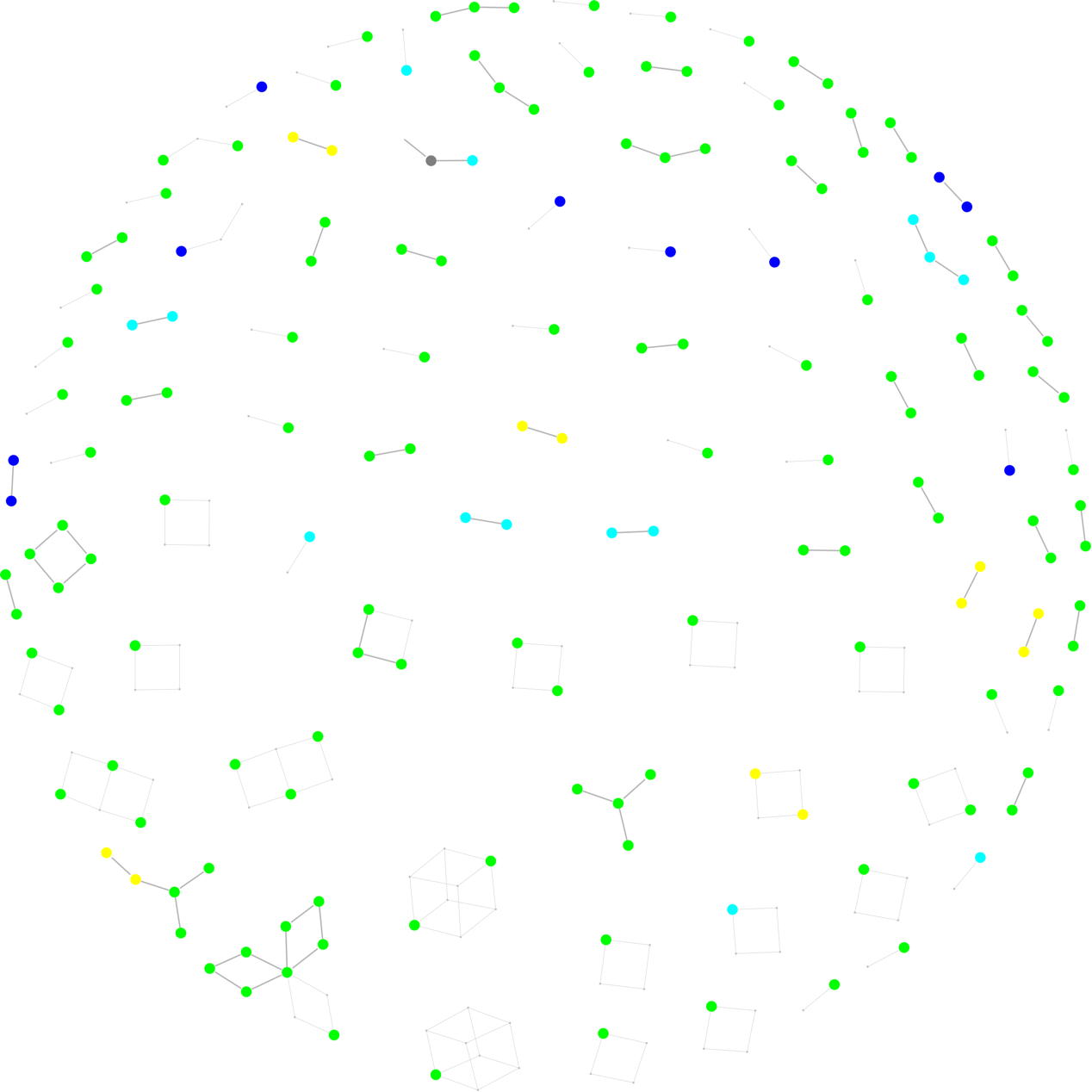

Supplement: Supplementary file 2 — Supplementary Information. [file 41598_2023_51012_MOESM2_ESM.zip › gutGH-SI/Networks/UniProt-O-glycan-networks-gut/p8095-GH-network-pp-og.jpg]

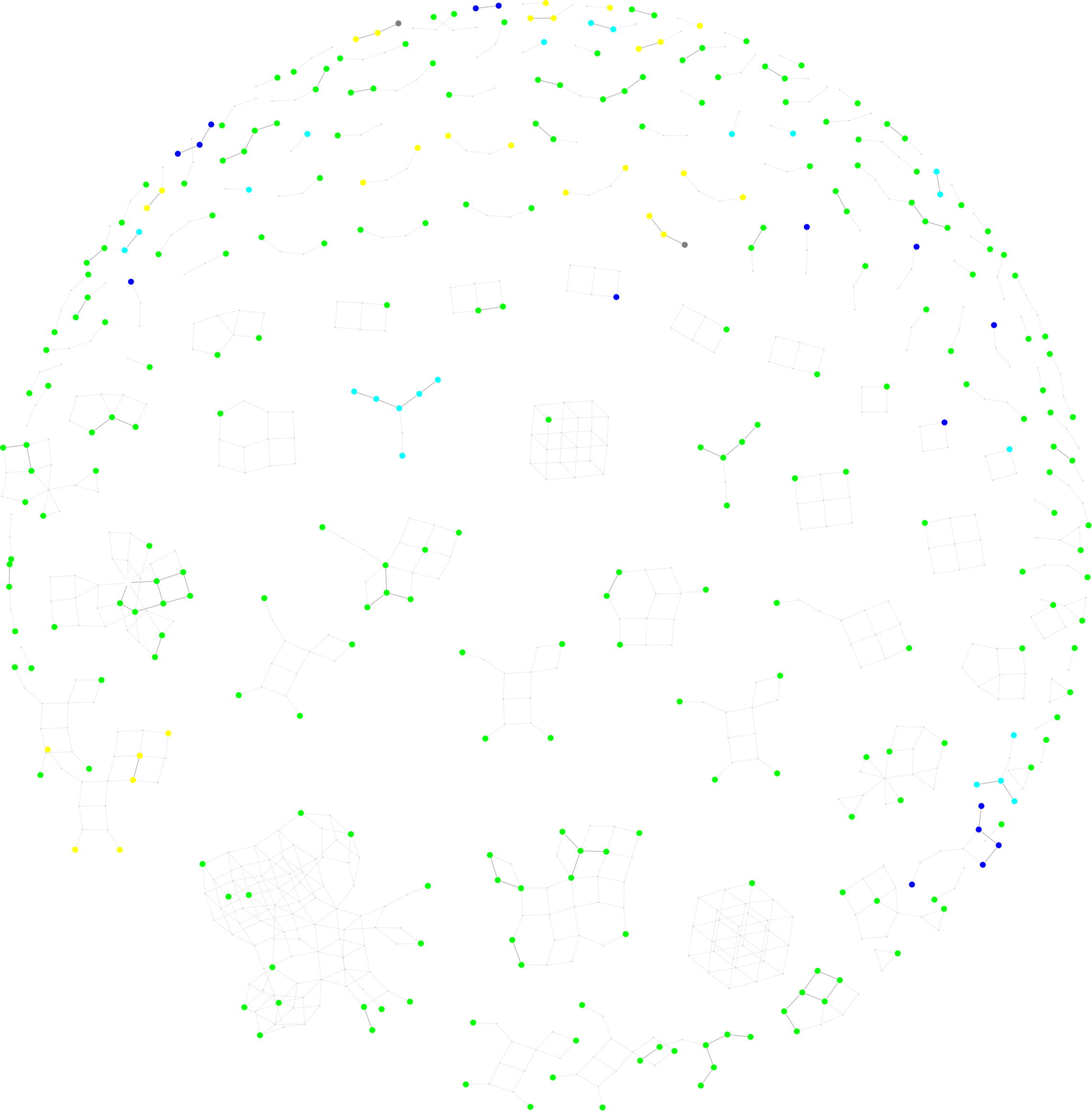

Supplement: Supplementary file 2 — Supplementary Information. [file 41598_2023_51012_MOESM2_ESM.zip › gutGH-SI/Networks/UniProt-O-glycan-networks-gut/p8155-GH-network-pp-og.jpg]

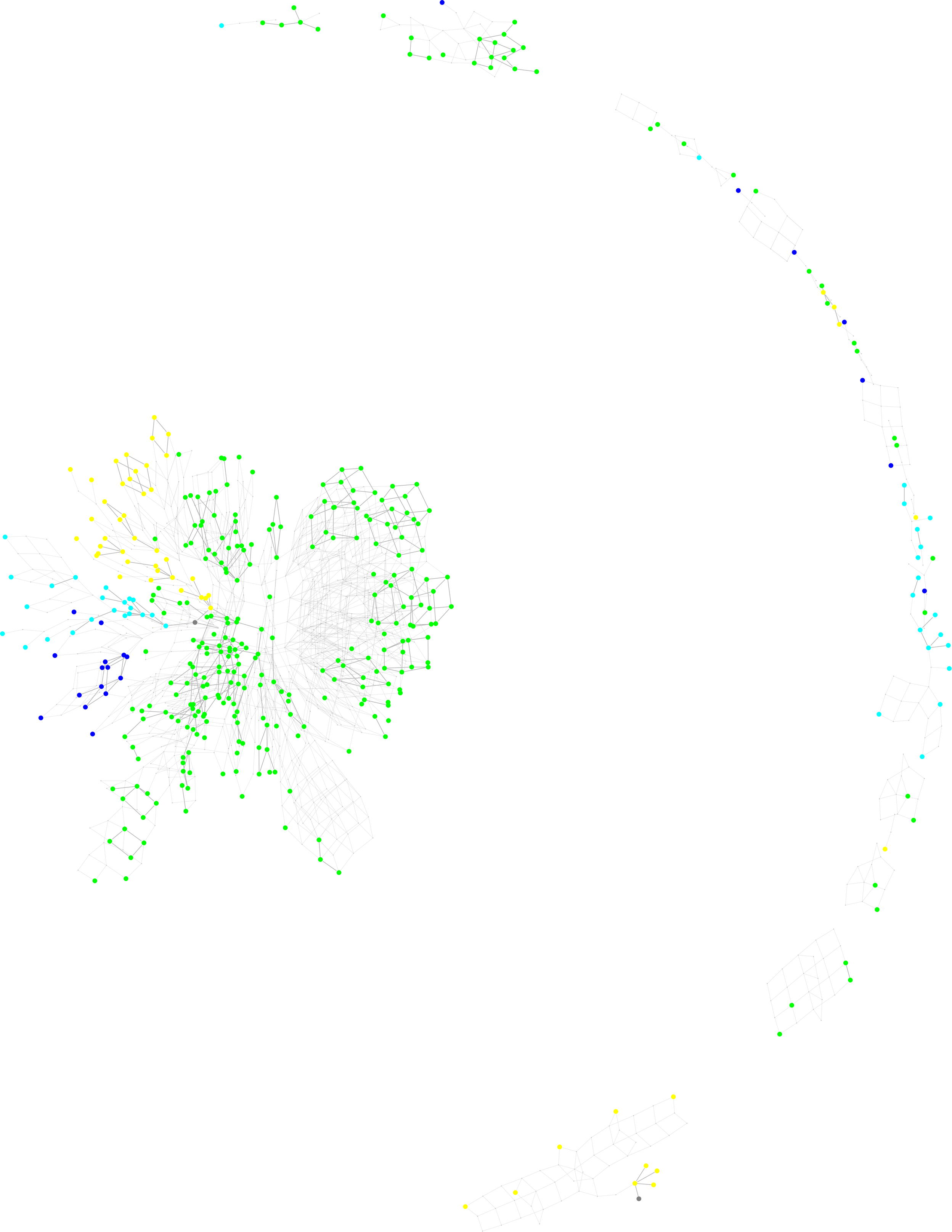

Supplement: Supplementary file 2 — Supplementary Information. [file 41598_2023_51012_MOESM2_ESM.zip › gutGH-SI/Networks/UniProt-O-glycan-networks-gut/p6912-GH-network-pp-og.jpg]

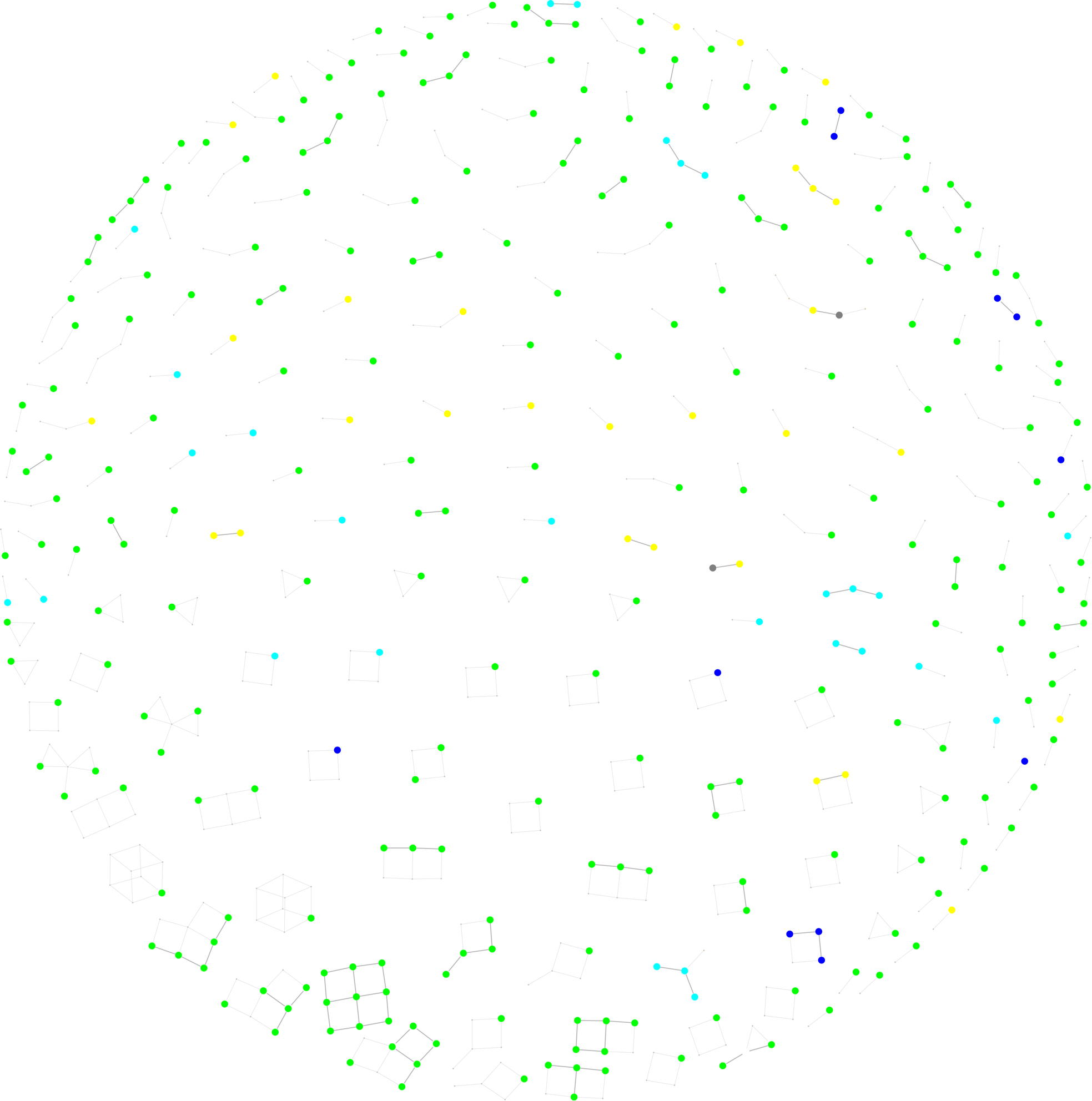

Supplement: Supplementary file 2 — Supplementary Information. [file 41598_2023_51012_MOESM2_ESM.zip › gutGH-SI/Networks/UniProt-O-glycan-networks-gut/p8049-GH-network-pp-og.jpg]

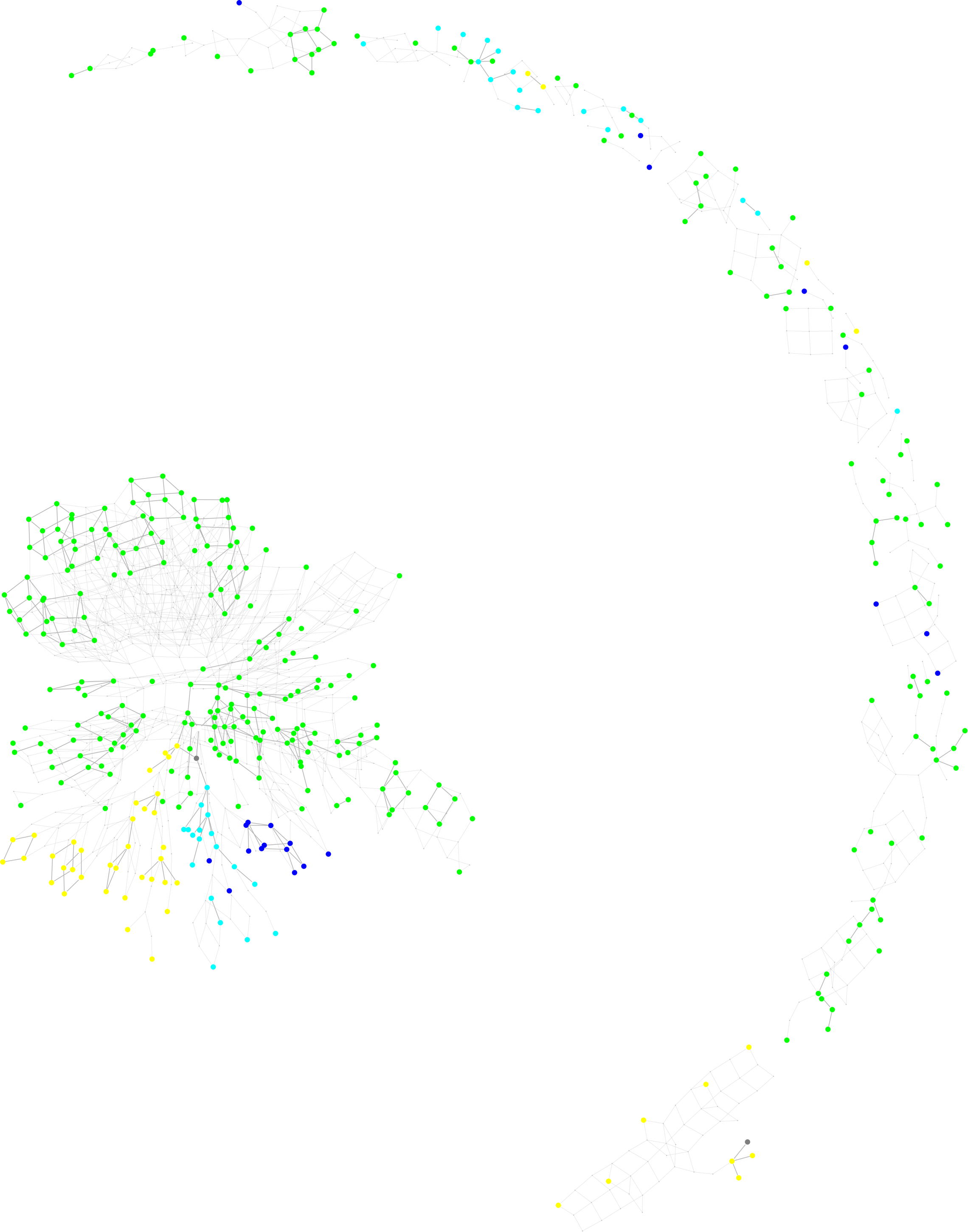

Supplement: Supplementary file 2 — Supplementary Information. [file 41598_2023_51012_MOESM2_ESM.zip › gutGH-SI/Networks/UniProt-O-glycan-networks-gut/p8088-GH-network-pp-og.jpg]

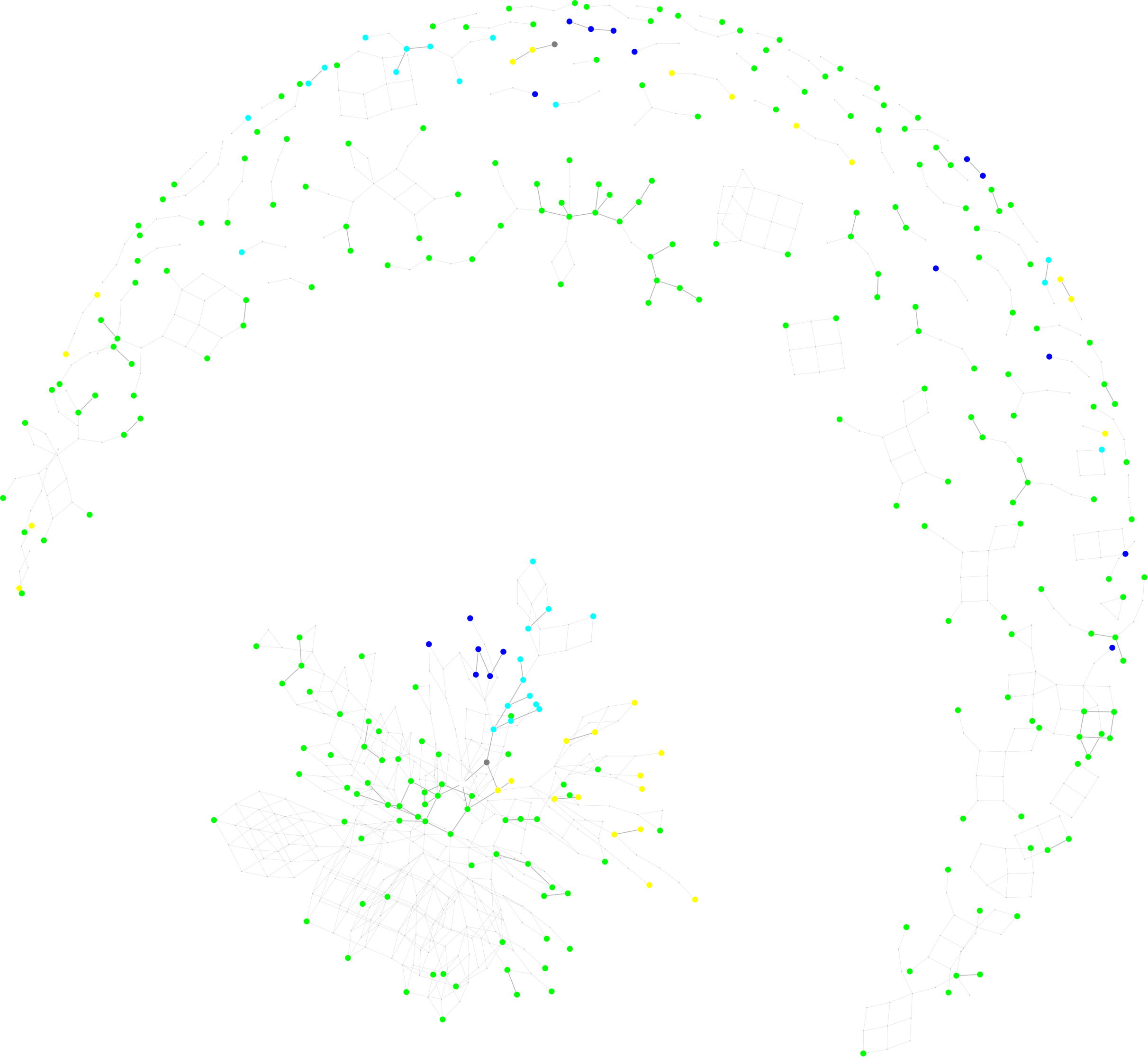

Supplement: Supplementary file 2 — Supplementary Information. [file 41598_2023_51012_MOESM2_ESM.zip › gutGH-SI/Networks/UniProt-O-glycan-networks-gut/p8091-GH-network-pp-og.jpg]

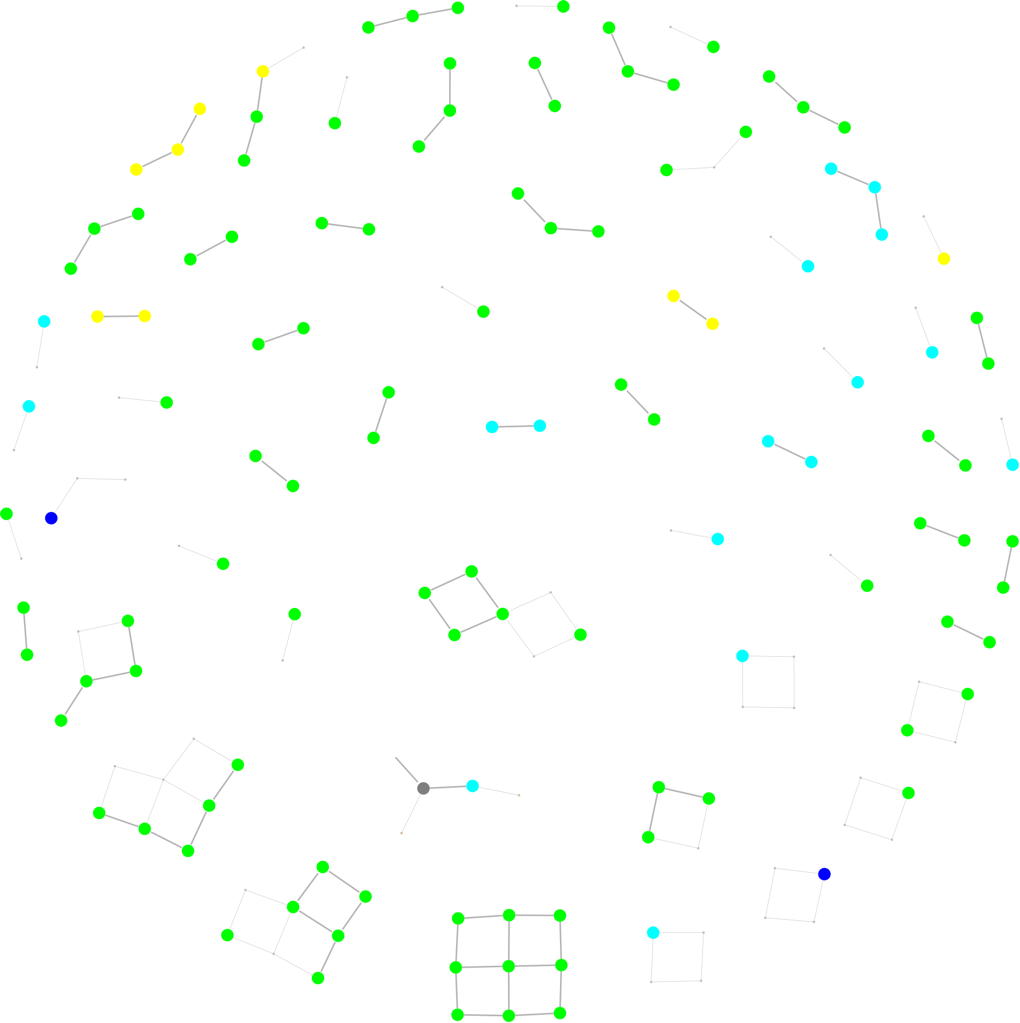

Supplement: Supplementary file 2 — Supplementary Information. [file 41598_2023_51012_MOESM2_ESM.zip › gutGH-SI/Networks/UniProt-O-glycan-networks-gut/p7989-GH-network-pp-og.jpg]

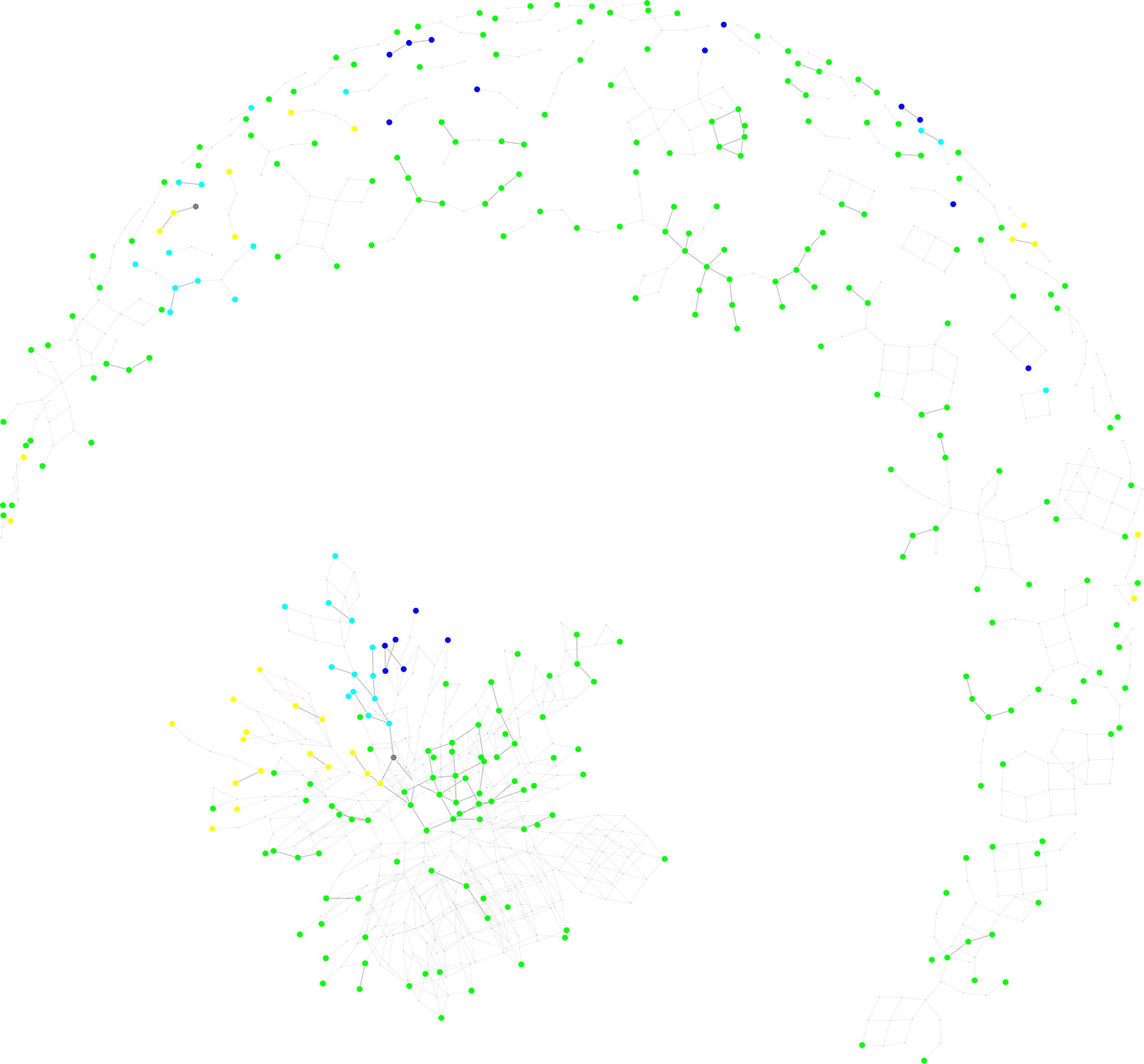

Supplement: Supplementary file 2 — Supplementary Information. [file 41598_2023_51012_MOESM2_ESM.zip › gutGH-SI/Networks/UniProt-O-glycan-networks-gut/p8089-GH-network-pp-og.jpg]

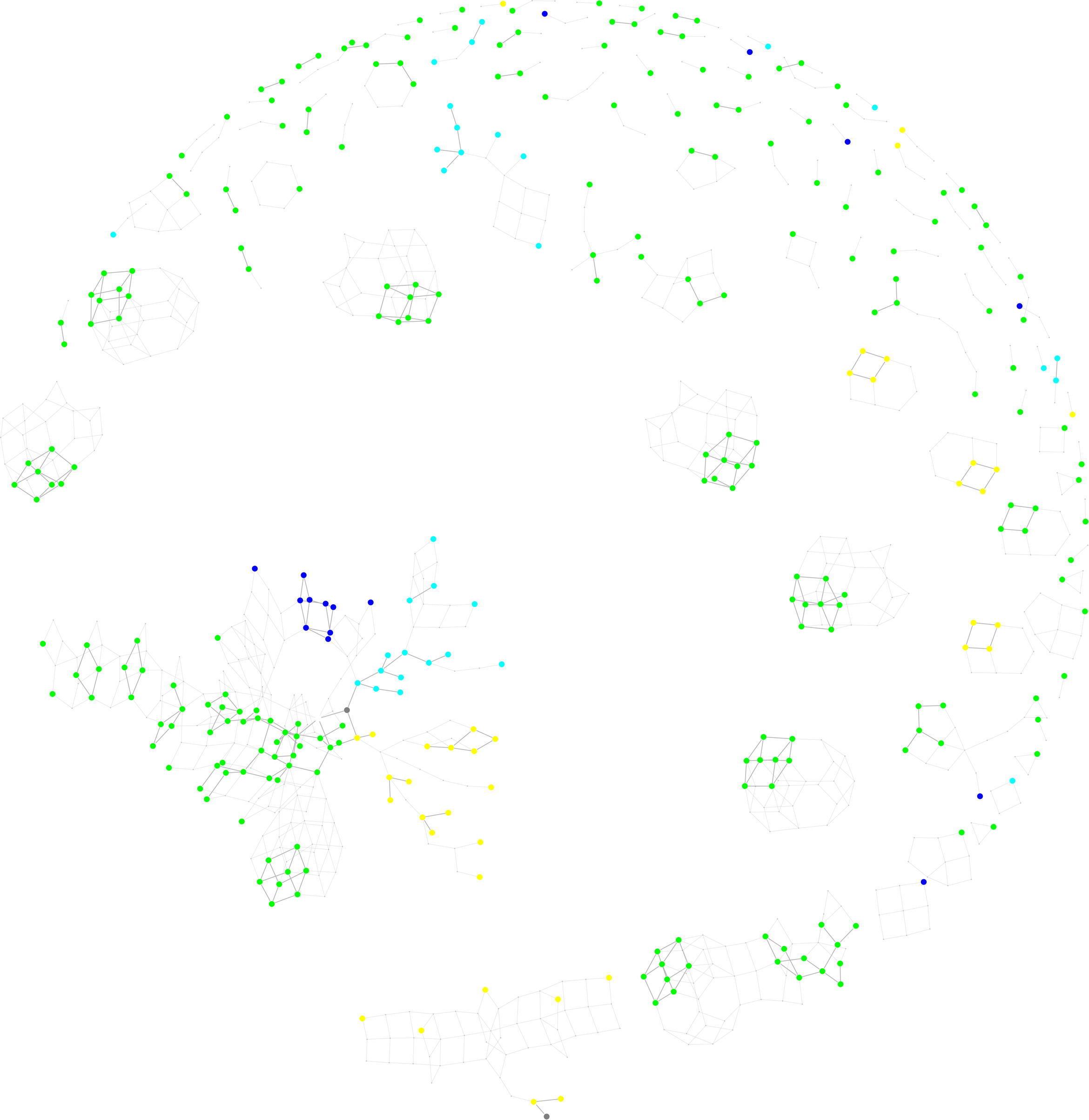

Supplement: Supplementary file 2 — Supplementary Information. [file 41598_2023_51012_MOESM2_ESM.zip › gutGH-SI/Networks/UniProt-O-glycan-networks-gut/p8122-GH-network-pp-og.jpg]

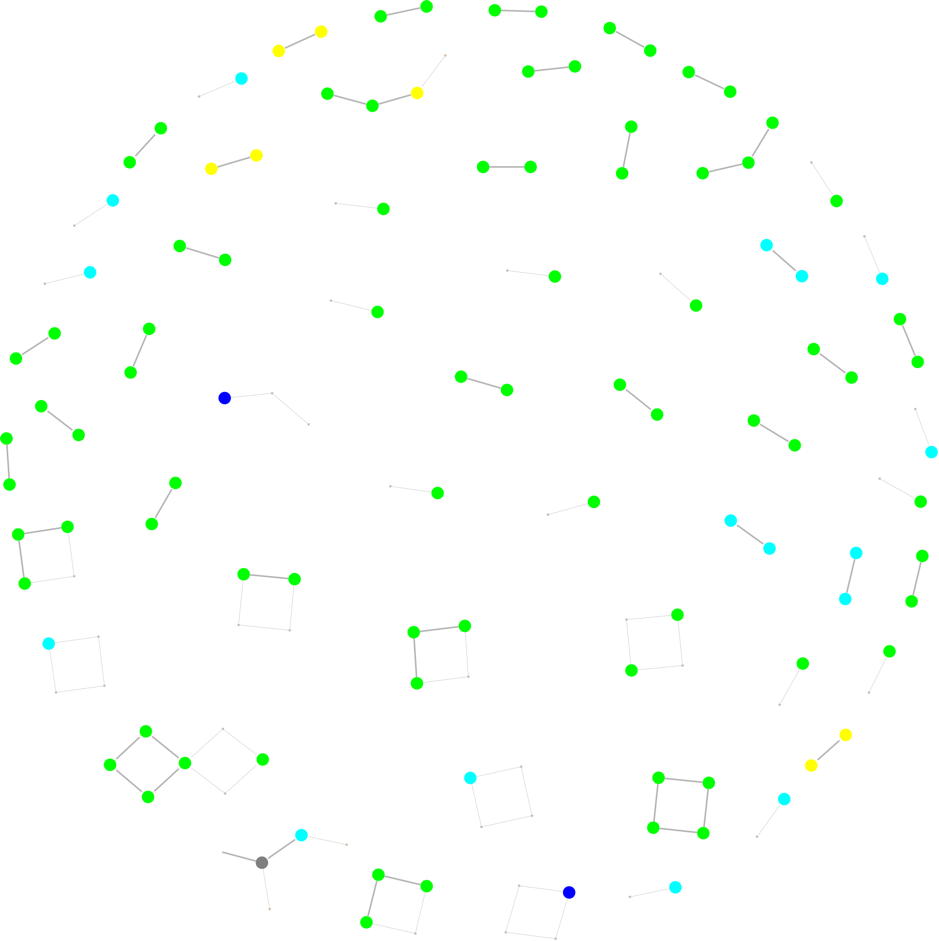

Supplement: Supplementary file 2 — Supplementary Information. [file 41598_2023_51012_MOESM2_ESM.zip › gutGH-SI/Networks/UniProt-O-glycan-networks-gut/p7991-GH-network-pp-og.jpg]

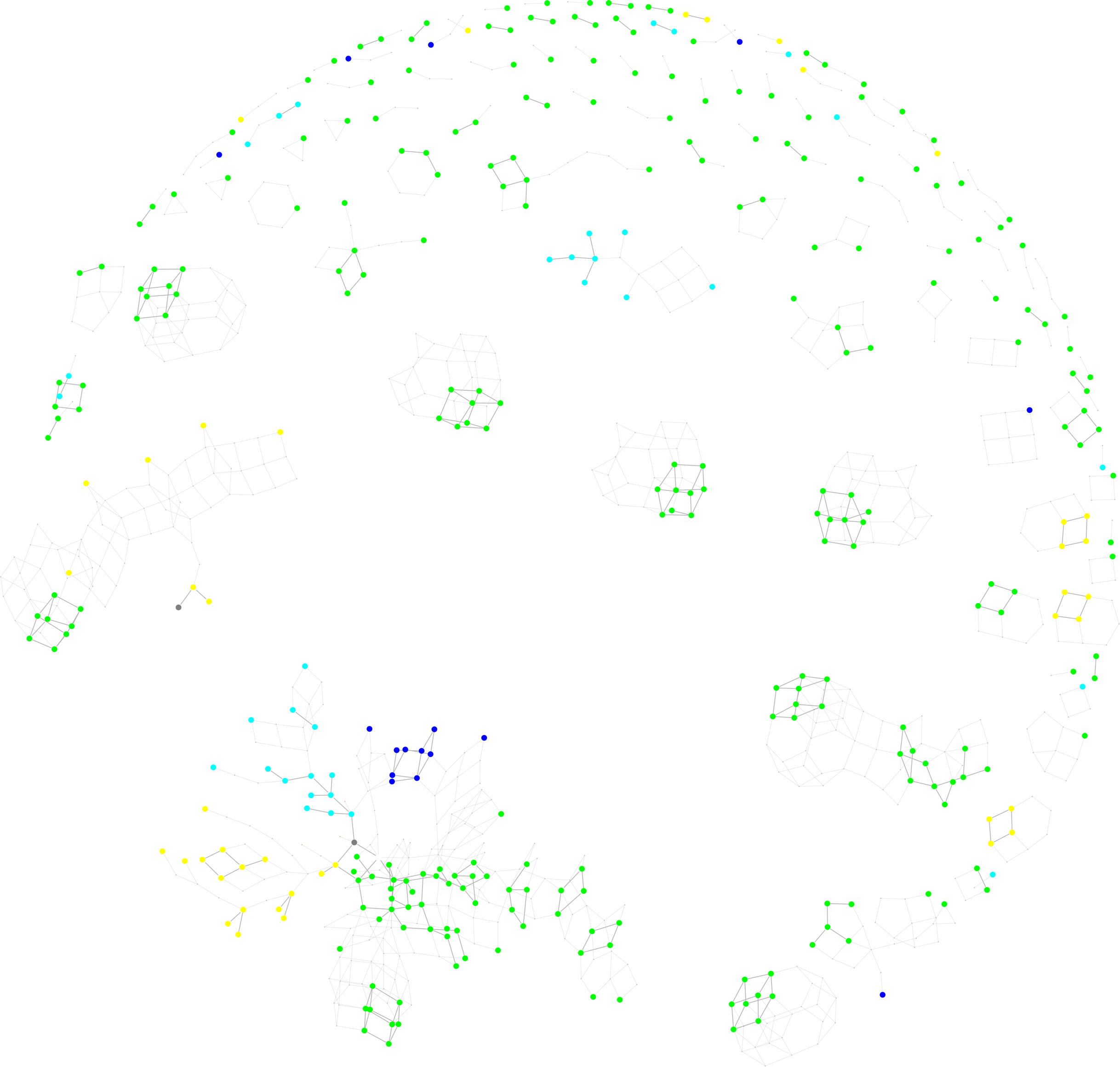

Supplement: Supplementary file 2 — Supplementary Information. [file 41598_2023_51012_MOESM2_ESM.zip › gutGH-SI/Networks/UniProt-O-glycan-networks-gut/p7992-GH-network-pp-og.jpg]

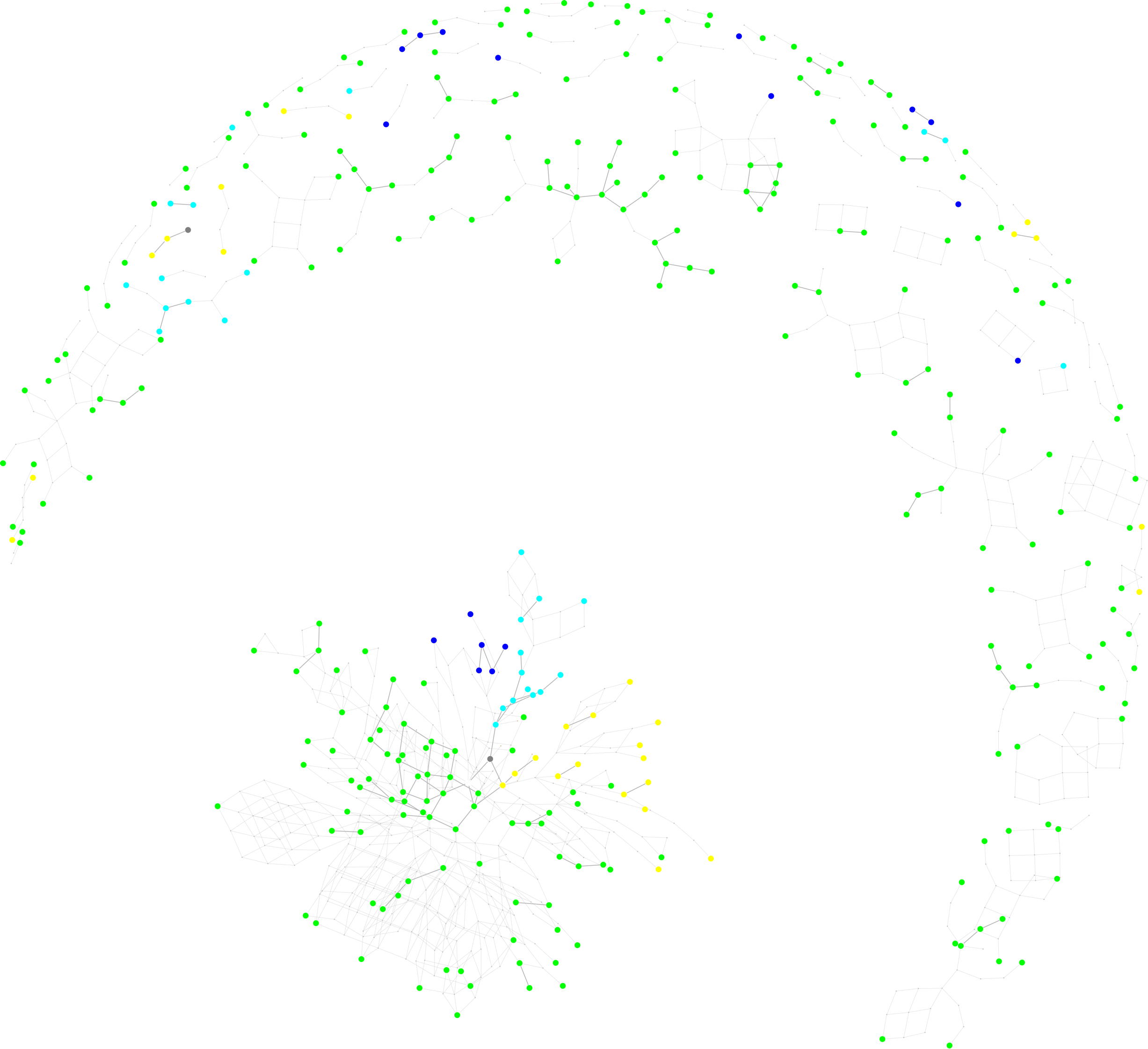

Supplement: Supplementary file 2 — Supplementary Information. [file 41598_2023_51012_MOESM2_ESM.zip › gutGH-SI/Networks/UniProt-O-glycan-networks-gut/p7961-GH-network-pp-og.jpg]

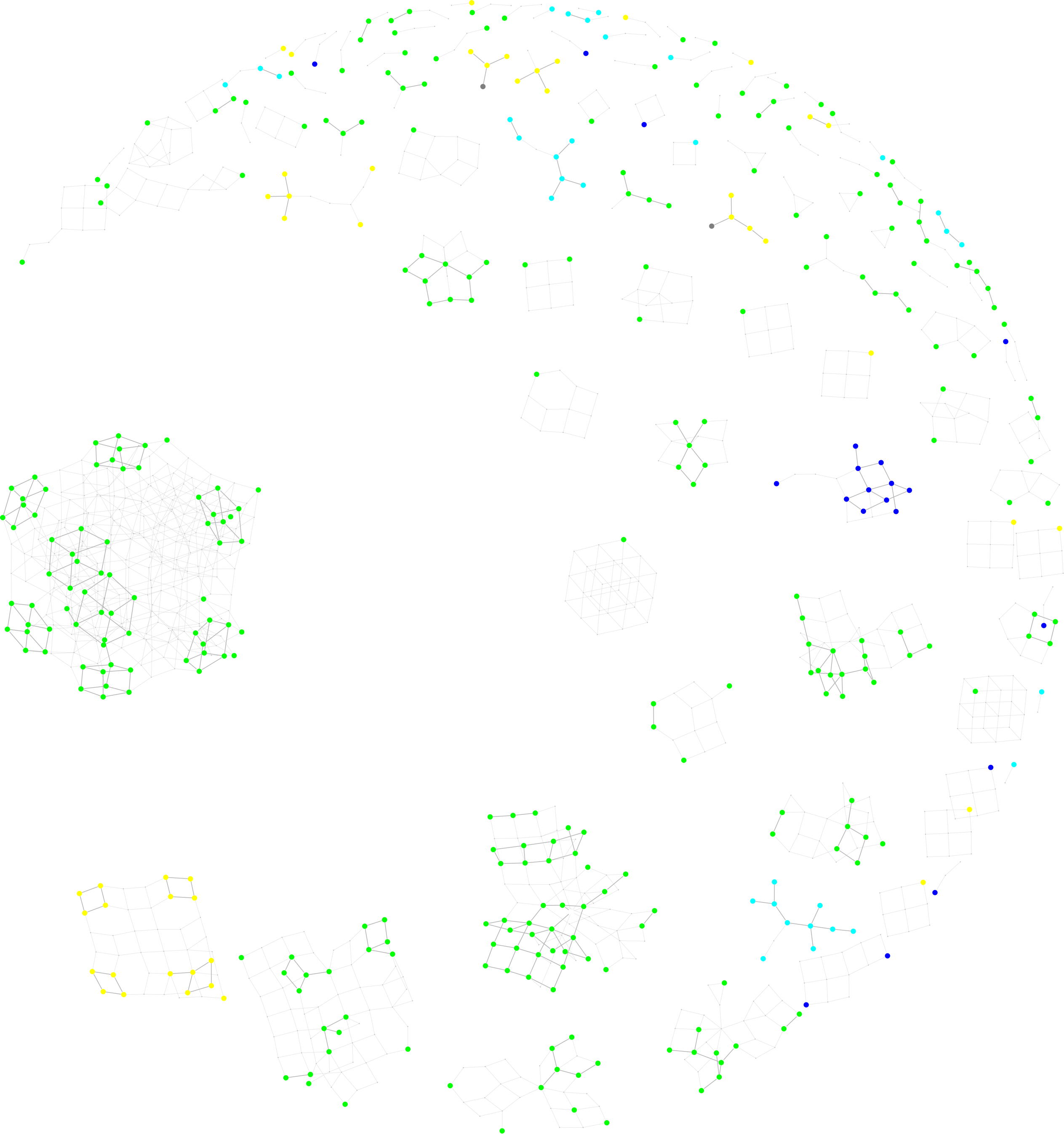

Supplement: Supplementary file 2 — Supplementary Information. [file 41598_2023_51012_MOESM2_ESM.zip › gutGH-SI/Networks/UniProt-O-glycan-networks-gut/p8152-GH-network-pp-og.jpg]

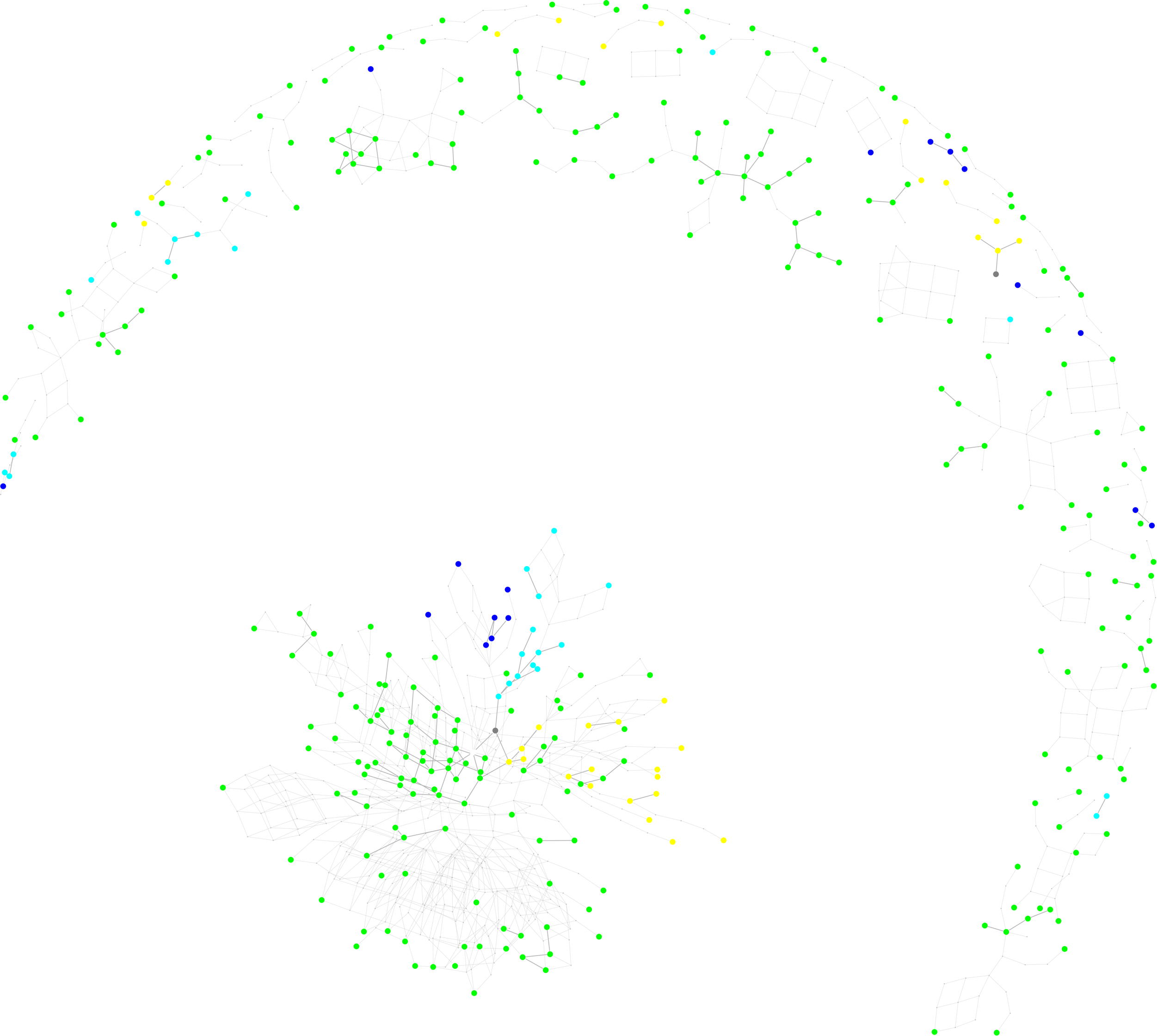

Supplement: Supplementary file 2 — Supplementary Information. [file 41598_2023_51012_MOESM2_ESM.zip › gutGH-SI/Networks/UniProt-O-glycan-networks-gut/p8073-GH-network-pp-og.jpg]

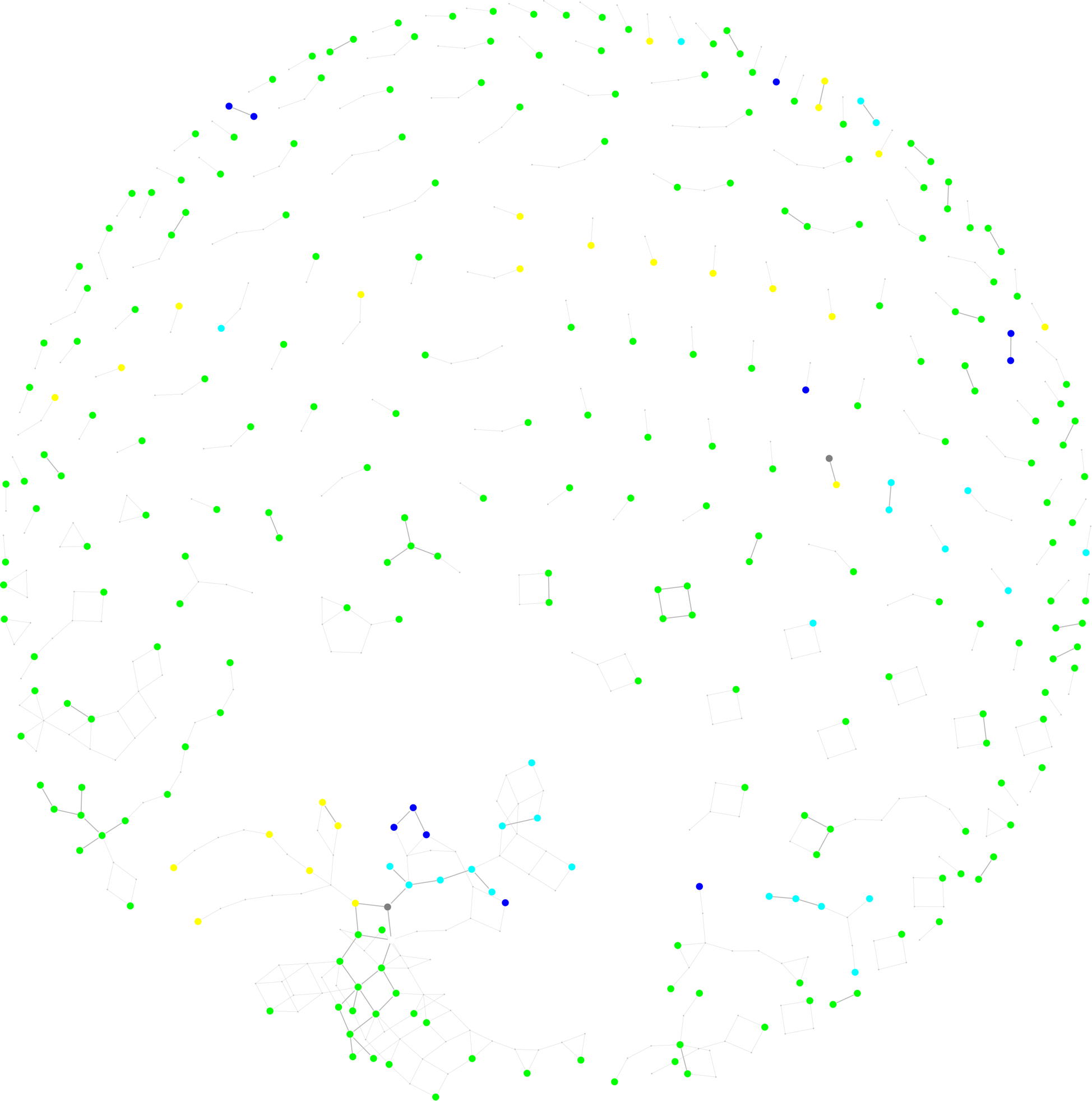

Supplement: Supplementary file 2 — Supplementary Information. [file 41598_2023_51012_MOESM2_ESM.zip › gutGH-SI/Networks/UniProt-O-glycan-networks-gut/p8121-GH-network-pp-og.jpg]

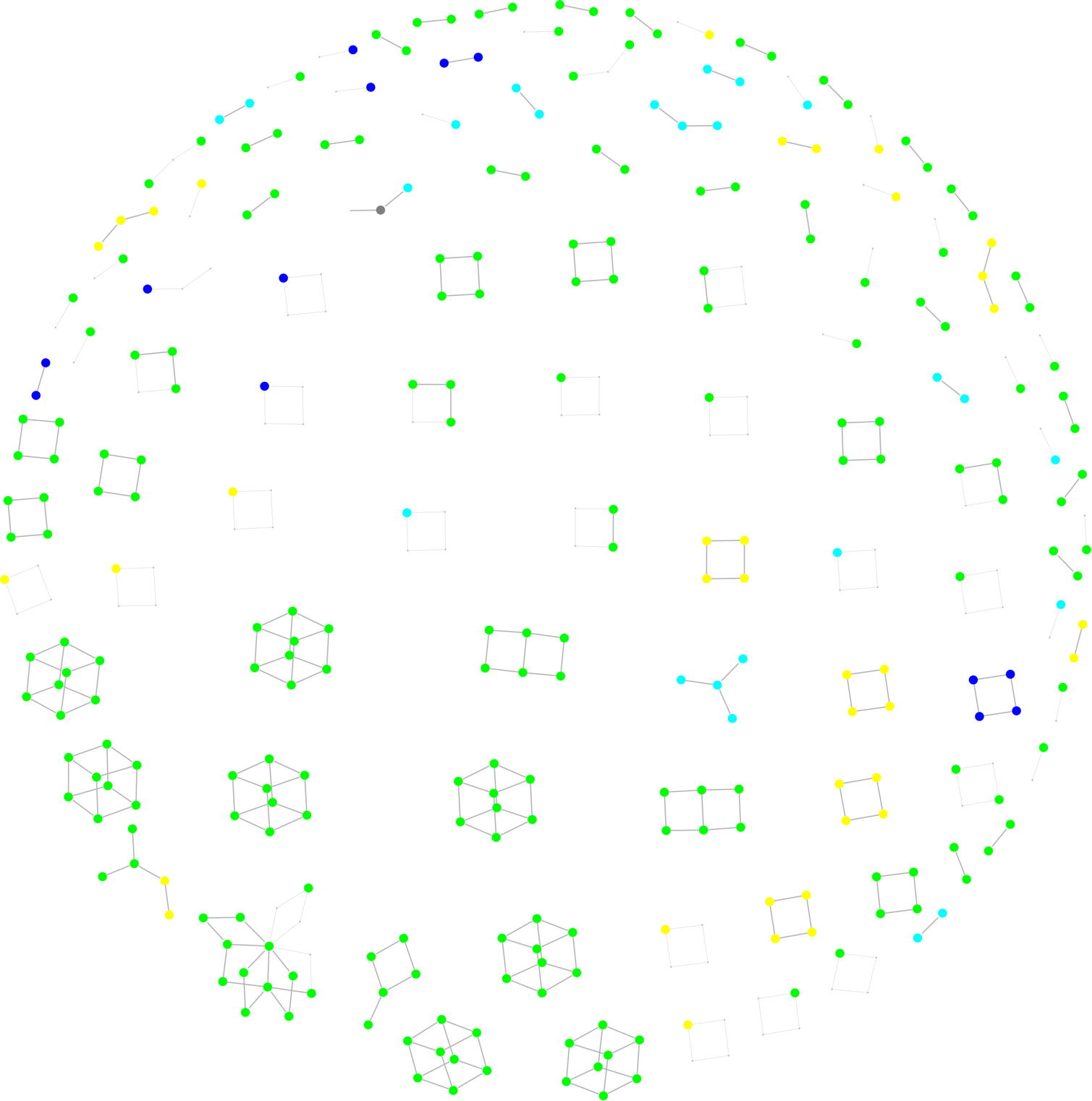

Supplement: Supplementary file 2 — Supplementary Information. [file 41598_2023_51012_MOESM2_ESM.zip › gutGH-SI/Networks/UniProt-O-glycan-networks-gut/p8124-GH-network-pp-og.jpg]

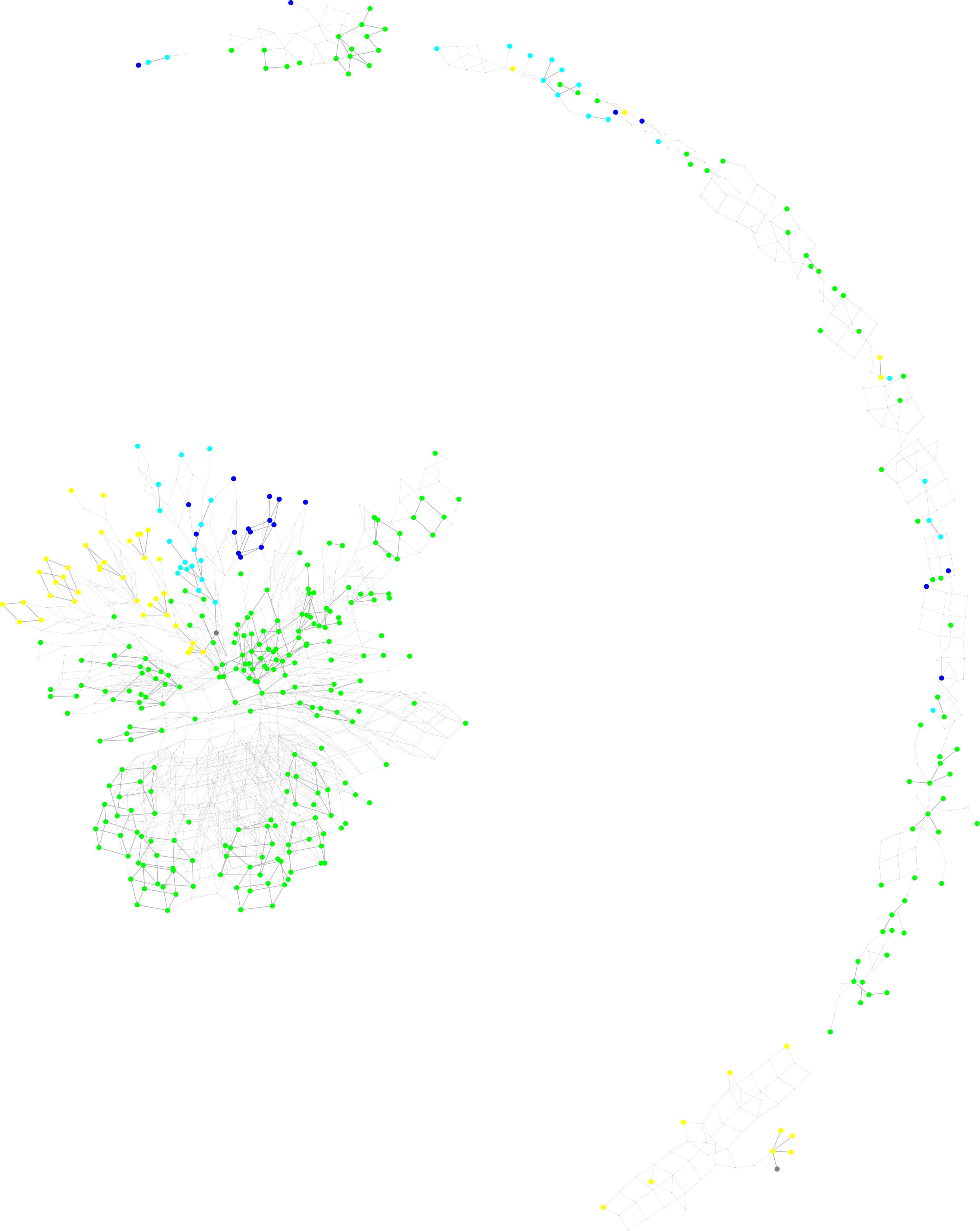

Supplement: Supplementary file 2 — Supplementary Information. [file 41598_2023_51012_MOESM2_ESM.zip › gutGH-SI/Networks/UniProt-O-glycan-networks-gut/p4360-GH-network-pp-og.jpg]

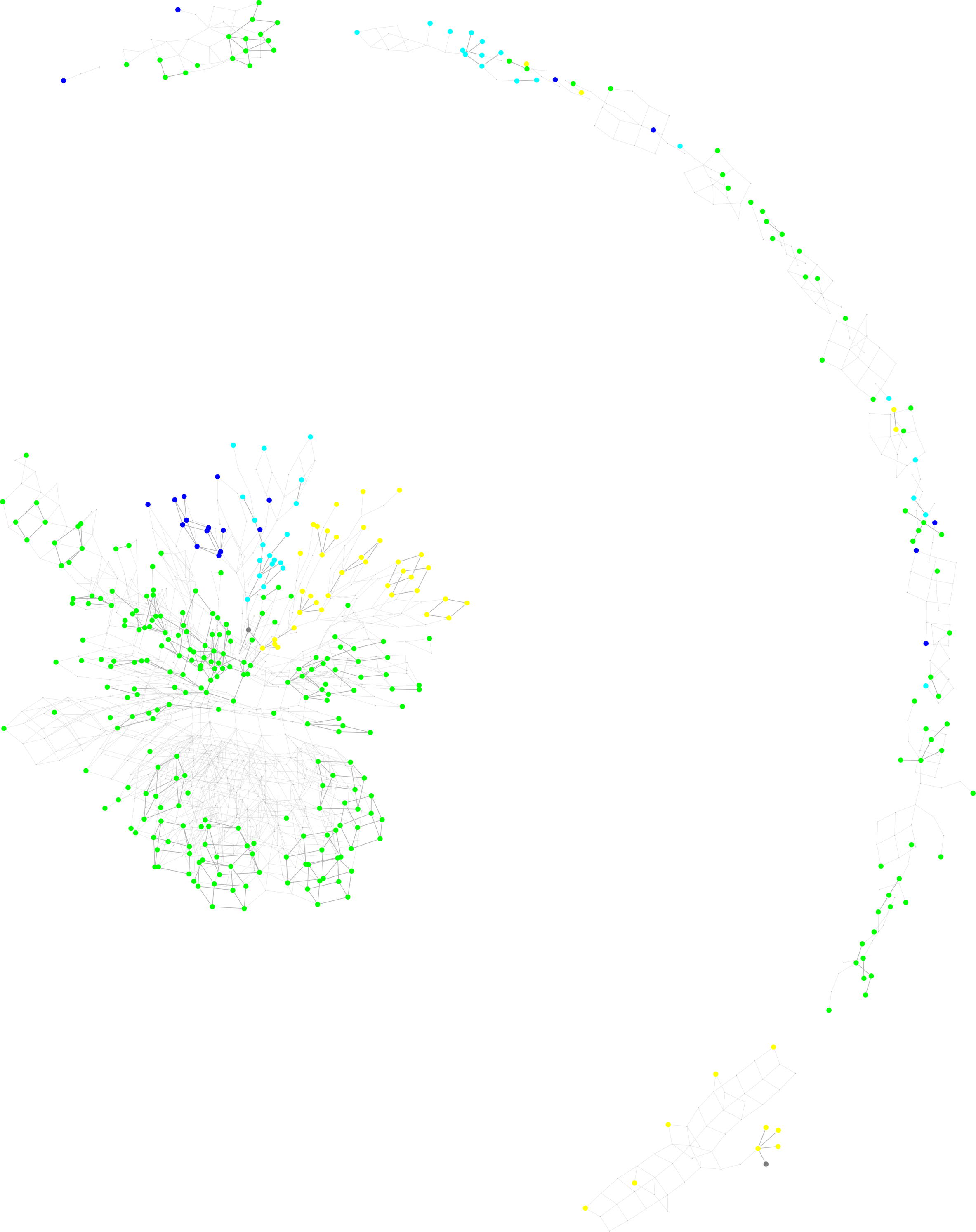

Supplement: Supplementary file 2 — Supplementary Information. [file 41598_2023_51012_MOESM2_ESM.zip › gutGH-SI/Networks/UniProt-O-glycan-networks-gut/p7944-GH-network-pp-og.jpg]

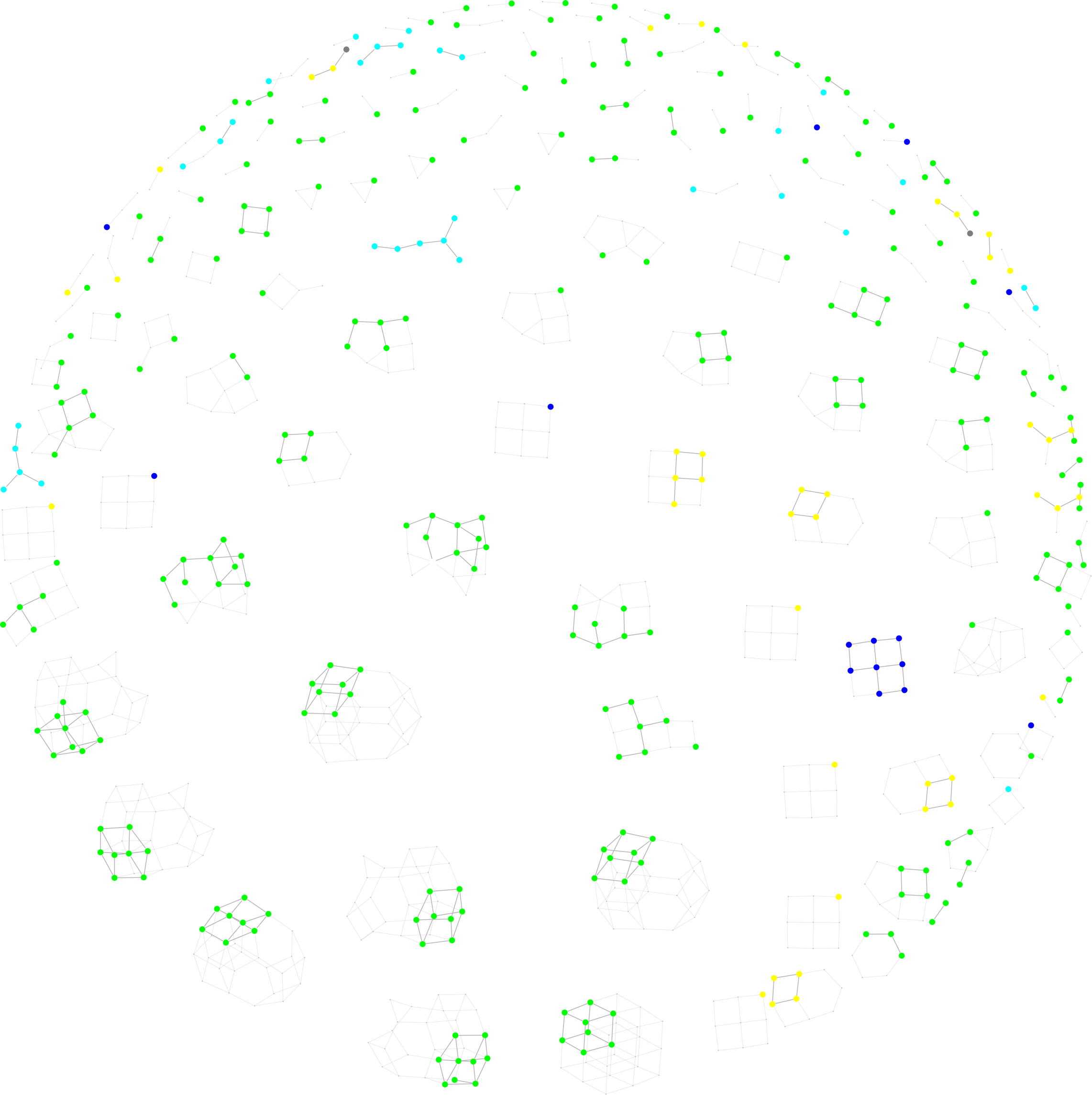

Supplement: Supplementary file 2 — Supplementary Information. [file 41598_2023_51012_MOESM2_ESM.zip › gutGH-SI/Networks/UniProt-O-glycan-networks-gut/p8184-GH-network-pp-og.jpg]

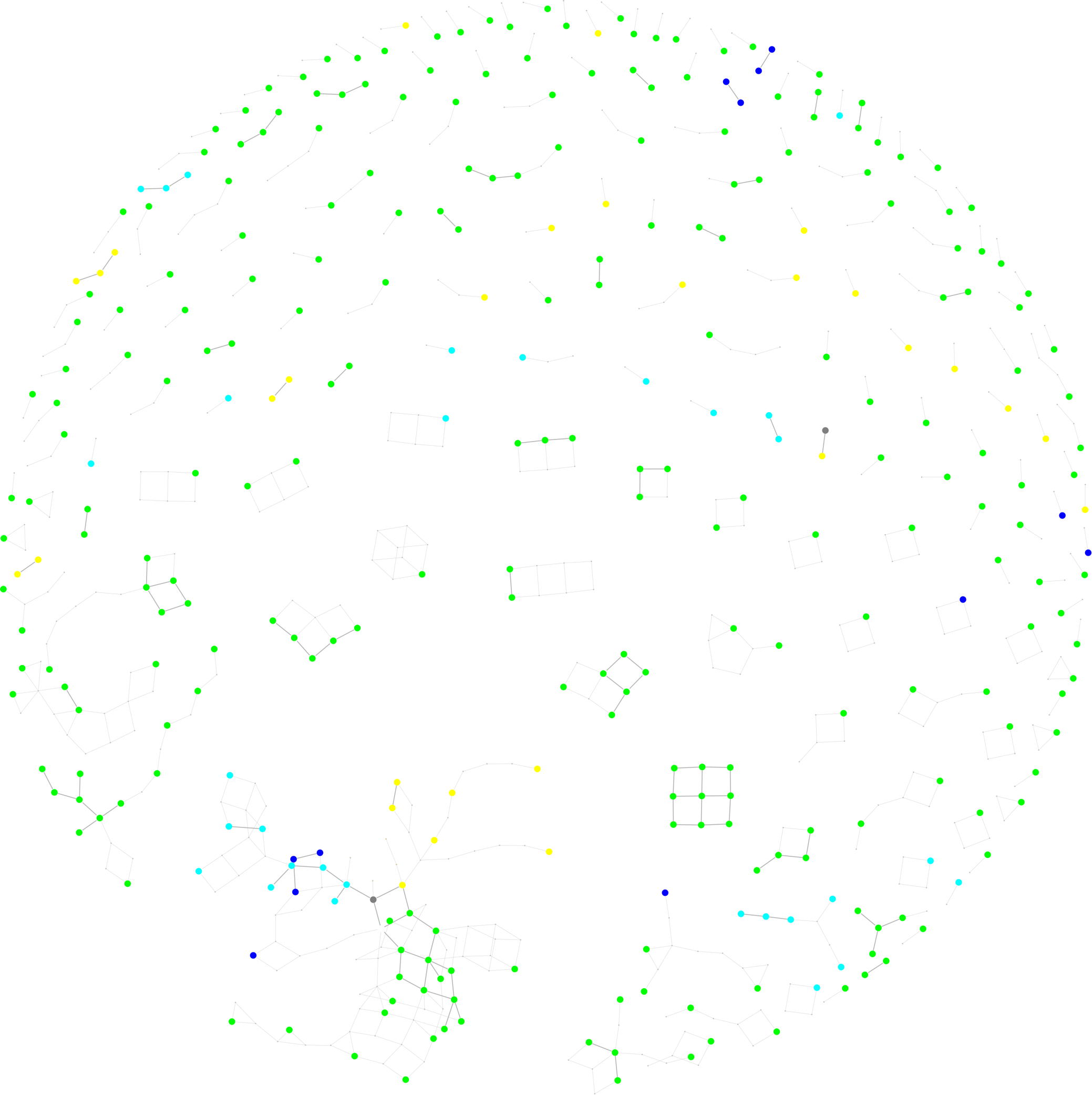

Supplement: Supplementary file 2 — Supplementary Information. [file 41598_2023_51012_MOESM2_ESM.zip › gutGH-SI/Networks/UniProt-O-glycan-networks-gut/p7985-GH-network-pp-og.jpg]

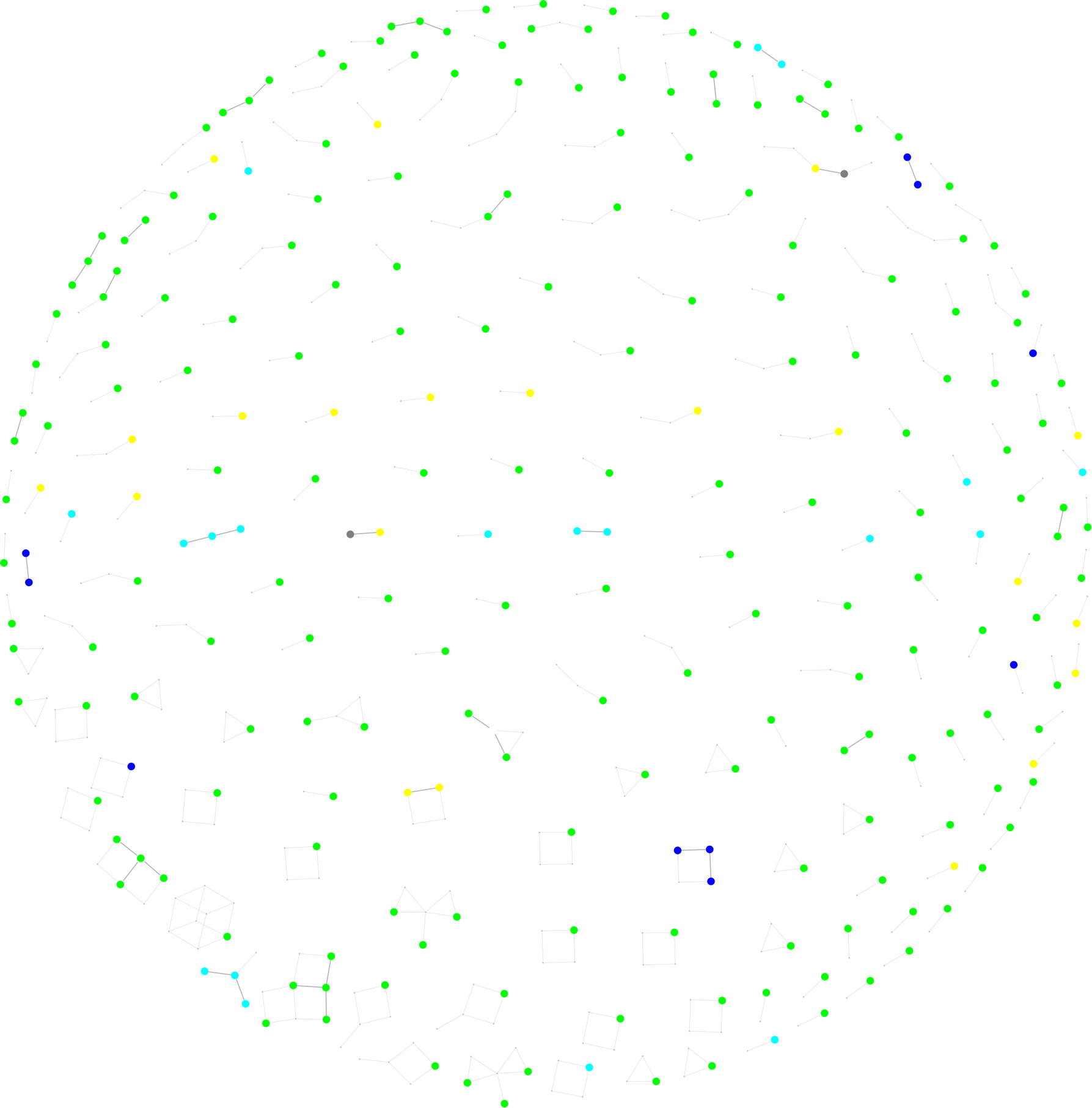

Supplement: Supplementary file 2 — Supplementary Information. [file 41598_2023_51012_MOESM2_ESM.zip › gutGH-SI/Networks/UniProt-O-glycan-networks-gut/p8059-GH-network-pp-og.jpg]

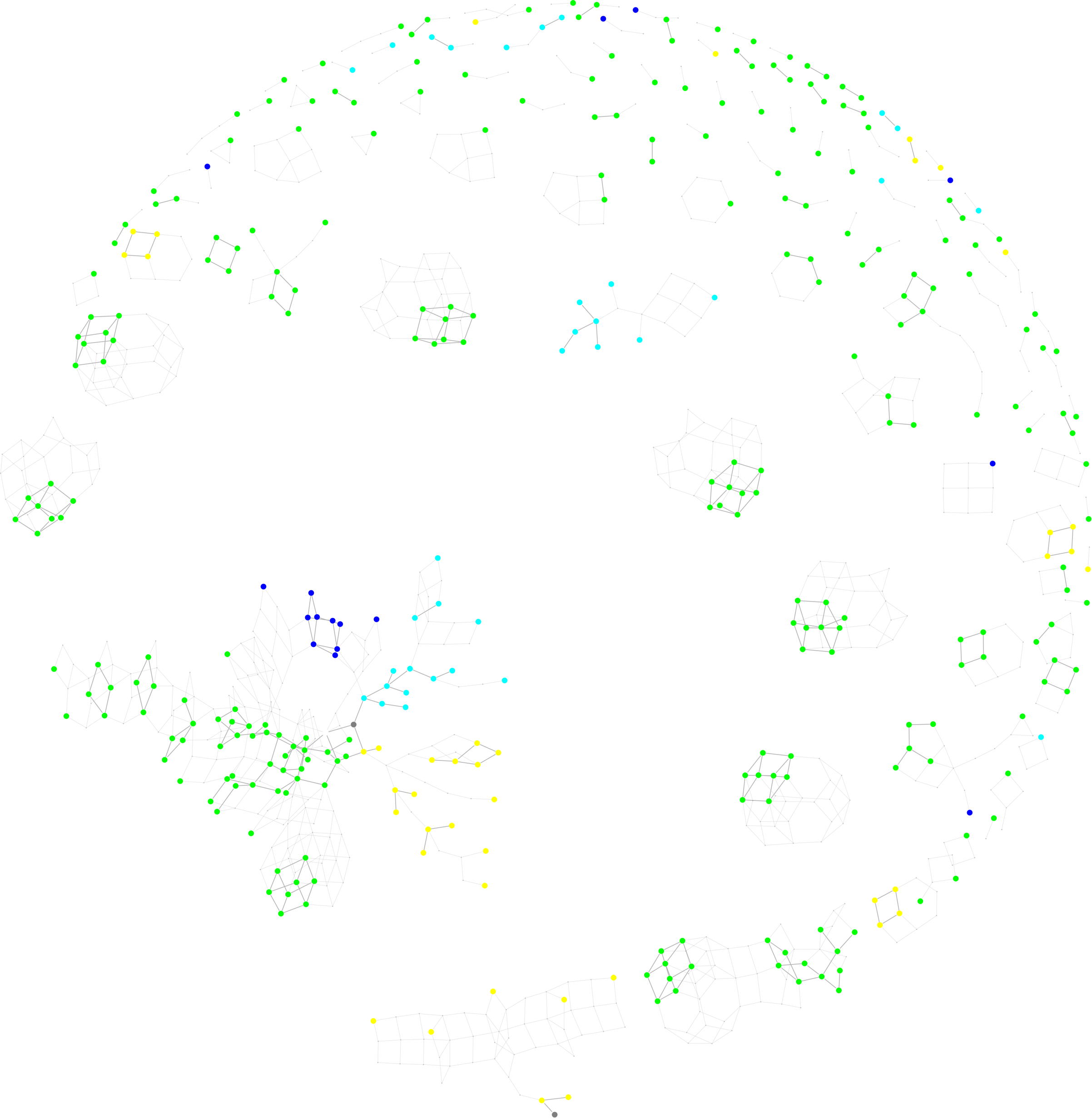

Supplement: Supplementary file 2 — Supplementary Information. [file 41598_2023_51012_MOESM2_ESM.zip › gutGH-SI/Networks/UniProt-O-glycan-networks-gut/p8120-GH-network-pp-og.jpg]

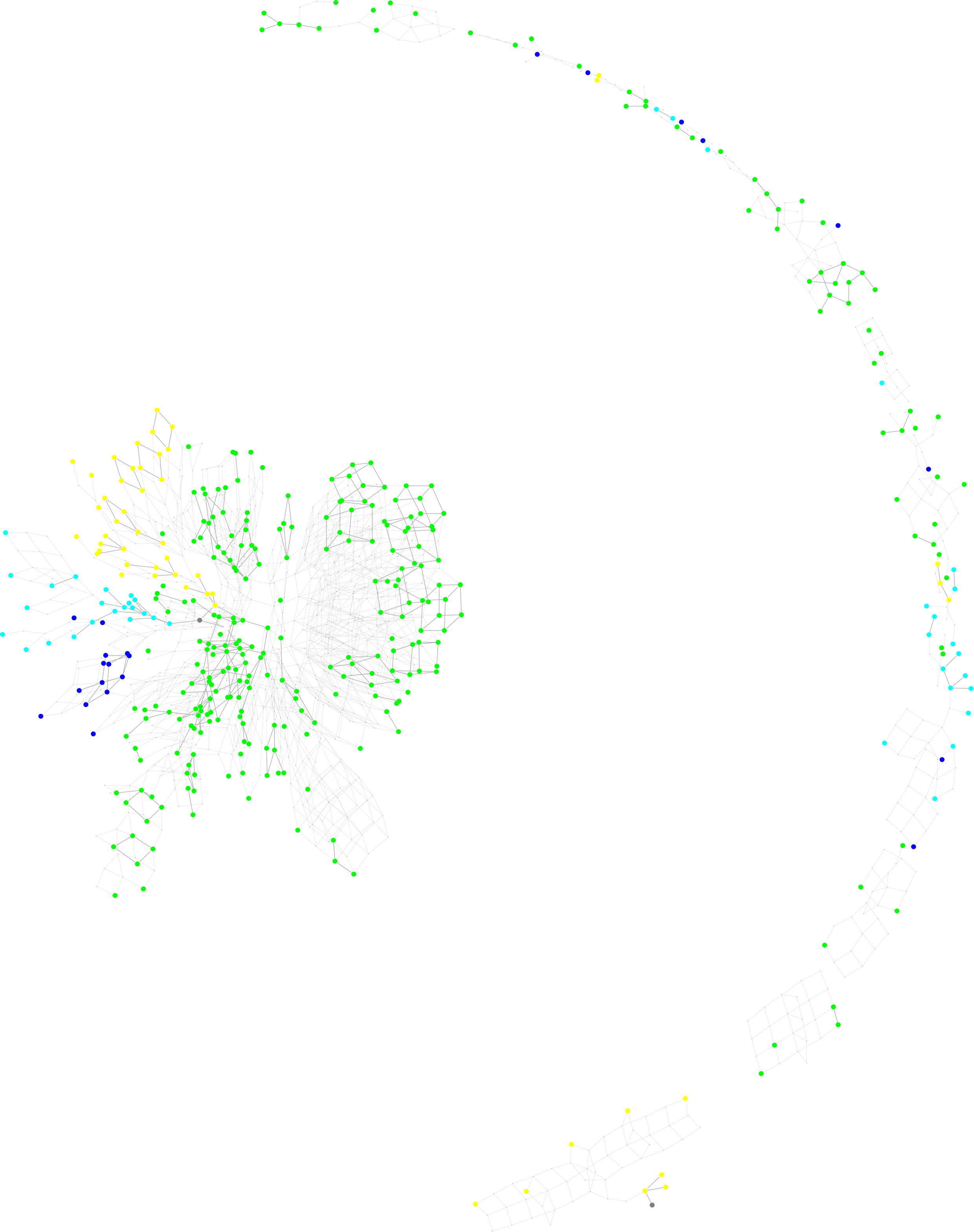

Supplement: Supplementary file 2 — Supplementary Information. [file 41598_2023_51012_MOESM2_ESM.zip › gutGH-SI/Networks/UniProt-O-glycan-networks-gut/p7952-GH-network-pp-og.jpg]

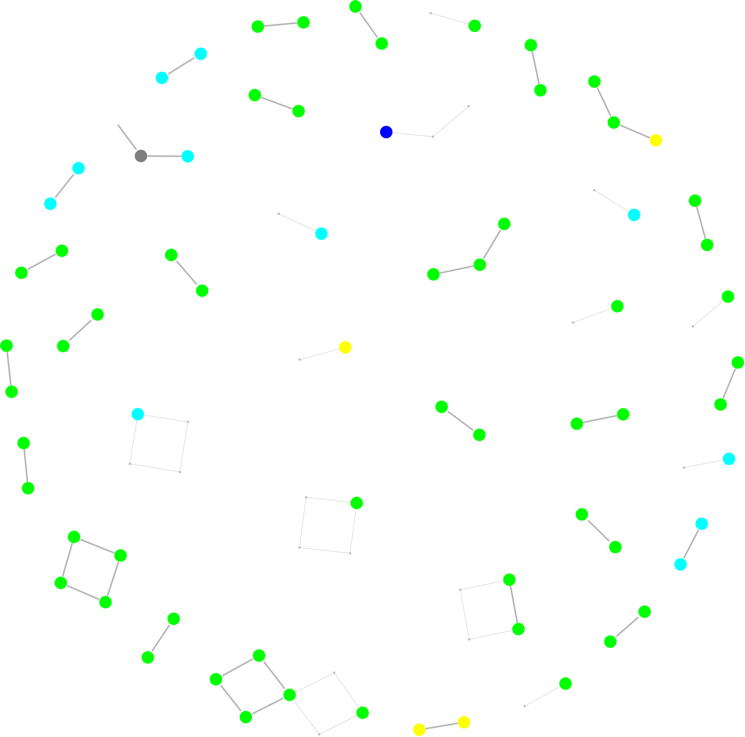

Supplement: Supplementary file 2 — Supplementary Information. [file 41598_2023_51012_MOESM2_ESM.zip › gutGH-SI/Networks/UniProt-O-glycan-networks-gut/p8125-GH-network-pp-og.jpg]

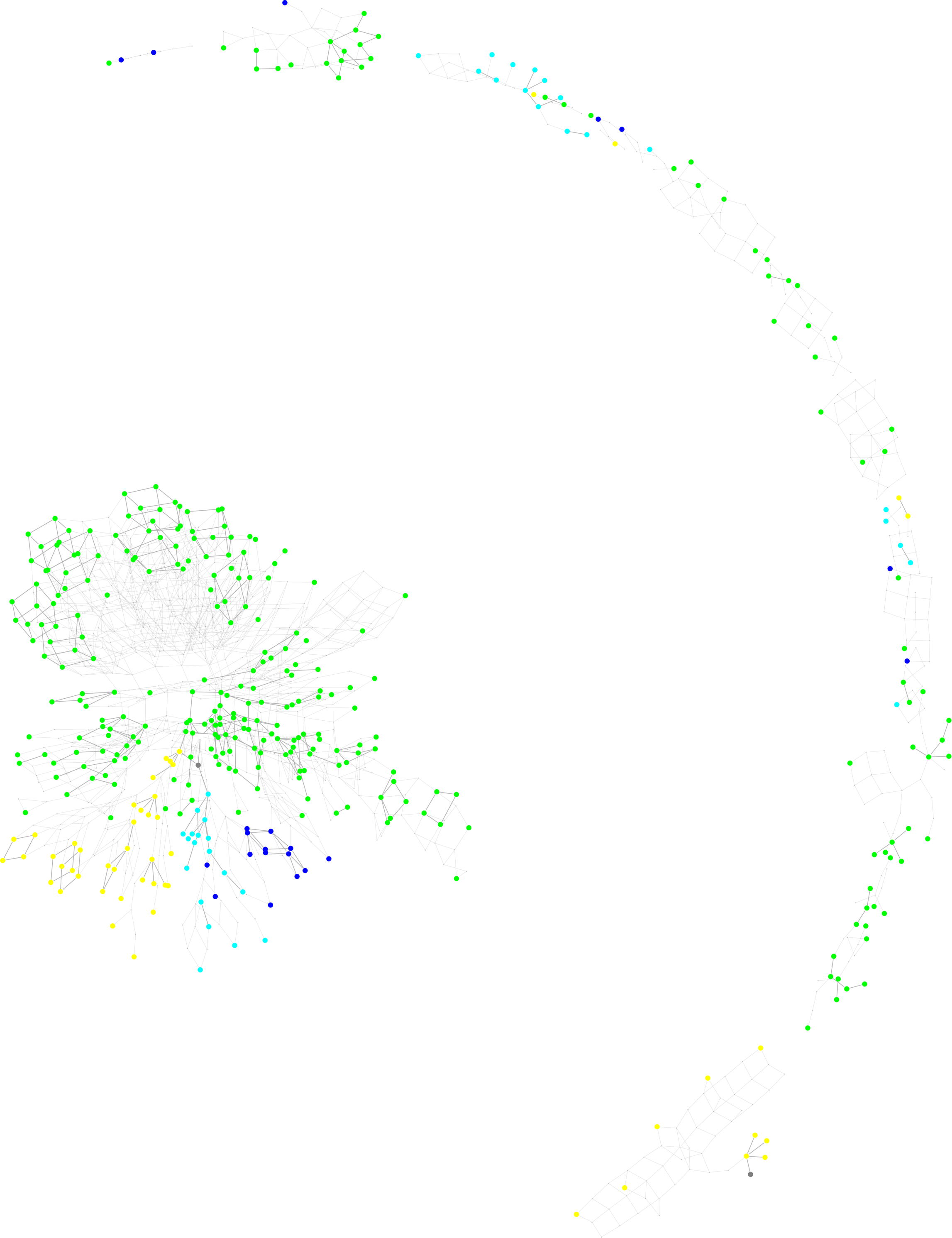

Supplement: Supplementary file 2 — Supplementary Information. [file 41598_2023_51012_MOESM2_ESM.zip › gutGH-SI/Networks/UniProt-O-glycan-networks-gut/p8072-GH-network-pp-og.jpg]

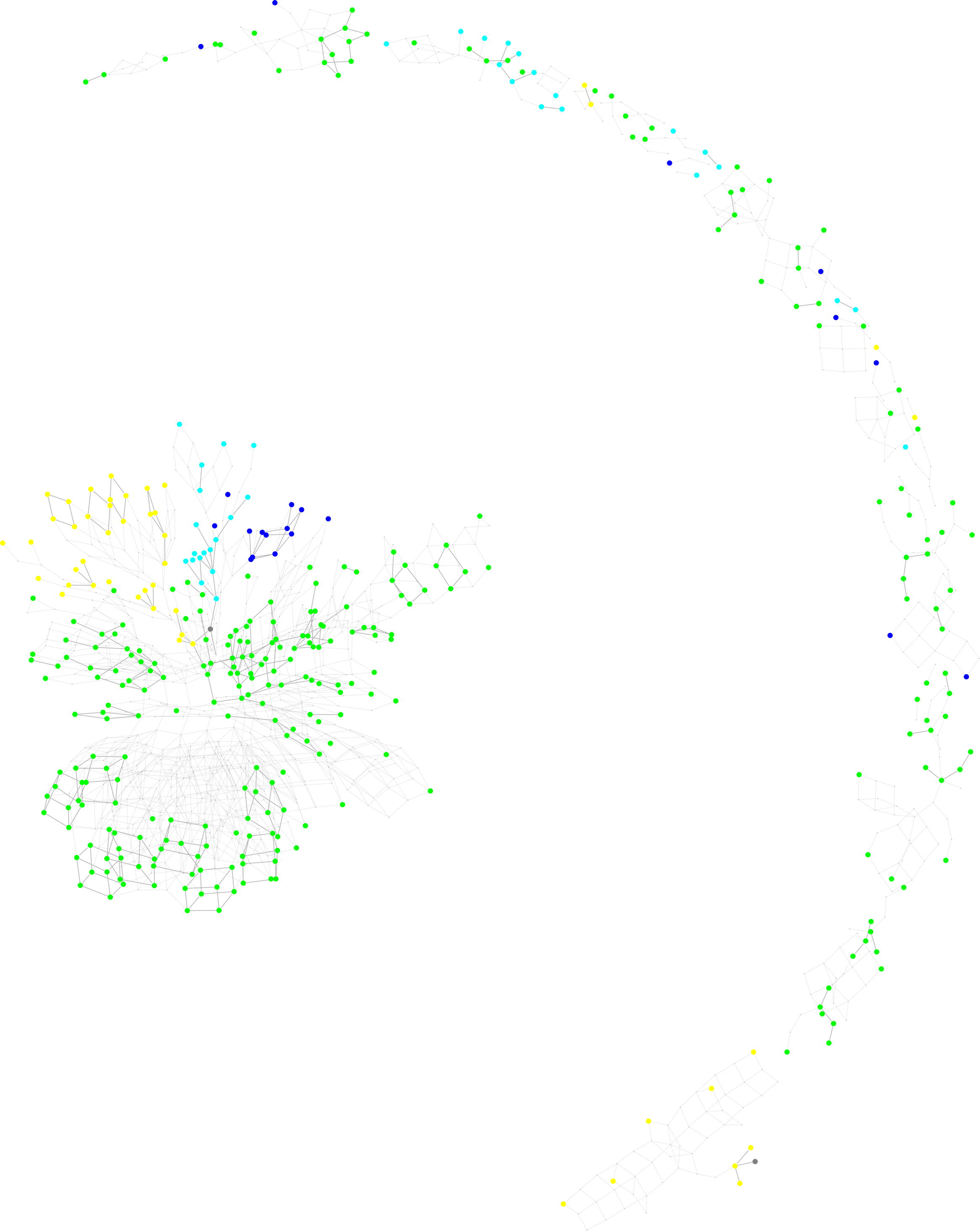

Supplement: Supplementary file 2 — Supplementary Information. [file 41598_2023_51012_MOESM2_ESM.zip › gutGH-SI/Networks/UniProt-O-glycan-networks-gut/p7960-GH-network-pp-og.jpg]

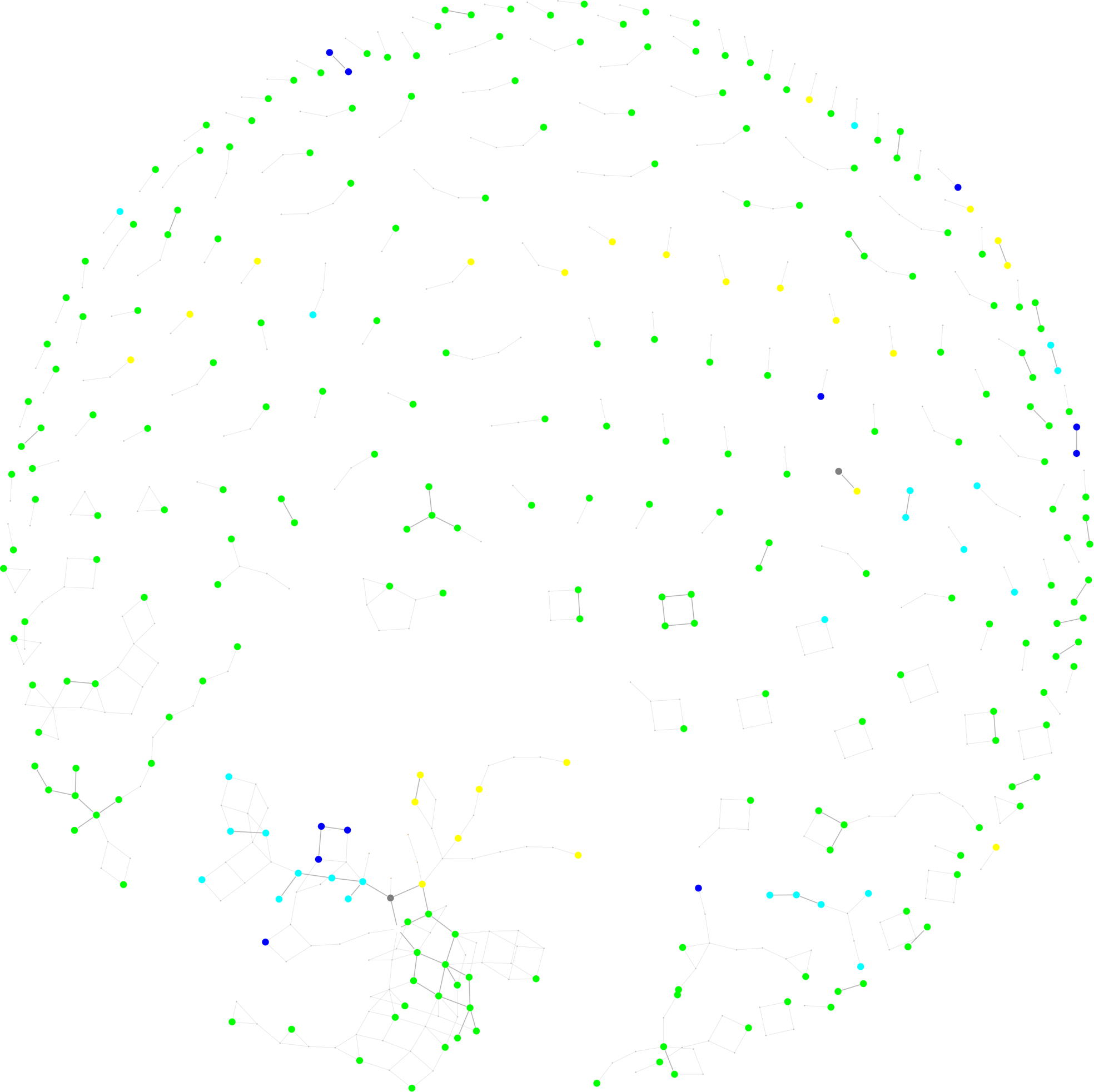

Supplement: Supplementary file 2 — Supplementary Information. [file 41598_2023_51012_MOESM2_ESM.zip › gutGH-SI/Networks/UniProt-O-glycan-networks-gut/p7993-GH-network-pp-og.jpg]

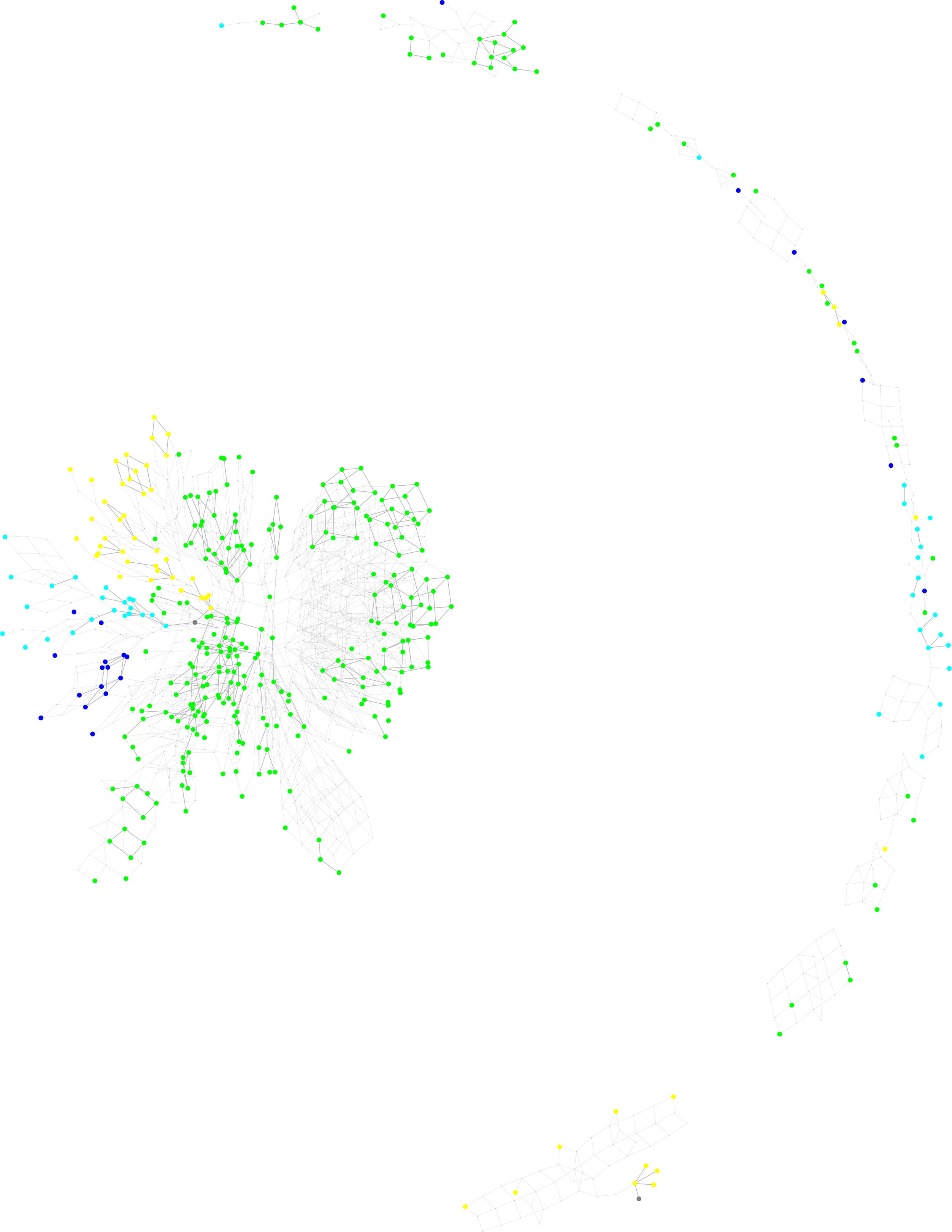

Supplement: Supplementary file 2 — Supplementary Information. [file 41598_2023_51012_MOESM2_ESM.zip › gutGH-SI/Networks/UniProt-O-glycan-networks-gut/p7936-GH-network-pp-og.jpg]

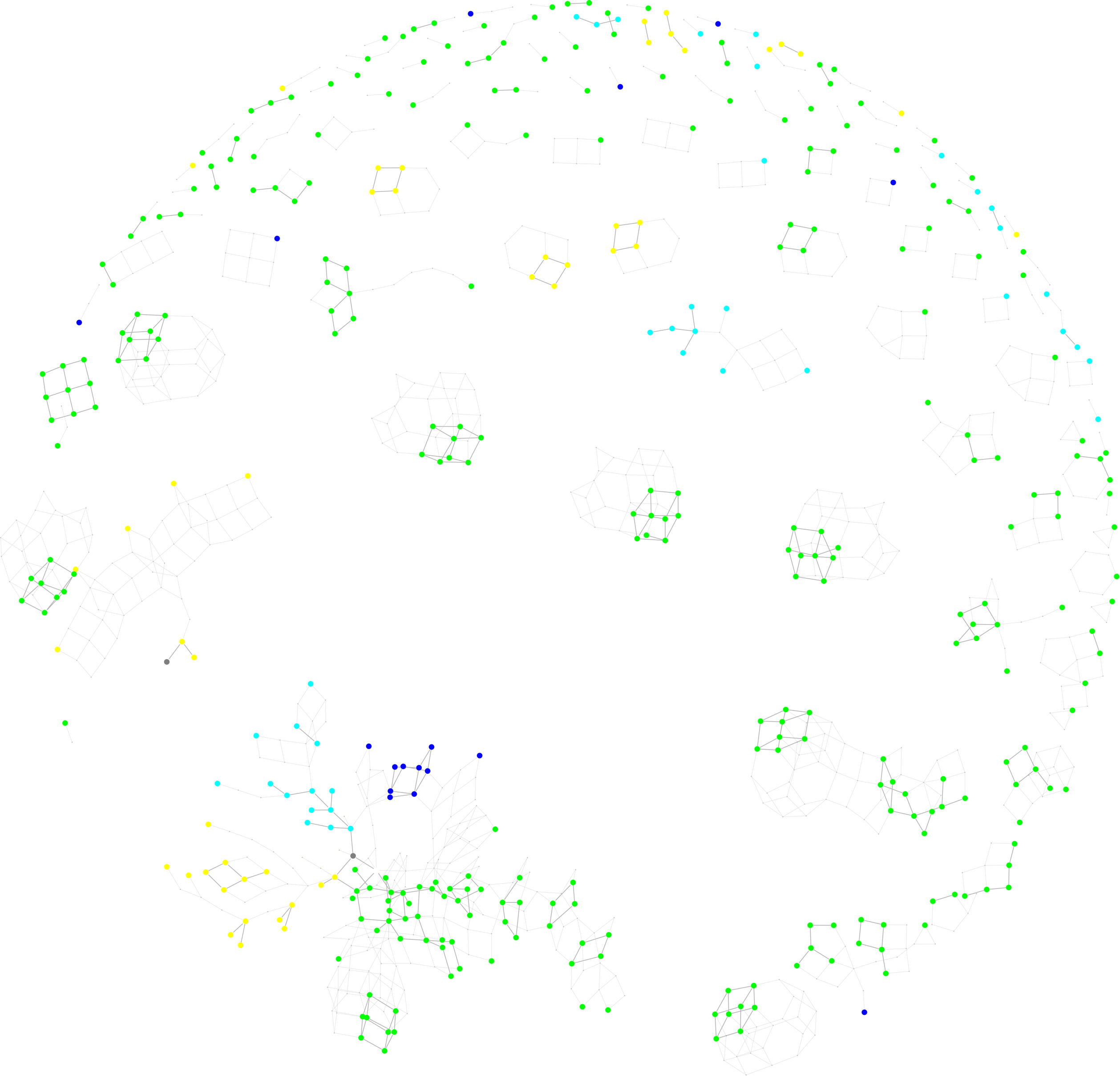

Supplement: Supplementary file 2 — Supplementary Information. [file 41598_2023_51012_MOESM2_ESM.zip › gutGH-SI/Networks/UniProt-O-glycan-networks-gut/p7984-GH-network-pp-og.jpg]

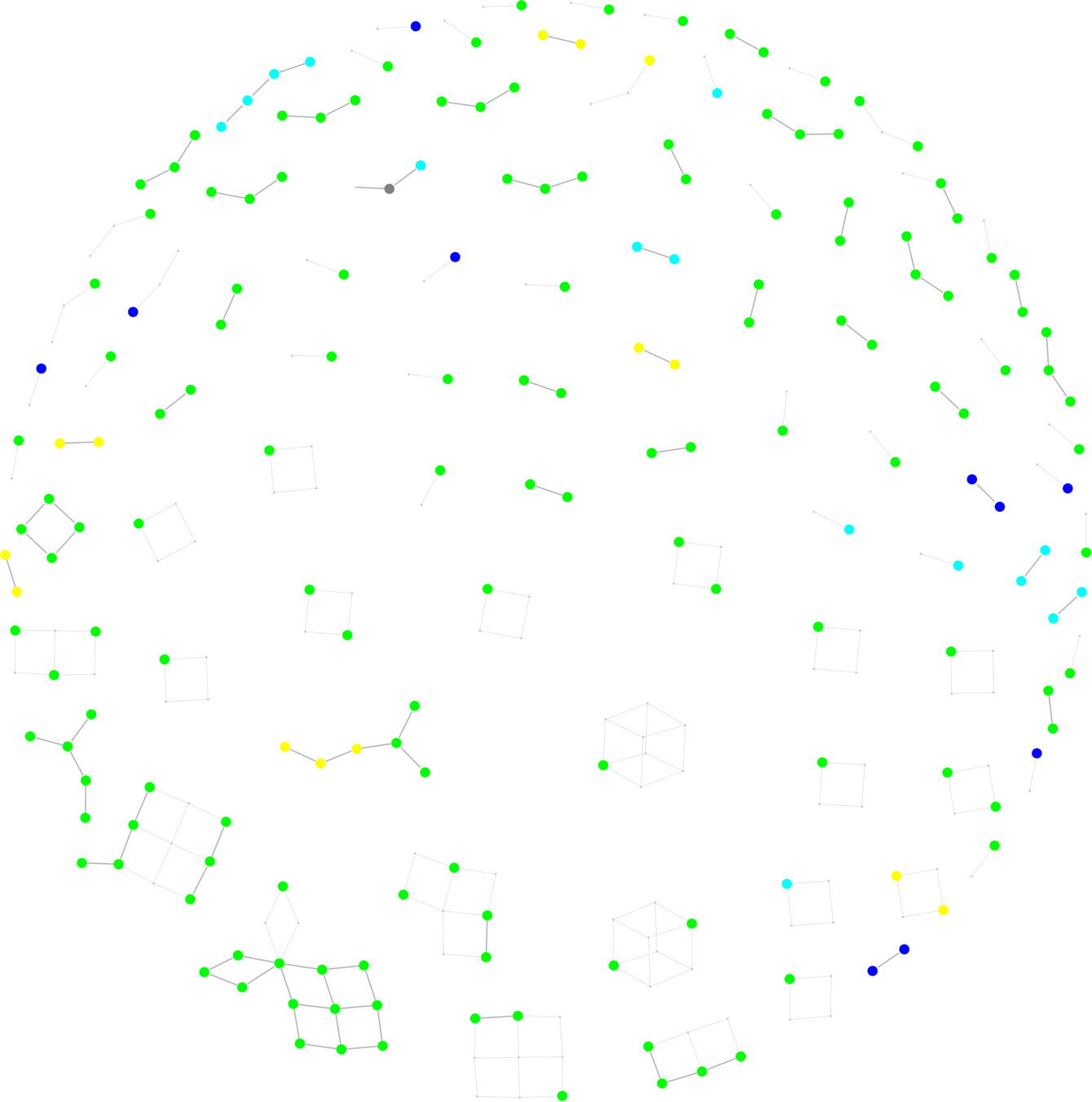

Supplement: Supplementary file 2 — Supplementary Information. [file 41598_2023_51012_MOESM2_ESM.zip › gutGH-SI/Networks/UniProt-O-glycan-networks-gut/p8093-GH-network-pp-og.jpg]

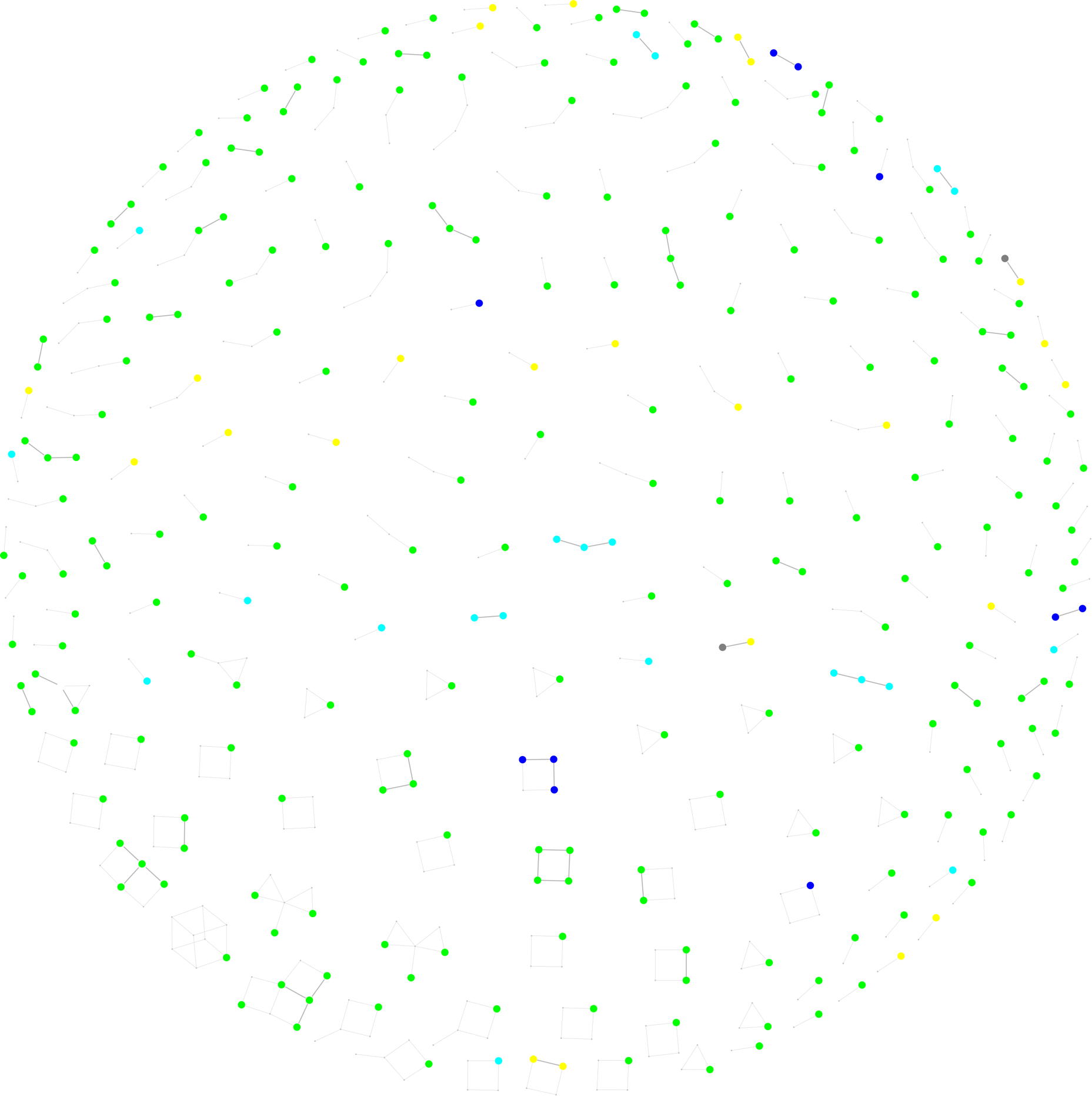

Supplement: Supplementary file 2 — Supplementary Information. [file 41598_2023_51012_MOESM2_ESM.zip › gutGH-SI/Networks/UniProt-O-glycan-networks-gut/p8185-GH-network-pp-og.jpg]
